# Genomic characterization and epidemiology of an emerging SARS-CoV-2 variant in Delhi, India

**DOI:** 10.1126/science.abj9932

**Published:** 2021-10-14

**Authors:** Mahesh S. Dhar, Robin Marwal, Radhakrishnan VS, Kalaiarasan Ponnusamy, Bani Jolly, Rahul C. Bhoyar, Viren Sardana, Salwa Naushin, Mercy Rophina, Thomas A. Mellan, Swapnil Mishra, Charles Whittaker, Saman Fatihi, Meena Datta, Priyanka Singh, Uma Sharma, Rajat Ujjainiya, Nitin Bhatheja, Mohit Kumar Divakar, Manoj K. Singh, Mohamed Imran, Vigneshwar Senthivel, Ranjeet Maurya, Neha Jha, Priyanka Mehta, Vivekanand A, Pooja Sharma, Arvinden VR, Urmila Chaudhary, Namita Soni, Lipi Thukral, Seth Flaxman, Samir Bhatt, Rajesh Pandey, Debasis Dash, Mohammed Faruq, Hemlata Lall, Hema Gogia, Preeti Madan, Sanket Kulkarni, Himanshu Chauhan, Shantanu Sengupta, Sandhya Kabra, The Indian SARS-CoV-2 Genomics Consortium (INSACOG), Ravindra K. Gupta, Sujeet K. Singh, Anurag Agrawal, Partha Rakshit, Vinay Nandicoori, Karthik Bharadwaj Tallapaka, Divya Tej Sowpati, K. Thangaraj, Murali Dharan Bashyam, Ashwin Dalal, Sridhar Sivasubbu, Vinod Scaria, Ajay Parida, Sunil K. Raghav, Punit Prasad, Apurva Sarin, Satyajit Mayor, Uma Ramakrishnan, Dasaradhi Palakodeti, Aswin Sai Narain Seshasayee, Manoj Bhat, Yogesh Shouche, Ajay Pillai, Tanzin Dikid, Saumitra Das, Arindam Maitra, Sreedhar Chinnaswamy, Nidhan Kumar Biswas, Anita Sudhir Desai, Chitra Pattabiraman, M. V. Manjunatha, Reeta S. Mani, Gautam Arunachal Udupi, Priya Abraham, Potdar Varsha Atul, Sarah S. Cherian

**Affiliations:** 1 National Centre for Disease Control, Delhi, India.; 2 CSIR-Institute of Genomics and Integrative Biology, New Delhi, India.; 3 Academy for Scientific and Innovative Research, Ghaziabad, India.; 4 Medical Research Council (MRC) Centre for Global Infectious Disease Analysis, Jameel Institute, School of Public Health, Imperial College London, London, UK.; 5 Department of Mathematics, Imperial College London, London, UK.; 6 Section of Epidemiology, Department of Public Health, University of Copenhagen, Copenhagen, Denmark.; 7 Department of Medicine, Cambridge Institute of Therapeutic Immunology and Infectious Disease (CITIID), University of Cambridge, Cambridge, UK.; 8 Africa Health Research Institute, KwaZulu-Natal, South Africa.

## Abstract

Delhi, the national capital of India, experienced multiple severe acute respiratory syndrome coronavirus 2 (SARS-CoV-2) outbreaks in 2020 and reached population seropositivity of >50% by 2021. During April 2021, the city became overwhelmed by COVID-19 cases and fatalities, as a new variant, B.1.617.2 (Delta), replaced B.1.1.7 (Alpha). A Bayesian model explains the growth advantage of Delta through a combination of increased transmissibility and reduced sensitivity to immune responses generated against earlier variants (median estimates: 1.5-fold greater transmissibility and 20% reduction in sensitivity). Seropositivity of an employee and family cohort increased from 42% to 87.5% between March and July 2021, with 27% reinfections, as judged by increased antibody concentration after a previous decline. The likely high transmissibility and partial evasion of immunity by the Delta variant contributed to an overwhelming surge in Delhi.

## Deadly surge in Delhi

In the spring of 2021, Delhi, India experienced a wave of coronavirus cases that overwhelmed healthcare services despite the population showing a high level of immune positivity. Dhar *et al*. collated a mixture of serosurveillance, quantitative polymerase chain reaction, and genomic data, finding that waves of variants had passed through the Delhi population during 2020 and 2021. The alpha (B.1.1.7) variant dominated in March 2021 and was rapidly replaced by the delta (B.1.617.2) variant in April and May 2021. The delta variant outcompeted its predecessors by mutations that enhanced replication, immune evasion, and host receptor avidity, thus increasing transmissibility, reinfection, and vaccination breakthrough. —CA

## Teaser

A surge of COVID-19 cases and fatalities peaked in May 2021 as the highly transmissible and immune evasive delta variant replaced the alpha variant.

**Fig. 1.**
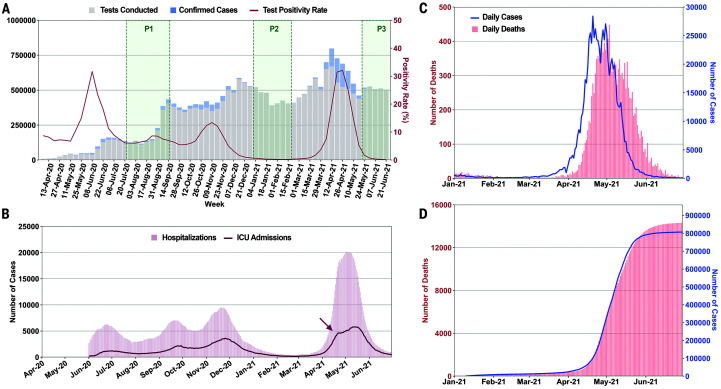
Multiple surges of SARS-CoV-2 infections in Delhi with an overwhelming outbreak in April and May 2021. (**A**) Weekly tests, confirmed cases, and test positivity rate in Delhi from April 2020 to June 2021. Sample collection period for CSIR serosurveys is marked as P1 to P3. (**B**) Number of hospitalizations and ICU admissions plotted on a daily basis from June 2020 to June 2021. The arrow marks the possible saturation of ICU capacity (*3*). (**C**) Daily cases and daily deaths from January 2021 to June 2021. (**D**) Time-advanced and scaled cumulative cases, fitted to cumulative deaths. Time advancement of cumulative reported cases by 8 days was done for maximal coincidence with scaled cumulative deaths. Case fatality ratio = averaged scaling factor (cumulative deaths / time-advanced cumulative cases). Mean ± SD, 0.019 ± 0.003.

After escaping relatively unscathed during the first wave of the COVID-19 pandemic, India witnessed a ferocious second COVID-19 wave starting in March 2021 that accounted for about half of global cases by the first week of May. Severe acute respiratory syndrome coronavirus 2 (SARS-CoV-2) had spread widely throughout India during the first wave, with the third national serosurvey in January 2021 finding that 21.4% of adults and 25.3% of 10- to 17-year-old adolescents were seropositive (*1*). Delhi, the national capital, was not included in the national serosurvey but had undergone multiple periods of high transmission in 2020 (Fig. 1A). In a district-wise stratified serosurvey conducted by the Delhi government in January 2021, overall seropositivity was reported to be 56.1% [95% confidence interval (CI), 55.5 to 56.8%], ranging from 49.1 to 62.2% across 11 districts (*2*). This level of seropositivity was expected to confer some protection against future outbreaks.

Despite high seropositivity, Delhi was among the most-affected cities during the second wave. The rise in new cases was exceptionally rapid in April 2021, increasing from ~2000 to 20,000 between 31 March and 16 April. This was accompanied by a rapid rise in hospitalizations and intensive care unit (ICU) admissions (Fig. 1B). In this emergency situation with saturated bed occupancy by 12 April, major private hospitals were declared by the state as COVID-care-only facilities, and senior medical students, including those from branches of alternative medicine, were pressed into service (*3*). Deaths rose proportionately (Fig. 1C), and the case fatality ratio (CFR), estimated as the scaling factor between time-advanced cases and deaths (Fig. 1D), was stable (mean, SD; 1.9, 0.3%). Population spread of SARS-CoV-2 is underestimated by test-positive cases alone (*1*, *2*). To better understand the degree of spread and the factors leading to the unexpectedly severe outbreak, we used all available data, including testing, sequencing, serosurveys, and serially followed cohorts.

**Fig. 2.**
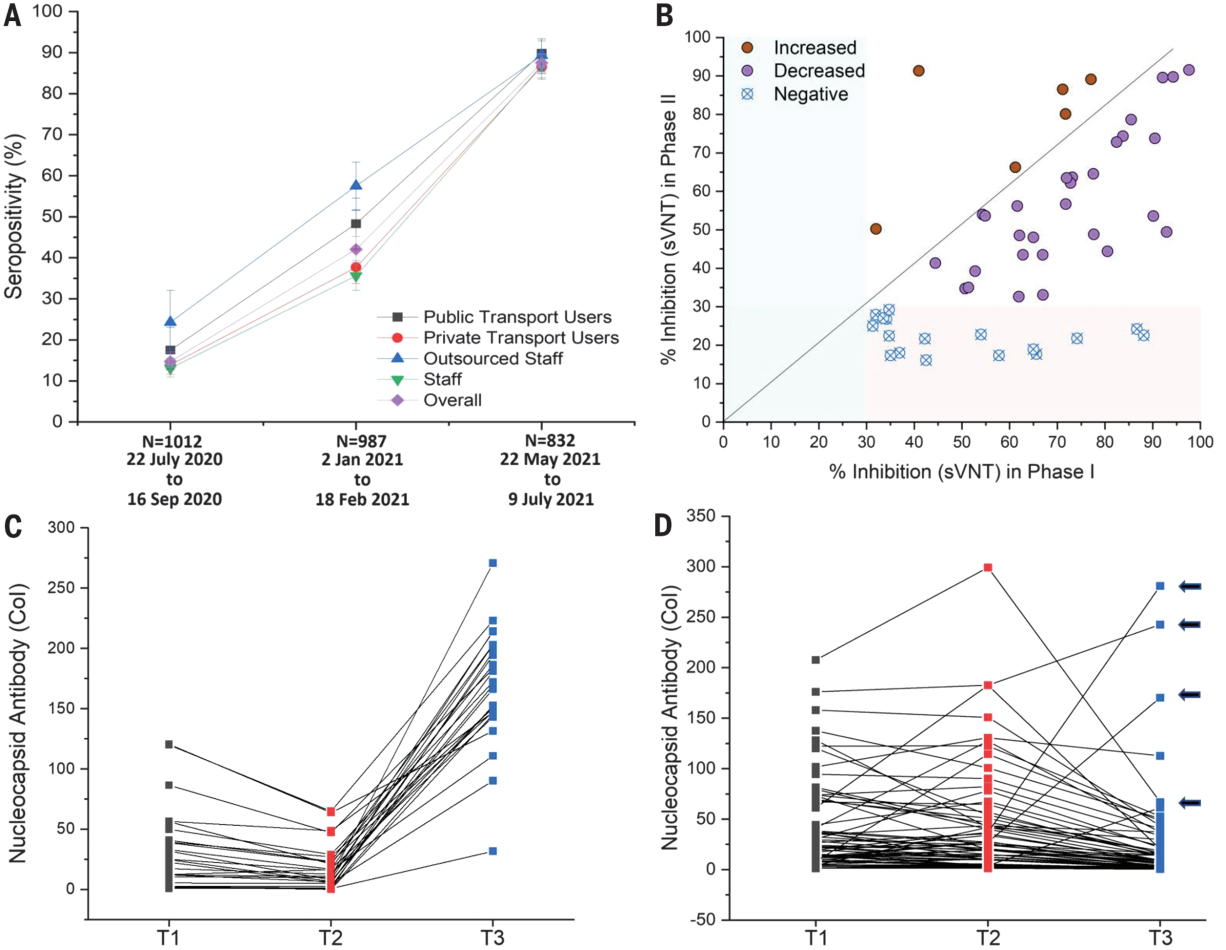
Serological estimates of prior infections, preexisting immunity, and new infections for the April and May 2021 outbreak. (**A**) Seropositivity in the CSIR cohort, subdivided by nature of employment and use of public transport, plotted for different time periods (phase I to phase III, proportion ± 95% CI). Details are provided in table S1. (**B**) Variability and temporal decline in neutralization capacity estimated by sVNT assay between phases I and II (*n* = 52 subjects). (**C**) Serial antibody concentration measurements in initially seropositive subjects (*n* = 91). Pattern suggestive of reinfections is shown (decline followed by rise, *n* = 25). (**D**) Remaining data (*n* = 66 subjects), with four indeterminate reinfection cases indicated with arrows. Antibody concentration is reported in multiples of the assay cutoff index value (CoI).

In the absence of finely resolved or serial data from national and state surveys, we focused on data for Delhi participants of a national serosurvey of the Council of Scientific and Industrial Research (CSIR, India) employees and their family members (Fig. 2A and table S1). Samples were initially collected from the end of July to mid-September 2020 (phase I). Subsequently, second and third surveys were done in January and February (phase II) and the end of May to early July 2021 (phase III), bracketing the time period of interest. The cohort details and serosurvey methodology have been previously published (*4*).

Infection was determined by anti-nucleocapsid assay, which is not affected by immunization with spike protein–based vaccines. The presence of neutralizing antibodies to wild-type SARS-CoV-2 spike protein was estimated by a surrogate viral neutralization test (sVNT, Genscript). Previous results from the full cohort have been comparable to government serosurveys, but Delhi cohort values have been slightly lower. This may be due to an overrepresentation of members with the ability to reduce exposure and avoid public transport (Fig. 2A). Within these limitations, the Delhi cohort showed a rise in seropositivity from 14.7% in phase I (95% CI, 12.6 to 17.0%) to 42.1% in phase II (95% CI, 39.0 to 45.2%). About one-third of neutralizing antibody–positive subjects at phase I became neutralizing antibody–negative by phase II, with most showing declining inhibition on sVNT assays (Fig. 2B) (*5*). Phase III seropositivity increased to 87.5% (95% CI, 85.0 to 89.7%) among unvaccinated subjects. New infections between March and June 2021 are thus likely to have vastly exceeded known cases. Among 91 previously infected subjects with serial measurements at three time points, including phase III (T3), 25 (27.5%; 95% CI, 18.4 to 37.5%) had a pattern of declining antibody concentration between T1 and T2, followed by a sharp rise at T3, indicative of reinfection (Fig. 2C). Confirmation of reinfection by either reverse transcription polymerase chain reaction (RT-PCR) (*n* = 8) or symptomatic illness (*n* = 2) was available for 10 of the 25 subjects. No severe illness or hospitalization was reported in reinfections.

**Fig. 3.**
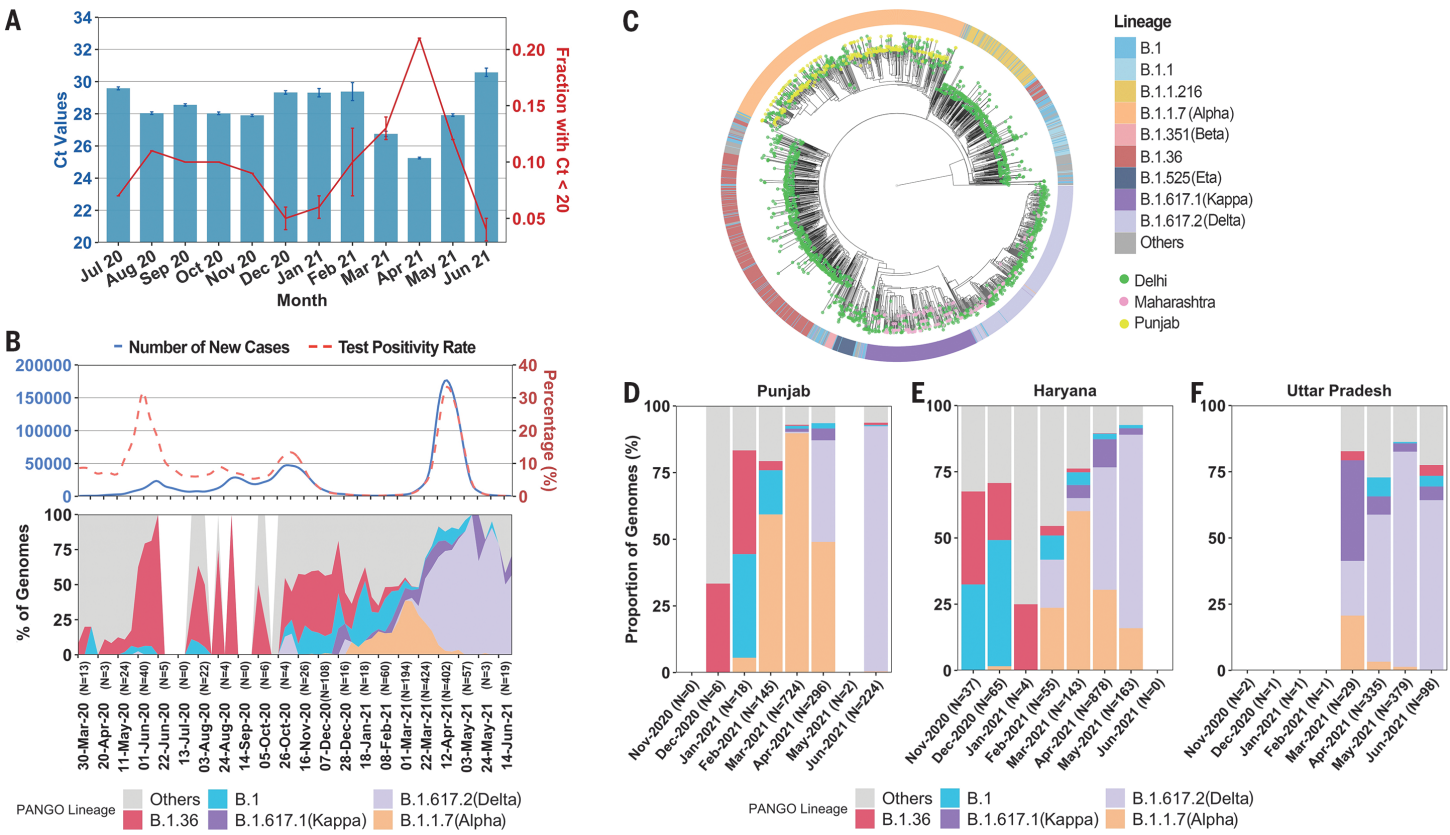
Genomic-epidemiologic correlations. (**A**) Time trends of Ct values (mean ± SE) and high viral load samples (proportion ± SE) for *Orf1* gene (E gene data, fig. S1). (**B**) Smoothed graph of main lineages in Delhi from March 2020 to May 2021 in biweekly increments. New cases and TPR are aligned and plotted on the same timeline. (**C**) Phylogenetic analysis for variant of concern (VOC) strains between Delhi and states (Punjab and Maharashtra) with known VOC outbreaks before April 2021. Further analysis suggesting a superspreading event for Alpha is shown in fig. S3. (**D** to **F**) Month-wise proportions of different lineages (*n* > 3 sequenced samples) in states surrounding Delhi. Additional data are shown in figs. S4 and S5.

Time periods of increased transmission were associated with declining RT-PCR cycle threshold (Ct) values (Fig. 3A and fig. S1), attributable to a higher proportion of recently infected individuals with high viral loads (*6*). However, the Ct decline was far greater in April 2021 (dCt, SE: −4.06, 0.27; *P* < 0.001) than seen previously (November 2020 dCt: ~−1.5). Comparing April 2021 with November 2020, high viral load samples (Ct < 20) doubled in clinical samples (*P* < 0.001) and nearly doubled in campus surveillance testing data, where most positives were from recently infected individuals [15% (*n* = 297) versus 9% (*n* = 358); *P* = 0.02].

Genome analysis trends, in representative samples drawn from the general population over the same period, showed seeding and expansion of B.1.1.7 (Alpha), B.1.617.1 (Kappa), and B.1.617.2 (Delta) lineages in 2021, with Delta becoming the dominant lineage in Delhi during April (Fig. 3B). The proportion of the Delta variant was strongly correlated to the rise in cases and health care stress (fig. S2). Overall, the genomic and epidemiological data were most consistent with the hypothesis that a new variant with higher infectivity, Delta, was driving the unexpected overwhelming surge in Delhi. Recent in vitro data supports the possibility of a higher replication rate for Delta, thereby explaining potentially higher viral loads in RT-PCR data and greater transmissibility (*7*).

We further investigated the sequence of seeding and spread of the Alpha, Kappa, and Delta variants of SARS-CoV-2. Phylogenetic analysis showed common origins between the Alpha variants in Delhi and Punjab, and between the Kappa or Delta variants in Delhi and Maharashtra, where Kappa and Delta were first sequenced (Fig. 3C and fig. S3). There was a recurring pattern of initial smaller outbreaks with Alpha, followed by larger outbreaks coinciding with Alpha-to-Delta transition across all neighboring states (fig. S4). The substantial relative growth advantage of Delta was explored in terms of transmissibility and/or immune escape. The rise of Delta, but not other lineages, was temporally coincident with a rise in the test positivity rate (TPR) and new cases during the surge (fig. S5). While overall vaccination levels were only about 5% in Delhi, most health care workers had received one or two doses of ChAdOx1-nCov19 (AstraZeneca/Serum Institute, India) or BBV152 (Bharat Biotech, India) (*8*, *9*). We sequenced 24 breakthrough infections starting at least 1 week after the first dose, collected between 22 March and 28 April 2021 at the National Centre for Disease Control (NCDC). The ratio of Delta to non-Delta lineages was 850:1211 from 20 March to 30 April. In contrast, the ratio of Delta to non-Delta lineages was 13:3 in 16 breakthroughs after one dose and 7:1 in eight breakthroughs after the second dose of vaccine. Although the small sample size and lack of a formal control group preclude definitive analysis, estimated higher odds for Delta in vaccination breakthroughs (odds ratio, 7.1; 95% CI, 2.4 to 20.9) corroborate other reports of reduced vaccine effectiveness against Delta (*10*).

To better characterize how the properties of Delta might differ from other SARS-CoV-2 lineages previously circulating in the city, we used a Bayesian model of SARS-CoV-2 transmission and mortality that simultaneously models the dynamics of two categories of virus (B.1.617.2 and non-B.1.617.2) (*11*), while also explicitly incorporating natural waning of immunity derived from prior infection, with the duration of immunity consistent with the results of recent longitudinal cohort studies (*12*, *13*). Details of the model and input data are given in the supplementary methods. Briefly, the model is fitted to COVID-19 mortality data, genomic sequence data presented here and from GISAID [with Phylogenetic Assignment of Named Global Outbreak Lineages ( PANGOLIN) classification] (*14*–*16*), and serological data presented alongside an additional longitudinal serosurvey carried out in the city from July to December 2020 (*17*). Substantial uncertainties remain as to the date of introduction of B.1.617.2 into Delhi and the degree of COVID-19 death underascertainment. We therefore explored a range of different scenarios in which we varied underascertainment (10, 33, 50, and 66%) and introduction dates (15 January 2021, 31 January 2021, 14 February 2021, and 28 February 2021).

**Fig. 4.**
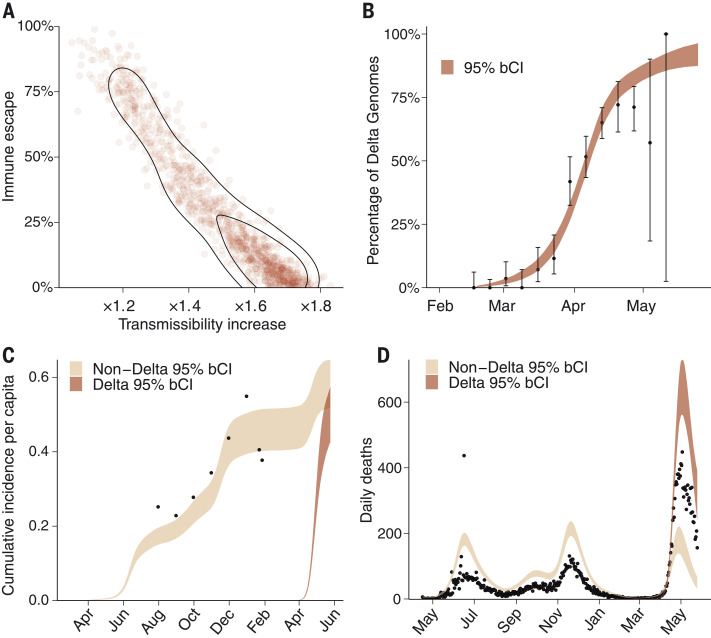
Estimates of the epidemiological characteristics of the Delta variant. Values were inferred from a two-category Bayesian transmission model fitted to mortality, serosurvey, and genomic data from Delhi, India. (**A**) Joint posterior distribution, with isoclines corresponding to the 90% and 50% enclosures of posterior density of the Delta variant immune escape and transmissibility increase relative to non-Delta categories. Immune escape has a median of 20% with 50% Bayesian credible interval (bCI) of 10 to 50%, and transmissibility has a median increase of 1.5 with 50% bCI of 1.3 to 1.7. (**B**) Delta fraction over time, inferred by the model. Black dots represent genome sampling data points, with exact binomial confidence intervals. (**C**) Serosurvey data (black dots) and inferred cumulative incidence for Delta and non-Delta variant categories. (**D**) Mortality data (black dots) and inferred deaths assuming 50% underreporting. Other underascertainment scenarios are presented in the supplementary materials.

Using this framework for an introduction date of 14 February 2021 and mortality underascertainment of 50%, our results, shown in Fig. 4, indicate that the Delta variant is 1.3- to 1.7-fold [50% Bayesian credible interval (bCI)] more transmissible than earlier and co-circulating SARS-CoV-2 lineages in Delhi, including the Alpha variant. Notably, the model also indicates that the Delta variant can partially evade immunity elicited by prior infection, with prior infection providing only 50 to 90% (50% bCI) of the protection against infection with the Delta variant that it provides against previous lineages. There is an inherent trade-off between transmissibility and immune escape, and the worst-case scenario of both very high transmissibility and immune escape is rejected a posteriori by the data. Figure 4A also highlights the nature of uncertainty in the exact level of immune escape and transmissibility increase, because these inferred characteristics of the Delta variant are collinear given the modeling framework used and data currently available. The main limitations of the model are due to biases in the data and our choice of priors. For example, there is an unknown degree of underreporting, and serological estimates are likely to be systematically biased. Similarly, there are uncertainties in estimates of the temporal waning of immunity, the date of the first introduction of Delta, and the true infection fatality rate of SARS-CoV-2 variants in Delhi. We explicitly consider these limitations and partially mitigate such biases, as described in the supplementary materials. Overall, the main findings are robust to variation in prior assumptions, including both changing underascertainment and the date of introduction (supplementary methods and tables S2 and S3). The results are valid for a population where most of the immunity arose from prior infection (rather than vaccination), which is true for Delhi. On the basis of median estimates of the model (Fig. 4A) and high transmissibility of the background Alpha lineage (*18*), Delta should potentially be at least twice as transmissible as the wild-type lineage.

We note that after the massive Delta outbreak, new cases in Delhi and other North Indian states have stayed extremely low, with TPR in Delhi below 0.1% as of September 2021. This fits the serological picture presented in Fig. 2, with a very high fraction of the population being recently infected and with good immunity to Delta. However, Delta outbreaks have continued in parts of India and elsewhere in the world, despite moderately high seropositivity or vaccination levels that were previously considered to be adequate (*7*, *19*, *20*). We conclude that the Delta variant is capable of initiating fast-rising outbreaks in populations with immune responses to prior variants, resulting in reinfections and vaccination breakthroughs. Public health strategies may need to be revised to account for variants with heightened transmissibility and immune escape.

## Supplementary Material

20211014-1

## Supplementary Materials

### This PDF file includes:

Materials and Methods Supplementary Text Figs. S1 to S5 Tables S1 to S3 List of INSACOG Consortium Members References (*23*–*42*)

### Other Supplementary Material for this manuscript includes the following:

MDAR Reproducibility Checklist

## Notes

View/request a protocol for this paper from *Bio-protocol*.
