# Supplementary material for: Genomic characterization and epidemiology of an emerging SARS-CoV-2 variant in Delhi, India
Source: Science. 2021 Oct 14;374(6570):995–9. doi: 10.1126/science.abj9932 (PMC7612010; doi:10.1126/science.abj9932)
Supplement: 20211014-1 [file science.abj9932.v1.pdf]

Cite as: M. S. Dhar *et al.*, *Science*
10.1126/science.abj9932 (2021).

Genomic characterization and epidemiology of an emerging SARS-CoV-2 variant in Delhi, India

Mahesh S. Dhar[1]†, Robin Marwal[1]†, Radhakrishnan VS[1]†, Kalaiarasan Ponnusamy[1]†, Bani Jolly[2,3]†, Rahul C. Bhoyar[2]†, Viren Sardana[2,3], Salwa Naushin[2,3], Mercy Rophina[2,3], Thomas A. Mellan[4], Swapnil Mishra[4], Charles Whittaker[4], Saman Fatihi[2,3], Meena Datta[1], Priyanka Singh[1], Uma Sharma[1], Rajat Ujjainiya[2,3], Nitin Bhatheja[2], Mohit Kumar Divakar[2,3], Manoj K. Singh[1], Mohamed Imran[2,3], Vigneshwar Senthivel[2,3], Ranjeet Maurya[2,3], Neha Jha[2], Priyanka Mehta[2], Vivekanand A[2,3], Pooja Sharma[2,3], Arvinden VR[2,3], Urmila Chaudhary[1], Namita Soni[1], Lipi Thukral[2,3], Seth Flaxman[5], Samir Bhatt[4,6], Rajesh Pandey[2,3], Debasis Dash[2,3], Mohammed Faruq[2,3], Hemlata Lall[1], Hema Gogia[1], Preeti Madan[1], Sanket Kulkarni[1], Himanshu Chauhan[1], Shantanu Sengupta[2,3], Sandhya Kabra[1], The Indian SARS-CoV-2 Genomics Consortium (INSACOG)‡, Ravindra K. Gupta[7,8], Sujeet K. Singh[1], Anurag Agrawal[2,3]*, Partha Rakshit[1]*

[1]National Centre for Disease Control, Delhi, India. [2]CSIR-Institute of Genomics and Integrative Biology, New Delhi, India. [3]Academy for Scientific and Innovative Research, Ghaziabad, India. [4]Medical Research Council (MRC) Centre for Global Infectious Disease Analysis, Jameel Institute, School of Public Health, Imperial College London, London, UK. [5]Department of Mathematics, Imperial College London, London, UK. [6]Section of Epidemiology, Department of Public Health, University of Copenhagen, Copenhagen, Denmark. [7]Department of Medicine, Cambridge Institute of Therapeutic Immunology and Infectious Disease (CITIID), University of Cambridge, Cambridge, UK. [8]Africa Health Research Institute, KwaZulu-Natal, South Africa.

†These authors contributed equally to this work.

‡The Indian SARS-CoV-2 Genomics Consortium (INSACOG) collaborators and affiliations are listed in the supplementary materials.

***Corresponding author. Email: partho_rakshit@yahoo.com (P.R.); a.agrawal@igib.in (A.A.)**

Delhi, the national capital of India, has experienced multiple SARS-CoV-2 outbreaks in 2020 and reached population seropositivity of over 50% by 2021. During April 2021, the city became overwhelmed by COVID-19 cases and fatalities, as a new variant B.1.617.2 (Delta) replaced B.1.1.7 (Alpha). A Bayesian model explains the growth advantage of Delta through a combination of increased transmissibility and reduced sensitivity to immune responses generated against earlier variants (median estimates; ×1.5-fold, 20% reduction). Seropositivity of an employee and family cohort increased from 42% to 87.5% between March and July 2021, with 27% reinfections, as judged by increased antibody concentration after a previous decline. The likely high transmissibility and partial evasion of immunity by the Delta variant contributed to an overwhelming surge in Delhi.

After escaping relatively unscathed during the first wave of the COVID-19 pandemic, India witnessed a ferocious second COVID-19 wave, starting in March 2021 and accounting for about half of global cases by the first week of May. SARS-CoV-2 had spread widely throughout India in the first wave, with the third national serosurvey in January 2021 finding that 21.4% of adults and 25.3% of 10- to 17-year-old adolescents were seropositive (*1*). Delhi, the national capital, was not included in the national serosurvey but had undergone multiple periods of high transmission in 2020 (Fig. 1A). In a district-wise stratified serosurvey conducted by the Delhi Government in January 2021, overall seropositivity was reported to be 56.1% (95% CI, 55.5–56.8%), ranging from 49.1% to 62.2% across 11 districts (*2*). This was expected to confer some protection from future outbreaks.

Despite high seropositivity, Delhi was amongst the most affected cities during the second wave. The rise in new cases was exceptionally rapid in April, going from approximately 2000 to 20,000 between 31 March and 16 April. This was accompanied by a rapid rise in hospitalizations and ICU admissions (Fig. 1B). In this emergency situation with saturated bed occupancy by 12 April, major private hospitals were declared by the state as full COVID care-only and senior medical students, including from alternative medicine branches, were pressed into service (*3*). Deaths rose proportionately (Fig. 1C) and the case-fatality ratio (CFR), estimated as the scaling factor between time-advanced cases and deaths (Fig. 1D), was stable (mean, SD; 1.9, 0.3%). Population spread of SARS-CoV-2 is underestimated by test positive cases alone (*1*, *2*). To better understand the degree of spread and the factors leading to the unexpectedly severe outbreak, we used all available data including testing, sequencing, serosurveys, and serially followed cohorts.

In the absence of finely resolved or serial data from national and state surveys, we focused on data for Delhi participants of a national serosurvey of Council of Scientific and

Industrial Research (CSIR, India) employees and their family members (Fig. 2A and table S1). Samples were initially collected from the end of July to mid-September 2020 (Phase I). Subsequently second and third surveys were held in January/February (Phase II) and end of May to early July 2021 (Phase III), bracketing the time period of interest. The cohort details and serosurvey methodology have been previously published (*4*).

Infection was determined by anti-nucleocapsid assay, which is not affected by immunization with spike protein based vaccines. The presence of neutralizing antibodies (NAb) to wild-type SARS-CoV-2 spike protein was estimated by a surrogate viral neutralization test (sVNT, Genscript). Previous results from the full cohort have been comparable to government serosurveys, but Delhi cohort values have been slightly lower. This may be due to an overrepresentation of members with the ability to reduce exposure and avoid public transport (Fig. 2A). Within these limitations, the Delhi cohort showed a rise in seropositivity from 14.7% in Phase I (95% CI, 12.6–17.0%) to 42.1% in Phase II (95% CI, 39.0–45.2%). About one-third of NAb^{+ve} subjects at Phase I became NAb^{-ve} by Phase II, with most showing declining inhibition on sVNT assays (Fig. 2B) (*5*). Phase III seropositivity increased to 87.5% (95% CI, 85.0–89.7%) amongst unvaccinated subjects. New infections between March and June 2021 are thus likely to have vastly exceeded known cases. Amongst 91 previously infected subjects with serial measurements at three time points including Phase III (T3), 25 (27.5%, 95% CI 18.4–37.5%) had a pattern of declining antibody concentration between T1 and T2, followed by a sharp rise at T3, indicative of reinfection (Fig. 2C). Confirmation of reinfection by either RT-PCR (n = 8) or symptomatic illness (n = 2) was available for 10 of the 25 subjects. No severe illness or hospitalization was reported in reinfections.

Time periods of increased transmission were associated with declining RT-PCR cycle threshold (Ct) values (Fig. 3A and fig. S1), attributable to a higher proportion of recently infected individuals with high viral loads (*6*). However, the Ct decline was far greater in April 2021 (dCt, SE; −4.06, 0.27, $P < 0.001$) than seen previously (dCt Nov 2020 ~ −1.5). Comparing April 2021 with November 2020, high viral load samples (Ct < 20) doubled in clinical samples ($P < 0.001$), and nearly doubled in campus surveillance testing data, where most positives were from recently infected individuals [15% (n = 297) vs. 9% (n = 358), P = 0.02].

Genome analysis trends, in representative samples drawn from the general population over the same period, showed seeding and expansion of B.1.1.7 (Alpha), B.1.617.1 (Kappa) and B.1.617.2 (Delta) lineages in 2021, with Delta becoming the dominant lineage in Delhi during April (Fig. 3B). The proportion of Delta variant was strongly correlated to the rise in cases and healthcare stress (fig. S2). Overall, the genomic and epidemiological data were most consistent with the hypothesis that a new variant with higher infectivity, Delta, was driving the unexpected overwhelming surge in Delhi. Recent in vitro data supports the possibility of a higher replication rate for Delta, thereby explaining potentially higher viral loads in RT-PCR data and greater transmissibility (*7*).

We further investigated the sequence of seeding and spread of Alpha, Kappa, and Delta variants of SARS-CoV-2. Phylogenetic analysis showed common origins between Alpha variants in Delhi and Punjab, and between Kappa or Delta variants in Delhi and Maharashtra, where Kappa and Delta were first sequenced (Fig. 3C and fig. S3). There was a recurring pattern of initial smaller outbreaks with Alpha variant, followed by larger outbreaks coinciding with Alpha to Delta transition across all neighboring states (fig. S4). The substantial relative growth advantage of Delta was explored in terms of transmissibility and/or immune escape. The rise of Delta, but not other lineages, was temporally coincident with a rise in test positivity rate (TPR) and new cases during the surge (fig. S5). While overall vaccination levels were only about 5% in Delhi, most healthcare workers had received one or two doses of ChAdOx1-nCov19 (Astra-Zeneca / Serum Institute, India) or BBV152 (Bharat Biotech, India) (*8*, *9*). We sequenced 24 breakthrough infections starting at least one week after the first dose, collected between 22 March and 28 April at NCDC. The ratio of Delta to non-Delta lineages was 850:1211 from 20 March to 30 April. In contrast, Delta to other lineage ratio was 13:3 in sixteen breakthroughs post one dose and 7:1 in eight breakthroughs post the second dose of vaccine. While the small sample size and lack of a formal control group preclude definitive analysis, estimated higher odds for Delta in vaccination breakthroughs (OR, 7.1; 95% CI, 2.4–20.9) corroborate other reports of reduced vaccine effectiveness against Delta (*10*).

To better characterize how the properties of Delta might differ from other SARS-CoV-2 lineages previously circulating in the city, we used a Bayesian model of SARS-CoV-2 transmission and mortality that simultaneously models the dynamics of two-categories of virus ("B.1.617.2" and "non-B.1.617.2") (*11*), while also explicitly incorporating natural waning of immunity derived from prior infection, with the duration of immunity consistent with the results of recent longitudinal cohort studies (*12*, *13*). Details of the model and input data are in supplementary methods. Briefly, the model is fitted to COVID-19 mortality data, genomic sequence data presented here and from GISAID (with Pangolin lineage classification) (*14–16*) and serological data presented alongside an additional longitudinal serosurvey carried out in the city in the period July to December 2020 (*17*). Substantial uncertainties remain as to the date of introduction of B.1.617.2 into Delhi and the degree of COVID-19 death under-ascertainment. We, therefore, explored a range of different scenarios

in which we varied under-ascertainment (10%, 33%, 50%, and 66%) and introduction dates (15 January 2021, 31 January 2021, 14 February 2021, and 28 February 2021).

Using this framework for an introduction date of 14 February 2021 and mortality under-ascertainment of 50%, our results shown in Fig. 4 indicate that the Delta variant is ×1.3–1.7 (50% bCI) fold more transmissible than earlier/co-circulating SARS-CoV-2 lineages in Delhi, including the Alpha variant. Importantly, the model also indicates that the Delta variant can partially evade immunity elicited by prior infection, with prior infection providing only 50 to 90% (50% bCI) of the protection against infection with Delta variant that it provides against previous lineages. We note there is an inherent trade-off between transmissibility and immune escape, and that the worst-case scenario of both very high transmissibility and immune escape is rejected a posteriori by the data. Figure 4A also highlights the nature of uncertainty in the exact level of immune escape and transmissibility increase, since these inferred characteristics of the Delta variant are collinear given the modelling framework used and data currently available. The main limitations of the model are due to biases in the data and our choices of priors. For example, there is an unknown degree of underreporting and serological estimates are likely to be systematically biased. Similarly, there are uncertainties in estimates of the temporal waning of immunity, the date of the first introduction of Delta, and the true infection fatality rate of SARS-CoV-2 variants in Delhi. We explicitly consider these limitations and partially mitigate such biases, as described in the supplementary materials. Overall, the main findings are robust to variation in prior assumptions, including both changing under-ascertainment and the date of introduction (supplementary methods; tables S2 and S3). The results are valid for a population where the majority of immunity arose from prior infection (rather than vaccination), which is true for Delhi. Based on median estimates of the model (Fig. 4A) and high transmissibility of the background Alpha lineage (*18*), Delta should potentially be at least twice as transmissible as the wildtype lineage.

We note that after the massive Delta outbreak, new cases in Delhi and other North Indian states have stayed extremely low, with TPR in Delhi below 0.1% as of September 2021. This fits the serological picture presented in Fig. 2, with a very high fraction of the population being recently infected and with good immunity to Delta. However, Delta outbreaks have continued in parts of India and elsewhere in the world, despite moderately high seropositivity or vaccination levels that were previously considered to be adequate (*7*, *19*, *20*). We conclude that the Delta variant is capable of initiating fast-rising outbreaks in populations with immune responses to prior variants, resulting in reinfections and vaccination breakthroughs. Public health strategies may need to be revised to account for variants with heightened transmissibility and immune escape.

REFERENCES AND NOTES

1. M. V. Murhekar, T. Bhatnagar, J. W. V. Thangaraj, V. Saravanakumar, M. S. Kumar, S. Selvaraju, K. Rade, C. P. G. Kumar, R. Sabarinathan, A. Turuk, S. Asthana, R. Balachandar, S. D. Bangar, A. K. Bansal, V. Chopra, D. Das, A. K. Deb, K. R. Devi, V. Dhikav, G. R. Dwivedi, S. M. S. Khan, M. S. Kumar, A. Laxmaiah, M. Madhukar, A. Mahapatra, C. Rangaraju, J. Turuk, R. Yadav, R. Andhalkar, K. Arunraj, D. K. Bharadwaj, P. Bharti, D. Bhattacharya, J. Bhat, A. S. Chahal, D. Chakraborty, A. Chaudhury, H. Deval, S. Dhatrak, R. Dayal, D. Elantamilan, P. Giridharan, I. Haq, R. K. Hudda, B. Jagjeevan, A. Kalliath, S. Kanungo, N. N. Krishnan, J. S. Kshatri, A. Kumar, N. Kumar, V. G. V. Kumar, G. G. J. N. Lakshmi, G. Mehta, N. K. Mishra, A. Mitra, K. Nagbhushanam, A. Nimmathota, A. R. Nirmala, A. K. Pandey, G. V. Prasad, M. A. Qurieshi, S. D. Reddy, A. Robinson, S. Sahay, R. Saxena, K. Sekar, V. K. Shukla, H. B. Singh, P. K. Singh, P. Singh, R. Singh, N. Srinivasan, D. S. Varma, A. Viramgami, V. C. Wilson, S. Yadav, S. Yadav, K. Zaman, A. Chakrabarti, A. Das, R. S. Dhaliwal, S. Dutta, R. Kant, A. M. Khan, K. Narain, S. Narasimhaiah, C. Padmapriyadarshini, K. Pandey, S. Pati, S. Patil, H. Rajkumar, T. Ramarao, Y. K. Sharma, S. Singh, S. Panda, D. C. S. Reddy, B. Bhargava, T. Anand, G. R. Babu, H. Chauhan, T. Dikid, R. R. Gangakhedkar, S. Kant, S. Kulkarni, J. P. Muliyil, R. M. Pandey, S. Sarkar, N. Shah, A. Shrivastava, S. K. Singh, S. Zodpe, A. Hindupur, P. R. Asish, M. Chellakumar, D. Chokkalingam, S. Dasgupta, M. M. E. Gowtham, A. Jose, K. Kalaiyarasi, N. N. Karthik, T. Karunakaran, G. Kiruthika, H. Dinesh Kumar, S. Sarath Kumar, M. P. Sarath Kumar, E. Michaelraj, J. Pradhan, E. B. Arun Prasath, D. Gladys Angelin Rachel, S. Rani, A. Rozario, R. Sivakumar, P. Gnana Soundari, K. Sujeetha, A. Vinod; ICMR Serosurveillance Group, SARS-CoV-2 seroprevalence among the general population and healthcare workers in India, December 2020-January 2021. *Int. J. Infect. Dis.* **108**, 145–155 (2021). doi:10.1016/j.ijid.2021.05.040 Medline
2. S. Jain (@SatyendarJain), Twitter post, 2 February 2021, 5:19 a.m.; https://twitter.com/satyendarjain/status/1356547817427226624?lang=en.
3. Government of National Capital Territory of Delhi, Orders F.23/Misc/COVID-19/DGHS/NHC/2020/Pt XVIII/90-97 and No.01/H&FW/COVID-Cell/2020/07/ss4hfw/726, 12 April 2021; http://health.delhigovt.nic.in/wps/wcm/connect/doit_health/Health/Home/Covid19/Covid+19+Related+order+April+2021.
4. S. Naushin, V. Sardana, R. Ujjainiya, N. Bhatheja, R. Kutum, A. K. Bhaskar, S. Pradhan, S. Prakash, R. Khan, B. S. Rawat, K. B. Tallapaka, M. Anumalla, G. R. Chandak, A. Lahiri, S. Kar, S. R. Mulay, M. N. Mugale, M. Srivastava, S. Khan, A. Srivastava, B. Tomar, M. Veerapandian, G. Venkatachalam, S. R. Vijayakumar, A. Agarwal, D. Gupta, P. M. Halami, M. S. Peddha, G. M. Sundaram, R. P. Veeranna, A. Pal, V. K. Agarwal, A. K. Maurya, R. K. Singh, A. K. Raman, S. K. Anandasadagopan, P. Karuppanan, S. Venkatesan, H. K. Sardana, A. Kothari, R. Jain, A. Thakur, D. S. Parihar, A. Saifi, J. Kaur, V. Kumar, A. Mishra, I. Gogeri, G. Rayasam, P. Singh, R. Chakraborty, G. Chaturvedi, P. Karunakar, R. Yadav, S. Singhmar, D. Singh, S. Sarkar, P. Bhattacharya, S. Acharya, V. Singh, S. Verma, D. Soni, S. Seth, S. Vashisht, S. Thakran, F. Fatima, A. P. Singh, A. Sharma, B. Sharma, M. Subramanian, Y. S. Padwad, V. Hallan, V. Patial, D. Singh, N. V. Tripude, P. Chakrabarti, S. K. Maity, D. Ganguly, J. Sarkar, S. Ramakrishna, B. N. Kumar, K. A. Kumar, S. G. Gandhi, P. S. Jamwal, R. Chouhan, V. L. Jamwal, N. Kapoor, D. Ghosh, G. Thakkar, U. Subudhi, P. Sen, S. R. Chaudhury, R. Kumar, P. Gupta, A. Tuli, D. Sharma, R. P. Ringe, A. D, M. Kulkarni, D. Shanmugam, M. S. Dharne, S. G. Dastager, R. Joshi, A. P. Patil, S. N. Mahajan, A. H. Khan, V. Wagh, R. K. Yadav, A. Khilari, M. Bhadange, A. H. Chaurasiya, S. E. Kulsange, K. Khairnar, S. Paranjape, J. Kalita, N. G. Sastry, T. Phukan, P. Manna, W. Romi, P. Bharali, D. Ozah, R. K. Sahu, E. V. Babu, R. Sukumaran, A. R. Nair, P. K. Valappil, A. Puthiyamadam, A. Velayudhanpillai, K. Chodankar, S. Damare, Y. Madhavi, V. V. Aggarwal, S. Dahiya, A. Agrawal, D. Dash, S. Sengupta, Insights from a Pan India Sero-Epidemiological survey (Phenome-India Cohort) for SARS-CoV2. *eLife* **10**, e66537 (2021). doi:10.7554/eLife.66537 Medline
5. J. M. Dan, J. Mateus, Y. Kato, K. M. Hastie, E. D. Yu, C. E. Faliti, A. Grifoni, S. I. Ramirez, S. Haupt, A. Frazier, C. Nakao, V. Rayaprolu, S. A. Rawlings, B. Peters, F. Krammer, V. Simon, E. O. Saphire, D. M. Smith, D. Weiskopf, A. Sette, S. Crotty, Immunological memory to SARS-CoV-2 assessed for up to 8 months after

infection. *Science* 371, eabf4063 (2021). doi:10.1126/science.abf4063 Medline
6. J. A. Hay, L. Kennedy-Shaffer, S. Kanjilal, N. J. Lennon, S. B. Gabriel, M. Lipsitch, M. J. Mina, Estimating epidemiologic dynamics from cross-sectional viral load distributions. *Science* 373, eabh0635 (2021). doi:10.1126/science.abh0635 Medline
7. P. Mlcochova, S. Kemp, M. S. Dhar, G. Papa, B. Meng, I. A. T. M. Ferreira, R. Datir, D. A. Collier, A. Albecka, S. Singh, R. Pandey, J. Brown, J. Zhou, N. Goonawardane, S. Mishra, C. Whittaker, T. Mellan, R. Marwal, M. Datta, S. Sengupta, K. Ponnusamy, V. S. Radhakrishnan, A. Abdullahi, O. Charles, P. Chattopadhyay, P. Devi, D. Caputo, T. Peacock, D. C. Wattal, N. Goel, A. Satwik, R. Vaishya, M. Agarwal, A. Mavousian, J. H. Lee, J. Bassi, C. Silacci-Fegni, C. Saliba, D. Pinto, T. Irie, I. Yoshida, W. L. Hamilton, K. Sato, S. Bhatt, S. Flaxman, L. C. James, D. Corti, L. Piccoli, W. S. Barclay, P. Rakshit, A. Agrawal, R. K. Gupta; Indian SARS-CoV-2 Genomics Consortium (INSACOG); Genotype to Phenotype Japan (G2P-Japan) Consortium; CITIID-NIHR BioResource COVID-19 Collaboration, SARS-CoV-2 B.1.617.2 Delta variant replication and immune evasion. *Nature* 10.1038/s41586-021-03944-y (2021). doi:10.1038/s41586-021-03944-y Medline
8. P. M. Folegatti, K. J. Ewer, P. K. Aley, B. Angus, S. Becker, S. Belij-Rammerstorfer, D. Bellamy, S. Bibi, M. Bittaye, E. A. Clutterbuck, C. Dold, S. N. Faust, A. Finn, A. L. Flaxman, B. Hallis, P. Heath, D. Jenkin, R. Lazarus, R. Makinson, A. M. Minassian, K. M. Pollock, M. Ramasamy, H. Robinson, M. Snape, R. Tarrant, M. Voysey, C. Green, A. D. Douglas, A. V. S. Hill, T. Lambe, S. C. Gilbert, A. J. Pollard,; Oxford COVID Vaccine Trial Group, Safety and immunogenicity of the ChAdOx1 nCoV-19 vaccine against SARS-CoV-2: A preliminary report of a phase 1/2, single-blind, randomised controlled trial. *Lancet* 396, 467–478 (2020). doi:10.1016/S0140-6736(20)31604-4 Medline
9. R. Ella, S. Reddy, W. Blackwelder, V. Potdar, P. Yadav, V. Sarangi, V. Kumar Aileni, S. Kanungo, S. Rai, P. Reddy, S. Verma, C. Singh, S. Redkar, S. Mohapatra, A. Pandey, P. Ranganadin, R. Gumashta, M. Multani, S. Mohammad, P. Bhatt, L. Kumari, G. Sapkal, N. Gupta, P. Abraham, S. Panda, S. Prasad, B. Bhargava, K. Ella, K. M. Vadrevu; COVAXIN Study Group, Efficacy, safety, and lot to lot immunogenicity of an inactivated SARS-CoV-2 vaccine (BBV152): a, double-blind, randomised, controlled phase 3 trial. medRxiv 2021.06.30.21259439 [Preprint] (2021). https://doi.org/10.1101/2021.06.30.21259439.
10. J. Lopez Bernal, N. Andrews, C. Gower, E. Gallagher, R. Simmons, S. Thelwall, J. Stowe, E. Tessier, N. Groves, G. Dabrera, R. Myers, C. N. J. Campbell, G. Amirthalingam, M. Edmunds, M. Zambon, K. E. Brown, S. Hopkins, M. Chand, M. Ramsay, Effectiveness of Covid-19 vaccines against the B.1.617.2 (delta) variant. *N. Engl. J. Med.* 385, 585–594 (2021). doi:10.1056/NEJMoa2108891 Medline
11. S. Flaxman, S. Mishra, A. Gandy, H. J. T. Unwin, T. A. Mellan, H. Coupland, C. Whittaker, H. Zhu, T. Berah, J. W. Eaton, M. Monod, A. C. Ghani, C. A. Donnelly, S. Riley, M. A. C. Vollmer, N. M. Ferguson, L. C. Okell, S. Bhatt; Imperial College COVID-19 Response Team, Estimating the effects of non-pharmaceutical interventions on COVID-19 in Europe. *Nature* 584, 257–261 (2020). doi:10.1038/s41586-020-2405-7 Medline
12. C. H. Hansen, D. Michlmayr, S. M. Gubbels, K. Mølbak, S. Ethelberg, Assessment of protection against reinfection with SARS-CoV-2 among 4 million PCR-tested individuals in Denmark in 2020: A population-level observational study. *Lancet* 397, 1204–1212 (2021). doi:10.1016/S0140-6736(21)00575-4 Medline
13. V. J. Hall, S. Foulkes, A. Charlett, A. Atti, E. J. M. Monk, R. Simmons, E. Wellington, M. J. Cole, A. Saei, B. Oguti, K. Munro, S. Wallace, P. D. Kirwan, M. Shrotri, A. Vusirikala, S. Rokadiya, M. Kall, M. Zambon, M. Ramsay, T. Brooks, C. S. Brown, M. A. Chand, S. Hopkins; SIREN Study Group, SARS-CoV-2 infection rates of antibody-positive compared with antibody-negative health-care workers in England: A large, multicentre, prospective cohort study (SIREN). *Lancet* 397, 1459–1469 (2021). doi:10.1016/S0140-6736(21)00675-9 Medline
14. Y. Shu, J. McCauley, GISAID: Global initiative on sharing all influenza data - from vision to reality. *Euro Surveill.* 22, 30494 (2017). doi:10.2807/1560-7917.ES.2017.22.13.30494 Medline
15. A. Rambaut, E. C. Holmes, Á. O'Toole, V. Hill, J. T. McCrone, C. Ruis, L. du Plessis, O. G. Pybus, A dynamic nomenclature proposal for SARS-CoV-2 lineages to assist genomic epidemiology. *Nat. Microbiol.* 5, 1403–1407 (2020). doi:10.1038/s41564-020-0770-5 Medline
16. Á. O'Toole, E. Scher, A. Underwood, B. Jackson, V. Hill, J. T. McCrone, R. Colquhoun, C. Ruis, K. Abu-Dahab, B. Taylor, C. Yeats, L. du Plessis, D. Maloney, N. Medd, S. W. Attwood, D. M. Aanensen, E. C. Holmes, O. G. Pybus, A. Rambaut, Assignment of epidemiological lineages in an emerging pandemic using the pangolin tool. *Virus Evol.* 7, veab064 (2021). doi:10.1093/ve/veab064 Medline
17. A. Velumani, C. Nikam, W. Suraweera, S. H. Fu, H. Gelband, P. Brown, I. Bogoch, N. Nagelkerke, P. Jha, SARS-CoV-2 seroprevalence in 12 cities of India from July-December 2020. medRxiv 2021.03.19.21253429 [Preprint] (2021). https://doi.org/10.1101/2021.03.19.21253429.
18. N. G. Davies, S. Abbott, R. C. Barnard, C. I. Jarvis, A. J. Kucharski, J. D. Munday, C. A. B. Pearson, T. W. Russell, D. C. Tully, A. D. Washburne, T. Wenseleers, A. Gimma, W. Waites, K. L. M. Wong, K. van Zandvoort, J. D. Silverman, K. Diaz-Ordaz, R. Keogh, R. M. Eggo, S. Funk, M. Jit, K. E. Atkins, W. J. Edmunds; CMMID COVID-19 Working Group; COVID-19 Genomics UK (COG-UK) Consortium, Estimated transmissibility and impact of SARS-CoV-2 lineage B.1.1.7 in England. *Science* 372, eabg3055 (2021). doi:10.1126/science.abg3055 Medline
19. D. Planas, D. Veyer, A. Baidaliuk, I. Staropoli, F. Guivel-Benhassine, M. M. Rajah, C. Planchais, F. Porrot, N. Robillard, J. Puech, M. Prot, F. Gallais, P. Gantner, A. Velay, J. Le Guen, N. Kassis-Chikhani, D. Edriss, L. Belec, A. Seve, L. Courtellemont, H. Péré, L. Hocqueloux, S. Fafi-Kremer, T. Prazuck, H. Mouquet, T. Bruel, E. Simon-Lorière, F. A. Rey, O. Schwartz, Reduced sensitivity of SARS-CoV-2 variant Delta to antibody neutralization. *Nature* 596, 276–280 (2021). doi:10.1038/s41586-021-03777-9 Medline
20. S. Mishra, S. Mindermann, M. Sharma, C. Whittaker, T. A. Mellan, T. Wilton, D. Klapsa, R. Mate, M. Fritzsche, M. Zambon, J. Ahuja, A. Howes, X. Miscouridou, G. P. Nason, O. Ratmann, E. Semenova, G. Leech, J. F. Sandkühler, C. Rogers-Smith, M. Vollmer, H. J. T. Unwin, Y. Gal, M. Chand, A. Gandy, J. Martin, E. Volz, N. M. Ferguson, S. Bhatt, J. M. Brauner, S. Flaxman; COVID-19 Genomics UK (COG-UK) Consortium, Changing composition of SARS-CoV-2 lineages and rise of Delta variant in England. *EClinicalMedicine* 39, 101064 (2021). doi:10.1016/j.eclinm.2021.101064 Medline
21. banijolly/ncov-Delhi-Epidemiology: nCoV Delhi-Epidemiology, version 1.0, Zenodo (2021): https://doi.org/10.5281/zenodo.5521073.
22. T. Mellan, M. Swapnil, C. Whittaker, S. Bhatt, S. Flaxman, ImperialCollegeLondon/Delta_Variant_Delhi: Genomic characterization and Epidemiology of an emerging SARS- CoV-2 variant in Delhi, India, version 1.0, Zenodo (2021); https://doi.org/10.5281/zenodo.5524442.
23. F. Wu, S. Zhao, B. Yu, Y. M. Chen, W. Wang, Z. G. Song, Y. Hu, Z. W. Tao, J. H. Tian, Y. Y. Pei, M. L. Yuan, Y. L. Zhang, F. H. Dai, Y. Liu, Q. M. Wang, J. J. Zheng, L. Xu, E. C. Holmes, Y. Z. Zhang, A new coronavirus associated with human respiratory disease in China. *Nature* 579, 265–269 (2020). doi:10.1038/s41586-020-2008-3 Medline
24. J. Hadfield, C. Megill, S. M. Bell, J. Huddleston, B. Potter, C. Callender, P. Sagulenko, T. Bedford, R. A. Neher, Nextstrain: Real-time tracking of pathogen evolution. *Bioinformatics* 34, 4121–4123 (2018). doi:10.1093/bioinformatics/bty407 Medline
25. P. Sagulenko, V. Puller, R. A. Neher, TreeTime: Maximum-likelihood phylodynamic analysis. *Virus Evol.* 4, vex042 (2018). doi:10.1093/ve/vex042 Medline
26. B. Q. Minh, H. A. Schmidt, O. Chernomor, D. Schrempf, M. D. Woodhams, A. von Haeseler, R. Lanfear, IQ-TREE 2: New models and efficient methods for phylogenetic inference in the genomic era. *Mol. Biol. Evol.* 37, 1530–1534 (2020). doi:10.1093/molbev/msaa015 Medline
27. G. Yu, Using ggtree to visualize data on tree-like structures. *Curr. Protoc. Bioinformatics* 69, e96 (2020). doi:10.1002/cpbi.96 Medline
28. N. R. Faria, T. A. Mellan, C. Whittaker, I. M. Claro, D. D. S. Candido, S. Mishra, M. A. E. Crispim, F. C. S. Sales, I. Hawryluk, J. T. McCrone, R. J. G. Hulswit, L. A. M. Franco, M. S. Ramundo, J. G. de Jesus, P. S. Andrade, T. M. Coletti, G. M. Ferreira, C. A. M. Silva, E. R. Manuli, R. H. M. Pereira, P. S. Peixoto, M. U. G. Kraemer, N. Gaburo Jr., C. D. C. Camilo, H. Hoeltgebaum, W. M. Souza, E. C. Rocha, L. M. de Souza, M. C. de Pinho, L. J. T. Araujo, F. S. V. Malta, A. B. de Lima, J. D. P. Silva, D. A. G. Zauli, A. C. S. Ferreira, R. P. Schnekenberg, D. J. Laydon, P. G. T. Walker, H. M. Schlüter, A. L. P. Dos Santos, M. S. Vidal, V. S. Del Caro, R. M. F. Filho, H. M. Dos Santos, R. S. Aguiar, J. L. Proença-Modena, B. Nelson, J. A. Hay, M. Monod, X. Miscouridou, H. Coupland, R. Sonabend, M. Vollmer, A. Gandy, C. A. Prete Jr., V. H. Nascimento, M. A. Suchard, T. A. Bowden, S. L. K. Pond, C. H. Wu, O. Ratmann, N. M. Ferguson, C. Dye, N. J. Loman, P. Lemey, A. Rambaut, N. A. Fraiji, M. D. P. S. S. Carvalho, O. G. Pybus, S. Flaxman, S. Bhatt, E. C. Sabino, Genomics and

epidemiology of the P.1 SARS-CoV-2 lineage in Manaus, Brazil. *Science* **372**, 815–821 (2021). doi:10.1126/science.abh2644 Medline

29. H. J. T. Unwin, S. Mishra, V. C. Bradley, A. Gandy, T. A. Mellan, H. Coupland, J. Ish-Horowicz, M. A. C. Vollmer, C. Whittaker, S. L. Filippi, X. Xi, M. Monod, O. Ratmann, M. Hutchinson, F. Valka, H. Zhu, I. Hawryluk, P. Milton, K. E. C. Ainslie, M. Baguelin, A. Boonyasiri, N. F. Brazeau, L. Cattarino, Z. Cucunuba, G. Cuomo-Dannenburg, I. Dorigatti, O. D. Eales, J. W. Eaton, S. L. van Elsland, R. G. FitzJohn, K. A. M. Gaythorpe, W. Green, W. Hinsley, B. Jeffrey, E. Knock, D. J. Laydon, J. Lees, G. Nedjati-Gilani, P. Nouvellet, L. Okell, K. V. Parag, I. Siveroni, H. A. Thompson, P. Walker, C. E. Walters, O. J. Watson, L. K. Whittles, A. C. Ghani, N. M. Ferguson, S. Riley, C. A. Donnelly, S. Bhatt, S. Flaxman, State-level tracking of COVID-19 in the United States. *Nat. Commun.* **11**, 6189 (2020). doi:10.1038/s41467-020-19652-6 Medline
30. W. Feller, On the integral equation of renewal theory. *Ann. Math. Stat.* **12**, 243–267 (1941). doi:10.1214/aoms/1177731708
31. R. Bellman, T. Harris, On age-dependent binary branching processes. *Ann. Math.* **55**, 280 (1952). doi:10.2307/1969779
32. N. Brazeau, R. Verity, S. Jenks, H. Fu, C. Whittaker, P. Winskill, I. Dorigatti, P. Walker, S. Riley, R. P. Schnekenberg, H. Hoeltgebaum, T. A. Mellan, S. Mishra, J. T. Unwin, O. J. Watson, Z. M. Cucunubá, M. Baguelin, L. Whittles, S. Bhatt, A. C. Ghani, N. M. Ferguson, L. C. Okell, Report 34: COVID-19 infection fatality ratio: Estimates from seroprevalence (Imperial College London, 2020); https://doi.org/10.25561/83545.
33. M. Banaji, Estimating COVID-19 infection fatality rate in Mumbai during 2020. medRxiv 2021.04.08.21255101 [Preprint] (2021). https://doi.org/10.1101/2021.04.08.21255101.
34. S. Purkayastha, R. Bhattacharyya, R. Bhaduri, R. Kundu, X. Gu, M. Salvatore, D. Ray, S. Mishra, B. Mukherjee, A comparison of five epidemiological models for transmission of SARS-CoV-2 in India. *BMC Infect. Dis.* **21**, 533 (2021). doi:10.1186/s12879-021-06077-9 Medline
35. M. Pons-Salort, J. John, O. J. Watson, N. F. Brazeau, R. Verity, G. Kang, N. C. Grassly, Reconstructing the COVID-19 epidemic in Delhi, India: Infection attack rate and reporting of deaths. medRxiv 2021.03.23.21254092 [Preprint] (2021). https://doi.org/10.1101/2021.03.23.21254092.
36. M. V. Murhekar, T. Bhatnagar, S. Selvaraju, K. Rade, V. Saravanakumar, J. W. Vivian Thangaraj, M. S. Kumar, N. Shah, R. Sabarinathan, A. Turuk, P. K. Anand, S. Asthana, R. Balachandar, S. D. Bangar, A. K. Bansal, J. Bhat, D. Chakraborty, C. Rangaraju, V. Chopra, D. Das, A. K. Deb, K. R. Devi, G. R. Dwivedi, S. M. Salim Khan, I. Haq, M. S. Kumar, A. Laxmaiah, Madhuka, A. Mahapatra, A. Mitra, A. R. Nirmala, A. Pagdhune, M. A. Qurieshi, T. Ramarao, S. Sahay, Y. K. Sharma, M. B. Shrinivasa, V. K. Shukla, P. K. Singh, A. Viramgami, V. C. Wilson, R. Yadav, C. P. Girish Kumar, H. E. Luke, U. D. Ranganathan, S. Babu, K. Sekar, P. D. Yadav, G. N. Sapkal, A. Das, P. Das, S. Dutta, R. Hemalatha, A. Kumar, K. Narain, S. Narasimhaiah, S. Panda, S. Pati, S. Patil, K. Sarkar, S. Singh, R. Kant, S. Tripathy, G. S. Toteja, G. R. Babu, S. Kant, J. P. Muliyil, R. M. Pandey, S. Sarkar, S. K. Singh, S. Zodpey, R. R. Gangakhedkar, D. C. S. Reddy, B. Bhargava, Prevalence of SARS-CoV-2 infection in India: Findings from the national serosurvey, May-June 2020. *Indian J. Med. Res.* **152**, 48–60 (2020). doi:10.4103/ijmr.IJMR_3290_20 Medline
37. J. Unnikrishnan, S. Mangalathu, R. V. Kutty, Estimating under-reporting of COVID-19 cases in Indian states: An approach using a delay-adjusted case fatality ratio. *BMJ Open* **11**, e042584 (2021). doi:10.1136/bmjopen-2020-042584 Medline
38. R. K. Biswas, A. Afiaz, S. Huq, Underreporting COVID-19: The curious case of the Indian subcontinent. *Epidemiol. Infect.* **148**, e207 (2020). doi:10.1017/S0950268820002095 Medline
39. J. Hellewell, T. W. Russell, R. Beale, G. Kelly, C. Houlihan, E. Nastouli, A. J. Kucharski; SAFER Investigators and Field Study Team; Crick COVID-19 Consortium; CMMID COVID-19 working group, Estimating the effectiveness of routine asymptomatic PCR testing at different frequencies for the detection of SARS-CoV-2 infections. *BMC Med.* **19**, 106 (2021). doi:10.1186/s12916-021-01982-x Medline
40. B. Borremans, A. Gamble, K. C. Prager, S. K. Helman, A. M. McClain, C. Cox, V. Savage, J. O. Lloyd-Smith, Quantifying antibody kinetics and RNA detection during early-phase SARS-CoV-2 infection by time since symptom onset. *eLife* **9**, e60122 (2020). doi:10.7554/eLife.60122 Medline
41. J. A. Scott, A. Gandy, S. Mishra, J. Unwin, S. Flaxman, S. Bhatt, Epidemia: Modeling of epidemics using hierarchical Bayesian models, R package version 1.0 (2020); https://imperialcollegelondon.github.io/epidemia/.
42. S. Bhatt, N. Ferguson, S. Flaxman, A. Gandy, S. Mishra, J. A. Scott, Semi-mechanistic Bayesian modeling of COVID-19 with renewal processes. arXiv:2012.00394v2 [stat.AP] (2020).

ACKNOWLEDGMENTS

Acknowledgments: Suggestions by N. K Arora, G. Padmanabhan and member of the Scientific and Clinical Advisory Group of INSACOG are acknowledged. Coordinators of CSIR labs and centers are acknowledged for CSIR-Cohort. We gratefully acknowledge the authors from the originating laboratories and the submitting laboratories, who generated and shared via GISAID genetic sequence data on which this research is based. A full list acknowledging GISAID contributors for the data used in this study can be found in data S3. **Funding:** Support from Ministry of Health and Family Welfare, Council of Scientific and Industrial Research (CSIR-Phenome India Grant, MLP-2007; InGen-CoV2, MLP-2005), and Department of Biotechnology (INSACOG), India, is acknowledged. **Author contributions:** Conceived study: A.A., P.R., S.K.S., R.K.G., S.Ka., S.S., M.F., D.D., R.P., S.B., S.Fl., R.C.B., B.J.; Designed study and experiments: A.A., P.R., S.K.S., R.K.G, S.Ka., S.S., M.F., R.P., U.S., P.Si., M.D., R.C.B., K.P., R.V.S., R.Mar., M.S.D.; Performed experiments: M.S.D., R.Mar., R.V.S., R.C.B., S.N., M.D., P.Si., U.S., R.U., M.K.D., M.K.S., M.I., V.Se., P.Sh.; Patient data collection and analysis: M.S.D., R.Mar., R.V.S., K.P., M.D., P.Si., U.S., M.K.S., U.C., N.S., H.L., H.G., P.Ma., S.Ku., H.C., P.R.; Performed bioinformatics analyses: M.S.D., R.Mar., R.V.S., K.P., B.J., S.Fa., N.B., R.Mau., N.J., P.Me., V.A., A.V.R., L.T.; Performed statistical analyses: A.A., V.Sa., T.A.M., S.M., C.W., S.Fl., S.B., D.D.; Interpreted data: A.A., P.R., M.S.D., R.Mar., R.V.S., K.P., B.J., V.Sa., S.N., M.R., T.A.M., S.M., C.W., R.U., N.B., L.T., S.Fl., S.B., R.P., D.D., M.F., S.S., S.Ka., R.K.G., S.K.S. **Competing interests:** The authors declare that they have no competing interests. **Data and materials availability:** All data used in the analysis is accessible through supplementary materials as data files or repository links (*21*) including accession numbers for all genomic data that has been deposited at GISAID. Code and data for replication of the Bayesian inference are available in the following repository (*22*). Biological samples from infectious patients and patient details were collected for the purpose of public health, and cannot be shared for reasons of confidentiality and to comply with Indian government regulations. This work is licensed under a Creative Commons Attribution 4.0 International (CC BY 4.0) license, which permits unrestricted use, distribution, and reproduction in any medium, provided the original work is properly cited. To view a copy of this license, visit https://creativecommons.org/licenses/by/4.0/. This license does not apply to figures/photos/artwork or other content included in the article that is credited to a third party; obtain authorization from the rights holder before using such material.

SUPPLEMENTARY MATERIALS

science.org/doi/10.1126/science.abj9932
Materials and Methods
Supplementary Text
Figs. S1 to S5
Tables S1 to S3
List of INSACOG Consortium Members
References (*23*–*42*)
MDAR Reproducibility Checklist
Data S1 to S9

15 June 2021; accepted 6 October 2021
Published online 14 October 2021
10.1126/science.abj9932

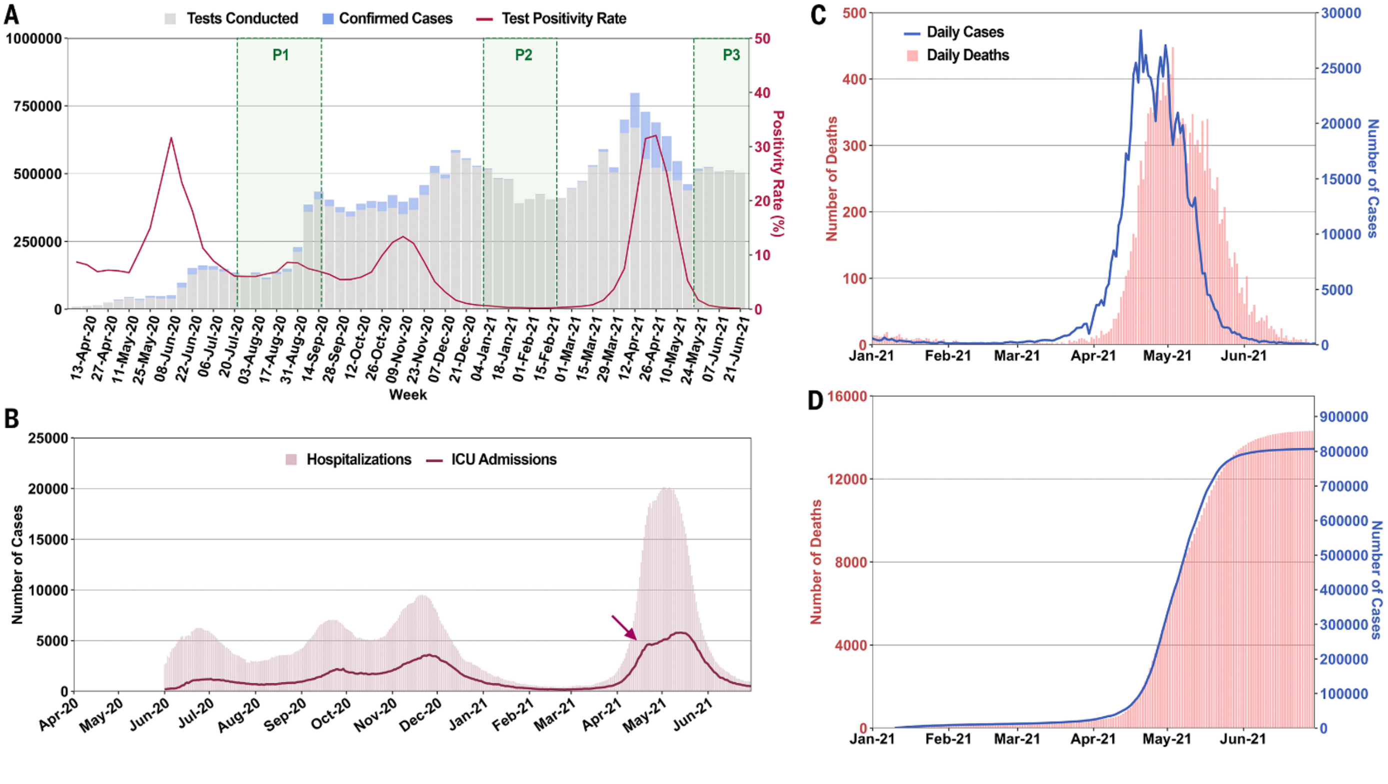

Fig. 1. Multiple surges of SARS-CoV2 infections in Delhi with an overwhelming outbreak in April–May 2021. (**A**) Weekly tests, new cases and test positivity rates (TPR) in Delhi from April 2020 to June 2021. Sample collection period for CSIR serosurveys is marked as P1-P3. (**B**) Number of hospitalized and ICU patients plotted on a daily basis from June 2020 to 2021. Arrowhead marks possible saturation of ICU capacity (*3*). (**C**) Daily cases and deaths from January to June 2021. (**D**) Time advanced and scaled cumulative cases, fitted to cumulative deaths. Time advancement of cumulative reported cases by 8 days was done for maximal coincidence with scaled cumulative deaths. CFR = averaged scaling factor [cumulative deaths/time advanced cumulative cases]; (Mean ± SD; 0.019 ± 0.003).

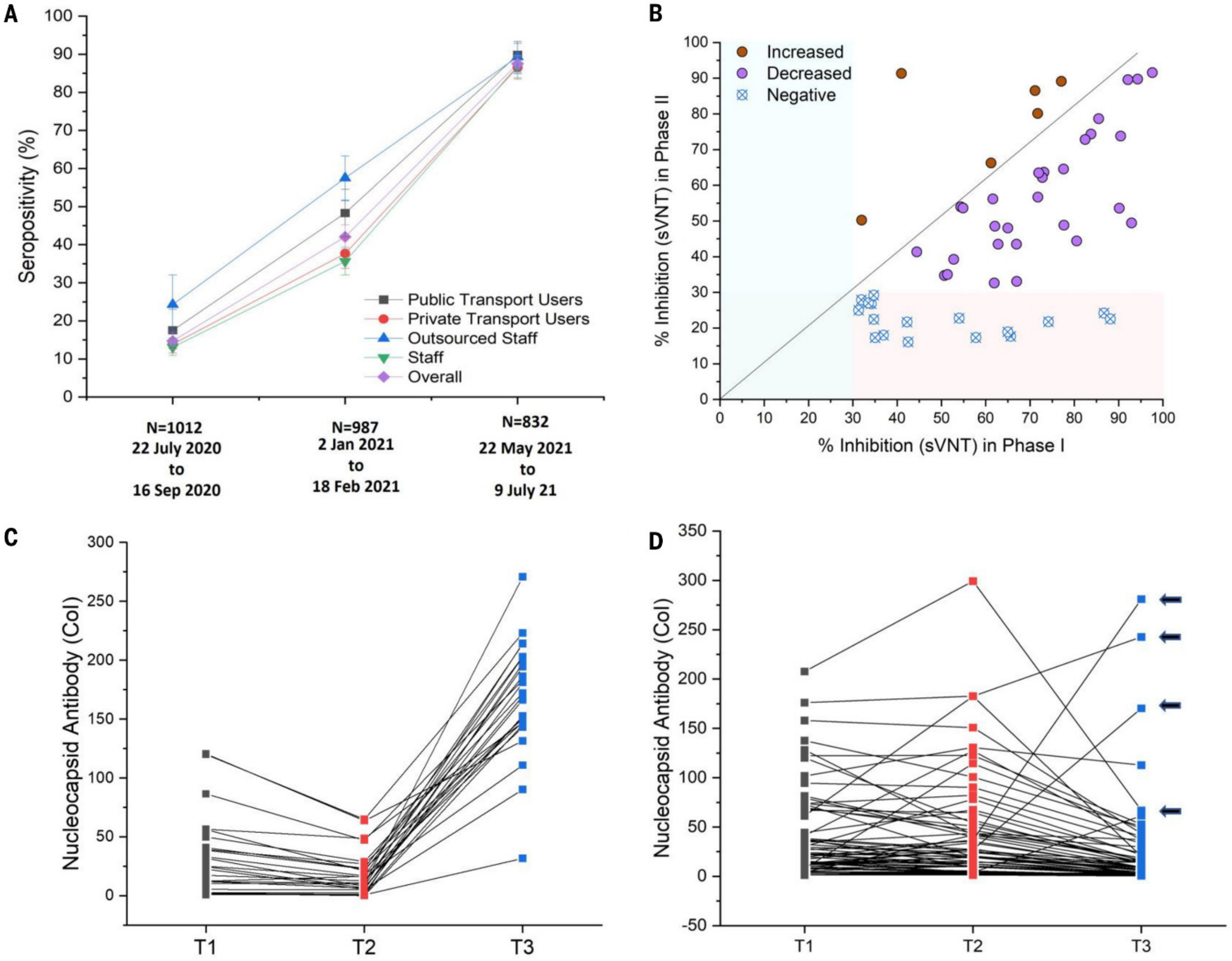

Fig. 2. Serological estimates of prior infections, preexisting immunity and new infections for the April–May outbreak. (**A**) Seropositivity in CSIR cohort, subdivided by nature of employment and use of public transport, is plotted for different time-periods (Phase I to Phase III, proportion ± 95% CI). Details are in table S1. (**B**) Variability and temporal decline in neutralization capacity estimated by sVNT assay between Phase I and II (*n* = 52). (**C**) Serial antibody concentration measurements in initially seropositive subjects (*n* = 91). Pattern suggestive of reinfections is shown (decline followed by rise, *n* = 25). (**D**) shows remaining data (*n* = 66), with four indeterminate reinfection cases indicated by arrowheads.

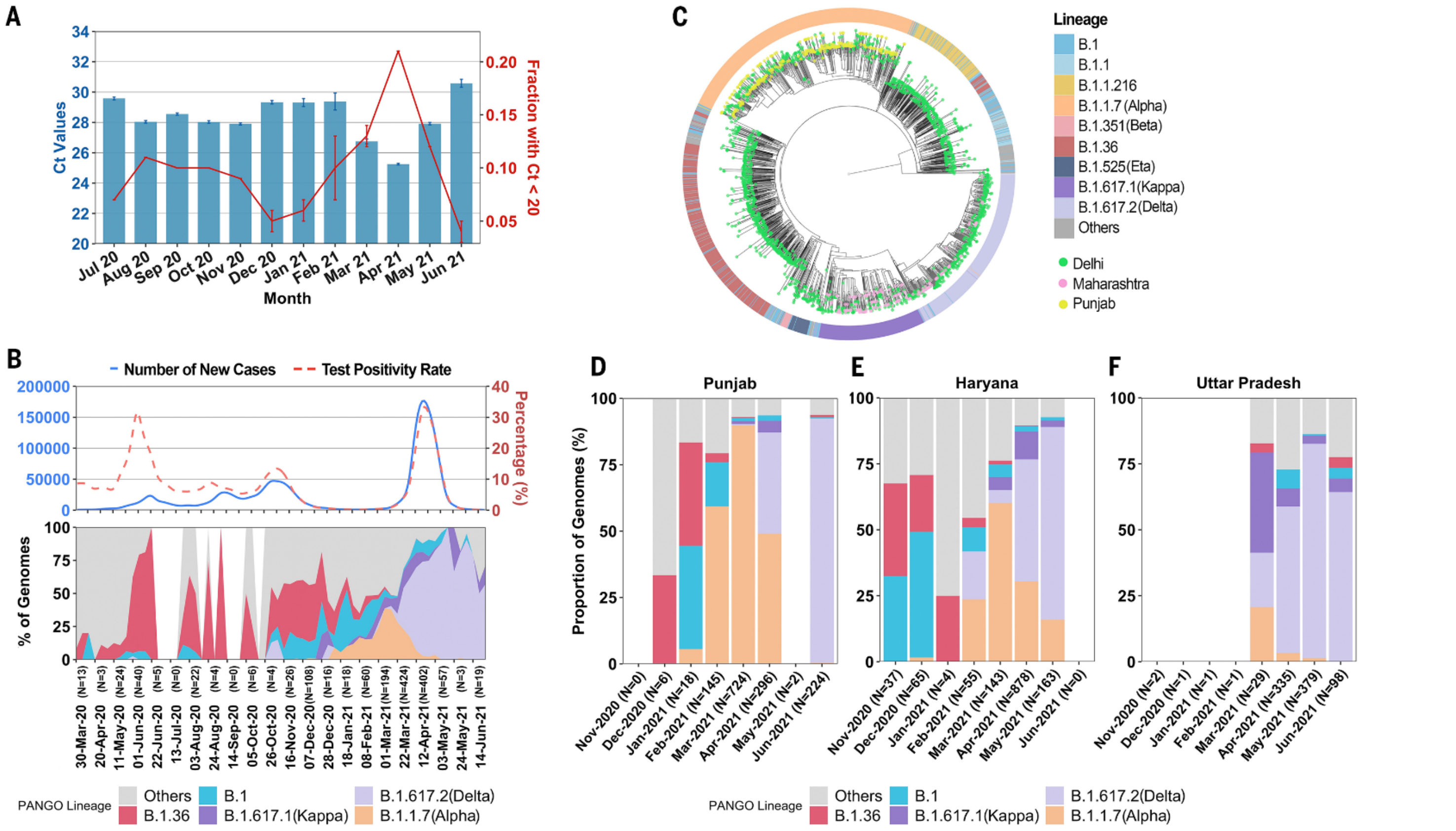

Fig. 3. Genomic-epidemiologic correlations. (**A**) Time-trends of Ct values (mean ± SE) and high viral load samples (proportion ± SE) for Orf1 gene (E gene data) (fig. S1). (**B**) Smoothed graph of main lineages in Delhi from March 2020 to May 2021 in biweekly increments. New cases and TPR are aligned and plotted on the same timeline. (**C**) Phylogenetic analysis for VOC strains between Delhi and states (Punjab and Maharashtra) with known VOC outbreaks before April 2021. Further analysis suggesting a super-spreading event for Alpha is shown in fig. S3. (**D** to **F**) Month-wise proportions of different lineages (*n* > 3) in states surrounding Delhi. Additional data are shown in figs. S4 and S5.

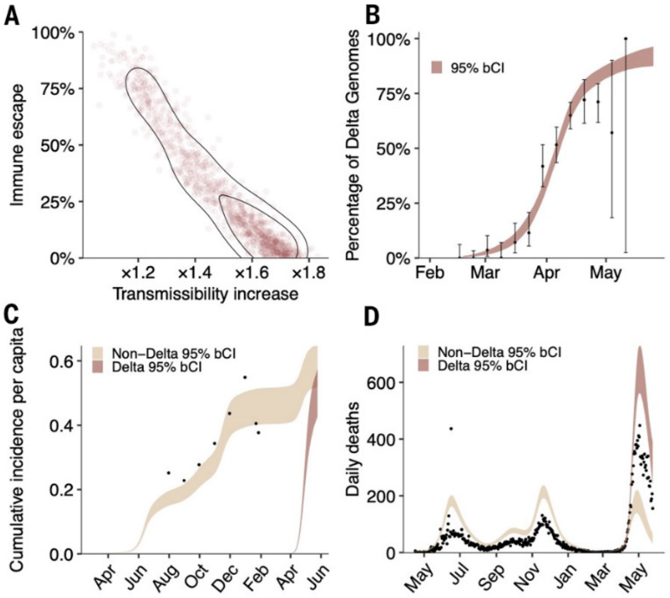

Fig. 4. Estimates of the epidemiological characteristic of the Delta variant. Values were inferred from a two category Bayesian transmission model fitted to mortality, serosurvey and genomic data from Delhi, India. (**A**) Joint posterior distribution, with isoclines corresponding to the 90% and 50% enclosures of posterior density of the Delta variant immune escape and transmissibility increase relative to non-Delta categories. Immune escape has a median of 20% with (10–50%) bCI50%, and transmissibility increase has a median of ×1.5 with (1.3–1.7) bCI50%. (**B**) Delta fraction over time inferred by the model. Black dots represent genome sampling data points, with exact binomial confidence intervals. (**C**) Serosurvey data (black dots) and inferred cumulative incidence for Delta and non-Delta variant categories. (**D**) Mortality data (black dots) and inferred deaths assuming 50% under reporting. Other under-ascertainment scenarios are presented in the supplementary materials.
