# Supplementary material for: Genomic characterization and epidemiology of an emerging SARS-CoV-2 variant in Delhi, India
Source: Science. 2021 Oct 14;374(6570):995–9. doi: 10.1126/science.abj9932 (PMC7612010; doi:10.1126/science.abj9932)
Supplement: Supplementary file 4 — Data S1 to S9 [file science.abj9932_data_s1_to_s9.zip › science.abj9932_data_s3.pdf]

We gratefully acknowledge the following Authors from the Originating laboratories responsible for obtaining the specimens, as well as the Submitting laboratories where the genome data were generated and shared via GISAID, on which this research is based.

All Submitters of data may be contacted directly via www.gisaid.org

Authors are sorted alphabetically.

Accession ID	Originating Laboratory	Submitting Laboratory	Authors
EPI_ISL_2502630, EPI_ISL_2502631, EPI_ISL_2502632, EPI_ISL_2502633, EPI_ISL_2502634, EPI_ISL_2502635, EPI_ISL_2502636, EPI_ISL_2502637, EPI_ISL_2502638, EPI_ISL_2502639, EPI_ISL_2502640, EPI_ISL_2502641, EPI_ISL_2502642, EPI_ISL_2502643, EPI_ISL_2502644, EPI_ISL_2502645, EPI_ISL_2502646, EPI_ISL_2502647, EPI_ISL_2502648, EPI_ISL_2502649, EPI_ISL_2502650, EPI_ISL_2502651, EPI_ISL_2502652, EPI_ISL_2502653, EPI_ISL_2502654, EPI_ISL_2502655, EPI_ISL_2502656, EPI_ISL_2502657, EPI_ISL_2502658, EPI_ISL_2502659, EPI_ISL_2502660, EPI_ISL_2502661, EPI_ISL_2502662, EPI_ISL_2502663, EPI_ISL_2502664, EPI_ISL_2502665, EPI_ISL_2502666, EPI_ISL_2696940, EPI_ISL_2696941, EPI_ISL_2696942, EPI_ISL_2696943, EPI_ISL_2696944, EPI_ISL_2696945, EPI_ISL_2727891, EPI_ISL_2727892, EPI_ISL_2727893, EPI_ISL_2727894, EPI_ISL_2727895, EPI_ISL_2727896, EPI_ISL_2727897, EPI_ISL_2727898, EPI_ISL_2727899, EPI_ISL_2790002, EPI_ISL_2790003, EPI_ISL_2790004			
see above	All India Institute of Medical Sciences Delhi Hospital	Virology Laboratory, AIIMS Delhi	Aashish Choudhary; Chitra Sarkar; Deepankar Srigyan; Dibyabhaba Pradhan; Jyoti Jethani; Lalit Dar; Lata Rani; Manish Soneja; Megha Brijwal; Nazneen Arif; Pooja Pandey; Puneet Kaur; Rakesh Lodha; Randeep Guleria; Ritu Gupta; Shivram Dhakad; Subrata Sinha; Sumedha Bagga
EPI_ISL_2100679, EPI_ISL_2100680, EPI_ISL_2100681, EPI_ISL_2100682, EPI_ISL_2100683, EPI_ISL_2100684, EPI_ISL_2100685, EPI_ISL_2100686, EPI_ISL_2100687, EPI_ISL_2100688, EPI_ISL_2100689, EPI_ISL_2100690, EPI_ISL_2100691, EPI_ISL_2100692, EPI_ISL_2100693, EPI_ISL_2100694, EPI_ISL_2100695, EPI_ISL_2100696, EPI_ISL_2100697, EPI_ISL_2100698, EPI_ISL_2100699, EPI_ISL_2100700, EPI_ISL_2105583, EPI_ISL_2105584, EPI_ISL_2105585			
see above	All India Institute of Medical Sciences, Ansari Nagar Delhi	CSIR-Institute of Genomics and Integrative Biology	Animesh Ray; Asangala Kamai; Ashwin Varadarajan; Ayush Goel; Bharathram Uppilli*; Devasish Desai; Manish Kumar; Mohammed Ahmed; Mohammed Faruq; Naveet Wig; Pooja Sharma*; Rajesh Pandey; Sarafaraz Alam; Saruchi Wadhwa; Satish Swain; Sheeba Saifi; Sushma Rajpoot; Umang Arora
EPI_ISL_1972130, EPI_ISL_1972131, EPI_ISL_1972132, EPI_ISL_1972133, EPI_ISL_1972134, EPI_ISL_1972135, EPI_ISL_1972136, EPI_ISL_1972137, EPI_ISL_1972138, EPI_ISL_1972139, EPI_ISL_1972140, EPI_ISL_1972141, EPI_ISL_1972142, EPI_ISL_1972143, EPI_ISL_1972144, EPI_ISL_1972145, EPI_ISL_1972146, EPI_ISL_1972147			
see above	All India Institute of Medical Sciences, Bhopal	All India Institute of Medical Sciences, Bhopal	Anvita Gupta Malhotra; Arun Raghuvanshi; Ashvini Kumar Yadav; Debasis Biswas and Sarman Singh; Dipesh Kale; Jitendra Singh; Prem Shankar; Shashwati Nema
EPI_ISL_2426190, EPI_ISL_2426191, EPI_ISL_2426192, EPI_ISL_2426193, EPI_ISL_2426194, EPI_ISL_2426195, EPI_ISL_2426196, EPI_ISL_2426197, EPI_ISL_2426198, EPI_ISL_2426199, EPI_ISL_2426200, EPI_ISL_2426201, EPI_ISL_2426202, EPI_ISL_2426203, EPI_ISL_2426204, EPI_ISL_2426205, EPI_ISL_2426206, EPI_ISL_2426207, EPI_ISL_2426208, EPI_ISL_2426209, EPI_ISL_2426210, EPI_ISL_2426211, EPI_ISL_2426212, EPI_ISL_2426213, EPI_ISL_2426214, EPI_ISL_2426215, EPI_ISL_2426216, EPI_ISL_2426217, EPI_ISL_2426218, EPI_ISL_2426219, EPI_ISL_2426220, EPI_ISL_2426221, EPI_ISL_2426222, EPI_ISL_2426223, EPI_ISL_2426224, EPI_ISL_2426225, EPI_ISL_2426226, EPI_ISL_2426227, EPI_ISL_2426228, EPI_ISL_2426229, EPI_ISL_2426230, EPI_ISL_2426231, EPI_ISL_2426232, EPI_ISL_2426233, EPI_ISL_2426234, EPI_ISL_2426235, EPI_ISL_2426236, EPI_ISL_2426237, EPI_ISL_2426238, EPI_ISL_2426239, EPI_ISL_2426240, EPI_ISL_2426241, EPI_ISL_2426242, EPI_ISL_2426243, EPI_ISL_2426244, EPI_ISL_2426245, EPI_ISL_2426247, EPI_ISL_2426248, EPI_ISL_2426249, EPI_ISL_2426250, EPI_ISL_2426251, EPI_ISL_2426252, EPI_ISL_2426253			
see above	All India Institute of Medical Sciences, Delhi	CSIR Institute of Genomics and Integrative Biology	Abhinav Jain; Afra Shamnath; Anjali Bajaj; Arvinden VR; Bani Jolly; COVID CBNAAT CORE GROUP; Kiran Bala; Mercy Rophina; Mohamed Imran; Mohit Kumar Divakar; Nayer Jamshed; Praveen Aggarwal; Rahul C. Bhoyar; Rama Chaudhry; Randeep Guleria; Ritu Gupta; Sridhar Sivasubbu; Subrata Sinha; Urvashi B Singh; Vigneshwar Senthivel; Vinod Scaria
EPI_ISL_2389628, EPI_ISL_2389630	B.J. Medical College and Civil Hospital, Ahmedabad	Gujarat Biotechnology Research Centre	Chaitanya Joshi; Dinesh Kumar; Janvi Raval; Madhvi Joshi; Nitesh Shah; Nitin Savaliya; Pranay Shah; Ramesh Pandit; Sonal Sharma; Twinkle Soni; Umang Mishra; Zarna Patel; Zuber Saiyed
EPI_ISL_1544114	B.J. Medical College and Civil hospital, Ahmedabad	Gujarat Biotechnology Research Centre	Chaitanya Joshi; Dinesh Kumar; Dipa Kinariwala; Janvi Raval; Kamlesh J Upadhyay; Madhvi Joshi; Nitesh Shah; Nitin Savaliya; Pranay Shah; Ramesh Pandit; Sanjay Kapadia; Sonal Sharma; Twinkle Soni; Umang Mishra; Zarna Patel; Zuber Saiyed
EPI_ISL_2373501, EPI_ISL_2373502, EPI_ISL_2373503, EPI_ISL_2373504, EPI_ISL_2373505, EPI_ISL_2373506, EPI_ISL_2373507, EPI_ISL_2373508, EPI_ISL_2373509, EPI_ISL_2373510, EPI_ISL_2373511, EPI_ISL_2373512, EPI_ISL_2373513, EPI_ISL_2373514, EPI_ISL_2373515, EPI_ISL_2373516, EPI_ISL_2373517, EPI_ISL_2373518, EPI_ISL_2373519, EPI_ISL_2373520, EPI_ISL_2373521, EPI_ISL_2373522, EPI_ISL_2373523, EPI_ISL_2373524, EPI_ISL_2373525, EPI_ISL_2373526, EPI_ISL_2373527, EPI_ISL_2373528, EPI_ISL_2373529, EPI_ISL_2373530, EPI_ISL_2373531, EPI_ISL_2373532, EPI_ISL_2373533, EPI_ISL_2373534, EPI_ISL_2373535, EPI_ISL_2373536, EPI_ISL_2373537, EPI_ISL_2373538, EPI_ISL_2373539, EPI_ISL_2373540, EPI_ISL_2373541, EPI_ISL_2373542, EPI_ISL_2373543, EPI_ISL_2373544, EPI_ISL_2373545, EPI_ISL_2373546, EPI_ISL_2373547, EPI_ISL_2373548, EPI_ISL_2373549, EPI_ISL_2373550, EPI_ISL_2373551, EPI_ISL_2373552, EPI_ISL_2373553, EPI_ISL_2373554, EPI_ISL_2373555, EPI_ISL_2373556, EPI_ISL_2373557, EPI_ISL_2373558, EPI_ISL_2373559, EPI_ISL_2373560, EPI_ISL_2373561, EPI_ISL_2373562, EPI_ISL_2373563, EPI_ISL_2373564, EPI_ISL_2373565, EPI_ISL_2373566, EPI_ISL_2373567, EPI_ISL_2373568, EPI_ISL_2373569, EPI_ISL_2373570, EPI_ISL_2373571, EPI_ISL_2373572, EPI_ISL_2373573, EPI_ISL_2373574, EPI_ISL_2373575, EPI_ISL_2373576, EPI_ISL_2373577, EPI_ISL_2373578, EPI_ISL_2373579, EPI_ISL_2373580, EPI_ISL_2373581, EPI_ISL_2373582, EPI_ISL_2373583, EPI_ISL_2373584, EPI_ISL_2373585, EPI_ISL_2373586, EPI_ISL_2373587, EPI_ISL_2373588, EPI_ISL_2373589, EPI_ISL_2373590, EPI_ISL_2373591, EPI_ISL_2373592, EPI_ISL_2373593, EPI_ISL_2373594, EPI_ISL_2373595, EPI_ISL_2373596, EPI_ISL_2373597, EPI_ISL_2373598, EPI_ISL_2373599, EPI_ISL_2373600, EPI_ISL_2373601, EPI_ISL_2373602, EPI_ISL_2373603, EPI_ISL_2373604, EPI_ISL_2373605, EPI_ISL_2373606, EPI_ISL_2373607, EPI_ISL_2373608, EPI_ISL_2373609, EPI_ISL_2373610, EPI_ISL_2373611, EPI_ISL_2373612, EPI_ISL_2373613, EPI_ISL_2400313, EPI_ISL_2400314, EPI_ISL_2400316, EPI_ISL_2400320, EPI_ISL_2400321, EPI_ISL_2400322, EPI_ISL_2400323, EPI_ISL_2400324, EPI_ISL_2400325, EPI_ISL_2400326, EPI_ISL_2400327, EPI_ISL_2400328, EPI_ISL_2400329, EPI_ISL_2400330, EPI_ISL_2400331, EPI_ISL_2400332			
see above	Banaras Hindu University	CSIR-Centre for Cellular and Molecular Biology-INSACOG	; Abhay Kumar Yadav; Abhishek Pathak; Ajay Kumar Yadav; Amareshwar Vodapalli; Ara Sreenivas; Archana Bharadwaj Siva; Ashish; B Himasri; Blessy B John; Chetan Sahni; Debashruti Das; Deepa Devadas; Divya Tej Sowpati; Gunjan Rai; Gyaneshwer Chaubey; Jaya Chakraborty; Karthik Bharadwaj Tallapaka; Lamuk Zaveri; Maneesha Upadhyay; Manpreet Kaur; Nitish Kumar Singh; Payel Mukherjee; Prajjval Pratap Singh; Priyoneel Basu; Rakesh K Mishra; Royana Singh; Saumya Singh; Saurabh Singh; Shani Vishwakarma; Sharath Chandra Thota; Shivam Tiwari; Shivani Mishra; Shreekant Verma; Sofia Banu; Surendra Pratap Mishra; Tribhuwan Mohan Mohapatra; Tulasi Nagabandi; Umesh Choudhary; Valli Nagalakshmi Undamatla; Vijay Kumar Shukla; Viswagithe S L; Yashvant Patel
EPI_ISL_1908685, EPI_ISL_1908686, EPI_ISL_1908687, EPI_ISL_1908688, EPI_ISL_1908689, EPI_ISL_1908690, EPI_ISL_1908691, EPI_ISL_1908692, EPI_ISL_1908693, EPI_ISL_1908694, EPI_ISL_1908695, EPI_ISL_1908696, EPI_ISL_1908697, EPI_ISL_1908698, EPI_ISL_1908699, EPI_ISL_1908700, EPI_ISL_1908701, EPI_ISL_1908702, EPI_ISL_1908703, EPI_ISL_1908704, EPI_ISL_1908705, EPI_ISL_1908706, EPI_ISL_1908707, EPI_ISL_1908708, EPI_ISL_1908709, EPI_ISL_1908710, EPI_ISL_1908711, EPI_ISL_1908712, EPI_ISL_1908713, EPI_ISL_1908714, EPI_ISL_1908715, EPI_ISL_1908716, EPI_ISL_1908717, EPI_ISL_1908718, EPI_ISL_1908719, EPI_ISL_1908720, EPI_ISL_1908721, EPI_ISL_1908722, EPI_ISL_1908723, EPI_ISL_1908724, EPI_ISL_1908725, EPI_ISL_1908726, EPI_ISL_1908727, EPI_ISL_1908728, EPI_ISL_1908729, EPI_ISL_1908730, EPI_ISL_1908731, EPI_ISL_1908732, EPI_ISL_1908733, EPI_ISL_1908734, EPI_ISL_1908736, EPI_ISL_1908737, EPI_ISL_1908738, EPI_ISL_1908739, EPI_ISL_1908740, EPI_ISL_1908741, EPI_ISL_1908742, EPI_ISL_1908743, EPI_ISL_1908744, EPI_ISL_1908745, EPI_ISL_1908746, EPI_ISL_1908747, EPI_ISL_1908748, EPI_ISL_1908749, EPI_ISL_1908750, EPI_ISL_1908751, EPI_ISL_1908752, EPI_ISL_1908753, EPI_ISL_1908754, EPI_ISL_1908755, EPI_ISL_1908756, EPI_ISL_1908757, EPI_ISL_1908758, EPI_ISL_1908759, EPI_ISL_1908760, EPI_ISL_1908761, EPI_ISL_1908762, EPI_ISL_1908763, EPI_ISL_1908764, EPI_ISL_1908765, EPI_ISL_1908766, EPI_ISL_1908767, EPI_ISL_1908768, EPI_ISL_1908769, EPI_ISL_1908770, EPI_ISL_1908771, EPI_ISL_1908772, EPI_ISL_1908773, EPI_ISL_1908774, EPI_ISL_1908775, EPI_ISL_1908776, EPI_ISL_1908777, EPI_ISL_1908778, EPI_ISL_1908779, EPI_ISL_1908780, EPI_ISL_1908781, EPI_ISL_1908782, EPI_ISL_1908783, EPI_ISL_1908784, EPI_ISL_1908785, EPI_ISL_1908786, EPI_ISL_1970088, EPI_ISL_1970089, EPI_ISL_1970090, EPI_ISL_1970091, EPI_ISL_1970092, EPI_ISL_1970093, EPI_ISL_1970094, EPI_ISL_1970095, EPI_ISL_1970096, EPI_ISL_1970097, EPI_ISL_1970098, EPI_ISL_1970099, EPI_ISL_1970100, EPI_ISL_1970101, EPI_ISL_1970102, EPI_ISL_1970103, EPI_ISL_1970104, EPI_ISL_1970105, EPI_ISL_1970106, EPI_ISL_1970107, EPI_ISL_1970108, EPI_ISL_1970109, EPI_ISL_1970110, EPI_ISL_1970111, EPI_ISL_1970112, EPI_ISL_1970113, EPI_ISL_1970114, EPI_ISL_1970115, EPI_ISL_1970116, EPI_ISL_1970117, EPI_ISL_1970118, EPI_ISL_1970119, EPI_ISL_1970120, EPI_ISL_1970121, EPI_ISL_1970122, EPI_ISL_1970123, EPI_ISL_1970124, EPI_ISL_1970125, EPI_ISL_1970126, EPI_ISL_1970127, EPI_ISL_1970128, EPI_ISL_1970129, EPI_ISL_1970130, EPI_ISL_1970131, EPI_ISL_1970132, EPI_ISL_1970133, EPI_ISL_1970134, EPI_ISL_1970135, EPI_ISL_1970136, EPI_ISL_1970137, EPI_ISL_1970138, EPI_ISL_1970139, EPI_ISL_1970140, EPI_ISL_1970141, EPI_ISL_1970142, EPI_ISL_1970144, EPI_ISL_1970145, EPI_ISL_1970146, EPI_ISL_1970147, EPI_ISL_1970148, EPI_ISL_1970149, EPI_ISL_1970150, EPI_ISL_1970151, EPI_ISL_1970152, EPI_ISL_1970153, EPI_ISL_1970154, EPI_ISL_1970155, EPI_ISL_1970156, EPI_ISL_1970157, EPI_ISL_1970158, EPI_ISL_1970159, EPI_ISL_1970160, EPI_ISL_1970161, EPI_ISL_1970162, EPI_ISL_1970163, EPI_ISL_1970164, EPI_ISL_1970165, EPI_ISL_1970166, EPI_ISL_1970167, EPI_ISL_1970168, EPI_ISL_1970169, EPI_ISL_1970170, EPI_ISL_1970171, EPI_ISL_1970172, EPI_ISL_1970173, EPI_ISL_1970174, EPI_ISL_1970175, EPI_ISL_1970176, EPI_ISL_1970177, EPI_ISL_1970178, EPI_ISL_1970179, EPI_ISL_1970180, EPI_ISL_1970181, EPI_ISL_1970182, EPI_ISL_1970183, EPI_ISL_1970184, EPI_ISL_1970185, EPI_ISL_1970186, EPI_ISL_1970187, EPI_ISL_1970188, EPI_ISL_1970189, EPI_ISL_1970190, EPI_ISL_1970191, EPI_ISL_1970192, EPI_ISL_1970193, EPI_ISL_1970194, EPI_ISL_1970195, EPI_ISL_1970196, EPI_ISL_1970197, EPI_ISL_1970198, EPI_ISL_1970199, EPI_ISL_1970200, EPI_ISL_1970201, EPI_ISL_1970202, EPI_ISL_1970203, EPI_ISL_1970204, EPI_ISL_1970205, EPI_ISL_1970206, EPI_ISL_1970207, EPI_ISL_1970208, EPI_ISL_1970209, EPI_ISL_1970210, EPI_ISL_1970211, EPI_ISL_1970212, EPI_ISL_1970213, EPI_ISL_1970214, EPI_ISL_1970215, EPI_ISL_1970216, EPI_ISL_1970217, EPI_ISL_1970218, EPI_ISL_1970220, EPI_ISL_1970221, EPI_ISL_1970222, EPI_ISL_1970223, EPI_ISL_1970224, EPI_ISL_1970225, EPI_ISL_1970226, EPI_ISL_1970227, EPI_ISL_1970228, EPI_ISL_1970229, EPI_ISL_1970230, EPI_ISL_1970231, EPI_ISL_1970232, EPI_ISL_1970233, EPI_ISL_1970234, EPI_ISL_1970235, EPI_ISL_1970236, EPI_ISL_1970237, EPI_ISL_1970238, EPI_ISL_1970239, EPI_ISL_1970240, EPI_ISL_1970241, EPI_ISL_1970242, EPI_ISL_1970243, EPI_ISL_1970244, EPI_ISL_1970245, EPI_ISL_1970246, EPI_ISL_1970247, EPI_ISL_1970248, EPI_ISL_1970249, EPI_ISL_1970250, EPI_ISL_1970251, EPI_ISL_1970252, EPI_ISL_1970253, EPI_ISL_1970254, EPI_ISL_1970255, EPI_ISL_1970256, EPI_ISL_1970257, EPI_ISL_1970258, EPI_ISL_1970259, EPI_ISL_1970260, EPI_ISL_1970261, EPI_ISL_1970262, EPI_ISL_1970263, EPI_ISL_1970264, EPI_ISL_1970265, EPI_ISL_1970266, EPI_ISL_1970267, EPI_ISL_1970268, EPI_ISL_1970269, EPI_ISL_1970270, EPI_ISL_1970271, EPI_ISL_1970272, EPI_ISL_1970273, EPI_ISL_1970274, EPI_ISL_1970275, EPI_ISL_1970276, EPI_ISL_1970277, EPI_ISL_1970278, EPI_ISL_1970279, EPI_ISL_1970280, EPI_ISL_1970281, EPI_ISL_1970282, EPI_ISL_1970283, EPI_ISL_1970284, EPI_ISL_1970285, EPI_ISL_1970286, EPI_ISL_1970287, EPI_ISL_1970288, EPI_ISL_1970289, EPI_ISL_1970290, EPI_ISL_1970291, EPI_ISL_1970292, EPI_ISL_1970293, EPI_ISL_1970294, EPI_ISL_1970295, EPI_ISL_1970296, EPI_ISL_1970297, EPI_ISL_1970298, EPI_ISL_1970299, EPI_ISL_1970300, EPI_ISL_1970301, EPI_ISL_1970302, EPI_ISL_1970303, EPI_ISL_1970304, EPI_ISL_1970305, EPI_ISL_1970306, EPI_ISL_1970307, EPI_ISL_1970308, EPI_ISL_1970309, EPI_ISL_1970310, EPI_ISL_1970311, EPI_ISL_1970312, EPI_ISL_1970313, EPI_ISL_1970314, EPI_ISL_1970315, EPI_ISL_1970316, EPI_ISL_1970317, EPI_ISL_1970318, EPI_ISL_1970319, EPI_ISL_1970320, EPI_ISL_1970321, EPI_ISL_1970322, EPI_ISL_1970323, EPI_ISL_1970324, EPI_ISL_1970325, EPI_ISL_1970326, EPI_ISL_1970327, EPI_ISL_1970328, EPI_ISL_1970329, EPI_ISL_1970330, EPI_ISL_1970331, EPI_ISL_1970332, EPI_ISL_1970333, EPI_ISL_1970334, EPI_ISL_1970335, EPI_ISL_1970336, EPI_ISL_1970337, EPI_ISL_1970338, EPI_ISL_1970339, EPI_ISL_1970340, EPI_ISL_1970341, EPI_ISL_1970342, EPI_ISL_1970343, EPI_ISL_2504529, EPI_ISL_2504530, EPI_ISL_2504531, EPI_ISL_2504532, EPI_ISL_2504533, EPI_ISL_2504534, EPI_ISL_2504535, EPI_ISL_2504536, EPI_ISL_2504537, EPI_ISL_2504538, EPI_ISL_2504539, EPI_ISL_2504540, EPI_ISL_2504541, EPI_ISL_2504542, EPI_ISL_2504543, EPI_ISL_2504544, EPI_ISL_2504545, EPI_ISL_2504546, EPI_ISL_2504547, EPI_ISL_2504548, EPI_ISL_2504549, EPI_ISL_2504550, EPI_ISL_2504551, EPI_ISL_2504552, EPI_ISL_2504553, EPI_ISL_2504554, EPI_ISL_2504555, EPI_ISL_2504556, EPI_ISL_2504557, EPI_ISL_2504558, EPI_ISL_2504559, EPI_ISL_2504560, EPI_ISL_2504561, EPI_ISL_2504562, EPI_ISL_2504563, EPI_ISL_2504564, EPI_ISL_2504565, EPI_ISL_2504566, EPI_ISL_2504567, EPI_ISL_2504568, EPI_ISL_2504569, EPI_ISL_2504570, EPI_ISL_2504571, EPI_ISL_2504572, EPI_ISL_2504573, EPI_ISL_2504574, EPI_ISL_2504575, EPI_ISL_2504576, EPI_ISL_2504577, EPI_ISL_2504578, EPI_ISL_2504579, EPI_ISL_2504580, EPI_ISL_2504581, EPI_ISL_2504582, EPI_ISL_2504583, EPI_ISL_2504584, EPI_ISL_2504585, EPI_ISL_2504586, EPI_ISL_2504587, EPI_ISL_2504588, EPI_ISL_2504589, EPI_ISL_2504590, EPI_ISL_2504591, EPI_ISL_2504592, EPI_ISL_2504593, EPI_ISL_2504594, EPI_ISL_2504595, EPI_ISL_2504596, EPI_ISL_2504597, EPI_ISL_2504598, EPI_ISL_2504599, EPI_ISL_2504600, EPI_ISL_2504601, EPI_ISL_2504602, EPI_ISL_2504603, EPI_ISL_2504604, EPI_ISL_2504605, EPI_ISL_2504606, EPI_ISL_2504607, EPI_ISL_2504608, EPI_ISL_2504609, EPI_ISL_2504610, EPI_ISL_2504611, EPI_ISL_2504612, EPI_ISL_2504613, EPI_ISL_2504614, EPI_ISL_2504615			
see above	Biotechnology Division, NCDC Delhi	NCDC Delhi, Biotechnology Division	Hema Gogia; Hemlata Lall; Kalaiarasan Ponnusamy; Mahesh S Dhar; Manoj K Singh; Meena Datta; Partha Rakshit; Preeti Madan; Priyanka Singh; Radhakrishnan V. S; Robin Marwal; Sandhya Kabra; Sujeet K Singh; Uma Sharma
EPI_ISL_2614645	CSIR Institute of Himalayan Bioresource Technology	CSIR Institute of Himalayan Bioresource Technology	Narendra Tirpude; Ravi Kumar; Vishal Acharya
EPI_ISL_3276866, EPI_ISL_3276867, EPI_ISL_3276868, EPI_ISL_3276869, EPI_ISL_3276870, EPI_ISL_3276871, EPI_ISL_3276872, EPI_ISL_3276873, EPI_ISL_3276874, EPI_ISL_3276876, EPI_ISL_3276877, EPI_ISL_3276878, EPI_ISL_3276879, EPI_ISL_3276880, EPI_ISL_3276881, EPI_ISL_3276882, EPI_ISL_3276883, EPI_ISL_3276884, EPI_ISL_3276885, EPI_ISL_3276886, EPI_ISL_3276887, EPI_ISL_3276888, EPI_ISL_3276889, EPI_ISL_3276890, EPI_ISL_3276891, EPI_ISL_3276892, EPI_ISL_3276893, EPI_ISL_3276894, EPI_ISL_3276895, EPI_ISL_3276896, EPI_ISL_3276897, EPI_ISL_3276898, EPI_ISL_3276899, EPI_ISL_3276900, EPI_ISL_3276901, EPI_ISL_3276902, EPI_ISL_3276903, EPI_ISL_3276904, EPI_ISL_3276905, EPI_ISL_3276906, EPI_ISL_3276907, EPI_ISL_3276908, EPI_ISL_3276909, EPI_ISL_3276910, EPI_ISL_3276911, EPI_ISL_3276912, EPI_ISL_3276913, EPI_ISL_3276914, EPI_ISL_3276915, EPI_ISL_3276916, EPI_ISL_3276917, EPI_ISL_3276918, EPI_ISL_3276919, EPI_ISL_3276920, EPI_ISL_3276921, EPI_ISL_3276922, EPI_ISL_3276923, EPI_ISL_3276924, EPI_ISL_3276925, EPI_ISL_3276926, EPI_ISL_3276927, EPI_ISL_3276928, EPI_ISL_3276929, EPI_ISL_3276930, EPI_ISL_3276931, EPI_ISL_3276932, EPI_ISL_3276933, EPI_ISL_3276934, EPI_ISL_3276935, EPI_ISL_3276936, EPI_ISL_3276937, EPI_ISL_3276938, EPI_ISL_3276939, EPI_ISL_3276940, EPI_ISL_3276941, EPI_ISL_3276942, EPI_ISL_3276943, EPI_ISL_3276944, EPI_ISL_3276945, EPI_ISL_3276946, EPI_ISL_3276947, EPI_ISL_3276948, EPI_ISL_3276949, EPI_ISL_3276950, EPI_ISL_3276951, EPI_ISL_3276952, EPI_ISL_3276953, EPI_ISL_3276954, EPI_ISL_3276955, EPI_ISL_3276956, EPI_ISL_3276957, EPI_ISL_3276958, EPI_ISL_3276959, EPI_ISL_3276960, EPI_ISL_3276961, EPI_ISL_3276962, EPI_ISL_3276963, EPI_ISL_3276964, EPI_ISL_3276965, EPI_ISL_3276966, EPI_ISL_3276967, EPI_ISL_3276968, EPI_ISL_3276969, EPI_ISL_3276970, EPI_ISL_3276971, EPI_ISL_3276972, EPI_ISL_3276973, EPI_ISL_3276974, EPI_ISL_3276975, EPI_ISL_3276976, EPI_ISL_3276977, EPI_ISL_3276978, EPI_ISL_3276979, EPI_ISL_3276980, EPI_ISL_3276981, EPI_ISL_3276982, EPI_ISL_3276983, EPI_ISL_3276984, EPI_ISL_3276985, EPI_ISL_3276986, EPI_ISL_3276987, EPI_ISL_3276988, EPI_ISL_3276989, EPI_ISL_3276990, EPI_ISL_3276991, EPI_ISL_3276992, EPI_ISL_3276993, EPI_ISL_3276994, EPI_ISL_3276995, EPI_ISL_3276996, EPI_ISL_3276997, EPI_ISL_3276998, EPI_ISL_3276999, EPI_ISL_3277000, EPI_ISL_3277001, EPI_ISL_3277002, EPI_ISL_3277003, EPI_ISL_3277004, EPI_ISL_3277005, EPI_ISL_3277006, EPI_ISL_3277007, EPI_ISL_3277008, EPI_ISL_3277009, EPI_ISL_3277010, EPI_ISL_3277011, EPI_ISL_3277012, EPI_ISL_3277013, EPI_ISL_3277014, EPI_ISL_3277015, EPI_ISL_3277016, EPI_ISL_3277017, EPI_ISL_3277018, EPI_ISL_3277019, EPI_ISL_3277020, EPI_ISL_3277021, EPI_ISL_3277022, EPI_ISL_3277023, EPI_ISL_3277024, EPI_ISL_3277025, EPI_ISL_3277026, EPI_ISL_3277027, EPI_ISL_3277028, EPI_ISL_3277029, EPI_ISL_3277030, EPI_ISL_3277031, EPI_ISL_3277032, EPI_ISL_3277033, EPI_ISL_3277034, EPI_ISL_3277035, EPI_ISL_3277036, EPI_ISL_3277037, EPI_ISL_3277038, EPI_ISL_3277039, EPI_ISL_3277040, EPI_ISL_3277041, EPI_ISL_3277042, EPI_ISL_3277043, EPI_ISL_3277044, EPI_ISL_3277045, EPI_ISL_3277046, EPI_ISL_3277047, EPI_ISL_3277048, EPI_ISL_3277049, EPI_ISL_3277050, EPI_ISL_3277051, EPI_ISL_3277052, EPI_ISL_3277053, EPI_ISL_3277054, EPI_ISL_3277055, EPI_ISL_3277056, EPI_ISL_3277057, EPI_ISL_3277058, EPI_ISL_3277059, EPI_ISL_3277060, EPI_ISL_3277061, EPI_ISL_3277062, EPI_ISL_3277063, EPI_ISL_3277064, EPI_ISL_3277065, EPI_ISL_3277066, EPI_ISL_3277067, EPI_ISL_3277068, EPI_ISL_3277069, EPI_ISL_3277070, EPI_ISL_3277071, EPI_ISL_3277072, EPI_ISL_3277073, EPI_ISL_3277074, EPI_ISL_3277075, EPI_ISL_3277076, EPI_ISL_3277077, EPI_ISL_3277078, EPI_ISL_3277079, EPI_ISL_3277080, EPI_ISL_3277081, EPI_ISL_3277082, EPI_ISL_3277083, EPI_ISL_3277084, EPI_ISL_3277085, EPI_ISL_3277086, EPI_ISL_3277087, EPI_ISL_3277088, EPI_ISL_3277089, EPI_ISL_3277090, EPI_ISL_3277091, EPI_ISL_3277092, EPI_ISL_3277094, EPI_ISL_3277095, EPI_ISL_3277096, EPI_ISL_3277097, EPI_ISL_3277098, EPI_ISL_3277099, EPI_ISL_3277100, EPI_ISL_3277101, EPI_ISL_3277102, EPI_ISL_3277103, EPI_ISL_3277104, EPI_ISL_3277105, EPI_ISL_3277106, EPI_ISL_3277107, EPI_ISL_3277108, EPI_ISL_3277109, EPI_ISL_3277110, EPI_ISL_3277111, EPI_ISL_3277112, EPI_ISL_3277113, EPI_ISL_3277114, EPI_ISL_3277115, EPI_ISL_3277116, EPI_ISL_3277117, EPI_ISL_3277118, EPI_ISL_3277119, EPI_ISL_3277120, EPI_ISL_3277121, EPI_ISL_3277122, EPI_ISL_3277123, EPI_ISL_3277124, EPI_ISL_3277125, EPI_ISL_3277126, EPI_ISL_3277127, EPI_ISL_3277128, EPI_ISL_3277129, EPI_ISL_3277130, EPI_ISL_3277131, EPI_ISL_3277132, EPI_ISL_3277133, EPI_ISL_3277134, EPI_ISL_3277135, EPI_ISL_3277136, EPI_ISL_3277137, EPI_ISL_3277138, EPI_ISL_3277139			
see above	Clinical Virology Laboratory, Institute of Liver and Biliary Sciences	ILBS	Amit Pandey; Chhagan Bihari Sharma; Diptanu Paul; Ekta Gupta; Reshu Agarwal; Shiv Kumar Sarin; Varun Suroliya
EPI_ISL_578697, EPI_ISL_605799, EPI_ISL_605800, EPI_ISL_605801, EPI_ISL_605802, EPI_ISL_605804, EPI_ISL_605805, EPI_ISL_605806, EPI_ISL_605807, EPI_ISL_605808, EPI_ISL_605809, EPI_ISL_605810, EPI_ISL_605813, EPI_ISL_605814, EPI_ISL_605815, EPI_ISL_2424135, EPI_ISL_2426145, EPI_ISL_2426146, EPI_ISL_2426147, EPI_ISL_2426148, EPI_ISL_2426149, EPI_ISL_2426150, EPI_ISL_2426151, EPI_ISL_2426152, EPI_ISL_2426153, EPI_ISL_2426154, EPI_ISL_2426155, EPI_ISL_2426156, EPI_ISL_2426157, EPI_ISL_2426158, EPI_ISL_2426159, EPI_ISL_2426160, EPI_ISL_2426161, EPI_ISL_2426162, EPI_ISL_2426163, EPI_ISL_2426164, EPI_ISL_2426165, EPI_ISL_2426166, EPI_ISL_2426167, EPI_ISL_2426168, EPI_ISL_2426169, EPI_ISL_2426170, EPI_ISL_2426171, EPI_ISL_2426172, EPI_ISL_2426173, EPI_ISL_2426174, EPI_ISL_2426175, EPI_ISL_2426176, EPI_ISL_2426177, EPI_ISL_2426178, EPI_ISL_2426179, EPI_ISL_2426180, EPI_ISL_2426181, EPI_ISL_2426182, EPI_ISL_2426183, EPI_ISL_2426184, EPI_ISL_2426185, EPI_ISL_2426186, EPI_ISL_2426187, EPI_ISL_2426188, EPI_ISL_2426189			
see above	Clinical Virology Laboratory, Institute of Liver and Biliary Sciences	ILBS - IGIB	Abhishek Padhi; Amit Pandey; Anurag Agrawal; Aparna S; Bharathram Uppili; Chhagan Bihari Sharma; Ekta Gupta; Jaswinder Singh Maras; Manish Kumar; Mohammed Faruq; Neha Jha; Partha Chattopadhyay; Pooja Sharma; Pratibha Kale; Priti Devi; Priyanka Mehta; Rajesh Pandey; Ranjeet Maurya; Reshu Agarwal; Sarfaraz Alam; Sheeba Saifi; Sheetalnath Rooge; Shiv Kumar Sarin; Shridhar Sivasubbu; Shvetank Sharma; Vinod Scaria
EPI_ISL_3276875	Clinical Virology Laboratory, Institute of Liver and Biliary Sciences	ILBS on behalf of 'Proyecto Argentino Interinstitucional de genomica de SARS-CoV-2' (PAIS Consortium)	Amit Pandey; Chhagan Bihari Sharma; Diptanu Paul; Ekta Gupta; Reshu Agarwal; Shiv Kumar Sarin; Varun Suroliya
EPI_ISL_2100635, EPI_ISL_2100636, EPI_ISL_2100637, EPI_ISL_2100638, EPI_ISL_2100639, EPI_ISL_2100640, EPI_ISL_2100641, EPI_ISL_2100642, EPI_ISL_2100643, EPI_ISL_2100644, EPI_ISL_2100645, EPI_ISL_2100646, EPI_ISL_2100647, EPI_ISL_2100648, EPI_ISL_2100649, EPI_ISL_2100650, EPI_ISL_2100651, EPI_ISL_2100652, EPI_ISL_2100653, EPI_ISL_2100654, EPI_ISL_2100655, EPI_ISL_2100656, EPI_ISL_2100657, EPI_ISL_2100658, EPI_ISL_2100659, EPI_ISL_2100660, EPI_ISL_2100661, EPI_ISL_2100662, EPI_ISL_2100663, EPI_ISL_2100664, EPI_ISL_2100665, EPI_ISL_2100666, EPI_ISL_2100667, EPI_ISL_2100668, EPI_ISL_2100669, EPI_ISL_2100670, EPI_ISL_2100671, EPI_ISL_2100672, EPI_ISL_2100673, EPI_ISL_2100674, EPI_ISL_2100675, EPI_ISL_2100676, EPI_ISL_2100677, EPI_ISL_2100678, EPI_ISL_2105576, EPI_ISL_2105577, EPI_ISL_2105578, EPI_ISL_2105579, EPI_ISL_2105580, EPI_ISL_2105581, EPI_ISL_2105582, EPI_ISL_2106198, EPI_ISL_2158575, EPI_ISL_2158576, EPI_ISL_2158577, EPI_ISL_2158578, EPI_ISL_2158579, EPI_ISL_2158580, EPI_ISL_2158581, EPI_ISL_2158582, EPI_ISL_2158583, EPI_ISL_2158584, EPI_ISL_2158585, EPI_ISL_2158586, EPI_ISL_2158587, EPI_ISL_2158588, EPI_ISL_2158589, EPI_ISL_2158590, EPI_ISL_2158591, EPI_ISL_2158592, EPI_ISL_2158593, EPI_ISL_2158594, EPI_ISL_2158595, EPI_ISL_2158596, EPI_ISL_2225586, EPI_ISL_2225587, EPI_ISL_2225588, EPI_ISL_2225589, EPI_ISL_2225590, EPI_ISL_2225591, EPI_ISL_2225592, EPI_ISL_2225593, EPI_ISL_2225594, EPI_ISL_2225595, EPI_ISL_2225596, EPI_ISL_2225597, EPI_ISL_2225598, EPI_ISL_2225599, EPI_ISL_2225600			
see above	Delhi North District	CSIR-Institute of Genomics and Integrative Biology	Animesh Ray; Aparna; Asangala Kamai; Ashwin Varadarajan; Ayush Goel; Bharathram Uppilli*; Devasish Desai; Manish Kumar; Mohammed Ahmed; Mohammed Faruq; Naveet Wig; Pooja Sharma*; Rajesh Pandey; Sana Zahra; Sarafaraz Alam; Saruchi Wadhwa; Satish Swain; Sheeba Saifi; Sushma Rajpoot; Umang Arora
EPI_ISL_1844693, EPI_ISL_1844698, EPI_ISL_1844703, EPI_ISL_1844707, EPI_ISL_1844711	Department of Microbiology, Dr YSPGMC	Biotechnology Division, NCDC	Hema Gogia; Hemlata Lall; Kalaiarasan Ponnusamy; Mahesh S Dhar; Manoj K Singh; Meena Datta; Partha Rakshit; Preeti Madan; Priyanka Singh; Radhakrishnan V. S; Robin Marwal; Sandhya Kabra; Sujeet K Singh; Uma Sharma
EPI_ISL_459911, EPI_ISL_459912, EPI_ISL_459913, EPI_ISL_459914, EPI_ISL_459915, EPI_ISL_459916, EPI_ISL_459917, EPI_ISL_459918, EPI_ISL_459919, EPI_ISL_459920, EPI_ISL_459921, EPI_ISL_459922, EPI_ISL_459923, EPI_ISL_459924, EPI_ISL_459925, EPI_ISL_459931, EPI_ISL_459932, EPI_ISL_459933, EPI_ISL_459934, EPI_ISL_459935, EPI_ISL_459936, EPI_ISL_459937, EPI_ISL_459938, EPI_ISL_459939, EPI_ISL_459940, EPI_ISL_459941, EPI_ISL_459942, EPI_ISL_459943, EPI_ISL_459944, EPI_ISL_459945, EPI_ISL_459946, EPI_ISL_459947, EPI_ISL_459948, EPI_ISL_459949, EPI_ISL_459950, EPI_ISL_459951, EPI_ISL_459952			

see above	Devki Devi Foundation, a unit of Max Healthcare	CSIR-IGIB/Max	Anurag Agrawal; Bansidhar Tarai; Bharathram Uppili; Mitali Mukerji; Mohammed Faruq; Nishu Tyagi; Pooja Sharma; Poonam Das; Rajesh Pandey#; Samreen Siddiqui; Saruchi Wadhwa; Sujeet Jha; Vinita Jha; Vivekanand A

EPI_ISL_1703720, EPI_ISL_1703962, EPI_ISL_1703963, EPI_ISL_1704055, EPI_ISL_1704056, EPI_ISL_1704057, EPI_ISL_1704658, EPI_ISL_1704659, EPI_ISL_1704660, EPI_ISL_1704661, EPI_ISL_1704662, EPI_ISL_1704663, EPI_ISL_1704664, EPI_ISL_1704665, EPI_ISL_1704666, EPI_ISL_2546295

see above	ICMR-National Institute of Virology - INSACOG	NIV Influenza	Dr. Varsha Potdar; Dr. Varsha Potdar and NIC Team

EPI_ISL_2878570, EPI_ISL_2878571, EPI_ISL_2878572, EPI_ISL_2878573, EPI_ISL_2878574, EPI_ISL_2878575, EPI_ISL_2878576, EPI_ISL_2878577, EPI_ISL_2878578, EPI_ISL_2878579, EPI_ISL_2878580, EPI_ISL_2878582, EPI_ISL_2878584, EPI_ISL_2878585, EPI_ISL_2878586, EPI_ISL_2878587, EPI_ISL_2878588, EPI_ISL_2878589, EPI_ISL_2878590, EPI_ISL_2878591, EPI_ISL_2878592, EPI_ISL_2878593,
EPI_ISL_2878594, EPI_ISL_2878595, EPI_ISL_2878596, EPI_ISL_2878597, EPI_ISL_2878598, EPI_ISL_2878599, EPI_ISL_2878600, EPI_ISL_2878601, EPI_ISL_2878602, EPI_ISL_2878603, EPI_ISL_2878604, EPI_ISL_2878605, EPI_ISL_2878606, EPI_ISL_2878607, EPI_ISL_2878608, EPI_ISL_2878609, EPI_ISL_2878610, EPI_ISL_2878611, EPI_ISL_2878612, EPI_ISL_2878613, EPI_ISL_2878614, EPI_ISL_2878615,
EPI_ISL_2878616, EPI_ISL_2878617, EPI_ISL_2878618, EPI_ISL_2878619, EPI_ISL_2878620, EPI_ISL_2878621, EPI_ISL_2878622, EPI_ISL_2878623, EPI_ISL_2878624, EPI_ISL_2878625, EPI_ISL_2878626, EPI_ISL_2878627, EPI_ISL_2878628, EPI_ISL_2878629, EPI_ISL_2878630, EPI_ISL_2878631, EPI_ISL_2878632, EPI_ISL_2878633, EPI_ISL_2878634, EPI_ISL_2878635, EPI_ISL_2878636, EPI_ISL_2878637,
EPI_ISL_2878638, EPI_ISL_2878639, EPI_ISL_2878640, EPI_ISL_2878641, EPI_ISL_2878642, EPI_ISL_2878643, EPI_ISL_2878644, EPI_ISL_2878645, EPI_ISL_2878657, EPI_ISL_2878658, EPI_ISL_2878659, EPI_ISL_2878660, EPI_ISL_2878661, EPI_ISL_2878662, EPI_ISL_2878663, EPI_ISL_2878664, EPI_ISL_2878665, EPI_ISL_2878666, EPI_ISL_2878667, EPI_ISL_2878668, EPI_ISL_2878669, EPI_ISL_2878670,
EPI_ISL_2878671, EPI_ISL_2878672, EPI_ISL_2878673, EPI_ISL_2878674, EPI_ISL_2878675, EPI_ISL_2878676, EPI_ISL_2878677, EPI_ISL_2878678, EPI_ISL_2878679, EPI_ISL_2878695, EPI_ISL_2878696, EPI_ISL_2878697, EPI_ISL_2878698, EPI_ISL_2878699, EPI_ISL_2878700, EPI_ISL_2878701, EPI_ISL_2878702, EPI_ISL_2878706, EPI_ISL_2878707, EPI_ISL_2878708, EPI_ISL_2878709, EPI_ISL_2878710,
EPI_ISL_2879069, EPI_ISL_2879074, EPI_ISL_2879170, EPI_ISL_2879171, EPI_ISL_2879180, EPI_ISL_2879181, EPI_ISL_2879182, EPI_ISL_2879241, EPI_ISL_2879242, EPI_ISL_2879243, EPI_ISL_2879244, EPI_ISL_2879245, EPI_ISL_2879250, EPI_ISL_2879251, EPI_ISL_2879252, EPI_ISL_2879253, EPI_ISL_2879254, EPI_ISL_2879255, EPI_ISL_2879256, EPI_ISL_2879257, EPI_ISL_2879258, EPI_ISL_2879259,
EPI_ISL_2879260, EPI_ISL_2879261, EPI_ISL_2879262, EPI_ISL_2879263, EPI_ISL_2879264, EPI_ISL_2879265, EPI_ISL_2879266, EPI_ISL_2879275, EPI_ISL_2879276, EPI_ISL_2879277, EPI_ISL_2879278, EPI_ISL_2879279, EPI_ISL_2879280, EPI_ISL_2879281, EPI_ISL_2879282, EPI_ISL_2879283, EPI_ISL_2879284, EPI_ISL_2879285, EPI_ISL_2879286, EPI_ISL_2879287, EPI_ISL_2879288, EPI_ISL_2879289,
EPI_ISL_2879290, EPI_ISL_2879291, EPI_ISL_2879292, EPI_ISL_2879293, EPI_ISL_2879294, EPI_ISL_2879295, EPI_ISL_2879296, EPI_ISL_2879297, EPI_ISL_2879298, EPI_ISL_2879299, EPI_ISL_2879300, EPI_ISL_2879301, EPI_ISL_2879302, EPI_ISL_2879303, EPI_ISL_2879304, EPI_ISL_2879305, EPI_ISL_2879306, EPI_ISL_2879307, EPI_ISL_2879308, EPI_ISL_2879309, EPI_ISL_2879310, EPI_ISL_2879311,
EPI_ISL_2879312, EPI_ISL_2879313, EPI_ISL_2879314, EPI_ISL_2879315, EPI_ISL_2879316, EPI_ISL_2879317, EPI_ISL_2879318, EPI_ISL_2879319, EPI_ISL_2879320, EPI_ISL_2879321, EPI_ISL_2879322, EPI_ISL_2879323, EPI_ISL_2879324, EPI_ISL_2879325, EPI_ISL_2879326, EPI_ISL_2879327, EPI_ISL_2879328, EPI_ISL_2879329, EPI_ISL_2879330, EPI_ISL_2879331, EPI_ISL_2879332, EPI_ISL_2879333,
EPI_ISL_2879334, EPI_ISL_2879335, EPI_ISL_2879336, EPI_ISL_2879337, EPI_ISL_2879338, EPI_ISL_2879339, EPI_ISL_2879340, EPI_ISL_2879341, EPI_ISL_2879342, EPI_ISL_2879343, EPI_ISL_2879344, EPI_ISL_2879345, EPI_ISL_2879346, EPI_ISL_2879347, EPI_ISL_2879348, EPI_ISL_2879356, EPI_ISL_2879357, EPI_ISL_2879358, EPI_ISL_2879359, EPI_ISL_2879360, EPI_ISL_2879361, EPI_ISL_2879362,
EPI_ISL_2879376, EPI_ISL_2879377, EPI_ISL_2879378, EPI_ISL_2879379, EPI_ISL_2879380, EPI_ISL_2879381, EPI_ISL_2879383, EPI_ISL_2879384, EPI_ISL_2879385, EPI_ISL_2879386, EPI_ISL_2879387, EPI_ISL_2879389, EPI_ISL_2879390, EPI_ISL_2879391, EPI_ISL_2879392, EPI_ISL_2879393, EPI_ISL_2879394, EPI_ISL_2879395, EPI_ISL_2879396, EPI_ISL_2879397, EPI_ISL_2879398, EPI_ISL_2879399,
EPI_ISL_2879400, EPI_ISL_2879401, EPI_ISL_2879402, EPI_ISL_2879403, EPI_ISL_2879404, EPI_ISL_2879405, EPI_ISL_2879406, EPI_ISL_2879407, EPI_ISL_2879408, EPI_ISL_2879409, EPI_ISL_2879410, EPI_ISL_2879411, EPI_ISL_2879412, EPI_ISL_2879413, EPI_ISL_2879414, EPI_ISL_2879415, EPI_ISL_2879416, EPI_ISL_2879418, EPI_ISL_2879419, EPI_ISL_2879420, EPI_ISL_2879421, EPI_ISL_2879422,
EPI_ISL_2879423, EPI_ISL_2879424, EPI_ISL_2879425, EPI_ISL_2879426, EPI_ISL_2879427, EPI_ISL_2879428, EPI_ISL_2879429, EPI_ISL_2879430, EPI_ISL_2879431, EPI_ISL_2879432, EPI_ISL_2879446, EPI_ISL_2879447, EPI_ISL_2879448, EPI_ISL_2879449, EPI_ISL_2879450, EPI_ISL_2879451, EPI_ISL_2879452, EPI_ISL_2879453, EPI_ISL_2879454, EPI_ISL_2879455, EPI_ISL_2879485, EPI_ISL_2879486,
EPI_ISL_2879487, EPI_ISL_2879488, EPI_ISL_2879489, EPI_ISL_2879490, EPI_ISL_2879491, EPI_ISL_2879492, EPI_ISL_2879493, EPI_ISL_2879494, EPI_ISL_2879495, EPI_ISL_2879496, EPI_ISL_2879497, EPI_ISL_2879498, EPI_ISL_2879499, EPI_ISL_2879500, EPI_ISL_2879501, EPI_ISL_2879502, EPI_ISL_2879503, EPI_ISL_2879504, EPI_ISL_2879505, EPI_ISL_2879506, EPI_ISL_2879507, EPI_ISL_2879508,
EPI_ISL_2879509, EPI_ISL_2879510, EPI_ISL_2879545, EPI_ISL_2879546, EPI_ISL_2879547, EPI_ISL_2879548, EPI_ISL_2879549, EPI_ISL_2879550, EPI_ISL_2879551, EPI_ISL_2879552, EPI_ISL_2879553, EPI_ISL_2879554, EPI_ISL_2879555, EPI_ISL_2879556, EPI_ISL_2879557, EPI_ISL_2879558, EPI_ISL_2879559, EPI_ISL_2879560, EPI_ISL_2879561, EPI_ISL_2879562, EPI_ISL_2879563, EPI_ISL_2879564,
EPI_ISL_2879565, EPI_ISL_2879566, EPI_ISL_2879567, EPI_ISL_2879568, EPI_ISL_2879569, EPI_ISL_2879570, EPI_ISL_2879571, EPI_ISL_2879572, EPI_ISL_2879573, EPI_ISL_2879574, EPI_ISL_2879575, EPI_ISL_2879576, EPI_ISL_2879577, EPI_ISL_2879578, EPI_ISL_2879579, EPI_ISL_2879580, EPI_ISL_2879581, EPI_ISL_2879582, EPI_ISL_2879583, EPI_ISL_2879584, EPI_ISL_2879585, EPI_ISL_2879586,
EPI_ISL_2879587, EPI_ISL_2879588, EPI_ISL_2879589, EPI_ISL_2879590, EPI_ISL_2879591, EPI_ISL_2879592, EPI_ISL_2879593, EPI_ISL_2879594, EPI_ISL_2879613, EPI_ISL_2879614, EPI_ISL_2879615, EPI_ISL_2879616, EPI_ISL_2879617, EPI_ISL_2879618, EPI_ISL_2879641, EPI_ISL_2879642, EPI_ISL_2879643, EPI_ISL_2879644, EPI_ISL_2879645, EPI_ISL_2879646, EPI_ISL_2879647, EPI_ISL_2879648,
EPI_ISL_2879649, EPI_ISL_2879650, EPI_ISL_2879651, EPI_ISL_2879652, EPI_ISL_2879653, EPI_ISL_2879654, EPI_ISL_2879655, EPI_ISL_2879656, EPI_ISL_2879657, EPI_ISL_2879658, EPI_ISL_2879659, EPI_ISL_2879660, EPI_ISL_2879661, EPI_ISL_2879662, EPI_ISL_2879663, EPI_ISL_2879664, EPI_ISL_2879665, EPI_ISL_2879678, EPI_ISL_2879679, EPI_ISL_2879680, EPI_ISL_2879690, EPI_ISL_2879691,
EPI_ISL_2879692, EPI_ISL_2879693, EPI_ISL_2879694, EPI_ISL_2879695, EPI_ISL_2879696, EPI_ISL_2879697, EPI_ISL_2879698, EPI_ISL_2879699, EPI_ISL_2879700, EPI_ISL_2879701, EPI_ISL_2879702, EPI_ISL_2879703, EPI_ISL_2879704, EPI_ISL_2879705, EPI_ISL_2879706, EPI_ISL_2879707, EPI_ISL_2879708, EPI_ISL_2879709, EPI_ISL_2879710, EPI_ISL_2879711, EPI_ISL_2879712, EPI_ISL_2879713,
EPI_ISL_2879714, EPI_ISL_2879715, EPI_ISL_2879716, EPI_ISL_2879717, EPI_ISL_2879718, EPI_ISL_2879719, EPI_ISL_2879720, EPI_ISL_2879721, EPI_ISL_2879722, EPI_ISL_2879723, EPI_ISL_2879724, EPI_ISL_2879725, EPI_ISL_2879732, EPI_ISL_2879733, EPI_ISL_2879736, EPI_ISL_2879737, EPI_ISL_2879738, EPI_ISL_2879739, EPI_ISL_2879740, EPI_ISL_2879741, EPI_ISL_2879742, EPI_ISL_2879743,
EPI_ISL_2879744, EPI_ISL_2879749, EPI_ISL_2879750, EPI_ISL_2879751, EPI_ISL_2879752, EPI_ISL_2879753, EPI_ISL_2879754, EPI_ISL_2879755, EPI_ISL_2879781, EPI_ISL_2879787, EPI_ISL_2879788, EPI_ISL_2879789, EPI_ISL_2879790, EPI_ISL_2879791, EPI_ISL_2879792, EPI_ISL_2879793, EPI_ISL_2879794, EPI_ISL_2879795, EPI_ISL_2879796, EPI_ISL_2879797, EPI_ISL_2879798, EPI_ISL_2879799,
EPI_ISL_2879800, EPI_ISL_2879801, EPI_ISL_2879802, EPI_ISL_2879803, EPI_ISL_2879804, EPI_ISL_2879805, EPI_ISL_2879806, EPI_ISL_2879807, EPI_ISL_2879808, EPI_ISL_2879809, EPI_ISL_2879810, EPI_ISL_2879811, EPI_ISL_2879812, EPI_ISL_2879813, EPI_ISL_2879814, EPI_ISL_2879815, EPI_ISL_2879816, EPI_ISL_2879817, EPI_ISL_2879818, EPI_ISL_2879819, EPI_ISL_2879820, EPI_ISL_2879821,
EPI_ISL_2879822, EPI_ISL_2879823, EPI_ISL_2879824, EPI_ISL_2879825, EPI_ISL_2879826, EPI_ISL_2879827, EPI_ISL_2879828, EPI_ISL_2879829, EPI_ISL_2879830, EPI_ISL_2879831, EPI_ISL_2879832, EPI_ISL_2879833, EPI_ISL_2879834, EPI_ISL_2879835, EPI_ISL_2879836, EPI_ISL_2879837, EPI_ISL_2879838, EPI_ISL_2879839, EPI_ISL_2879840, EPI_ISL_2879841, EPI_ISL_2879842, EPI_ISL_2879843,
EPI_ISL_2879844, EPI_ISL_2879845, EPI_ISL_2879846, EPI_ISL_2879847, EPI_ISL_2879848, EPI_ISL_2879849, EPI_ISL_2879850, EPI_ISL_2879851, EPI_ISL_2879852, EPI_ISL_2879853, EPI_ISL_2879854, EPI_ISL_2879855, EPI_ISL_2879856, EPI_ISL_2879857, EPI_ISL_2879858, EPI_ISL_2879859, EPI_ISL_2879860, EPI_ISL_2879861, EPI_ISL_2879862, EPI_ISL_2879863, EPI_ISL_2879864, EPI_ISL_2879865,
EPI_ISL_2879866, EPI_ISL_2879867, EPI_ISL_2879868, EPI_ISL_2879869, EPI_ISL_2879870, EPI_ISL_2879871, EPI_ISL_2879872, EPI_ISL_2879873, EPI_ISL_2879874, EPI_ISL_2879875, EPI_ISL_2879876, EPI_ISL_2879877, EPI_ISL_2879878, EPI_ISL_2879879, EPI_ISL_2879880, EPI_ISL_2879881, EPI_ISL_2879913, EPI_ISL_2879914, EPI_ISL_2879915, EPI_ISL_2879916, EPI_ISL_2879917, EPI_ISL_2879918,
EPI_ISL_2879919, EPI_ISL_2879920, EPI_ISL_2879921, EPI_ISL_2879922, EPI_ISL_2879923, EPI_ISL_2879924, EPI_ISL_2879925, EPI_ISL_2879926, EPI_ISL_2879927, EPI_ISL_2879928, EPI_ISL_2879929, EPI_ISL_2879930, EPI_ISL_2879931, EPI_ISL_2879932, EPI_ISL_2879933, EPI_ISL_2879934, EPI_ISL_2879935, EPI_ISL_2879936, EPI_ISL_2879937, EPI_ISL_2879938, EPI_ISL_2879939, EPI_ISL_2879940,
EPI_ISL_2879941, EPI_ISL_2879942, EPI_ISL_2879943, EPI_ISL_2879944, EPI_ISL_2879945, EPI_ISL_2879946, EPI_ISL_2879947, EPI_ISL_2879948, EPI_ISL_2879949, EPI_ISL_2879950, EPI_ISL_2879951, EPI_ISL_2879952, EPI_ISL_2879953, EPI_ISL_2879954, EPI_ISL_2879955, EPI_ISL_2879956, EPI_ISL_2879957, EPI_ISL_2879958, EPI_ISL_2879959, EPI_ISL_2879960, EPI_ISL_2879961, EPI_ISL_2879962,
EPI_ISL_2879963, EPI_ISL_2879964, EPI_ISL_2879965, EPI_ISL_2879966, EPI_ISL_2879967, EPI_ISL_2879968, EPI_ISL_2879969, EPI_ISL_2879970, EPI_ISL_2879971, EPI_ISL_2879972, EPI_ISL_2879973, EPI_ISL_2879974, EPI_ISL_2879975, EPI_ISL_2879976, EPI_ISL_2879977, EPI_ISL_2879978, EPI_ISL_2879979, EPI_ISL_2879980, EPI_ISL_2879981, EPI_ISL_2879982, EPI_ISL_2879983, EPI_ISL_2879984,
EPI_ISL_2879985, EPI_ISL_2879986, EPI_ISL_2879987, EPI_ISL_2879988, EPI_ISL_2879989, EPI_ISL_2879990, EPI_ISL_2879991, EPI_ISL_2879992, EPI_ISL_2879993, EPI_ISL_2879994, EPI_ISL_2879995, EPI_ISL_2879996, EPI_ISL_2879997, EPI_ISL_2879998, EPI_ISL_2879999, EPI_ISL_2880000, EPI_ISL_2880001, EPI_ISL_2880002, EPI_ISL_2880003, EPI_ISL_2880004, EPI_ISL_2880005, EPI_ISL_2880006,
EPI_ISL_2880007, EPI_ISL_2880008, EPI_ISL_2880009, EPI_ISL_2880010, EPI_ISL_2880011, EPI_ISL_2880012, EPI_ISL_2880013, EPI_ISL_2880014, EPI_ISL_2880015, EPI_ISL_2880016, EPI_ISL_2880017, EPI_ISL_2880018, EPI_ISL_2880019, EPI_ISL_2880020, EPI_ISL_2880021, EPI_ISL_2880022, EPI_ISL_2880023, EPI_ISL_2880024, EPI_ISL_2880025, EPI_ISL_2880026, EPI_ISL_2880027, EPI_ISL_2880028,
EPI_ISL_2880029, EPI_ISL_2880030, EPI_ISL_2880031, EPI_ISL_2880032, EPI_ISL_2880033, EPI_ISL_2880034, EPI_ISL_2880035, EPI_ISL_2880036, EPI_ISL_2880037, EPI_ISL_2880038, EPI_ISL_2880039, EPI_ISL_2880040, EPI_ISL_2880041, EPI_ISL_2880042, EPI_ISL_2880043, EPI_ISL_2880044, EPI_ISL_2880045, EPI_ISL_2880046, EPI_ISL_2880047, EPI_ISL_2880048, EPI_ISL_2880049, EPI_ISL_2880050,
EPI_ISL_2880051, EPI_ISL_2880052, EPI_ISL_2880053, EPI_ISL_2880054, EPI_ISL_2880055, EPI_ISL_2880056, EPI_ISL_2880057, EPI_ISL_2880058, EPI_ISL_2880059, EPI_ISL_2880060, EPI_ISL_2880061, EPI_ISL_2880062, EPI_ISL_2880063, EPI_ISL_2880064, EPI_ISL_2880065, EPI_ISL_2880066, EPI_ISL_2880067, EPI_ISL_2880068, EPI_ISL_2880069, EPI_ISL_2880070, EPI_ISL_2880071, EPI_ISL_2880072,
EPI_ISL_2880073, EPI_ISL_2880074, EPI_ISL_2880075, EPI_ISL_2880076, EPI_ISL_2880077, EPI_ISL_2880078, EPI_ISL_2880079, EPI_ISL_2880080, EPI_ISL_2880081, EPI_ISL_2880082, EPI_ISL_2880083, EPI_ISL_2880084, EPI_ISL_2880085, EPI_ISL_2880086, EPI_ISL_2880087, EPI_ISL_2880088, EPI_ISL_2880089, EPI_ISL_2880090, EPI_ISL_2880091, EPI_ISL_2880092, EPI_ISL_2880093, EPI_ISL_2880094,
EPI_ISL_2880095, EPI_ISL_2880096, EPI_ISL_2880097, EPI_ISL_2880098, EPI_ISL_2880099, EPI_ISL_2880100, EPI_ISL_2880101, EPI_ISL_2880102, EPI_ISL_2880103, EPI_ISL_2880104, EPI_ISL_2880105, EPI_ISL_2880106, EPI_ISL_2880107, EPI_ISL_2880108, EPI_ISL_2880109, EPI_ISL_2880110, EPI_ISL_2880111, EPI_ISL_2880112, EPI_ISL_2880147, EPI_ISL_2880148, EPI_ISL_2880149, EPI_ISL_2880150,
EPI_ISL_2880151, EPI_ISL_2880152, EPI_ISL_2880153, EPI_ISL_2880154, EPI_ISL_2880155, EPI_ISL_2880156, EPI_ISL_2880157, EPI_ISL_2880158, EPI_ISL_2880159, EPI_ISL_2880160, EPI_ISL_2880161, EPI_ISL_2880162, EPI_ISL_2880163, EPI_ISL_2880164, EPI_ISL_2880165, EPI_ISL_2880166, EPI_ISL_2880167, EPI_ISL_2880168, EPI_ISL_2880169, EPI_ISL_2880170, EPI_ISL_2880171, EPI_ISL_2880172,
EPI_ISL_2880173, EPI_ISL_2880174, EPI_ISL_2880175, EPI_ISL_2880176, EPI_ISL_2880177, EPI_ISL_2880178, EPI_ISL_2880179, EPI_ISL_2880180, EPI_ISL_2880181, EPI_ISL_2880182, EPI_ISL_2880183, EPI_ISL_2880184, EPI_ISL_2880185, EPI_ISL_2880186, EPI_ISL_2880187, EPI_ISL_2880188, EPI_ISL_2880189, EPI_ISL_2880190, EPI_ISL_2880191, EPI_ISL_2880192, EPI_ISL_2880193, EPI_ISL_2880194,
EPI_ISL_2880195, EPI_ISL_2880196, EPI_ISL_2880197, EPI_ISL_2880198, EPI_ISL_2880199, EPI_ISL_2880200, EPI_ISL_2880201, EPI_ISL_2880202, EPI_ISL_2880203, EPI_ISL_2880204, EPI_ISL_2880205, EPI_ISL_2880206, EPI_ISL_2880215, EPI_ISL_2880216, EPI_ISL_2880217, EPI_ISL_2880218, EPI_ISL_2880219, EPI_ISL_2880220, EPI_ISL_2880221, EPI_ISL_2880222, EPI_ISL_2880223, EPI_ISL_2880224,
EPI_ISL_2880225, EPI_ISL_2880226, EPI_ISL_2880227, EPI_ISL_2880228, EPI_ISL_2880229, EPI_ISL_2880230, EPI_ISL_2880231, EPI_ISL_2880232, EPI_ISL_2880233, EPI_ISL_2880234, EPI_ISL_2880235, EPI_ISL_2880236, EPI_ISL_2880237, EPI_ISL_2880238, EPI_ISL_2880239, EPI_ISL_2880240, EPI_ISL_2880241, EPI_ISL_2880242, EPI_ISL_2880243, EPI_ISL_2880244, EPI_ISL_2880245, EPI_ISL_2880246,
EPI_ISL_2880247, EPI_ISL_2880248, EPI_ISL_2880249, EPI_ISL_2880250, EPI_ISL_2880251, EPI_ISL_2880252, EPI_ISL_2880253, EPI_ISL_2880254, EPI_ISL_2880255, EPI_ISL_2880256, EPI_ISL_2880257, EPI_ISL_2880258, EPI_ISL_2880259, EPI_ISL_2880260, EPI_ISL_2880261, EPI_ISL_2880262, EPI_ISL_2880263, EPI_ISL_2880264, EPI_ISL_2880265, EPI_ISL_2880266, EPI_ISL_2880267, EPI_ISL_2880268,
EPI_ISL_2880269, EPI_ISL_2880270, EPI_ISL_2880271, EPI_ISL_2880272, EPI_ISL_2880273, EPI_ISL_2880274, EPI_ISL_2880275, EPI_ISL_2880276, EPI_ISL_2880277, EPI_ISL_2880278, EPI_ISL_2880279, EPI_ISL_2880280, EPI_ISL_2880281, EPI_ISL_2880282, EPI_ISL_2880283, EPI_ISL_2880284, EPI_ISL_2880285, EPI_ISL_2880286, EPI_ISL_2880287, EPI_ISL_2880288, EPI_ISL_2880289, EPI_ISL_2880290,
EPI_ISL_2880291, EPI_ISL_2880292, EPI_ISL_2880293, EPI_ISL_2880294, EPI_ISL_2880295, EPI_ISL_2880296, EPI_ISL_2880297, EPI_ISL_2880298, EPI_ISL_2880299, EPI_ISL_2880300, EPI_ISL_2880301, EPI_ISL_2880302, EPI_ISL_2880303, EPI_ISL_2880304, EPI_ISL_2880305, EPI_ISL_2880306, EPI_ISL_2880307, EPI_ISL_2880308, EPI_ISL_2880309, EPI_ISL_2880310, EPI_ISL_2880311, EPI_ISL_2880312,
EPI_ISL_2880313, EPI_ISL_2880314, EPI_ISL_2880315, EPI_ISL_2880316, EPI_ISL_2880317, EPI_ISL_2880318, EPI_ISL_2880319, EPI_ISL_2880320, EPI_ISL_2880321, EPI_ISL_2880322, EPI_ISL_2880323, EPI_ISL_2880324, EPI_ISL_2880325, EPI_ISL_2880326, EPI_ISL_2880327, EPI_ISL_2880328, EPI_ISL_2880329, EPI_ISL_2880330, EPI_ISL_2880331, EPI_ISL_2880332, EPI_ISL_2880333, EPI_ISL_2880334,
EPI_ISL_2880335, EPI_ISL_2880336, EPI_ISL_2880337, EPI_ISL_2880338, EPI_ISL_2880339, EPI_ISL_2880340, EPI_ISL_2880341, EPI_ISL_2880342, EPI_ISL_2880343, EPI_ISL_2880344, EPI_ISL_2880345, EPI_ISL_2880346, EPI_ISL_2880347, EPI_ISL_2880348, EPI_ISL_2880349, EPI_ISL_2880350, EPI_ISL_2880351, EPI_ISL_2880352, EPI_ISL_2880353, EPI_ISL_2880354, EPI_ISL_2880355, EPI_ISL_2880358,
EPI_ISL_2880359, EPI_ISL_2880360, EPI_ISL_2880361, EPI_ISL_2880362, EPI_ISL_2880363, EPI_ISL_2880364, EPI_ISL_2880365, EPI_ISL_2880366, EPI_ISL_2880367, EPI_ISL_2880368, EPI_ISL_2880369, EPI_ISL_2880370, EPI_ISL_2880371, EPI_ISL_2880372, EPI_ISL_2880373, EPI_ISL_2880374, EPI_ISL_2880375, EPI_ISL_2880376, EPI_ISL_2880377, EPI_ISL_2880378, EPI_ISL_2880379, EPI_ISL_2880380,
EPI_ISL_2880381, EPI_ISL_2880382, EPI_ISL_2880383, EPI_ISL_2880384, EPI_ISL_2880385, EPI_ISL_2880386, EPI_ISL_2880387, EPI_ISL_2880388, EPI_ISL_2880389, EPI_ISL_2880390, EPI_ISL_2880391, EPI_ISL_2880392, EPI_ISL_2880393, EPI_ISL_2880394, EPI_ISL_2880395, EPI_ISL_2880396, EPI_ISL_2880397, EPI_ISL_2880398, EPI_ISL_2880399, EPI_ISL_2880400, EPI_ISL_2880401, EPI_ISL_2880402,
EPI_ISL_2880403, EPI_ISL_2880404, EPI_ISL_2880405, EPI_ISL_2880406, EPI_ISL_2880407, EPI_ISL_2880408, EPI_ISL_2880409, EPI_ISL_2880410, EPI_ISL_2880411, EPI_ISL_2880412, EPI_ISL_2880413, EPI_ISL_2880414, EPI_ISL_2880415, EPI_ISL_2880416, EPI_ISL_2880417, EPI_ISL_2880418, EPI_ISL_2880419, EPI_ISL_2880420, EPI_ISL_2880421, EPI_ISL_2880422, EPI_ISL_2880423, EPI_ISL_2880424,
EPI_ISL_2880425, EPI_ISL_2880426, EPI_ISL_2880427, EPI_ISL_2880428, EPI_ISL_2880429, EPI_ISL_2880430, EPI_ISL_2880431, EPI_ISL_2880432, EPI_ISL_2880433, EPI_ISL_2880434, EPI_ISL_2880435, EPI_ISL_2880436, EPI_ISL_2880437, EPI_ISL_2880438, EPI_ISL_2880439, EPI_ISL_2880440, EPI_ISL_2880441, EPI_ISL_2880442, EPI_ISL_2880443, EPI_ISL_2880444, EPI_ISL_2880445, EPI_ISL_2880446,
EPI_ISL_2880448, EPI_ISL_2880449, EPI_ISL_2880498, EPI_ISL_2880499, EPI_ISL_2880500, EPI_ISL_2880501, EPI_ISL_2880502, EPI_ISL_2880503, EPI_ISL_2880504, EPI_ISL_2880505, EPI_ISL_2880506, EPI_ISL_2880507, EPI_ISL_2880508, EPI_ISL_2880509, EPI_ISL_2880510, EPI_ISL_2880511, EPI_ISL_2880512, EPI_ISL_2880513, EPI_ISL_2880514, EPI_ISL_2880515, EPI_ISL_2880516, EPI_ISL_2880517,
EPI_ISL_2880518, EPI_ISL_2880519, EPI_ISL_2880520, EPI_ISL_2880521, EPI_ISL_2880522, EPI_ISL_2880523, EPI_ISL_2880524, EPI_ISL_2880525, EPI_ISL_2880526, EPI_ISL_2880527, EPI_ISL_2880528, EPI_ISL_2880529, EPI_ISL_2880530, EPI_ISL_2880531, EPI_ISL_2880532, EPI_ISL_2880533, EPI_ISL_2880534, EPI_ISL_2880535, EPI_ISL_2880536, EPI_ISL_2880537, EPI_ISL_2880538, EPI_ISL_2880539,
EPI_ISL_2880540, EPI_ISL_2880541, EPI_ISL_2880542, EPI_ISL_2880543, EPI_ISL_2880544, EPI_ISL_2880545, EPI_ISL_2880546, EPI_ISL_2880547, EPI_ISL_2880548, EPI_ISL_2880549, EPI_ISL_2880550, EPI_ISL_2880551, EPI_ISL_2880552, EPI_ISL_2880553, EPI_ISL_2880554, EPI_ISL_2880555, EPI_ISL_2880556, EPI_ISL_2880557, EPI_ISL_2880558, EPI_ISL_2880559, EPI_ISL_2880560, EPI_ISL_2880561,
EPI_ISL_2880562, EPI_ISL_2880563, EPI_ISL_2880564, EPI_ISL_2880565, EPI_ISL_2880566, EPI_ISL_2880567, EPI_ISL_2880568, EPI_ISL_2880569, EPI_ISL_2880570, EPI_ISL_2880571, EPI_ISL_2880572, EPI_ISL_2880573, EPI_ISL_2880574, EPI_ISL_2880575, EPI_ISL_2880576, EPI_ISL_2880577, EPI_ISL_2880578, EPI_ISL_2880579, EPI_ISL_2880580, EPI_ISL_2880581, EPI_ISL_2880582, EPI_ISL_2880583,
EPI_ISL_2880584, EPI_ISL_2880585, EPI_ISL_2880586, EPI_ISL_2880587, EPI_ISL_2880588, EPI_ISL_2880589, EPI_ISL_2880590, EPI_ISL_2880591, EPI_ISL_2880592, EPI_ISL_2880593, EPI_ISL_2880682, EPI_ISL_2880683, EPI_ISL_2880684, EPI_ISL_2880685, EPI_ISL_2880700, EPI_ISL_2880701, EPI_ISL_2880702, EPI_ISL_2880703, EPI_ISL_2880704, EPI_ISL_2880705, EPI_ISL_2880706, EPI_ISL_2880707,
EPI_ISL_2880708, EPI_ISL_2880709, EPI_ISL_2880710, EPI_ISL_2880711, EPI_ISL_2880712, EPI_ISL_2880713, EPI_ISL_2880714, EPI_ISL_2880715, EPI_ISL_2880716, EPI_ISL_2880717, EPI_ISL_2880718, EPI_ISL_2880719, EPI_ISL_2880720, EPI_ISL_2880721, EPI_ISL_2880722, EPI_ISL_2880723, EPI_ISL_2880724, EPI_ISL_2880725, EPI_ISL_2880726, EPI_ISL_2880727, EPI_ISL_2880728, EPI_ISL_2880729,
EPI_ISL_2880730, EPI_ISL_2880731, EPI_ISL_2880732, EPI_ISL_2880733, EPI_ISL_2880734, EPI_ISL_2880735, EPI_ISL_2880736, EPI_ISL_2880737, EPI_ISL_2880738, EPI_ISL_2880739, EPI_ISL_2880740, EPI_ISL_2880741, EPI_ISL_2880742, EPI_ISL_2880743, EPI_ISL_2880744, EPI_ISL_2880745, EPI_ISL_2880746, EPI_ISL_2880747, EPI_ISL_2880748, EPI_ISL_2880749, EPI_ISL_2880750, EPI_ISL_2880751,
EPI_ISL_2880752, EPI_ISL_2880753, EPI_ISL_2880754, EPI_ISL_2880755, EPI_ISL_2880756, EPI_ISL_2880757, EPI_ISL_2880758, EPI_ISL_2880759, EPI_ISL_2880760, EPI_ISL_2880761, EPI_ISL_2880762, EPI_ISL_2880763, EPI_ISL_2880764, EPI_ISL_2880765, EPI_ISL_2880766, EPI_ISL_2880767, EPI_ISL_2880768, EPI_ISL_2880769, EPI_ISL_2880770, EPI_ISL_2880771, EPI_ISL_2880772, EPI_ISL_2880773,
EPI_ISL_2880774, EPI_ISL_2880775, EPI_ISL_2880776, EPI_ISL_2880777, EPI_ISL_2880778, EPI_ISL_2880779, EPI_ISL_2880780, EPI_ISL_2880781, EPI_ISL_2880782, EPI_ISL_2880783, EPI_ISL_2880784, EPI_ISL_2880785, EPI_ISL_2880786, EPI_ISL_2880787, EPI_ISL_2880788, EPI_ISL_2880789, EPI_ISL_2880790, EPI_ISL_2880791, EPI_ISL_2880792, EPI_ISL_2880793, EPI_ISL_2880794, EPI_ISL_2880795,
EPI_ISL_2880796, EPI_ISL_2880797, EPI_ISL_2880798, EPI_ISL_2880799, EPI_ISL_2880800, EPI_ISL_2880801, EPI_ISL_2880802, EPI_ISL_2880803, EPI_ISL_2880804, EPI_ISL_2880805, EPI_ISL_2880806, EPI_ISL_2880807, EPI_ISL_2880808, EPI_ISL_2880809, EPI_ISL_2880810, EPI_ISL_2880811, EPI_ISL_2880812, EPI_ISL_2880813, EPI_ISL_2880814, EPI_ISL_2880815, EPI_ISL_2880816, EPI_ISL_2880817,
EPI_ISL_2880818, EPI_ISL_2880819, EPI_ISL_2880820, EPI_ISL_2880821, EPI_ISL_2880822, EPI_ISL_2880823, EPI_ISL_2880824, EPI_ISL_2880825, EPI_ISL_2880826, EPI_ISL_2880827, EPI_ISL_2880828, EPI_ISL_2880829, EPI_ISL_2880830, EPI_ISL_2880831, EPI_ISL_2880832, EPI_ISL_2880833, EPI_ISL_2880834, EPI_ISL_2880835, EPI_ISL_2880836, EPI_ISL_2880837, EPI_ISL_2880838, EPI_ISL_2880839,
EPI_ISL_2880840, EPI_ISL_2880841, EPI_ISL_2880842, EPI_ISL_2880843, EPI_ISL_2880844, EPI_ISL_2880845, EPI_ISL_2880846, EPI_ISL_2880847, EPI_ISL_2880848, EPI_ISL_2880849, EPI_ISL_2880850, EPI_ISL_2880851, EPI_ISL_2880852, EPI_ISL_2880853, EPI_ISL_2880854, EPI_ISL_2880855, EPI_ISL_2880856, EPI_ISL_2880857, EPI_ISL_2880858, EPI_ISL_2880859, EPI_ISL_2880860, EPI_ISL_2880861,
EPI_ISL_2880862, EPI_ISL_2880863, EPI_ISL_2880864, EPI_ISL_2880865, EPI_ISL_2880866, EPI_ISL_2880867, EPI_ISL_2880868, EPI_ISL_2880869, EPI_ISL_2880870, EPI_ISL_2880871, EPI_ISL_2880872, EPI_ISL_2880873, EPI_ISL_2880874, EPI_ISL_2880875, EPI_ISL_2880876, EPI_ISL_2880877, EPI_ISL_2880878, EPI_ISL_2880879, EPI_ISL_2880880, EPI_ISL_2880881, EPI_ISL_2880882, EPI_ISL_2880883,
EPI_ISL_2880884, EPI_ISL_2880885, EPI_ISL_2880886, EPI_ISL_2880887, EPI_ISL_2880888, EPI_ISL_2880889, EPI_ISL_2880890, EPI_ISL_2880891, EPI_ISL_2880892, EPI_ISL_2880893, EPI_ISL_2880894, EPI_ISL_2880895, EPI_ISL_2880897, EPI_ISL_2880898, EPI_ISL_2880899, EPI_ISL_2880900, EPI_ISL_2880901, EPI_ISL_2880902, EPI_ISL_2880903, EPI_ISL_2880904, EPI_ISL_2880905, EPI_ISL_2880906,
EPI_ISL_2880907, EPI_ISL_2880908, EPI_ISL_2880909, EPI_ISL_2880910, EPI_ISL_2880918, EPI_ISL_2880919, EPI_ISL_2880920, EPI_ISL_2880921, EPI_ISL_2880922, EPI_ISL_2880923, EPI_ISL_2880924, EPI_ISL_2880925, EPI_ISL_2880926, EPI_ISL_2880927, EPI_ISL_2880928, EPI_ISL_2880929, EPI_ISL_2880930, EPI_ISL_2880931, EPI_ISL_2880932, EPI_ISL_2880933, EPI_ISL_2880934, EPI_ISL_2881018,
EPI_ISL_2881019, EPI_ISL_2881020, EPI_ISL_2881021, EPI_ISL_2881022, EPI_ISL_2881023, EPI_ISL_2881024, EPI_ISL_2881025, EPI_ISL_2881026, EPI_ISL_2881027, EPI_ISL_2881028, EPI_ISL_2881029, EPI_ISL_2881030, EPI_ISL_2881031, EPI_ISL_2881032, EPI_ISL_2881033, EPI_ISL_2881034, EPI_ISL_2881035, EPI_ISL_2881036, EPI_ISL_2881037, EPI_ISL_2881038, EPI_ISL_2881039, EPI_ISL_2881040,
EPI_ISL_2881041, EPI_ISL_2881042, EPI_ISL_2881043, EPI_ISL_2881044, EPI_ISL_2881045, EPI_ISL_2881046, EPI_ISL_2881047, EPI_ISL_2881048, EPI_ISL_2881049, EPI_ISL_2881050, EPI_ISL_2881051, EPI_ISL_2881052, EPI_ISL_2881053, EPI_ISL_2881054, EPI_ISL_2881055, EPI_ISL_2881056, EPI_ISL_2881057, EPI_ISL_2881058, EPI_ISL_2881059, EPI_ISL_2881060, EPI_ISL_2881061, EPI_ISL_2881062,
EPI_ISL_2881063, EPI_ISL_2881064, EPI_ISL_2881065, EPI_ISL_2881066, EPI_ISL_2881067, EPI_ISL_2881068, EPI_ISL_2881069, EPI_ISL_2881070, EPI_ISL_2881071, EPI_ISL_2881072, EPI_ISL_2881073, EPI_ISL_2881074, EPI_ISL_2881075, EPI_ISL_2881076, EPI_ISL_2881077, EPI_ISL_2881078, EPI_ISL_2881079, EPI_ISL_2881080, EPI_ISL_2881081, EPI_ISL_2881082, EPI_ISL_2881083, EPI_ISL_2881084,
EPI_ISL_2881085, EPI_ISL_2881086, EPI_ISL_2881087, EPI_ISL_2881088, EPI_ISL_2881089, EPI_ISL_2881090, EPI_ISL_2881091, EPI_ISL_2881092, EPI_ISL_2881093, EPI_ISL_2881094, EPI_ISL_2881095, EPI_ISL_2881096, EPI_ISL_2881097, EPI_ISL_2881098, EPI_ISL_2881099, EPI_ISL_2881100, EPI_ISL_2881101, EPI_ISL_2881102, EPI_ISL_2881103, EPI_ISL_2881104, EPI_ISL_2881105, EPI_ISL_2881106,
EPI_ISL_2881107, EPI_ISL_2881108, EPI_ISL_2881109, EPI_ISL_2881110, EPI_ISL_2881111, EPI_ISL_2881112, EPI_ISL_2881113, EPI_ISL_2881114, EPI_ISL_2881115, EPI_ISL_2881116, EPI_ISL_2881117, EPI_ISL_2881118, EPI_ISL_2881119, EPI_ISL_2881120, EPI_ISL_2881121, EPI_ISL_2881122, EPI_ISL_2881123, EPI_ISL_2881124, EPI_ISL_2881125, EPI_ISL_2881126, EPI_ISL_2881127, EPI_ISL_2881128,
EPI_ISL_2881129, EPI_ISL_2881130, EPI_ISL_2881131, EPI_ISL_2881132, EPI_ISL_2881133, EPI_ISL_2881134, EPI_ISL_2881135, EPI_ISL_2881136, EPI_ISL_2881137, EPI_ISL_2881138, EPI_ISL_2881139, EPI_ISL_2881140, EPI_ISL_2881141, EPI_ISL_2881142, EPI_ISL_2881143, EPI_ISL_2881144, EPI_ISL_2881145, EPI_ISL_2881146, EPI_ISL_2881147, EPI_ISL_2881148, EPI_ISL_2881149, EPI_ISL_2881150,
EPI_ISL_2881151, EPI_ISL_2881152, EPI_ISL_2881153, EPI_ISL_2881154, EPI_ISL_2881155, EPI_ISL_2881156, EPI_ISL_2881157, EPI_ISL_2881158, EPI_ISL_2881159, EPI_ISL_2881160, EPI_ISL_2881161, EPI_ISL_2881162, EPI_ISL_2881163, EPI_ISL_2881164, EPI_ISL_2881165, EPI_ISL_2881166, EPI_ISL_2881167, EPI_ISL_2881168, EPI_ISL_2881169, EPI_ISL_2881170, EPI_ISL_2881171, EPI_ISL_2881172,
EPI_ISL_2881173, EPI_ISL_2881174, EPI_ISL_2881175, EPI_ISL_2881176, EPI_ISL_2881177, EPI_ISL_2881178, EPI_ISL_2881179, EPI_ISL_2881180, EPI_ISL_2881181, EPI_ISL_2881182, EPI_ISL_2881183, EPI_ISL_2881184, EPI_ISL_2881185, EPI_ISL_2881186, EPI_ISL_2881187, EPI_ISL_2881188, EPI_ISL_2881189, EPI_ISL_2881190, EPI_ISL_2881191, EPI_ISL_2881192, EPI_ISL_2881193, EPI_ISL_2881194,
EPI_ISL_2881195, EPI_ISL_2881196, EPI_ISL_2881197, EPI_ISL_2881198, EPI_ISL_2881199, EPI_ISL_2881200, EPI_ISL_2881201, EPI_ISL_2881202, EPI_ISL_2881203, EPI_ISL_2881204, EPI_ISL_2881205, EPI_ISL_2881206, EPI_ISL_2881207, EPI_ISL_2881208, EPI_ISL_2881209, EPI_ISL_2881210, EPI_ISL_2881211, EPI_ISL_2881212, EPI_ISL_2881213, EPI_ISL_2881214, EPI_ISL_2881215, EPI_ISL_2881216,
EPI_ISL_2881217, EPI_ISL_2881218, EPI_ISL_2881219, EPI_ISL_2881220, EPI_ISL_2881221, EPI_ISL_2881222, EPI_ISL_2881223, EPI_ISL_2881224, EPI_ISL_2881225, EPI_ISL_2881226, EPI_ISL_2881227, EPI_ISL_2881228, EPI_ISL_2881229, EPI_ISL_2881230, EPI_ISL_2881231, EPI_ISL_2881232, EPI_ISL_2881233, EPI_ISL_2881234, EPI_ISL_2881235, EPI_ISL_2881236, EPI_ISL_2881237, EPI_ISL_2881238,
EPI_ISL_2881239, EPI_ISL_2881240, EPI_ISL_2881241, EPI_ISL_2881242, EPI_ISL_2881243, EPI_ISL_2881244, EPI_ISL_2881245, EPI_ISL_2881246, EPI_ISL_2881247, EPI_ISL_2881248, EPI_ISL_2881249, EPI_ISL_2881250, EPI_ISL_2881324, EPI_ISL_2881325, EPI_ISL_2881326, EPI_ISL_2881327, EPI_ISL_2881328, EPI_ISL_2881329, EPI_ISL_2881330, EPI_ISL_2881331, EPI_ISL_2881332, EPI_ISL_2881333,
EPI_ISL_2881334, EPI_ISL_2881335, EPI_ISL_2881336, EPI_ISL_2881337, EPI_ISL_2881338, EPI_ISL_2881339, EPI_ISL_2881340, EPI_ISL_2881341, EPI_ISL_2881342, EPI_ISL_2881343, EPI_ISL_2881344, EPI_ISL_2881345, EPI_ISL_2881346, EPI_ISL_2881347, EPI_ISL_2881348, EPI_ISL_2881349, EPI_ISL_2881350, EPI_ISL_2881351, EPI_ISL_2881352, EPI_ISL_2881353, EPI_ISL_2881354, EPI_ISL_2881355,
EPI_ISL_2881356, EPI_ISL_2881357, EPI_ISL_2881358, EPI_ISL_2881359, EPI_ISL_2881360, EPI_ISL_2881361, EPI_ISL_2881362, EPI_ISL_2881363, EPI_ISL_2881364, EPI_ISL_2881365, EPI_ISL_2881366, EPI_ISL_2881367, EPI_ISL_2881368, EPI_ISL_2881369, EPI_ISL_2881370, EPI_ISL_2881371, EPI_ISL_2881372, EPI_ISL_2881373, EPI_ISL_2881374, EPI_ISL_2881433, EPI_ISL_2881434, EPI_ISL_2881435,
EPI_ISL_2881436, EPI_ISL_2881437, EPI_ISL_2881438, EPI_ISL_2881439, EPI_ISL_2881440, EPI_ISL_2881441, EPI_ISL_2881442, EPI_ISL_2881443, EPI_ISL_2881444, EPI_ISL_2881445, EPI_ISL_2881446, EPI_ISL_2881447, EPI_ISL_2881448, EPI_ISL_2881449, EPI_ISL_2881450, EPI_ISL_2881451, EPI_ISL_2881452, EPI_ISL_2881453, EPI_ISL_2881454, EPI_ISL_2881455, EPI_ISL_2881456, EPI_ISL_2881457,
EPI_ISL_2881458, EPI_ISL_2881459, EPI_ISL_2881460, EPI_ISL_2881461, EPI_ISL_2881462, EPI_ISL_2881463, EPI_ISL_2881464, EPI_ISL_2881465, EPI_ISL_2881466, EPI_ISL_2881467, EPI_ISL_2881468, EPI_ISL_2881469, EPI_ISL_2881470, EPI_ISL_2881471, EPI_ISL_2881472, EPI_ISL_2881473, EPI_ISL_2881474, EPI_ISL_2881475, EPI_ISL_2881476, EPI_ISL_2881477, EPI_ISL_2881478, EPI_ISL_2881479,
EPI_ISL_2881480, EPI_ISL_2881481, EPI_ISL_2881482, EPI_ISL_2881483, EPI_ISL_2881484, EPI_ISL_2881485, EPI_ISL_2881486, EPI_ISL_2881487, EPI_ISL_2881488, EPI_ISL_2881489, EPI_ISL_2881490, EPI_ISL_2881491, EPI_ISL_2881492, EPI_ISL_2881493, EPI_ISL_2881494, EPI_ISL_2881495, EPI_ISL_2881496, EPI_ISL_2881497, EPI_ISL_2881498, EPI_ISL_2881499, EPI_ISL_2881500, EPI_ISL_2881501,
EPI_ISL_2881502, EPI_ISL_2881503, EPI_ISL_2881504, EPI_ISL_2881505, EPI_ISL_2881506, EPI_ISL_2881507, EPI_ISL_2881508, EPI_ISL_2881509, EPI_ISL_2881510, EPI_ISL_2881511, EPI_ISL_2881512, EPI_ISL_2881513, EPI_ISL_2881514, EPI_ISL_2881515, EPI_ISL_2881516, EPI_ISL_2881517, EPI_ISL_2881518, EPI_ISL_2881519, EPI_ISL_2881520, EPI_ISL_2881521, EPI_ISL_2881522, EPI_ISL_2881523,
EPI_ISL_2881524, EPI_ISL_2881525, EPI_ISL_2881526, EPI_ISL_2881527, EPI_ISL_2881528, EPI_ISL_2881529, EPI_ISL_2881530, EPI_ISL_2881531, EPI_ISL_2881532, EPI_ISL_2881533, EPI_ISL_2881534, EPI_ISL_2881535, EPI_ISL_2881536, EPI_ISL_2881537, EPI_ISL_2881538, EPI_ISL_2881539, EPI_ISL_2881540, EPI_ISL_2881541, EPI_ISL_2881542, EPI_ISL_2881543, EPI_ISL_2881544, EPI_ISL_2881545,
EPI_ISL_2881546, EPI_ISL_2881547, EPI_ISL_2881548, EPI_ISL_2881549, EPI_ISL_2881550, EPI_ISL_2881551, EPI_ISL_2881552, EPI_ISL_2881553, EPI_ISL_2881554, EPI_ISL_2881555, EPI_ISL_2881556, EPI_ISL_2881557, EPI_ISL_2881558, EPI_ISL_2881559, EPI_ISL_2881560, EPI_ISL_2881561, EPI_ISL_2881562, EPI_ISL_2881563, EPI_ISL_2881564, EPI_ISL_2881565, EPI_ISL_2881566, EPI_ISL_2881567,
EPI_ISL_2881568, EPI_ISL_2881569, EPI_ISL_2881570, EPI_ISL_2881571, EPI_ISL_2881572, EPI_ISL_2881573, EPI_ISL_2881574, EPI_ISL_2881575, EPI_ISL_2881576, EPI_ISL_2881577, EPI_ISL_2881578, EPI_ISL_2881579, EPI_ISL_2881580, EPI_ISL_2881581, EPI_ISL_2881582, EPI_ISL_2881583, EPI_ISL_2881584, EPI_ISL_2881585, EPI_ISL_2881586, EPI_ISL_2881587, EPI_ISL_2881588, EPI_ISL_2881589,
EPI_ISL_2881590, EPI_ISL_2881591, EPI_ISL_2881592, EPI_ISL_2881593, EPI_ISL_2881594, EPI_ISL_2881595, EPI_ISL_2881596, EPI_ISL_2881597, EPI_ISL_2881598, EPI_ISL_2881599, EPI_ISL_2881600, EPI_ISL_2881601, EPI_ISL_2881602, EPI_ISL_2881603, EPI_ISL_2881604, EPI_ISL_2881605, EPI_ISL_2881606, EPI_ISL_2881607, EPI_ISL_2881608, EPI_ISL_2881609, EPI_ISL_2881610, EPI_ISL_2881611,
EPI_ISL_2881612, EPI_ISL_2881613, EPI_ISL_2881614, EPI_ISL_2881615, EPI_ISL_2881616, EPI_ISL_2881617, EPI_ISL_2881618, EPI_ISL_2881619, EPI_ISL_2881620, EPI_ISL_2881621, EPI_ISL_2881622, EPI_ISL_2881623, EPI_ISL_2881624, EPI_ISL_2881625, EPI_ISL_2881626, EPI_ISL_2881627, EPI_ISL_2881628, EPI_ISL_2881629, EPI_ISL_2881630, EPI_ISL_2881631, EPI_ISL_2881632, EPI_ISL_2881633,
EPI_ISL_2881634, EPI_ISL_2881635, EPI_ISL_2881636, EPI_ISL_2881637, EPI_ISL_2881638, EPI_ISL_2881639, EPI_ISL_2881640, EPI_ISL_2881641, EPI_ISL_2881642, EPI_ISL_2881643, EPI_ISL_2881644, EPI_ISL_2881645, EPI_ISL_2881646, EPI_ISL_2881647, EPI_ISL_2881648, EPI_ISL_2881649, EPI_ISL_2881650, EPI_ISL_2881651, EPI_ISL_2881652, EPI_ISL_2881653, EPI_ISL_2881654, EPI_ISL_2881655,
EPI_ISL_2881656, EPI_ISL_2881657, EPI_ISL_2881658, EPI_ISL_2881659, EPI_ISL_2881660, EPI_ISL_2881661, EPI_ISL_2881662, EPI_ISL_2881663, EPI_ISL_2881664, EPI_ISL_2881665, EPI_ISL_2881666, EPI_ISL_2881667, EPI_ISL_2881668, EPI_ISL_2881669, EPI_ISL_2881670, EPI_ISL_2881671, EPI_ISL_2881672, EPI_ISL_2881673, EPI_ISL_2881674, EPI_ISL_2881675, EPI_ISL_2881676, EPI_ISL_2881677,
EPI_ISL_2881678, EPI_ISL_2881679, EPI_ISL_2881680, EPI_ISL_2881681, EPI_ISL_2881682, EPI_ISL_2881683, EPI_ISL_2881684, EPI_ISL_2881685, EPI_ISL_2881686, EPI_ISL_2881687, EPI_ISL_2881688, EPI_ISL_2881689, EPI_ISL_2881690, EPI_ISL_2881691, EPI_ISL_2881692, EPI_ISL_2881693, EPI_ISL_2881694, EPI_ISL_2881695, EPI_ISL_2881696, EPI_ISL_2881697, EPI_ISL_2881698, EPI_ISL_2881699,
EPI_ISL_2881700, EPI_ISL_2881701, EPI_ISL_2881702, EPI_ISL_2881703, EPI_ISL_2881704, EPI_ISL_2881705, EPI_ISL_2881706, EPI_ISL_2881707, EPI_ISL_2881708, EPI_ISL_2881709, EPI_ISL_2881710, EPI_ISL_2881711, EPI_ISL_2881712, EPI_ISL_2881713, EPI_ISL_2881714, EPI_ISL_2881715, EPI_ISL_2881716, EPI_ISL_2881717, EPI_ISL_2881718, EPI_ISL_2881719, EPI_ISL_2881720, EPI_ISL_3267189,
EPI_ISL_3267190, EPI_ISL_3267191, EPI_ISL_3267192, EPI_ISL_3267193, EPI_ISL_3267203, EPI_ISL_3267204, EPI_ISL_3267207, EPI_ISL_3267209, EPI_ISL_3267211, EPI_ISL_3267214, EPI_ISL_3267215, EPI_ISL_3267216, EPI_ISL_3267217, EPI_ISL_3267220, EPI_ISL_3267233, EPI_ISL_3267234, EPI_ISL_3267235, EPI_ISL_3267236, EPI_ISL_3267237, EPI_ISL_3267238, EPI_ISL_3267239, EPI_ISL_3267240,
EPI_ISL_3267241, EPI_ISL_3267242, EPI_ISL_3267243, EPI_ISL_3267244, EPI_ISL_3267245, EPI_ISL_3267246, EPI_ISL_3267247, EPI_ISL_3267248, EPI_ISL_3267249, EPI_ISL_3267250, EPI_ISL_3267251, EPI_ISL_3267252, EPI_ISL_3267257, EPI_ISL_3267264, EPI_ISL_3267266, EPI_ISL_3267273, EPI_ISL_3267277, EPI_ISL_3267278, EPI_ISL_3267279, EPI_ISL_3267280, EPI_ISL_3267281, EPI_ISL_3267282,
EPI_ISL_3267283, EPI_ISL_3267284, EPI_ISL_3267285, EPI_ISL_3267286, EPI_ISL_3267287, EPI_ISL_3267288, EPI_ISL_3267289, EPI_ISL_3267290, EPI_ISL_3267291, EPI_ISL_3267292, EPI_ISL_3267293, EPI_ISL_3267294, EPI_ISL_3267295, EPI_ISL_3267310, EPI_ISL_3267311, EPI_ISL_3267312, EPI_ISL_3267313, EPI_ISL_3267314, EPI_ISL_3267315, EPI_ISL_3267316, EPI_ISL_3267317, EPI_ISL_3267367,
EPI_ISL_3267371, EPI_ISL_3267372, EPI_ISL_3267373, EPI_ISL_3267374, EPI_ISL_3267375, EPI_ISL_3267376, EPI_ISL_3267377, EPI_ISL_3267389, EPI_ISL_3267390, EPI_ISL_3267391, EPI_ISL_3267392, EPI_ISL_3267393, EPI_ISL_3267394, EPI_ISL_3267395, EPI_ISL_3267396, EPI_ISL_3267397, EPI_ISL_3267398, EPI_ISL_3267399, EPI_ISL_3267400, EPI_ISL_3267401, EPI_ISL_3267402, EPI_ISL_3267403,
EPI_ISL_3267404, EPI_ISL_3267405, EPI_ISL_3267406, EPI_ISL_3267407, EPI_ISL_3267408, EPI_ISL_3267409, EPI_ISL_3267410

see above	INSACOG Surveillance	INSACOG at CSIR Institute of Genomics and Integrative Biology	INSACOG
EPI_ISL_2547436, EPI_ISL_2547451	INSACOG-MEGHALAYA	National Institute of Biomedical Genomics – INSACOG	Arindam Maitra; E Marak; E Shadap; Nidhan Kumar Biswas; R. Lyngdoh; Saumitra Das; Sreedhar Chinnaswamy
EPI_ISL_2521609	INSACOG-Mizoram	National Institute of Biomedical Genomics – INSACOG	Arindam Maitra; Gracy Laldinmawii; N Senthil Kumar; Nidhan Kumar Biswas; Saumitra Das; Sreedhar Chinnaswamy; Swagnik Roy
EPI_ISL_2503382, EPI_ISL_2548821	INSACOG-WB	National Institute of Biomedical Genomics – INSACOG	Ajay Chakraborti; Arindam Maitra; Bhaswati Bandyopadhyay; Nidhan Kumar Biswas; Saumitra Das; Sreedhar Chinnaswamy; Tamal Ghosh

EPI_ISL_3242393, EPI_ISL_3242394, EPI_ISL_3242395, EPI_ISL_3242396, EPI_ISL_3242397, EPI_ISL_3242398, EPI_ISL_3242399, EPI_ISL_3242400, EPI_ISL_3243197, EPI_ISL_3243198, EPI_ISL_3243199, EPI_ISL_3243200, EPI_ISL_3243201, EPI_ISL_3243202, EPI_ISL_3243203, EPI_ISL_3243204, EPI_ISL_3243205			
see above	Indian Institute of Science Education and Research	CDFD	Ashwin Dalal; Asmita Gupta; Divya Vashisht; Indranil Banerjee; Murali Bashyam; Reelina Basu; Vinay Donipadi
EPI_ISL_2955714, EPI_ISL_2955715, EPI_ISL_2955716, EPI_ISL_2955717, EPI_ISL_2955718, EPI_ISL_2955719, EPI_ISL_2955720, EPI_ISL_2955721, EPI_ISL_2955722, EPI_ISL_2955723, EPI_ISL_2955724, EPI_ISL_2955725, EPI_ISL_2955726, EPI_ISL_2955727, EPI_ISL_2955728, EPI_ISL_2955729, EPI_ISL_2955730, EPI_ISL_2955731, EPI_ISL_2955732, EPI_ISL_2955733, EPI_ISL_2955734, EPI_ISL_2955735, EPI_ISL_2955736, EPI_ISL_2955737, EPI_ISL_2955738, EPI_ISL_2955739, EPI_ISL_2955740, EPI_ISL_2955741, EPI_ISL_2955742, EPI_ISL_2955743, EPI_ISL_2955744, EPI_ISL_2955745, EPI_ISL_2955746, EPI_ISL_2955747, EPI_ISL_2955748, EPI_ISL_2955749, EPI_ISL_2955750, EPI_ISL_2955751, EPI_ISL_2955752, EPI_ISL_2955753, EPI_ISL_2955754, EPI_ISL_2955755, EPI_ISL_2955756, EPI_ISL_2955757, EPI_ISL_2955758, EPI_ISL_2955759, EPI_ISL_2955760, EPI_ISL_2955761, EPI_ISL_2955762, EPI_ISL_2955763, EPI_ISL_2955764, EPI_ISL_2955765, EPI_ISL_2955766, EPI_ISL_2955767, EPI_ISL_2955768, EPI_ISL_3066818, EPI_ISL_3066819, EPI_ISL_3066820, EPI_ISL_3066821, EPI_ISL_3066822, EPI_ISL_3066823, EPI_ISL_3066824, EPI_ISL_3066825, EPI_ISL_3066826, EPI_ISL_3066827, EPI_ISL_3066828, EPI_ISL_3066829, EPI_ISL_3066830, EPI_ISL_3066831, EPI_ISL_3066832, EPI_ISL_3066833, EPI_ISL_3066834, EPI_ISL_3066835, EPI_ISL_3066836, EPI_ISL_3066837			
see above	Indraprastha Apollo Hospital, New Delhi, India	NCDC Delhi, Biotechnology Division INSACOG	Hema Gogia; Hemlata Lall; Kalaiarasan Ponnusamy; Mahesh S Dhar; Manoj K Singh; Meena Datta; Partha Rakshit; Preeti Madan; Priyanka Singh; Radhakrishnan V. S; Robin Marwal; Sandhya Kabra; Sujeet K Singh; Uma Sharma
EPI_ISL_1844778, EPI_ISL_1844782	Maulana Azad Medical College	Biotechnology Division, NCDC	Hema Gogia; Hemlata Lall; Kalaiarasan Ponnusamy; Mahesh S Dhar; Manoj K Singh; Meena Datta; Partha Rakshit; Preeti Madan; Priyanka Singh; Radhakrishnan V. S; Robin Marwal; Sandhya Kabra; Sujeet K Singh; Uma Sharma
EPI_ISL_508415, EPI_ISL_508416, EPI_ISL_508417, EPI_ISL_508418, EPI_ISL_508420, EPI_ISL_508421, EPI_ISL_508422, EPI_ISL_653833, EPI_ISL_653834, EPI_ISL_653835, EPI_ISL_653836, EPI_ISL_653837, EPI_ISL_653838, EPI_ISL_653839, EPI_ISL_653840, EPI_ISL_653841, EPI_ISL_653842, EPI_ISL_653843, EPI_ISL_653844, EPI_ISL_653845, EPI_ISL_653846, EPI_ISL_653847, EPI_ISL_653848, EPI_ISL_653849, EPI_ISL_653850, EPI_ISL_653851, EPI_ISL_653852, EPI_ISL_653853, EPI_ISL_653854, EPI_ISL_653855, EPI_ISL_653856, EPI_ISL_653857, EPI_ISL_653858			
see above	Maulana Azad Medical College	National Institute of Biomedical Genomics	Arindam Maitra; Oves Siddiqui; Saumitra Das; Sonal Saxena; Vikas Manchanda
EPI_ISL_862051	Multidisciplinary Research Unit, DHR-ICMR, Institute of Medical Sciences, Banaras Hindu University	Multidisciplinary Research Unit, DHR-ICMR, Institute of Medical Sciences, Banaras Hindu University	Abhay Kumar Yadav; Arup Acharjee; Ashish; Chetan Sahni; Deepa Devadas; Jay Prakash Maurya; Manpreet Kaur; Nitish Kumar Singh; Prashant Singh; Priyoneel Basu; Richa Arya; Royana Singh; Sanjay Kumar; Surendra Pratap Mishra; Tribhuwan Mohan Mahapatra
EPI_ISL_3200822, EPI_ISL_3265676, EPI_ISL_3266066, EPI_ISL_3266068, EPI_ISL_3266069, EPI_ISL_3266070, EPI_ISL_3266071			
see above	Multidisciplinary Research Unit, Institute of Medical Sciences, Banaras Hindu University, Varanasi - 221005	Multidisciplinary Research Unit, Institute of Medical Sciences, Banaras Hindu University, Varanasi - 221005	Abhay Kumar Yadav; Ajay Kumar Yadav; Ashish Ashish; Chetan Sahni; Deepa Devadas; Gunjan Rai; Janhavi Yadav; Manpreet Kaur; Meghna Wadi; Nargis Khanam; Nitish Kumar Singh; Pranav Singh; Priyoneel Basu; Ragini Mishra; Royana Singh; Sangeeta Rai; Sarita Chowdhary; Saumya Singh; Saurabh Singh; Shani Vishwakarma; Shivam Tiwari; Shivangi Mishra; Surendra Pratap Mishra; Tribhuwan Mohan Mohapatra; Umesh Chaudhary
EPI_ISL_3200821, EPI_ISL_3265677	Multidisciplinary Research Unit, Institute of Medical Sciences, Banaras Hindu University, Varanasi -221005	Multidisciplinary Research Unit, Institute of Medical Sciences, Banaras Hindu University, Varanasi -221005	Abhay Kumar Yadav; Ajay Kumar Yadav; Ashish Ashish; Chetan Sahni; Deepa Devadas; Gunjan Rai; Janhavi Yadav; Manpreet Kaur; Meghna Wadi; Nargis Khanam; Nitish Kumar Singh; Priyoneel Basu; Ragini Mishra; Royana Singh; Sangeeta Rai; Sarita Chowdhary; Saumya Singh; Saurabh Singh; Shani Vishwakarma; Shivam Tiwari; Shivangi Mishra; Surendra Pratap Mishra; Tribhuwan Mohan Mohapatra; Umesh Chaudhary
EPI_ISL_2227163	Multidisiciplinary Research Unit, Institute of Medical Sciences, Banaras Hindu University, Varanasi - 221005	Multidisiciplinary Research Unit, Institute of Medical Sciences, Banaras Hindu University, Varanasi - 221005	Abhay Kumar Yadav; Ashish; Chetan Sahni; Deepa Devadas; Manpreet Kaur; Nitish Kumar Singh; Priyoneel Basu; Royana Singh; Saumya Singh; Shani Vishwakarma; Shivani Mishra; Surendra Pratap Mishra; Tribhuwan Mohan Mohapatra
EPI_ISL_528683, EPI_ISL_528684	NCDC Institute of Genomics and Integrative Biology	NCDC Institute of Genomics and Integrative Biology	Ajit Shewale; Akshay Kanakan; Anurag Agrawal; Azka Khan; Bharathram Uppili; Mahesh S. Dhar; Mohammed Faruq; Partha Rakshit; Pooja Sharma; RadhaKrishnan VS; Rajesh Pandey; Robin Marwal; Sandhya Kabra; Simmi Tiwari; Sujeet Singh; Tushar Nale; Vivekanand A
EPI_ISL_581505, EPI_ISL_581506, EPI_ISL_581507, EPI_ISL_614157	NCDC/IGIB	NCDC/IGIB	Ajit Shewale; Akshay Kanakan; Anurag Agrawal; Azka Khan; Bharathram Uppili; Mahesh S. Dhar; Mohammed Faruq; Nishu Tyagi; Partha Rakshit; Pooja Sharma; RadhaKrishnan VS; Rajesh Pandey; Robin Marwal; Sandhya Kabra; Simmi Tiwari; Sujeet Singh; Tushar Nale; Vivekanand A
EPI_ISL_1844765, EPI_ISL_1844767, EPI_ISL_1844769, EPI_ISL_1844771, EPI_ISL_1844773	NORTHERN RAILWAY CENTRAL HOSPITAL	Biotechnology Division, NCDC	Hema Gogia; Hemlata Lall; Kalaiarasan Ponnusamy; Mahesh S Dhar; Manoj K Singh; Meena Datta; Partha Rakshit; Preeti Madan; Priyanka Singh; Radhakrishnan V. S; Robin Marwal; Sandhya Kabra; Sujeet K Singh; Uma Sharma
EPI_ISL_2099534, EPI_ISL_2099535, EPI_ISL_2099536, EPI_ISL_2099537, EPI_ISL_2099538, EPI_ISL_2099539, EPI_ISL_2099540, EPI_ISL_2099541, EPI_ISL_2099542, EPI_ISL_2099543, EPI_ISL_2099544, EPI_ISL_2099545, EPI_ISL_2099546, EPI_ISL_2099547, EPI_ISL_2099548, EPI_ISL_2099549, EPI_ISL_2099550, EPI_ISL_2099551, EPI_ISL_2099552, EPI_ISL_2099553, EPI_ISL_2099554, EPI_ISL_2099555, EPI_ISL_2099556, EPI_ISL_2099557, EPI_ISL_2099558, EPI_ISL_2099559, EPI_ISL_2099560, EPI_ISL_2099561, EPI_ISL_2099562, EPI_ISL_2099563, EPI_ISL_2099564, EPI_ISL_2099565, EPI_ISL_2099566, EPI_ISL_2099567, EPI_ISL_2099568, EPI_ISL_2099569, EPI_ISL_2099570, EPI_ISL_2099571, EPI_ISL_2099572, EPI_ISL_2099573, EPI_ISL_2099574, EPI_ISL_2099575, EPI_ISL_2099576, EPI_ISL_2099577, EPI_ISL_2099578, EPI_ISL_2099579, EPI_ISL_2099580, EPI_ISL_2099581, EPI_ISL_2099582, EPI_ISL_2099583, EPI_ISL_2099584, EPI_ISL_2099585, EPI_ISL_2099586, EPI_ISL_2099587, EPI_ISL_2099588, EPI_ISL_2099589, EPI_ISL_2099590, EPI_ISL_2099591, EPI_ISL_2099592, EPI_ISL_2099593, EPI_ISL_2099594, EPI_ISL_2099595, EPI_ISL_2099596, EPI_ISL_2099597, EPI_ISL_2099598, EPI_ISL_2099599, EPI_ISL_2099600, EPI_ISL_2099601, EPI_ISL_2099602, EPI_ISL_2099603, EPI_ISL_2099604, EPI_ISL_2099605, EPI_ISL_2099606, EPI_ISL_2099607, EPI_ISL_2099608, EPI_ISL_2099609, EPI_ISL_2099610, EPI_ISL_2099611, EPI_ISL_2099612, EPI_ISL_2099613, EPI_ISL_2099614, EPI_ISL_2099615, EPI_ISL_2099616, EPI_ISL_2099617, EPI_ISL_2099618, EPI_ISL_2099619, EPI_ISL_2099620, EPI_ISL_2099621, EPI_ISL_2099622, EPI_ISL_2099623, EPI_ISL_2099624, EPI_ISL_2099625, EPI_ISL_2099626, EPI_ISL_2099627, EPI_ISL_2099628, EPI_ISL_2099629, EPI_ISL_2099630, EPI_ISL_2099631, EPI_ISL_2099632, EPI_ISL_2099633, EPI_ISL_2099634, EPI_ISL_2099635, EPI_ISL_2099636, EPI_ISL_2099637, EPI_ISL_2099638, EPI_ISL_2099639, EPI_ISL_2099640, EPI_ISL_2099641, EPI_ISL_2099642, EPI_ISL_2099643, EPI_ISL_2099644, EPI_ISL_2099645, EPI_ISL_2099646, EPI_ISL_2099647, EPI_ISL_2099648, EPI_ISL_2099649, EPI_ISL_2099650, EPI_ISL_2099651, EPI_ISL_2099652, EPI_ISL_2099653, EPI_ISL_2099654, EPI_ISL_2099655, EPI_ISL_2099656, EPI_ISL_2099657, EPI_ISL_2099658, EPI_ISL_2099659, EPI_ISL_2099660, EPI_ISL_2099661, EPI_ISL_2099662, EPI_ISL_2099663, EPI_ISL_2099664, EPI_ISL_2099665, EPI_ISL_2099666, EPI_ISL_2099667, EPI_ISL_2099668, EPI_ISL_2099669, EPI_ISL_2099670, EPI_ISL_2099671, EPI_ISL_2099672, EPI_ISL_2099673, EPI_ISL_2099674, EPI_ISL_2099675, EPI_ISL_2099676, EPI_ISL_2099677, EPI_ISL_2099678, EPI_ISL_2099679, EPI_ISL_2099680, EPI_ISL_2099681, EPI_ISL_2099682, EPI_ISL_2099683, EPI_ISL_2099684, EPI_ISL_2099685, EPI_ISL_2099686, EPI_ISL_2099687, EPI_ISL_2099689, EPI_ISL_2099690, EPI_ISL_2099691, EPI_ISL_2099692, EPI_ISL_2099693, EPI_ISL_2099694, EPI_ISL_2099695, EPI_ISL_2099696, EPI_ISL_2099697, EPI_ISL_2099698, EPI_ISL_2099699, EPI_ISL_2099700, EPI_ISL_2099701, EPI_ISL_2099702, EPI_ISL_2099703, EPI_ISL_2099704, EPI_ISL_2099705, EPI_ISL_2099706, EPI_ISL_2099707, EPI_ISL_2099708, EPI_ISL_2099709, EPI_ISL_2099710, EPI_ISL_2099711, EPI_ISL_2099713, EPI_ISL_2099714, EPI_ISL_2099715, EPI_ISL_2099716, EPI_ISL_2099717, EPI_ISL_2099718, EPI_ISL_2099719, EPI_ISL_2099720, EPI_ISL_2099721, EPI_ISL_2099722, EPI_ISL_2099723, EPI_ISL_2099724, EPI_ISL_2099725, EPI_ISL_2099726, EPI_ISL_2099727, EPI_ISL_2099728, EPI_ISL_2099729, EPI_ISL_2099733, EPI_ISL_2099734, EPI_ISL_2099735, EPI_ISL_2099737, EPI_ISL_2099738, EPI_ISL_2099739, EPI_ISL_2099740, EPI_ISL_2099741, EPI_ISL_2099742, EPI_ISL_2099743, EPI_ISL_2099744, EPI_ISL_2099745, EPI_ISL_2099746, EPI_ISL_2099747, EPI_ISL_2099748, EPI_ISL_2099749, EPI_ISL_2099752, EPI_ISL_2099753, EPI_ISL_2099754, EPI_ISL_2099755, EPI_ISL_2099756, EPI_ISL_2099757, EPI_ISL_2099758, EPI_ISL_2099759, EPI_ISL_2099760, EPI_ISL_2099761, EPI_ISL_2099762, EPI_ISL_2099763, EPI_ISL_2099764, EPI_ISL_2099765, EPI_ISL_2099766, EPI_ISL_2099767, EPI_ISL_2099768, EPI_ISL_2233467, EPI_ISL_2233468, EPI_ISL_2233469, EPI_ISL_2233470, EPI_ISL_2233471, EPI_ISL_2233472, EPI_ISL_2233473, EPI_ISL_2233474, EPI_ISL_2233475, EPI_ISL_2233476, EPI_ISL_2233477, EPI_ISL_2233478, EPI_ISL_2233479, EPI_ISL_2233480, EPI_ISL_2233481, EPI_ISL_2233482, EPI_ISL_2233483, EPI_ISL_2233484, EPI_ISL_2233485, EPI_ISL_2233486, EPI_ISL_2233487, EPI_ISL_2233488, EPI_ISL_2233489, EPI_ISL_2233490, EPI_ISL_2233491, EPI_ISL_2233492, EPI_ISL_2233493, EPI_ISL_2233494, EPI_ISL_2233495, EPI_ISL_2233496, EPI_ISL_2233497, EPI_ISL_2233498, EPI_ISL_2233499, EPI_ISL_2233500, EPI_ISL_2233501, EPI_ISL_2233502, EPI_ISL_2233503, EPI_ISL_2233504, EPI_ISL_2233505, EPI_ISL_2233506, EPI_ISL_2233507, EPI_ISL_2233508, EPI_ISL_2233509, EPI_ISL_2233510, EPI_ISL_2233511, EPI_ISL_2233512, EPI_ISL_2233513, EPI_ISL_2233514, EPI_ISL_2233515, EPI_ISL_2233516, EPI_ISL_2233517, EPI_ISL_2233518, EPI_ISL_2233519, EPI_ISL_2233520, EPI_ISL_2233521, EPI_ISL_2233522, EPI_ISL_2233523, EPI_ISL_2233524, EPI_ISL_2233525, EPI_ISL_2233526, EPI_ISL_2233527, EPI_ISL_2233528, EPI_ISL_2233529, EPI_ISL_2233530, EPI_ISL_2233531, EPI_ISL_2233532, EPI_ISL_2233533, EPI_ISL_2233534, EPI_ISL_2233535, EPI_ISL_2233536, EPI_ISL_2233537, EPI_ISL_2233538, EPI_ISL_2233539, EPI_ISL_2233540, EPI_ISL_2233541, EPI_ISL_2233542, EPI_ISL_2233543, EPI_ISL_2233544, EPI_ISL_2233545, EPI_ISL_2233546, EPI_ISL_2233547, EPI_ISL_2233548, EPI_ISL_2233549, EPI_ISL_2233550, EPI_ISL_2233551, EPI_ISL_2233552, EPI_ISL_2233553, EPI_ISL_2233554, EPI_ISL_2233555, EPI_ISL_2233556, EPI_ISL_2233557, EPI_ISL_2233558, EPI_ISL_2233559, EPI_ISL_2233560, EPI_ISL_2233561, EPI_ISL_2233562, EPI_ISL_2233563, EPI_ISL_2233564, EPI_ISL_2233565, EPI_ISL_2233566, EPI_ISL_2233567, EPI_ISL_2233568, EPI_ISL_2233569, EPI_ISL_2233570, EPI_ISL_2233571, EPI_ISL_2233572, EPI_ISL_2233573, EPI_ISL_2233574, EPI_ISL_2233575, EPI_ISL_2233576, EPI_ISL_2233577, EPI_ISL_2233578, EPI_ISL_2233579, EPI_ISL_2233580, EPI_ISL_2233581, EPI_ISL_2233582, EPI_ISL_2233583, EPI_ISL_2233584, EPI_ISL_2233585, EPI_ISL_2233586, EPI_ISL_2233587, EPI_ISL_2233588, EPI_ISL_2233589, EPI_ISL_2233590, EPI_ISL_2233591, EPI_ISL_2233592, EPI_ISL_2233593, EPI_ISL_2233594, EPI_ISL_2233595, EPI_ISL_2233596, EPI_ISL_2233597, EPI_ISL_2233598, EPI_ISL_2233599, EPI_ISL_2233600, EPI_ISL_2233601, EPI_ISL_2233602, EPI_ISL_2233603, EPI_ISL_2233604, EPI_ISL_2233605, EPI_ISL_2233606, EPI_ISL_2233607, EPI_ISL_2233608, EPI_ISL_2233609, EPI_ISL_2233610, EPI_ISL_2233611, EPI_ISL_2233612, EPI_ISL_2233613, EPI_ISL_2233614, EPI_ISL_2233615, EPI_ISL_2233616, EPI_ISL_2233617, EPI_ISL_2233618, EPI_ISL_2233619, EPI_ISL_2233620, EPI_ISL_2233621, EPI_ISL_2233622, EPI_ISL_2233623, EPI_ISL_2233624, EPI_ISL_2233625, EPI_ISL_2233626, EPI_ISL_2233627, EPI_ISL_2233628, EPI_ISL_2233629, EPI_ISL_2233630, EPI_ISL_2233631, EPI_ISL_2233632, EPI_ISL_2233633, EPI_ISL_2233634, EPI_ISL_2233635, EPI_ISL_2233636, EPI_ISL_2233637, EPI_ISL_2233638, EPI_ISL_2233639, EPI_ISL_2233640, EPI_ISL_2233641, EPI_ISL_2233642, EPI_ISL_2233643, EPI_ISL_2233644, EPI_ISL_2233645, EPI_ISL_2233646, EPI_ISL_2233647, EPI_ISL_2233648, EPI_ISL_2233649, EPI_ISL_2233650, EPI_ISL_2233651, EPI_ISL_2233652, EPI_ISL_2233653, EPI_ISL_2233654, EPI_ISL_2233655, EPI_ISL_2233656, EPI_ISL_2233657, EPI_ISL_2233658, EPI_ISL_2233659, EPI_ISL_2233660, EPI_ISL_2233661, EPI_ISL_2233662, EPI_ISL_2233663, EPI_ISL_2233664, EPI_ISL_2233665, EPI_ISL_2233666, EPI_ISL_2233667, EPI_ISL_2233668			
see above	National Centre for Disease Control	CDFD -INSACOG	Ashwin Dalal; Asmita Gupta; Divya Vashisht; Murali Bashyam; Pratyusha Bala; To be addded later; Vinay Donipadi
EPI_ISL_2448112, EPI_ISL_2448113, EPI_ISL_2448114, EPI_ISL_2448115, EPI_ISL_2448116, EPI_ISL_2448117, EPI_ISL_2448118, EPI_ISL_2448119, EPI_ISL_2448120, EPI_ISL_2448121, EPI_ISL_2448122, EPI_ISL_2448123, EPI_ISL_2448124, EPI_ISL_2448125, EPI_ISL_2448126, EPI_ISL_2448127, EPI_ISL_2448128, EPI_ISL_2448129, EPI_ISL_2448130, EPI_ISL_2448131, EPI_ISL_2448132, EPI_ISL_2448133, EPI_ISL_2448134, EPI_ISL_2448135, EPI_ISL_2448136, EPI_ISL_2448137, EPI_ISL_2448138, EPI_ISL_2448139, EPI_ISL_2448140, EPI_ISL_2448141, EPI_ISL_2448142, EPI_ISL_2448143, EPI_ISL_2448144, EPI_ISL_2448145, EPI_ISL_2448146, EPI_ISL_2448147, EPI_ISL_2448148, EPI_ISL_2448149, EPI_ISL_2448150, EPI_ISL_2448151, EPI_ISL_2448152, EPI_ISL_2448153, EPI_ISL_2448154, EPI_ISL_2448155, EPI_ISL_2448156, EPI_ISL_2448157, EPI_ISL_2448158, EPI_ISL_2448159, EPI_ISL_2448161, EPI_ISL_2448162, EPI_ISL_2448163, EPI_ISL_2448164, EPI_ISL_2448165, EPI_ISL_2448166, EPI_ISL_2448167, EPI_ISL_2448168, EPI_ISL_2448169, EPI_ISL_2448170, EPI_ISL_2448171, EPI_ISL_2448172, EPI_ISL_2448173, EPI_ISL_2448174, EPI_ISL_2448175, EPI_ISL_2448176, EPI_ISL_2448177, EPI_ISL_2448178, EPI_ISL_2448179, EPI_ISL_2448180, EPI_ISL_2448181, EPI_ISL_2448182, EPI_ISL_2448183, EPI_ISL_2448184, EPI_ISL_2448185, EPI_ISL_2448186, EPI_ISL_2448187, EPI_ISL_2448188, EPI_ISL_2448189, EPI_ISL_2448190, EPI_ISL_2448191, EPI_ISL_2448192, EPI_ISL_2448193, EPI_ISL_2448194, EPI_ISL_2448195, EPI_ISL_2448196, EPI_ISL_2448197, EPI_ISL_2448198, EPI_ISL_2448199, EPI_ISL_2448200, EPI_ISL_2448201, EPI_ISL_2448202, EPI_ISL_2448203, EPI_ISL_2448204, EPI_ISL_2448205, EPI_ISL_2448206, EPI_ISL_2448207, EPI_ISL_2448208, EPI_ISL_2448209, EPI_ISL_2448210, EPI_ISL_2448211, EPI_ISL_2448212, EPI_ISL_2448213, EPI_ISL_2448214, EPI_ISL_2448215, EPI_ISL_2448216, EPI_ISL_2448217, EPI_ISL_2448218, EPI_ISL_2448219, EPI_ISL_2448220, EPI_ISL_2448221, EPI_ISL_2448222, EPI_ISL_2448223, EPI_ISL_2448224, EPI_ISL_2448225, EPI_ISL_2448226, EPI_ISL_2448227, EPI_ISL_2448228, EPI_ISL_2448229, EPI_ISL_2448230, EPI_ISL_2448231, EPI_ISL_2448232, EPI_ISL_2448233, EPI_ISL_2448234, EPI_ISL_2448235, EPI_ISL_2448236, EPI_ISL_2448237, EPI_ISL_2448238, EPI_ISL_2448239, EPI_ISL_2448240, EPI_ISL_2448241, EPI_ISL_2448242, EPI_ISL_2448243, EPI_ISL_2448244, EPI_ISL_2448245, EPI_ISL_2448246, EPI_ISL_2448247, EPI_ISL_2448248, EPI_ISL_2448249, EPI_ISL_2448250, EPI_ISL_2448251, EPI_ISL_2448252, EPI_ISL_2448253, EPI_ISL_2448254, EPI_ISL_2448255, EPI_ISL_2448256, EPI_ISL_2448257, EPI_ISL_2448258, EPI_ISL_2448259, EPI_ISL_2448260, EPI_ISL_2448261, EPI_ISL_2448262, EPI_ISL_2448263, EPI_ISL_2448264, EPI_ISL_2448265, EPI_ISL_2448266, EPI_ISL_2448267, EPI_ISL_2448268, EPI_ISL_2448269, EPI_ISL_2448270, EPI_ISL_2448271, EPI_ISL_2448272, EPI_ISL_2448273, EPI_ISL_2448274, EPI_ISL_2448275, EPI_ISL_2448276, EPI_ISL_2448277, EPI_ISL_2448278, EPI_ISL_2448279, EPI_ISL_2448280, EPI_ISL_2448281, EPI_ISL_2448282, EPI_ISL_2448283, EPI_ISL_2448284, EPI_ISL_2448285, EPI_ISL_2448286, EPI_ISL_2448287, EPI_ISL_2448288, EPI_ISL_2448289, EPI_ISL_2448290, EPI_ISL_2448291, EPI_ISL_2448292, EPI_ISL_2448293, EPI_ISL_2448294, EPI_ISL_2448295, EPI_ISL_2448296, EPI_ISL_2458624, EPI_ISL_2458625, EPI_ISL_2458626, EPI_ISL_2458627, EPI_ISL_2458628, EPI_ISL_2458630, EPI_ISL_2458631, EPI_ISL_2458632, EPI_ISL_2458633, EPI_ISL_2458634, EPI_ISL_2458635, EPI_ISL_2458637, EPI_ISL_2458638, EPI_ISL_2458639, EPI_ISL_2458640, EPI_ISL_2458641, EPI_ISL_2458642, EPI_ISL_2458643, EPI_ISL_2458644, EPI_ISL_2458645, EPI_ISL_2458646, EPI_ISL_2458647, EPI_ISL_2458648, EPI_ISL_2458649, EPI_ISL_2458650, EPI_ISL_2458651, EPI_ISL_2458652, EPI_ISL_2458653, EPI_ISL_2458654, EPI_ISL_2458655, EPI_ISL_2458656, EPI_ISL_2458657, EPI_ISL_2458658, EPI_ISL_2458659, EPI_ISL_2458660, EPI_ISL_2458661, EPI_ISL_2458662, EPI_ISL_2458663, EPI_ISL_2458664, EPI_ISL_2458665, EPI_ISL_2458666, EPI_ISL_2458667, EPI_ISL_2458669, EPI_ISL_2458670, EPI_ISL_2458671, EPI_ISL_2458672, EPI_ISL_2458673, EPI_ISL_2458674, EPI_ISL_2458675, EPI_ISL_2458676, EPI_ISL_2458677, EPI_ISL_2458678, EPI_ISL_2458679, EPI_ISL_2458680, EPI_ISL_2458681, EPI_ISL_2458682, EPI_ISL_2458683, EPI_ISL_2458684, EPI_ISL_2458685, EPI_ISL_2458686, EPI_ISL_2458687, EPI_ISL_2458688, EPI_ISL_2458689, EPI_ISL_2458690, EPI_ISL_2458691, EPI_ISL_2458692, EPI_ISL_2458693, EPI_ISL_2458694, EPI_ISL_2458695, EPI_ISL_2458696, EPI_ISL_2458697, EPI_ISL_2458698, EPI_ISL_2458699, EPI_ISL_2458700, EPI_ISL_2458701, EPI_ISL_2458702, EPI_ISL_2458703, EPI_ISL_2458704, EPI_ISL_2458705, EPI_ISL_2458706, EPI_ISL_2458707, EPI_ISL_2458708, EPI_ISL_2458709, EPI_ISL_2458710, EPI_ISL_2458711, EPI_ISL_2458712, EPI_ISL_2458713, EPI_ISL_2458714, EPI_ISL_2458715, EPI_ISL_2458716, EPI_ISL_2458717, EPI_ISL_2458718, EPI_ISL_2458719, EPI_ISL_2458720, EPI_ISL_2458721, EPI_ISL_2458722, EPI_ISL_2458723, EPI_ISL_2458724, EPI_ISL_2458725, EPI_ISL_2458726, EPI_ISL_2458727, EPI_ISL_2458728, EPI_ISL_2458729, EPI_ISL_2458730, EPI_ISL_2458731, EPI_ISL_2458732, EPI_ISL_2458733, EPI_ISL_2458734, EPI_ISL_2458735, EPI_ISL_2458736, EPI_ISL_2458737, EPI_ISL_2458738, EPI_ISL_2458739, EPI_ISL_2458740, EPI_ISL_2458741, EPI_ISL_2458742, EPI_ISL_2458743, EPI_ISL_2458744, EPI_ISL_2458745, EPI_ISL_2458746, EPI_ISL_2458747, EPI_ISL_2458748, EPI_ISL_2458749, EPI_ISL_2458750, EPI_ISL_2458751, EPI_ISL_2458752, EPI_ISL_2458753, EPI_ISL_2458754, EPI_ISL_2458755, EPI_ISL_2458756, EPI_ISL_2458757, EPI_ISL_2458758, EPI_ISL_2458759, EPI_ISL_2458760, EPI_ISL_2458761, EPI_ISL_2458762, EPI_ISL_2458763, EPI_ISL_2458764, EPI_ISL_2458765, EPI_ISL_2458766, EPI_ISL_2458767, EPI_ISL_2458768, EPI_ISL_2458769, EPI_ISL_2458770, EPI_ISL_2458771, EPI_ISL_2458772, EPI_ISL_2458773, EPI_ISL_2458774, EPI_ISL_2458775, EPI_ISL_2458776, EPI_ISL_2458777, EPI_ISL_2458778, EPI_ISL_2458779, EPI_ISL_2458780, EPI_ISL_2458781, EPI_ISL_2458782, EPI_ISL_2458783, EPI_ISL_2458784, EPI_ISL_2458785, EPI_ISL_2458786, EPI_ISL_2458787, EPI_ISL_2458788, EPI_ISL_2458789, EPI_ISL_2458790, EPI_ISL_2458791, EPI_ISL_2458792, EPI_ISL_2458793, EPI_ISL_2458794, EPI_ISL_2458795, EPI_ISL_2458796, EPI_ISL_2458797, EPI_ISL_2458798, EPI_ISL_2458799, EPI_ISL_2458800, EPI_ISL_2458801, EPI_ISL_2458802, EPI_ISL_2458803, EPI_ISL_2458804, EPI_ISL_2458805, EPI_ISL_2458806, EPI_ISL_2458807, EPI_ISL_2458808, EPI_ISL_2458809, EPI_ISL_2458810, EPI_ISL_2458811, EPI_ISL_2458812, EPI_ISL_2458813, EPI_ISL_2458814, EPI_ISL_2458815, EPI_ISL_2458816, EPI_ISL_2458817, EPI_ISL_2458818, EPI_ISL_2458819, EPI_ISL_2458820, EPI_ISL_2458821, EPI_ISL_2458822, EPI_ISL_2458823, EPI_ISL_2458824, EPI_ISL_2458825, EPI_ISL_2458826, EPI_ISL_2458827, EPI_ISL_2458828, EPI_ISL_2458829, EPI_ISL_2458830, EPI_ISL_2458831, EPI_ISL_2458832, EPI_ISL_2458833, EPI_ISL_2458834, EPI_ISL_2458835, EPI_ISL_2458836, EPI_ISL_2458837, EPI_ISL_2458838, EPI_ISL_2458839, EPI_ISL_2458840, EPI_ISL_2458841, EPI_ISL_2458842, EPI_ISL_2458843, EPI_ISL_2458844, EPI_ISL_2458845, EPI_ISL_2458846, EPI_ISL_2458847, EPI_ISL_2458848, EPI_ISL_2458849, EPI_ISL_2458850, EPI_ISL_2458851, EPI_ISL_2458852, EPI_ISL_2458853, EPI_ISL_2458854, EPI_ISL_2458855, EPI_ISL_2458856, EPI_ISL_2458857, EPI_ISL_2458858, EPI_ISL_2458859, EPI_ISL_2458860, EPI_ISL_2458861, EPI_ISL_2458862, EPI_ISL_2458863, EPI_ISL_2458864, EPI_ISL_2458865, EPI_ISL_2458866, EPI_ISL_2458867, EPI_ISL_2458868, EPI_ISL_2458869, EPI_ISL_2458870, EPI_ISL_2458871, EPI_ISL_2458872, EPI_ISL_2458873, EPI_ISL_2458874, EPI_ISL_2458875, EPI_ISL_2458876, EPI_ISL_2458877, EPI_ISL_2458878, EPI_ISL_2458879, EPI_ISL_2458880, EPI_ISL_2458881, EPI_ISL_2458882, EPI_ISL_2458883, EPI_ISL_2458884, EPI_ISL_2458885, EPI_ISL_2458886, EPI_ISL_2458887, EPI_ISL_2458888, EPI_ISL_2458889, EPI_ISL_2458890, EPI_ISL_2458891, EPI_ISL_2458892, EPI_ISL_2458893, EPI_ISL_2458894, EPI_ISL_2458895, EPI_ISL_2458896, EPI_ISL_2458897, EPI_ISL_2458898, EPI_ISL_2458899, EPI_ISL_2458900, EPI_ISL_2458901, EPI_ISL_2458902, EPI_ISL_2458903, EPI_ISL_2458904, EPI_ISL_2458905, EPI_ISL_2458906, EPI_ISL_2458907, EPI_ISL_2458908, EPI_ISL_2458909, EPI_ISL_2458910, EPI_ISL_2458911, EPI_ISL_2458912, EPI_ISL_2458913, EPI_ISL_2458914, EPI_ISL_2458915, EPI_ISL_2458916, EPI_ISL_2458917, EPI_ISL_2458918, EPI_ISL_2458919, EPI_ISL_2458920, EPI_ISL_2458921, EPI_ISL_2458922, EPI_ISL_2458923, EPI_ISL_2458924, EPI_ISL_2458925, EPI_ISL_2458926, EPI_ISL_2458927, EPI_ISL_2458928, EPI_ISL_2458929, EPI_ISL_2458930, EPI_ISL_2458931, EPI_ISL_2458932, EPI_ISL_2458933, EPI_ISL_2458934, EPI_ISL_2458935, EPI_ISL_2458936, EPI_ISL_2458937, EPI_ISL_2458938, EPI_ISL_2458939, EPI_ISL_2458940, EPI_ISL_2458941, EPI_ISL_2458942, EPI_ISL_2458943, EPI_ISL_2458944, EPI_ISL_2458945, EPI_ISL_2458946, EPI_ISL_2458947, EPI_ISL_2458948, EPI_ISL_2458949, EPI_ISL_2458950, EPI_ISL_2458951, EPI_ISL_2458952, EPI_ISL_2458953, EPI_ISL_2458954, EPI_ISL_2458955, EPI_ISL_2458956, EPI_ISL_2458957, EPI_ISL_2458958, EPI_ISL_2458959, EPI_ISL_2458960, EPI_ISL_2458961, EPI_ISL_2458962, EPI_ISL_2458963, EPI_ISL_2458964, EPI_ISL_2458965, EPI_ISL_2458966, EPI_ISL_2458967, EPI_ISL_2458968, EPI_ISL_2458969, EPI_ISL_2458970, EPI_ISL_2458971, EPI_ISL_2458972, EPI_ISL_2458973, EPI_ISL_2458974, EPI_ISL_2458975, EPI_ISL_2458976, EPI_ISL_2458977, EPI_ISL_2458978, EPI_ISL_2458979, EPI_ISL_2458980, EPI_ISL_2458981, EPI_ISL_2458982, EPI_ISL_2458983, EPI_ISL_2458984, EPI_ISL_2458985, EPI_ISL_2458986, EPI_ISL_2458987, EPI_ISL_2458988, EPI_ISL_2458989, EPI_ISL_2458990, EPI_ISL_2458991, EPI_ISL_2458992, EPI_ISL_2458993, EPI_ISL_2458994, EPI_ISL_2458995, EPI_ISL_2458996, EPI_ISL_2458997, EPI_ISL_2458998, EPI_ISL_2458999, EPI_ISL_2459000, EPI_ISL_2459001, EPI_ISL_2459002, EPI_ISL_2459003, EPI_ISL_2459004, EPI_ISL_2459005, EPI_ISL_2459006, EPI_ISL_2459007, EPI_ISL_2459008, EPI_ISL_2459009, EPI_ISL_2459010, EPI_ISL_2459011, EPI_ISL_2459012, EPI_ISL_2459013, EPI_ISL_2459014, EPI_ISL_2459015, EPI_ISL_2459016, EPI_ISL_2459017, EPI_ISL_2459018, EPI_ISL_2459019, EPI_ISL_2459020, EPI_ISL_2459021, EPI_ISL_2459022, EPI_ISL_2459023, EPI_ISL_2459024, EPI_ISL_2459025, EPI_ISL_2459026, EPI_ISL_2459027, EPI_ISL_2459028, EPI_ISL_2459029, EPI_ISL_2459030, EPI_ISL_2459031, EPI_ISL_2459032, EPI_ISL_2459033, EPI_ISL_2459034, EPI_ISL_2459035, EPI_ISL_2459036, EPI_ISL_2459037, EPI_ISL_2459038, EPI_ISL_2459039, EPI_ISL_2459040, EPI_ISL_2459041, EPI_ISL_2459042, EPI_ISL_2459043, EPI_ISL_2459044, EPI_ISL_2459045, EPI_ISL_2459046, EPI_ISL_2459047, EPI_ISL_2459048, EPI_ISL_2459049, EPI_ISL_2459050, EPI_ISL_2459051, EPI_ISL_2459052, EPI_ISL_2459053, EPI_ISL_2459054, EPI_ISL_2459055, EPI_ISL_2459056, EPI_ISL_2459057, EPI_ISL_2459058, EPI_ISL_2459059, EPI_ISL_2459060, EPI_ISL_2459061, EPI_ISL_2459062, EPI_ISL_2459063, EPI_ISL_2459064, EPI_ISL_2459065, EPI_ISL_2459066, EPI_ISL_2459067, EPI_ISL_2459068, EPI_ISL_2459069, EPI_ISL_2459070, EPI_ISL_2459071, EPI_ISL_2459072, EPI_ISL_2459073, EPI_ISL_2459074, EPI_ISL_2459075, EPI_ISL_2459076, EPI_ISL_2459077, EPI_ISL_2459078, EPI_ISL_2459079, EPI_ISL_2459080, EPI_ISL_2459081, EPI_ISL_2459082, EPI_ISL_2459083, EPI_ISL_2459084, EPI_ISL_2459085, EPI_ISL_2459086, EPI_ISL_2459087, EPI_ISL_2459088, EPI_ISL_2459089, EPI_ISL_2459090, EPI_ISL_2459091, EPI_ISL_2459092, EPI_ISL_2459093, EPI_ISL_2459094, EPI_ISL_2459095, EPI_ISL_2459096, EPI_ISL_2459097, EPI_ISL_2459098, EPI_ISL_2459099, EPI_ISL_2459100, EPI_ISL_2459101, EPI_ISL_2459102, EPI_ISL_2459103, EPI_ISL_2459104, EPI_ISL_2459105, EPI_ISL_2459106, EPI_ISL_2459107, EPI_ISL_2459108, EPI_ISL_2459109, EPI_ISL_2459110, EPI_ISL_2459111, EPI_ISL_2459112, EPI_ISL_2459113, EPI_ISL_2459114, EPI_ISL_2459115, EPI_ISL_2459116, EPI_ISL_2459117, EPI_ISL_2459118, EPI_ISL_2459119, EPI_ISL_2459120, EPI_ISL_2459121, EPI_ISL_2459122, EPI_ISL_2459123, EPI_ISL_2459124, EPI_ISL_2459125, EPI_ISL_2459126, EPI_ISL_2459127, EPI_ISL_2459128, EPI_ISL_2459129, EPI_ISL_2459130, EPI_ISL_2459131, EPI_ISL_2459132, EPI_ISL_2459133, EPI_ISL_2459134, EPI_ISL_2459135, EPI_ISL_2459136, EPI_ISL_2459137, EPI_ISL_2459138, EPI_ISL_2459139, EPI_ISL_2459140, EPI_ISL_2459141, EPI_ISL_2459142, EPI_ISL_2459143, EPI_ISL_2459144, EPI_ISL_2459145, EPI_ISL_2459146, EPI_ISL_2459147, EPI_ISL_2459148, EPI_ISL_2459149, EPI_ISL_2459150, EPI_ISL_2459151, EPI_ISL_2459152, EPI_ISL_2459153, EPI_ISL_2459154, EPI_ISL_2459155, EPI_ISL_2459156, EPI_ISL_2459157, EPI_ISL_2459158, EPI_ISL_2459159, EPI_ISL_2459160, EPI_ISL_2459161, EPI_ISL_2459162, EPI_ISL_2459163, EPI_ISL_2459164, EPI_ISL_2459165, EPI_ISL_2459166, EPI_ISL_2459167, EPI_ISL_2459168, EPI_ISL_2459169, EPI_ISL_2459170, EPI_ISL_2459171, EPI_ISL_2459172, EPI_ISL_2459173, EPI_ISL_2459174, EPI_ISL_2459175, EPI_ISL_2459176, EPI_ISL_2459177, EPI_ISL_2459178, EPI_ISL_2459179, EPI_ISL_2459180, EPI_ISL_2459181, EPI_ISL_2459182, EPI_ISL_2459183, EPI_ISL_2459184, EPI_ISL_2459185, EPI_ISL_2459186, EPI_ISL_2459187, EPI_ISL_2459188, EPI_ISL_2459189, EPI_ISL_2459190, EPI_ISL_2459191, EPI_ISL_2459192, EPI_ISL_2459193, EPI_ISL_2459194, EPI_ISL_2459195, EPI_ISL_2459196, EPI_ISL_2459197, EPI_ISL_2459198, EPI_ISL_2459199, EPI_ISL_2459200, EPI_ISL_2459201, EPI_ISL_2459202, EPI_ISL_2459203, EPI_ISL_2459204, EPI_ISL_2459205, EPI_ISL_2459206, EPI_ISL_2459207, EPI_ISL_2459208, EPI_ISL_2459209, EPI_ISL_2459210, EPI_ISL_2459211, EPI_ISL_2459212, EPI_ISL_2459213, EPI_ISL_2459214, EPI_ISL_2459215, EPI_ISL_2459216, EPI_ISL_2459217, EPI_ISL_2459218, EPI_ISL_2459219, EPI_ISL_2459220, EPI_ISL_2459221, EPI_ISL_2459222, EPI_ISL_2459223, EPI_ISL_2459224, EPI_ISL_2459225, EPI_ISL_2459226, EPI_ISL_2459227, EPI_ISL_2459228, EPI_ISL_2459229, EPI_ISL_2459230, EPI_ISL_2459231, EPI_ISL_2459232, EPI_ISL_2459233, EPI_ISL_2459234, EPI_ISL_2459235, EPI_ISL_2459236, EPI_ISL_2459237, EPI_ISL_2459238, EPI_ISL_2459239, EPI_ISL_2459240, EPI_ISL_2459241, EPI_ISL_2459242, EPI_ISL_2459243, EPI_ISL_2459244, EPI_ISL_2459245, EPI_ISL_2459246, EPI_ISL_2459247, EPI_ISL_2459248, EPI_ISL_2459249, EPI_ISL_2459250, EPI_ISL_2459251, EPI_ISL_2459252, EPI_ISL_2459253, EPI_ISL_2459254, EPI_ISL_2459255, EPI_ISL_2459256, EPI_ISL_2459257, EPI_ISL_2459258, EPI_ISL_2459259, EPI_ISL_2459260, EPI_ISL_2459261, EPI_ISL_2459262, EPI_ISL_2459263, EPI_ISL_2459264, EPI_ISL_2459265, EPI_ISL_2459266, EPI_ISL_2459267, EPI_ISL_2459268, EPI_ISL_2459269, EPI_ISL_2459270, EPI_ISL_2459271, EPI_ISL_2459272, EPI_ISL_2459273, EPI_ISL_2459274, EPI_ISL_2459275, EPI_ISL_2459276, EPI_ISL_2459277, EPI_ISL_2459278, EPI_ISL_2459279, EPI_ISL_2459280, EPI_ISL_2459281, EPI_ISL_2459282, EPI_ISL_2459283, EPI_ISL_2459284, EPI_ISL_2459285, EPI_ISL_2459286, EPI_ISL_2459287, EPI_ISL_2459288, EPI_ISL_2459289, EPI_ISL_2459290, EPI_ISL_2459291, EPI_ISL_2459292, EPI_ISL_2459293, EPI_ISL_2459294, EPI_ISL_2459295, EPI_ISL_2459296, EPI_ISL_2459297, EPI_ISL_2459298, EPI_ISL_2459299, EPI_ISL_2459300, EPI_ISL_2459301, EPI_ISL_2459302, EPI_ISL_2459303, EPI_ISL_2459304, EPI_ISL_2459305, EPI_ISL_2459306, EPI_ISL_2459307, EPI_ISL_2459308, EPI_ISL_2459309, EPI_ISL_2459310, EPI_ISL_2459311, EPI_ISL_2459312, EPI_ISL_2459313, EPI_ISL_2459314, EPI_ISL_2459315, EPI_ISL_2459316, EPI_ISL_2459317, EPI_ISL_2459318, EPI_ISL_2459319, EPI_ISL_2459320, EPI_ISL_2459321, EPI_ISL_2459322, EPI_ISL_2459323, EPI_ISL_2459324, EPI_ISL_2459325, EPI_ISL_2459326, EPI_ISL_2459327, EPI_ISL_2459328, EPI_ISL_2459329, EPI_ISL_2459330, EPI_ISL_2459331, EPI_ISL_2459332, EPI_ISL_2459333, EPI_ISL_2459334, EPI_ISL_2459335, EPI_ISL_2459336, EPI_ISL_2459337, EPI_ISL_2459338, EPI_ISL_2459339, EPI_ISL_2459340, EPI_ISL_2459341, EPI_ISL_2459342, EPI_ISL_2459343, EPI_ISL_2459344, EPI_ISL_2459345, EPI_ISL_2459346, EPI_ISL_2459347, EPI_ISL_2459348, EPI_ISL_2459349, EPI_ISL_2459350, EPI_ISL_2459351, EPI_ISL_2459352, EPI_ISL_2459353, EPI_ISL_2459354, EPI_ISL_2459355, EPI_ISL_2459356, EPI_ISL_2459357, EPI_ISL_2459358, EPI_ISL_2459359, EPI_ISL_2459360, EPI_ISL_2459361, EPI_ISL_2459362, EPI_ISL_2459363, EPI_ISL_2459364, EPI_ISL_2459365, EPI_ISL_2459366, EPI_ISL_2459367, EPI_ISL_2459368, EPI_ISL_2459369, EPI_ISL_2459370, EPI_ISL_2459371, EPI_ISL_2459372, EPI_ISL_2459373, EPI_ISL_2459374, EPI_ISL_2459375, EPI_ISL_2459376, EPI_ISL_2459377, EPI_ISL_2459378, EPI_ISL_2459379, EPI_ISL_2459380, EPI_ISL_2459381, EPI_ISL_2459382, EPI_ISL_2459383, EPI_ISL_2459384, EPI_ISL_2459385, EPI_ISL_2459386, EPI_ISL_2459387, EPI_ISL_2459388, EPI_ISL_2459389, EPI_ISL_2459390, EPI_ISL_2459391, EPI_ISL_2459392, EPI_ISL_2459393, EPI_ISL_2459394, EPI_ISL_2459395, EPI_ISL_2459396, EPI_ISL_2459397, EPI_ISL_2459398, EPI_ISL_2459399, EPI_ISL_2459400, EPI_ISL_2459401, EPI_ISL_2459402, EPI_ISL_2459403, EPI_ISL_2459404, EPI_ISL_2459405, EPI_ISL_2459406, EPI_ISL_2459407, EPI_ISL_2459408, EPI_ISL_2459409, EPI_ISL_2459410, EPI_ISL_2459411, EPI_ISL_2459412, EPI_ISL_2459413, EPI_ISL_2459414, EPI_ISL_2459415, EPI_ISL_2459416, EPI_ISL_2459417, EPI_ISL_2459418, EPI_ISL_2459419, EPI_ISL_2459420, EPI_ISL_2459421, EPI_ISL_2459422, EPI_ISL_2459423, EPI_ISL_2459424, EPI_ISL_2459425, EPI_ISL_2459426, EPI_ISL_2459427, EPI_ISL_2459429, EPI_ISL_2459430, EPI_ISL_2459431, EPI_ISL_2459432, EPI_ISL_2459433,			

EPI_ISL_2459434, EPI_ISL_2459435, EPI_ISL_2459436, EPI_ISL_2459437, EPI_ISL_2459438, EPI_ISL_2459439, EPI_ISL_2459440, EPI_ISL_2459442, EPI_ISL_2459443, EPI_ISL_2459444, EPI_ISL_2459445, EPI_ISL_2459446, EPI_ISL_2459447, EPI_ISL_2459449, EPI_ISL_2459450, EPI_ISL_2459451, EPI_ISL_2459452, EPI_ISL_2459453, EPI_ISL_2459454, EPI_ISL_2459455, EPI_ISL_2459456, EPI_ISL_2459457,
EPI_ISL_2459458, EPI_ISL_2459459, EPI_ISL_2459460, EPI_ISL_2459461, EPI_ISL_2459462, EPI_ISL_2459463, EPI_ISL_2459464, EPI_ISL_2459465, EPI_ISL_2459466, EPI_ISL_2459467, EPI_ISL_2459469, EPI_ISL_2459470, EPI_ISL_2459471, EPI_ISL_2459472, EPI_ISL_2459473, EPI_ISL_2459474, EPI_ISL_2459475, EPI_ISL_2459476, EPI_ISL_2459477, EPI_ISL_2459478, EPI_ISL_2459479, EPI_ISL_2459480,
EPI_ISL_2459481, EPI_ISL_2459482, EPI_ISL_2459483, EPI_ISL_2459484, EPI_ISL_2459485, EPI_ISL_2459486, EPI_ISL_2459487, EPI_ISL_2459488, EPI_ISL_2459489, EPI_ISL_2459490, EPI_ISL_2459491, EPI_ISL_2459492, EPI_ISL_2459493, EPI_ISL_2459494, EPI_ISL_2459495, EPI_ISL_2459496, EPI_ISL_2459497, EPI_ISL_2459498, EPI_ISL_2459499, EPI_ISL_2459500, EPI_ISL_2459501, EPI_ISL_2459502,
EPI_ISL_2459503, EPI_ISL_2459504, EPI_ISL_2459505, EPI_ISL_2459506, EPI_ISL_2459507, EPI_ISL_2459508, EPI_ISL_2459509, EPI_ISL_2459510, EPI_ISL_2459511, EPI_ISL_2459512, EPI_ISL_2459513, EPI_ISL_2459514, EPI_ISL_2459515, EPI_ISL_2459516, EPI_ISL_2459517, EPI_ISL_2459518, EPI_ISL_2459519, EPI_ISL_2459520, EPI_ISL_2459521, EPI_ISL_2459522, EPI_ISL_2459523, EPI_ISL_2459524,
EPI_ISL_2459525, EPI_ISL_2459526, EPI_ISL_2459527, EPI_ISL_2459528, EPI_ISL_2459529, EPI_ISL_2459530, EPI_ISL_2459531, EPI_ISL_2459532, EPI_ISL_2459533, EPI_ISL_2459534, EPI_ISL_2459535, EPI_ISL_2459536, EPI_ISL_2459537, EPI_ISL_2459538, EPI_ISL_2459539, EPI_ISL_2459540, EPI_ISL_2459541, EPI_ISL_2459542, EPI_ISL_2459543, EPI_ISL_2459544, EPI_ISL_2459545, EPI_ISL_2459546,
EPI_ISL_2459547, EPI_ISL_2459548, EPI_ISL_2459549, EPI_ISL_2459550, EPI_ISL_2459551, EPI_ISL_2459552, EPI_ISL_2459553, EPI_ISL_2459554, EPI_ISL_2459555, EPI_ISL_2459556, EPI_ISL_2459557, EPI_ISL_2459558, EPI_ISL_2459559, EPI_ISL_2459560, EPI_ISL_2459561, EPI_ISL_2459562, EPI_ISL_2459563, EPI_ISL_2459564, EPI_ISL_2459565, EPI_ISL_2459566, EPI_ISL_2459567, EPI_ISL_2459568,
EPI_ISL_2459569, EPI_ISL_2459570, EPI_ISL_2459571, EPI_ISL_2459572, EPI_ISL_2459573, EPI_ISL_2459574, EPI_ISL_2459575, EPI_ISL_2459576, EPI_ISL_2459577, EPI_ISL_2459578, EPI_ISL_2459579, EPI_ISL_2459580, EPI_ISL_2459581, EPI_ISL_2459582, EPI_ISL_2459583, EPI_ISL_2459584, EPI_ISL_2459585, EPI_ISL_2459586, EPI_ISL_2459587, EPI_ISL_2459588, EPI_ISL_2459589, EPI_ISL_2459590,
EPI_ISL_2459591, EPI_ISL_2459592, EPI_ISL_2459593, EPI_ISL_2459594, EPI_ISL_2459595, EPI_ISL_2459596, EPI_ISL_2459597, EPI_ISL_2459598, EPI_ISL_2459599, EPI_ISL_2459600, EPI_ISL_2459601, EPI_ISL_2459602, EPI_ISL_2459603, EPI_ISL_2459604, EPI_ISL_2459605, EPI_ISL_2459606, EPI_ISL_2459607, EPI_ISL_2459608, EPI_ISL_2459609, EPI_ISL_2459610, EPI_ISL_2459611, EPI_ISL_2459612,
EPI_ISL_2459613, EPI_ISL_2459614, EPI_ISL_2459615, EPI_ISL_2459616, EPI_ISL_2459617, EPI_ISL_2459618, EPI_ISL_2459619, EPI_ISL_2459620, EPI_ISL_2459621, EPI_ISL_2459622, EPI_ISL_2459623, EPI_ISL_2459624, EPI_ISL_2459625, EPI_ISL_2459626, EPI_ISL_2459627, EPI_ISL_2459628, EPI_ISL_2459629, EPI_ISL_2459630, EPI_ISL_2459631, EPI_ISL_2459632, EPI_ISL_2459633, EPI_ISL_2459634,
EPI_ISL_2459635, EPI_ISL_2459636, EPI_ISL_2459637, EPI_ISL_2459638, EPI_ISL_2459639, EPI_ISL_2459640, EPI_ISL_2459641, EPI_ISL_2459642, EPI_ISL_2459643, EPI_ISL_2459644, EPI_ISL_2459645, EPI_ISL_2459646, EPI_ISL_2459647, EPI_ISL_2459648, EPI_ISL_2459649, EPI_ISL_2459650, EPI_ISL_2459651, EPI_ISL_2459652, EPI_ISL_2459653, EPI_ISL_2459654, EPI_ISL_2459655, EPI_ISL_2459656,
EPI_ISL_2459657, EPI_ISL_2459658, EPI_ISL_2459660, EPI_ISL_2459661, EPI_ISL_2459662, EPI_ISL_2459663, EPI_ISL_2459664, EPI_ISL_2459665, EPI_ISL_2459666, EPI_ISL_2459667, EPI_ISL_2459668, EPI_ISL_2459669, EPI_ISL_2459671, EPI_ISL_2459672, EPI_ISL_2459673, EPI_ISL_2459674, EPI_ISL_2459676, EPI_ISL_2459677, EPI_ISL_2459679, EPI_ISL_2459680, EPI_ISL_2459681, EPI_ISL_2459682,
EPI_ISL_2459683, EPI_ISL_2459684, EPI_ISL_2459685, EPI_ISL_2459686, EPI_ISL_2459687, EPI_ISL_2459688, EPI_ISL_2459689, EPI_ISL_2459690, EPI_ISL_2459691, EPI_ISL_2459693, EPI_ISL_2459694, EPI_ISL_2459695, EPI_ISL_2459696, EPI_ISL_2459697, EPI_ISL_2459698, EPI_ISL_2459700, EPI_ISL_2459701, EPI_ISL_2459702, EPI_ISL_2459703, EPI_ISL_2459705, EPI_ISL_2459706, EPI_ISL_2459707,
EPI_ISL_2459708, EPI_ISL_2459710, EPI_ISL_2459711, EPI_ISL_2459712, EPI_ISL_2459713, EPI_ISL_2459714, EPI_ISL_2459716, EPI_ISL_2459717, EPI_ISL_2459718, EPI_ISL_2459719, EPI_ISL_2459720, EPI_ISL_2459721, EPI_ISL_2459723, EPI_ISL_2459724, EPI_ISL_2459727, EPI_ISL_2459728, EPI_ISL_2459729, EPI_ISL_2459730, EPI_ISL_2459732, EPI_ISL_2459733, EPI_ISL_2459734, EPI_ISL_2459735,
EPI_ISL_2459736, EPI_ISL_2459737, EPI_ISL_2459742, EPI_ISL_2459744, EPI_ISL_2459746, EPI_ISL_2459750, EPI_ISL_2459755, EPI_ISL_2459756, EPI_ISL_2459761, EPI_ISL_2459762, EPI_ISL_2459764, EPI_ISL_2459766, EPI_ISL_2459768, EPI_ISL_2459770, EPI_ISL_2459774, EPI_ISL_2459779, EPI_ISL_2459784, EPI_ISL_2459787, EPI_ISL_2459788, EPI_ISL_2459789, EPI_ISL_2459791, EPI_ISL_2459792,
EPI_ISL_2459797, EPI_ISL_2459799, EPI_ISL_2459800, EPI_ISL_2459804, EPI_ISL_2459812, EPI_ISL_2459813, EPI_ISL_2459819, EPI_ISL_2459820, EPI_ISL_2459822, EPI_ISL_2459823, EPI_ISL_2459826, EPI_ISL_2459830, EPI_ISL_2459833, EPI_ISL_2459834, EPI_ISL_2459835, EPI_ISL_2459837, EPI_ISL_2459838, EPI_ISL_2459839, EPI_ISL_2459841, EPI_ISL_2459842, EPI_ISL_2459843, EPI_ISL_2459844,
EPI_ISL_2459848, EPI_ISL_2459850, EPI_ISL_2459852, EPI_ISL_2459853, EPI_ISL_2459855, EPI_ISL_2459856, EPI_ISL_2459857, EPI_ISL_2459858, EPI_ISL_2459859, EPI_ISL_2459860, EPI_ISL_2459861, EPI_ISL_2459862, EPI_ISL_2459863, EPI_ISL_2459864, EPI_ISL_2459865, EPI_ISL_2459866, EPI_ISL_2459867, EPI_ISL_2459870, EPI_ISL_2459871, EPI_ISL_2459872, EPI_ISL_2459873, EPI_ISL_2459874,
EPI_ISL_2459876, EPI_ISL_2459877, EPI_ISL_2459878, EPI_ISL_2459879, EPI_ISL_2459880, EPI_ISL_2459883, EPI_ISL_2459884, EPI_ISL_2459885, EPI_ISL_2459886, EPI_ISL_2459887, EPI_ISL_2459888, EPI_ISL_2459889, EPI_ISL_2459890, EPI_ISL_2459891, EPI_ISL_2459892, EPI_ISL_2459893, EPI_ISL_2459894, EPI_ISL_2459895, EPI_ISL_2459896, EPI_ISL_2459897, EPI_ISL_2459898, EPI_ISL_2459899,
EPI_ISL_2459900, EPI_ISL_2459901, EPI_ISL_2459902, EPI_ISL_2459903, EPI_ISL_2459904, EPI_ISL_2459905, EPI_ISL_2459906, EPI_ISL_2459907, EPI_ISL_2459908, EPI_ISL_2459909, EPI_ISL_2459910, EPI_ISL_2459911, EPI_ISL_2459912, EPI_ISL_2459913, EPI_ISL_2459914, EPI_ISL_2459915, EPI_ISL_2459916, EPI_ISL_2459917, EPI_ISL_2459918, EPI_ISL_2459919, EPI_ISL_2459920, EPI_ISL_2459921,
EPI_ISL_2459922, EPI_ISL_2459923, EPI_ISL_2459924, EPI_ISL_2459925, EPI_ISL_2459926, EPI_ISL_2459927, EPI_ISL_2459928, EPI_ISL_2459929, EPI_ISL_2459930, EPI_ISL_2459931, EPI_ISL_2459932, EPI_ISL_2459933, EPI_ISL_2459934, EPI_ISL_2459935, EPI_ISL_2459936, EPI_ISL_2459937, EPI_ISL_2459938, EPI_ISL_2459939, EPI_ISL_2459940, EPI_ISL_2459941, EPI_ISL_2459942, EPI_ISL_2459943,
EPI_ISL_2459944, EPI_ISL_2459945, EPI_ISL_2459946, EPI_ISL_2459947, EPI_ISL_2459948, EPI_ISL_2459949, EPI_ISL_2459950, EPI_ISL_2459951, EPI_ISL_2459952, EPI_ISL_2459953, EPI_ISL_2459954, EPI_ISL_2459955, EPI_ISL_2459956, EPI_ISL_2459957, EPI_ISL_2459958, EPI_ISL_2459959, EPI_ISL_2459960, EPI_ISL_2459961, EPI_ISL_2459962, EPI_ISL_2459963, EPI_ISL_2459964, EPI_ISL_2459965,
EPI_ISL_2459966, EPI_ISL_2459967, EPI_ISL_2459968, EPI_ISL_2459969, EPI_ISL_2459970, EPI_ISL_2459971, EPI_ISL_2459972, EPI_ISL_2459975, EPI_ISL_2459976, EPI_ISL_2459978, EPI_ISL_2459979, EPI_ISL_2459980, EPI_ISL_2459981, EPI_ISL_2459982, EPI_ISL_2459984, EPI_ISL_2459985, EPI_ISL_2459986, EPI_ISL_2459989, EPI_ISL_2459990, EPI_ISL_2459991, EPI_ISL_2459993, EPI_ISL_2459994,
EPI_ISL_2459995, EPI_ISL_2459996, EPI_ISL_2459998, EPI_ISL_2459999, EPI_ISL_2460000, EPI_ISL_2460001, EPI_ISL_2460002, EPI_ISL_2460004, EPI_ISL_2460005, EPI_ISL_2460006, EPI_ISL_2460008, EPI_ISL_2460011, EPI_ISL_2460012, EPI_ISL_2460013, EPI_ISL_2460014, EPI_ISL_2460015, EPI_ISL_2460017, EPI_ISL_2460019, EPI_ISL_2460020, EPI_ISL_2460022, EPI_ISL_2460023, EPI_ISL_2460024,
EPI_ISL_2460025, EPI_ISL_2460026, EPI_ISL_2460027, EPI_ISL_2460028, EPI_ISL_2460029, EPI_ISL_2460032, EPI_ISL_2460033, EPI_ISL_2460034, EPI_ISL_2460035, EPI_ISL_2460036, EPI_ISL_2460037, EPI_ISL_2460038, EPI_ISL_2460051, EPI_ISL_2460053, EPI_ISL_2460054, EPI_ISL_2460055, EPI_ISL_2460056, EPI_ISL_2460057, EPI_ISL_2460058, EPI_ISL_2460059, EPI_ISL_2460060, EPI_ISL_2460061,
EPI_ISL_2460062, EPI_ISL_2460063, EPI_ISL_2460064, EPI_ISL_2460065, EPI_ISL_2460066, EPI_ISL_2460067, EPI_ISL_2460068, EPI_ISL_2460069, EPI_ISL_2460070, EPI_ISL_2460071, EPI_ISL_2460074, EPI_ISL_2460075, EPI_ISL_2460076, EPI_ISL_2460077, EPI_ISL_2460078, EPI_ISL_2460079, EPI_ISL_2460080, EPI_ISL_2460081, EPI_ISL_2460082, EPI_ISL_2460083, EPI_ISL_2460084, EPI_ISL_2460085,
EPI_ISL_2460086, EPI_ISL_2460087, EPI_ISL_2460088, EPI_ISL_2460090, EPI_ISL_2460091, EPI_ISL_2460092, EPI_ISL_2460093, EPI_ISL_2460094, EPI_ISL_2460095, EPI_ISL_2460097, EPI_ISL_2460098, EPI_ISL_2460099, EPI_ISL_2460100, EPI_ISL_2460101, EPI_ISL_2460102, EPI_ISL_2460103, EPI_ISL_2460104, EPI_ISL_2460105, EPI_ISL_2460106, EPI_ISL_2460107, EPI_ISL_2460108, EPI_ISL_2460109,
EPI_ISL_2460110, EPI_ISL_2460111, EPI_ISL_2460112, EPI_ISL_2460113, EPI_ISL_2460114, EPI_ISL_2460115, EPI_ISL_2460117, EPI_ISL_2460118, EPI_ISL_2460119, EPI_ISL_2460120, EPI_ISL_2460121, EPI_ISL_2460122, EPI_ISL_2460123, EPI_ISL_2460124, EPI_ISL_2460125, EPI_ISL_2460126, EPI_ISL_2460127, EPI_ISL_2460128, EPI_ISL_2460129, EPI_ISL_2460130, EPI_ISL_2460131, EPI_ISL_2460132,
EPI_ISL_2460133, EPI_ISL_2460134, EPI_ISL_2460135, EPI_ISL_2460136, EPI_ISL_2460137, EPI_ISL_2460138, EPI_ISL_2460140, EPI_ISL_2460141, EPI_ISL_2460142, EPI_ISL_2460143, EPI_ISL_2460144, EPI_ISL_2460145, EPI_ISL_2460146, EPI_ISL_2460147, EPI_ISL_2460148, EPI_ISL_2460149, EPI_ISL_2460150, EPI_ISL_2460153, EPI_ISL_2460154, EPI_ISL_2460155, EPI_ISL_2460157, EPI_ISL_2460158,
EPI_ISL_2460159, EPI_ISL_2460160, EPI_ISL_2460161, EPI_ISL_2460162, EPI_ISL_2460163, EPI_ISL_2460164, EPI_ISL_2460165, EPI_ISL_2460166, EPI_ISL_2460167, EPI_ISL_2460169, EPI_ISL_2460170, EPI_ISL_2460171, EPI_ISL_2460173, EPI_ISL_2460174, EPI_ISL_2460175, EPI_ISL_2460176, EPI_ISL_2460177, EPI_ISL_2460178, EPI_ISL_2460179, EPI_ISL_2460180, EPI_ISL_2460181, EPI_ISL_2460182,
EPI_ISL_2460197, EPI_ISL_2460198, EPI_ISL_2460199, EPI_ISL_2460200, EPI_ISL_2460201, EPI_ISL_2460202, EPI_ISL_2460203, EPI_ISL_2460204, EPI_ISL_2460205, EPI_ISL_2460206, EPI_ISL_2460207, EPI_ISL_2460208, EPI_ISL_2460209, EPI_ISL_2460210, EPI_ISL_2460211, EPI_ISL_2460212, EPI_ISL_2460213, EPI_ISL_2460214, EPI_ISL_2460215, EPI_ISL_2460216, EPI_ISL_2460217, EPI_ISL_2460218,
EPI_ISL_2460220, EPI_ISL_2460221, EPI_ISL_2460222, EPI_ISL_2460223, EPI_ISL_2460224, EPI_ISL_2460225, EPI_ISL_2460226, EPI_ISL_2460227, EPI_ISL_2460228, EPI_ISL_2460234, EPI_ISL_2460235, EPI_ISL_2460236, EPI_ISL_2460237, EPI_ISL_2460238, EPI_ISL_2460239, EPI_ISL_2460240, EPI_ISL_2460241, EPI_ISL_2460242, EPI_ISL_2460243, EPI_ISL_2460244, EPI_ISL_2460245, EPI_ISL_2460246,
EPI_ISL_2460247, EPI_ISL_2460248, EPI_ISL_2460249, EPI_ISL_2460250, EPI_ISL_2460251, EPI_ISL_2460252, EPI_ISL_2460253, EPI_ISL_2460254, EPI_ISL_2460255, EPI_ISL_2460256, EPI_ISL_2460257, EPI_ISL_2460258, EPI_ISL_2460259, EPI_ISL_2460260, EPI_ISL_2460261, EPI_ISL_2460262, EPI_ISL_2460263, EPI_ISL_2460264, EPI_ISL_2460265, EPI_ISL_2460266, EPI_ISL_2460267, EPI_ISL_2460268,
EPI_ISL_2460269, EPI_ISL_2460270, EPI_ISL_2460271, EPI_ISL_2460272, EPI_ISL_2460273, EPI_ISL_2460274, EPI_ISL_2460275, EPI_ISL_2460276, EPI_ISL_2460277, EPI_ISL_2460278, EPI_ISL_2460279, EPI_ISL_2460280, EPI_ISL_2460281, EPI_ISL_2460282, EPI_ISL_2460283, EPI_ISL_2460284, EPI_ISL_2460285, EPI_ISL_2460286, EPI_ISL_2460287, EPI_ISL_2460288, EPI_ISL_2460289, EPI_ISL_2460290,
EPI_ISL_2460291, EPI_ISL_2460292, EPI_ISL_2460293, EPI_ISL_2460294, EPI_ISL_2460295, EPI_ISL_2460296, EPI_ISL_2460297, EPI_ISL_2460298, EPI_ISL_2460299, EPI_ISL_2460300, EPI_ISL_2460301, EPI_ISL_2460302, EPI_ISL_2460303, EPI_ISL_2460304, EPI_ISL_2460305, EPI_ISL_2460306, EPI_ISL_2460307, EPI_ISL_2460308, EPI_ISL_2460309, EPI_ISL_2460310, EPI_ISL_2460311, EPI_ISL_2460312,
EPI_ISL_2460313, EPI_ISL_2460314, EPI_ISL_2460315, EPI_ISL_2460316, EPI_ISL_2460317, EPI_ISL_2460318, EPI_ISL_2460319, EPI_ISL_2460320, EPI_ISL_2460321, EPI_ISL_2460322, EPI_ISL_2460323, EPI_ISL_2460324, EPI_ISL_2460325, EPI_ISL_2460326, EPI_ISL_2460327, EPI_ISL_2460328, EPI_ISL_2460329, EPI_ISL_2460330, EPI_ISL_2460331, EPI_ISL_2460332, EPI_ISL_2460333, EPI_ISL_2460334,
EPI_ISL_2460335, EPI_ISL_2460336, EPI_ISL_2460337, EPI_ISL_2460338, EPI_ISL_2460339, EPI_ISL_2460340, EPI_ISL_2460341, EPI_ISL_2460342, EPI_ISL_2460343, EPI_ISL_2460344, EPI_ISL_2460345, EPI_ISL_2460346, EPI_ISL_2460347, EPI_ISL_2460348, EPI_ISL_2460349, EPI_ISL_2460350, EPI_ISL_2460351, EPI_ISL_2460352, EPI_ISL_2460353, EPI_ISL_2460355, EPI_ISL_2460356,
EPI_ISL_2460357, EPI_ISL_2460358, EPI_ISL_2460359, EPI_ISL_2460360, EPI_ISL_2460361, EPI_ISL_2460362, EPI_ISL_2460363, EPI_ISL_2460364, EPI_ISL_2460365, EPI_ISL_2460366, EPI_ISL_2460367, EPI_ISL_2460368, EPI_ISL_2460369, EPI_ISL_2460370, EPI_ISL_2460371, EPI_ISL_2460372, EPI_ISL_2460373, EPI_ISL_2460374, EPI_ISL_2460375, EPI_ISL_2460376, EPI_ISL_2460377, EPI_ISL_2460378,
EPI_ISL_2460379, EPI_ISL_2460380, EPI_ISL_2460381, EPI_ISL_2460382, EPI_ISL_2460383, EPI_ISL_2460384, EPI_ISL_2460385, EPI_ISL_2460386, EPI_ISL_2460387, EPI_ISL_2460388, EPI_ISL_2460389, EPI_ISL_2460390, EPI_ISL_2460391, EPI_ISL_2460392, EPI_ISL_2460393, EPI_ISL_2460394, EPI_ISL_2460395, EPI_ISL_2460396, EPI_ISL_2460397, EPI_ISL_2460398, EPI_ISL_2460399, EPI_ISL_2460400,
EPI_ISL_2460402, EPI_ISL_2460403, EPI_ISL_2460404, EPI_ISL_2460405, EPI_ISL_2460406, EPI_ISL_2460407, EPI_ISL_2460408, EPI_ISL_2460409, EPI_ISL_2460410, EPI_ISL_2460411, EPI_ISL_2460412, EPI_ISL_2460413, EPI_ISL_2460414, EPI_ISL_2460415, EPI_ISL_2460416, EPI_ISL_2460417, EPI_ISL_2460418, EPI_ISL_2460419, EPI_ISL_2460420, EPI_ISL_2460421, EPI_ISL_2460422, EPI_ISL_2460423,
EPI_ISL_2460425, EPI_ISL_2460426, EPI_ISL_2460427, EPI_ISL_2460428, EPI_ISL_2460429, EPI_ISL_2460430, EPI_ISL_2460431, EPI_ISL_2460432, EPI_ISL_2460433, EPI_ISL_2460435, EPI_ISL_2460437, EPI_ISL_2460438, EPI_ISL_2460439, EPI_ISL_2460440, EPI_ISL_2460441, EPI_ISL_2460442, EPI_ISL_2460443, EPI_ISL_2460444, EPI_ISL_2460445, EPI_ISL_2460446, EPI_ISL_2460447, EPI_ISL_2460450,
EPI_ISL_2460451, EPI_ISL_2460452, EPI_ISL_2460453, EPI_ISL_2460454, EPI_ISL_2460455, EPI_ISL_2460456, EPI_ISL_2460457, EPI_ISL_2460458, EPI_ISL_2460459, EPI_ISL_2460460, EPI_ISL_2460461, EPI_ISL_2460462, EPI_ISL_2460463, EPI_ISL_2460464, EPI_ISL_2460465, EPI_ISL_2460466, EPI_ISL_2460467, EPI_ISL_2460468, EPI_ISL_2460469, EPI_ISL_2460470, EPI_ISL_2460471, EPI_ISL_2460472,
EPI_ISL_2460473, EPI_ISL_2460475, EPI_ISL_2460476, EPI_ISL_2460477, EPI_ISL_2460478, EPI_ISL_2460479, EPI_ISL_2460480, EPI_ISL_2460481, EPI_ISL_2460482, EPI_ISL_2460483, EPI_ISL_2460484, EPI_ISL_2460485, EPI_ISL_2460486, EPI_ISL_2460487, EPI_ISL_2460488, EPI_ISL_2460489, EPI_ISL_2460490, EPI_ISL_2460491, EPI_ISL_2460492, EPI_ISL_2460493, EPI_ISL_2460494, EPI_ISL_2460495,
EPI_ISL_2460496, EPI_ISL_2460498, EPI_ISL_2460499, EPI_ISL_2460500, EPI_ISL_2460501, EPI_ISL_2460502, EPI_ISL_2460503, EPI_ISL_2460504, EPI_ISL_2460505, EPI_ISL_2460506, EPI_ISL_2460507, EPI_ISL_2460508, EPI_ISL_2460509, EPI_ISL_2460510, EPI_ISL_2460511, EPI_ISL_2460512, EPI_ISL_2460513, EPI_ISL_2460514, EPI_ISL_2460515, EPI_ISL_2460516, EPI_ISL_2460517, EPI_ISL_2460518,
EPI_ISL_2460519, EPI_ISL_2460520, EPI_ISL_2460521, EPI_ISL_2460522, EPI_ISL_2460523, EPI_ISL_2460524, EPI_ISL_2460525, EPI_ISL_2460526, EPI_ISL_2460527, EPI_ISL_2460528, EPI_ISL_2460529, EPI_ISL_2460530, EPI_ISL_2460531, EPI_ISL_2460532, EPI_ISL_2460533, EPI_ISL_2460534, EPI_ISL_2460535, EPI_ISL_2460536, EPI_ISL_2460537, EPI_ISL_2460538, EPI_ISL_2460539, EPI_ISL_2460540,
EPI_ISL_2460541, EPI_ISL_2460542, EPI_ISL_2460543, EPI_ISL_2460544, EPI_ISL_2460545, EPI_ISL_2460546, EPI_ISL_2460547, EPI_ISL_2460548, EPI_ISL_2460549, EPI_ISL_2460550, EPI_ISL_2460551, EPI_ISL_2460552, EPI_ISL_2460553, EPI_ISL_2460554, EPI_ISL_2460555, EPI_ISL_2460556, EPI_ISL_2460557, EPI_ISL_2460558, EPI_ISL_2460559, EPI_ISL_2460560, EPI_ISL_2460561, EPI_ISL_2460562,
EPI_ISL_2460563, EPI_ISL_2460564, EPI_ISL_2460565, EPI_ISL_2460566, EPI_ISL_2460567, EPI_ISL_2460568, EPI_ISL_2460569, EPI_ISL_2460570, EPI_ISL_2460571, EPI_ISL_2460572, EPI_ISL_2460573, EPI_ISL_2460574, EPI_ISL_2460575, EPI_ISL_2460576, EPI_ISL_2460577, EPI_ISL_2460578, EPI_ISL_2460580, EPI_ISL_2460582, EPI_ISL_2460583, EPI_ISL_2460584, EPI_ISL_2460585, EPI_ISL_2460586,
EPI_ISL_2460587, EPI_ISL_2460588, EPI_ISL_2460589, EPI_ISL_2460590, EPI_ISL_2460591, EPI_ISL_2460592, EPI_ISL_2460593, EPI_ISL_2460594, EPI_ISL_2460595, EPI_ISL_2460596, EPI_ISL_2460597, EPI_ISL_2460598, EPI_ISL_2460599, EPI_ISL_2460600, EPI_ISL_2460601, EPI_ISL_2460602, EPI_ISL_2460603, EPI_ISL_2460604, EPI_ISL_2460605, EPI_ISL_2460606, EPI_ISL_2460607, EPI_ISL_2460608,
EPI_ISL_2460609, EPI_ISL_2460611, EPI_ISL_2460614, EPI_ISL_2460616, EPI_ISL_2460617, EPI_ISL_2460618, EPI_ISL_2460622, EPI_ISL_2460623, EPI_ISL_2460625, EPI_ISL_2460626, EPI_ISL_2460627, EPI_ISL_2460629, EPI_ISL_2460633, EPI_ISL_2460636, EPI_ISL_2460637, EPI_ISL_2460640, EPI_ISL_2460641, EPI_ISL_2460642, EPI_ISL_2460643, EPI_ISL_2460645, EPI_ISL_2460646,
EPI_ISL_2460647, EPI_ISL_2460648, EPI_ISL_2460649, EPI_ISL_2460650, EPI_ISL_2460651, EPI_ISL_2460652, EPI_ISL_2460653, EPI_ISL_2460654, EPI_ISL_2460655, EPI_ISL_2460656, EPI_ISL_2460657, EPI_ISL_2460658, EPI_ISL_2460670, EPI_ISL_2460681, EPI_ISL_2460682, EPI_ISL_2460683, EPI_ISL_2460684, EPI_ISL_2460685, EPI_ISL_2460686, EPI_ISL_2460697, EPI_ISL_2460698, EPI_ISL_2460699,
EPI_ISL_2460700, EPI_ISL_2460701, EPI_ISL_2460702, EPI_ISL_2460703, EPI_ISL_2460704, EPI_ISL_2460705, EPI_ISL_2460706, EPI_ISL_2460707, EPI_ISL_2460708, EPI_ISL_2460709, EPI_ISL_2460710, EPI_ISL_2460711, EPI_ISL_2460712, EPI_ISL_2460713, EPI_ISL_2460714, EPI_ISL_2460715, EPI_ISL_2460716, EPI_ISL_2460717, EPI_ISL_2460718, EPI_ISL_2460719, EPI_ISL_2460720, EPI_ISL_2460721,
EPI_ISL_2460722, EPI_ISL_2460723, EPI_ISL_2460724, EPI_ISL_2460725, EPI_ISL_2460726, EPI_ISL_2460727, EPI_ISL_2460728, EPI_ISL_2460729, EPI_ISL_2460730, EPI_ISL_2460731, EPI_ISL_2460732, EPI_ISL_2460733, EPI_ISL_2460734, EPI_ISL_2460735, EPI_ISL_2460736, EPI_ISL_2460737, EPI_ISL_2460738, EPI_ISL_2460739, EPI_ISL_2460740, EPI_ISL_2460741, EPI_ISL_2460742, EPI_ISL_2460743,
EPI_ISL_2460744, EPI_ISL_2460745, EPI_ISL_2460746, EPI_ISL_2460747, EPI_ISL_2460748, EPI_ISL_2460749, EPI_ISL_2460750, EPI_ISL_2460751, EPI_ISL_2460752, EPI_ISL_2460753, EPI_ISL_2460754, EPI_ISL_2460755, EPI_ISL_2460756, EPI_ISL_2460757, EPI_ISL_2460758, EPI_ISL_2460759, EPI_ISL_2460760, EPI_ISL_2460761, EPI_ISL_2460763, EPI_ISL_2460764, EPI_ISL_2460765,
EPI_ISL_2460766, EPI_ISL_2460767, EPI_ISL_2460768, EPI_ISL_2460769, EPI_ISL_2460770, EPI_ISL_2460771, EPI_ISL_2460774, EPI_ISL_2460775, EPI_ISL_2460776, EPI_ISL_2460777, EPI_ISL_2460778, EPI_ISL_2460779, EPI_ISL_2460780, EPI_ISL_2460781, EPI_ISL_2460782, EPI_ISL_2460783, EPI_ISL_2460784, EPI_ISL_2460785, EPI_ISL_2460786, EPI_ISL_2460787, EPI_ISL_2460788, EPI_ISL_2460789,
EPI_ISL_2460790, EPI_ISL_2460791, EPI_ISL_2460793, EPI_ISL_2460794, EPI_ISL_2460795, EPI_ISL_2460796, EPI_ISL_2460797, EPI_ISL_2460798, EPI_ISL_2460799, EPI_ISL_2460800, EPI_ISL_2460801, EPI_ISL_2460802, EPI_ISL_2460803, EPI_ISL_2460804, EPI_ISL_2460805, EPI_ISL_2460806, EPI_ISL_2460807, EPI_ISL_2460808, EPI_ISL_2460809, EPI_ISL_2460810, EPI_ISL_2460811,
EPI_ISL_2460812, EPI_ISL_2460813, EPI_ISL_2460814, EPI_ISL_2460815, EPI_ISL_2460816, EPI_ISL_2460817, EPI_ISL_2460818, EPI_ISL_2460819, EPI_ISL_2460820, EPI_ISL_2460821, EPI_ISL_2460822, EPI_ISL_2460823, EPI_ISL_2460824, EPI_ISL_2460825, EPI_ISL_2460826, EPI_ISL_2460827, EPI_ISL_2460828, EPI_ISL_2460829, EPI_ISL_2460830, EPI_ISL_2460831, EPI_ISL_2460832, EPI_ISL_2460833,
EPI_ISL_2460834, EPI_ISL_2460835, EPI_ISL_2460836, EPI_ISL_2460837, EPI_ISL_2460838, EPI_ISL_2460839, EPI_ISL_2460840, EPI_ISL_2460841, EPI_ISL_2460842, EPI_ISL_2460843, EPI_ISL_2460844, EPI_ISL_2460845, EPI_ISL_2460846, EPI_ISL_2460847, EPI_ISL_2460848, EPI_ISL_2460849, EPI_ISL_2460850, EPI_ISL_2460851, EPI_ISL_2460852, EPI_ISL_2460853, EPI_ISL_2460854, EPI_ISL_2460855,
EPI_ISL_2460856, EPI_ISL_2460857, EPI_ISL_2460858, EPI_ISL_2460859, EPI_ISL_2460860, EPI_ISL_2460861, EPI_ISL_2460862, EPI_ISL_2460863, EPI_ISL_2460864, EPI_ISL_2460865, EPI_ISL_2460866, EPI_ISL_2460867, EPI_ISL_2460868, EPI_ISL_2460869, EPI_ISL_2460870, EPI_ISL_2460871, EPI_ISL_2460872, EPI_ISL_2460873, EPI_ISL_2460874, EPI_ISL_2460875, EPI_ISL_2460876, EPI_ISL_2460877,
EPI_ISL_2460878, EPI_ISL_2460879, EPI_ISL_2460880, EPI_ISL_2460882, EPI_ISL_2460883, EPI_ISL_2460884, EPI_ISL_2460885, EPI_ISL_2460886, EPI_ISL_2460887, EPI_ISL_2460888, EPI_ISL_2460889, EPI_ISL_2460890, EPI_ISL_2460891, EPI_ISL_2460892, EPI_ISL_2460893, EPI_ISL_2460894, EPI_ISL_2460895, EPI_ISL_2460896, EPI_ISL_2460897, EPI_ISL_2460898, EPI_ISL_2460899, EPI_ISL_2460900,
EPI_ISL_2460901, EPI_ISL_2460902, EPI_ISL_2460904, EPI_ISL_2460905, EPI_ISL_2460906, EPI_ISL_2460907, EPI_ISL_2460908, EPI_ISL_2460909, EPI_ISL_2460910, EPI_ISL_2460911, EPI_ISL_2460912, EPI_ISL_2460913, EPI_ISL_2460914, EPI_ISL_2460915, EPI_ISL_2460916, EPI_ISL_2460917, EPI_ISL_2460918, EPI_ISL_2460919, EPI_ISL_2460920, EPI_ISL_2460921, EPI_ISL_2460922,
EPI_ISL_2460923, EPI_ISL_2460924, EPI_ISL_2460925, EPI_ISL_2460926, EPI_ISL_2460927, EPI_ISL_2460928, EPI_ISL_2460929, EPI_ISL_2460930, EPI_ISL_2460931, EPI_ISL_2460932, EPI_ISL_2460933, EPI_ISL_2460934, EPI_ISL_2460935, EPI_ISL_2460936, EPI_ISL_2460937, EPI_ISL_2460938, EPI_ISL_2460939, EPI_ISL_2460940, EPI_ISL_2460941, EPI_ISL_2460942, EPI_ISL_2460943, EPI_ISL_2460944,
EPI_ISL_2460945, EPI_ISL_2460946, EPI_ISL_2460947, EPI_ISL_2460948, EPI_ISL_2460949, EPI_ISL_2460950, EPI_ISL_2460951, EPI_ISL_2460952, EPI_ISL_2460953, EPI_ISL_2460954, EPI_ISL_2460955, EPI_ISL_2460956, EPI_ISL_2460957, EPI_ISL_2460958, EPI_ISL_2460960, EPI_ISL_2460961, EPI_ISL_2460962, EPI_ISL_2460963, EPI_ISL_2460964, EPI_ISL_2460965, EPI_ISL_2460966,
EPI_ISL_2460967, EPI_ISL_2460968, EPI_ISL_2460969, EPI_ISL_2460970, EPI_ISL_2460971, EPI_ISL_2460972, EPI_ISL_2460973, EPI_ISL_2460974, EPI_ISL_2460975, EPI_ISL_2460976, EPI_ISL_2460977, EPI_ISL_2460978, EPI_ISL_2460979, EPI_ISL_2460980, EPI_ISL_2460981, EPI_ISL_2460982, EPI_ISL_2460983, EPI_ISL_2460984, EPI_ISL_2460985, EPI_ISL_2460986, EPI_ISL_2460987, EPI_ISL_2460988,
EPI_ISL_2460989, EPI_ISL_2460990, EPI_ISL_2460991, EPI_ISL_2460992, EPI_ISL_2460993, EPI_ISL_2460994, EPI_ISL_2460995, EPI_ISL_2460996, EPI_ISL_2460997, EPI_ISL_2460998, EPI_ISL_2460999, EPI_ISL_2461000, EPI_ISL_2461001, EPI_ISL_2461002, EPI_ISL_2461003, EPI_ISL_2461004, EPI_ISL_2461005, EPI_ISL_2461006, EPI_ISL_2461007, EPI_ISL_2461008, EPI_ISL_2461009, EPI_ISL_2461010,
EPI_ISL_2461011, EPI_ISL_2461012, EPI_ISL_2461013, EPI_ISL_2461014, EPI_ISL_2461015, EPI_ISL_2461016, EPI_ISL_2461017, EPI_ISL_2461018, EPI_ISL_2461019, EPI_ISL_2461020, EPI_ISL_2461021, EPI_ISL_2461022, EPI_ISL_2461023, EPI_ISL_2461024, EPI_ISL_2461025, EPI_ISL_2461026, EPI_ISL_2461027, EPI_ISL_2461028, EPI_ISL_2461029, EPI_ISL_2461030, EPI_ISL_2461031, EPI_ISL_2461032,
EPI_ISL_2461033, EPI_ISL_2461034, EPI_ISL_2461035, EPI_ISL_2461036, EPI_ISL_2461037, EPI_ISL_2461038, EPI_ISL_2461039, EPI_ISL_2461040, EPI_ISL_2461041, EPI_ISL_2461042, EPI_ISL_2461043, EPI_ISL_2461044, EPI_ISL_2461045, EPI_ISL_2461046, EPI_ISL_2461047, EPI_ISL_2461048, EPI_ISL_2461049, EPI_ISL_2461050, EPI_ISL_2461051, EPI_ISL_2461053, EPI_ISL_2461054,
EPI_ISL_2461055, EPI_ISL_2461056, EPI_ISL_2461057, EPI_ISL_2461058, EPI_ISL_2461059, EPI_ISL_2461060, EPI_ISL_2461061, EPI_ISL_2461062, EPI_ISL_2461063, EPI_ISL_2461064, EPI_ISL_2461065, EPI_ISL_2461066, EPI_ISL_2461067, EPI_ISL_2461068, EPI_ISL_2461069, EPI_ISL_2461070, EPI_ISL_2461071, EPI_ISL_2461072, EPI_ISL_2461073, EPI_ISL_2461074, EPI_ISL_2461075, EPI_ISL_2461076,
EPI_ISL_2461077, EPI_ISL_2461078, EPI_ISL_2461079, EPI_ISL_2461080, EPI_ISL_2461081, EPI_ISL_2461082, EPI_ISL_2461083, EPI_ISL_2461084, EPI_ISL_2461085, EPI_ISL_2461086, EPI_ISL_2461087, EPI_ISL_2461088, EPI_ISL_2461089, EPI_ISL_2461090, EPI_ISL_2461091, EPI_ISL_2461092, EPI_ISL_2461093, EPI_ISL_2461094, EPI_ISL_2461095, EPI_ISL_2461096, EPI_ISL_2461097, EPI_ISL_2461098,
EPI_ISL_2461099, EPI_ISL_2461100, EPI_ISL_2461101, EPI_ISL_2461102, EPI_ISL_2461103, EPI_ISL_2461104, EPI_ISL_2461105, EPI_ISL_2461106, EPI_ISL_2461107, EPI_ISL_2461108, EPI_ISL_2461109, EPI_ISL_2461110, EPI_ISL_2461111, EPI_ISL_2461112, EPI_ISL_2461113, EPI_ISL_2461114, EPI_ISL_2461115, EPI_ISL_2461116, EPI_ISL_2461117, EPI_ISL_2461118, EPI_ISL_2461119, EPI_ISL_2461120,
EPI_ISL_2461121, EPI_ISL_2461122, EPI_ISL_2461123, EPI_ISL_2461124, EPI_ISL_2461125, EPI_ISL_2461126, EPI_ISL_2461127, EPI_ISL_2461128, EPI_ISL_2461129, EPI_ISL_2461130, EPI_ISL_2461132, EPI_ISL_2461133, EPI_ISL_2461134, EPI_ISL_2461135, EPI_ISL_2461136, EPI_ISL_2461137, EPI_ISL_2461138, EPI_ISL_2461139, EPI_ISL_2461140, EPI_ISL_2461141, EPI_ISL_2461142, EPI_ISL_2461143,
EPI_ISL_2461144, EPI_ISL_2461145, EPI_ISL_2461146, EPI_ISL_2461147, EPI_ISL_2461148, EPI_ISL_2461149, EPI_ISL_2461150, EPI_ISL_2461151, EPI_ISL_2461152, EPI_ISL_2461153, EPI_ISL_2461154, EPI_ISL_2461155, EPI_ISL_2461156, EPI_ISL_2461157, EPI_ISL_2461158, EPI_ISL_2461159, EPI_ISL_2461160, EPI_ISL_2461161, EPI_ISL_2461162, EPI_ISL_2461163, EPI_ISL_2461164, EPI_ISL_2461165,
EPI_ISL_2461166, EPI_ISL_2461167, EPI_ISL_2461168, EPI_ISL_2461169, EPI_ISL_2461170, EPI_ISL_2461171, EPI_ISL_2461172, EPI_ISL_2461173, EPI_ISL_2461174, EPI_ISL_2461175, EPI_ISL_2461176, EPI_ISL_2461177, EPI_ISL_2461178, EPI_ISL_2461179, EPI_ISL_2461180, EPI_ISL_2461181, EPI_ISL_2461182, EPI_ISL_2461183, EPI_ISL_2461184, EPI_ISL_2461185, EPI_ISL_2461186, EPI_ISL_2461187,
EPI_ISL_2461188, EPI_ISL_2461189, EPI_ISL_2461190, EPI_ISL_2461191, EPI_ISL_2461192, EPI_ISL_2461193, EPI_ISL_2461194, EPI_ISL_2461195, EPI_ISL_2461196, EPI_ISL_2461197, EPI_ISL_2461198, EPI_ISL_2461199, EPI_ISL_2461200, EPI_ISL_2461201, EPI_ISL_2461203, EPI_ISL_2461204, EPI_ISL_2461205, EPI_ISL_2461206, EPI_ISL_2461207, EPI_ISL_2461208, EPI_ISL_2461209, EPI_ISL_2461210,
EPI_ISL_2461211, EPI_ISL_2461212, EPI_ISL_2461213, EPI_ISL_2461214, EPI_ISL_2461215, EPI_ISL_2461216, EPI_ISL_2461217, EPI_ISL_2461218, EPI_ISL_2461219, EPI_ISL_2461220, EPI_ISL_2461221, EPI_ISL_2461222, EPI_ISL_2461223, EPI_ISL_2461224, EPI_ISL_2461225, EPI_ISL_2461226, EPI_ISL_2461227, EPI_ISL_2461228, EPI_ISL_2461229, EPI_ISL_2461230, EPI_ISL_2461231, EPI_ISL_2461232,
EPI_ISL_2461233, EPI_ISL_2461234, EPI_ISL_2461235, EPI_ISL_2461236, EPI_ISL_2461237, EPI_ISL_2461238, EPI_ISL_2461239, EPI_ISL_2461240, EPI_ISL_2461241, EPI_ISL_2461242, EPI_ISL_2461243, EPI_ISL_2461244, EPI_ISL_2461245, EPI_ISL_2461246, EPI_ISL_2461247, EPI_ISL_2461248, EPI_ISL_2461249, EPI_ISL_2461250, EPI_ISL_2461251, EPI_ISL_2461252, EPI_ISL_2461253, EPI_ISL_2461254,
EPI_ISL_2461255, EPI_ISL_2461256, EPI_ISL_2461257, EPI_ISL_2461259, EPI_ISL_2461260, EPI_ISL_2461261, EPI_ISL_2461262, EPI_ISL_2461263, EPI_ISL_2461264, EPI_ISL_2461265, EPI_ISL_2461266, EPI_ISL_2461267, EPI_ISL_2461268, EPI_ISL_2461269, EPI_ISL_2461270, EPI_ISL_2461271, EPI_ISL_2461272, EPI_ISL_2461273, EPI_ISL_2461274, EPI_ISL_2461275, EPI_ISL_2461276, EPI_ISL_2461277,
EPI_ISL_2461278, EPI_ISL_2461279, EPI_ISL_2461280, EPI_ISL_2461281, EPI_ISL_2461282, EPI_ISL_2461283, EPI_ISL_2461284, EPI_ISL_2461285, EPI_ISL_2461286, EPI_ISL_2461287, EPI_ISL_2461288, EPI_ISL_2461289, EPI_ISL_2461290, EPI_ISL_2461291, EPI_ISL_2461292, EPI_ISL_2461293, EPI_ISL_2461294, EPI_ISL_2461295, EPI_ISL_2461296, EPI_ISL_2461297, EPI_ISL_2461298, EPI_ISL_2461299,
EPI_ISL_2461300, EPI_ISL_2461301, EPI_ISL_2461302, EPI_ISL_2461303, EPI_ISL_2461304, EPI_ISL_2461305, EPI_ISL_2461306, EPI_ISL_2461307, EPI_ISL_2461308, EPI_ISL_2461309, EPI_ISL_2461310, EPI_ISL_2461311, EPI_ISL_2461312, EPI_ISL_2461313, EPI_ISL_2461314, EPI_ISL_2461315, EPI_ISL_2461316, EPI_ISL_2461317, EPI_ISL_2461318, EPI_ISL_2461319, EPI_ISL_2461320, EPI_ISL_2461321,
EPI_ISL_2461322, EPI_ISL_2461323, EPI_ISL_2461324, EPI_ISL_2461325, EPI_ISL_2461326, EPI_ISL_2461327, EPI_ISL_2461328, EPI_ISL_2461329, EPI_ISL_2461330, EPI_ISL_2461331, EPI_ISL_2461332, EPI_ISL_2461333, EPI_ISL_2461334, EPI_ISL_2461335, EPI_ISL_2461336, EPI_ISL_2461337, EPI_ISL_2461338, EPI_ISL_2461339, EPI_ISL_2461340, EPI_ISL_2461341, EPI_ISL_2461342, EPI_ISL_2461343,
EPI_ISL_2461344, EPI_ISL_2461345, EPI_ISL_2461346, EPI_ISL_2461347, EPI_ISL_2461348, EPI_ISL_2461349, EPI_ISL_2461350, EPI_ISL_2461351, EPI_ISL_2461352, EPI_ISL_2461353, EPI_ISL_2461354, EPI_ISL_2461355, EPI_ISL_2461357, EPI_ISL_2461358, EPI_ISL_2461359, EPI_ISL_2461360, EPI_ISL_2461361, EPI_ISL_2461362, EPI_ISL_2461363, EPI_ISL_2461364, EPI_ISL_2461365,
EPI_ISL_2461366, EPI_ISL_2461367, EPI_ISL_2461368, EPI_ISL_2461369, EPI_ISL_2461370, EPI_ISL_2461371, EPI_ISL_2461372, EPI_ISL_2461373, EPI_ISL_2461374, EPI_ISL_2461375, EPI_ISL_2461376, EPI_ISL_2461377, EPI_ISL_2461378, EPI_ISL_2461379, EPI_ISL_2461380, EPI_ISL_2461381, EPI_ISL_2461382, EPI_ISL_2461383, EPI_ISL_2461384, EPI_ISL_2461385, EPI_ISL_2461386, EPI_ISL_2461387,
EPI_ISL_2461388, EPI_ISL_2461389, EPI_ISL_2461390, EPI_ISL_2461391, EPI_ISL_2461392, EPI_ISL_2461393, EPI_ISL_2461394, EPI_ISL_2461395, EPI_ISL_2461396, EPI_ISL_2461397, EPI_ISL_2461398, EPI_ISL_2461399, EPI_ISL_2461400, EPI_ISL_2461401, EPI_ISL_2461402, EPI_ISL_2461403, EPI_ISL_2461404, EPI_ISL_2461405, EPI_ISL_2461406, EPI_ISL_2461407, EPI_ISL_2461408, EPI_ISL_2461409,
EPI_ISL_2461410, EPI_ISL_2461411, EPI_ISL_2461412, EPI_ISL_2461414, EPI_ISL_2461415, EPI_ISL_2461416, EPI_ISL_2461417, EPI_ISL_2461418, EPI_ISL_2461419, EPI_ISL_2461420, EPI_ISL_2461421, EPI_ISL_2461422, EPI_ISL_2461423, EPI_ISL_2461424, EPI_ISL_2461425, EPI_ISL_2461426, EPI_ISL_2461427, EPI_ISL_2461428, EPI_ISL_2461429, EPI_ISL_2461430, EPI_ISL_2461431, EPI_ISL_2461432,
EPI_ISL_2461433, EPI_ISL_2461434, EPI_ISL_2461435, EPI_ISL_2461436, EPI_ISL_2461437, EPI_ISL_2461438, EPI_ISL_2461439, EPI_ISL_2461440, EPI_ISL_2461441, EPI_ISL_2461442, EPI_ISL_2461443, EPI_ISL_2461444, EPI_ISL_2461445, EPI_ISL_2461446, EPI_ISL_2461447, EPI_ISL_2461448, EPI_ISL_2461449, EPI_ISL_2461450, EPI_ISL_2461451, EPI_ISL_2461452, EPI_ISL_2461453, EPI_ISL_2461454,
EPI_ISL_2461455, EPI_ISL_2461456, EPI_ISL_2461457, EPI_ISL_2461458, EPI_ISL_2461459, EPI_ISL_2461460, EPI_ISL_2461461, EPI_ISL_2461462, EPI_ISL_2461463, EPI_ISL_2461464, EPI_ISL_2461465, EPI_ISL_2461466, EPI_ISL_2461467, EPI_ISL_2461468, EPI_ISL_2461469, EPI_ISL_2461470, EPI_ISL_2461471, EPI_ISL_2461472, EPI_ISL_2461473, EPI_ISL_2461474, EPI_ISL_2461475, EPI_ISL_2461476,
EPI_ISL_2461477, EPI_ISL_2461478, EPI_ISL_2461479, EPI_ISL_2461480, EPI_ISL_2461481, EPI_ISL_2461482, EPI_ISL_2461483, EPI_ISL_2461484, EPI_ISL_2461485, EPI_ISL_2461486, EPI_ISL_2461487, EPI_ISL_2461488, EPI_ISL_2461489, EPI_ISL_2461490, EPI_ISL_2461491, EPI_ISL_2461492, EPI_ISL_2461493, EPI_ISL_2461494, EPI_ISL_2461495, EPI_ISL_2461496, EPI_ISL_2461497, EPI_ISL_2461498,
EPI_ISL_2461499, EPI_ISL_2461500, EPI_ISL_2461501, EPI_ISL_2461502, EPI_ISL_2461509, EPI_ISL_2461510, EPI_ISL_2461511, EPI_ISL_2461512, EPI_ISL_2461513, EPI_ISL_2461514, EPI_ISL_2461515, EPI_ISL_2461516, EPI_ISL_2461517, EPI_ISL_2461518, EPI_ISL_2461519, EPI_ISL_2461520, EPI_ISL_2461521, EPI_ISL_2461522, EPI_ISL_2461523, EPI_ISL_2461524, EPI_ISL_2461525, EPI_ISL_2461526,
EPI_ISL_2461527, EPI_ISL_2461528, EPI_ISL_2461530, EPI_ISL_2461532, EPI_ISL_2461534, EPI_ISL_2461535, EPI_ISL_2461536, EPI_ISL_2461537, EPI_ISL_2461538, EPI_ISL_2461539, EPI_ISL_2461540, EPI_ISL_2461541, EPI_ISL_2461542, EPI_ISL_2461543, EPI_ISL_2461544, EPI_ISL_2461545, EPI_ISL_2461546, EPI_ISL_2461547, EPI_ISL_2461548, EPI_ISL_2461549,
EPI_ISL_2461550, EPI_ISL_2461551, EPI_ISL_2461552, EPI_ISL_2461553, EPI_ISL_2461554, EPI_ISL_2461555, EPI_ISL_2461556, EPI_ISL_2461557, EPI_ISL_2461558, EPI_ISL_2461559, EPI_ISL_2461560, EPI_ISL_2461561, EPI_ISL_2461562, EPI_ISL_2461563, EPI_ISL_2461564, EPI_ISL_2461565, EPI_ISL_2461566, EPI_ISL_2461567, EPI_ISL_2461568, EPI_ISL_2461569, EPI_ISL_2461570, EPI_ISL_2461571,
EPI_ISL_2461572, EPI_ISL_2461573, EPI_ISL_2461574, EPI_ISL_2461575, EPI_ISL_2461576, EPI_ISL_2461577, EPI_ISL_2461578, EPI_ISL_2461579, EPI_ISL_2461580, EPI_ISL_2461581, EPI_ISL_2461582, EPI_ISL_2461583, EPI_ISL_2461585, EPI_ISL_2461586, EPI_ISL_2461587, EPI_ISL_2461588, EPI_ISL_2461589, EPI_ISL_2461590, EPI_ISL_2461591, EPI_ISL_2461592, EPI_ISL_2461593,
EPI_ISL_2461594, EPI_ISL_2461595, EPI_ISL_2461596, EPI_ISL_2461597, EPI_ISL_2461598, EPI_ISL_2461599, EPI_ISL_2461600, EPI_ISL_2461601, EPI_ISL_2461602, EPI_ISL_2461603, EPI_ISL_2461604, EPI_ISL_2461605, EPI_ISL_2461606, EPI_ISL_2461607, EPI_ISL_2461608, EPI_ISL_2461609, EPI_ISL_2461610, EPI_ISL_2461611, EPI_ISL_2461612, EPI_ISL_2461613, EPI_ISL_2461614, EPI_ISL_2461615,
EPI_ISL_2461616, EPI_ISL_2461617, EPI_ISL_2461618, EPI_ISL_2461619, EPI_ISL_2461620, EPI_ISL_2461621, EPI_ISL_2461622, EPI_ISL_2461624, EPI_ISL_2461625, EPI_ISL_2461626, EPI_ISL_2461627, EPI_ISL_2461628, EPI_ISL_2461629, EPI_ISL_2461630, EPI_ISL_2461631, EPI_ISL_2461632, EPI_ISL_2461633, EPI_ISL_2461634, EPI_ISL_2461635, EPI_ISL_2461636, EPI_ISL_2461637, EPI_ISL_2461638,
EPI_ISL_2461639, EPI_ISL_2461640, EPI_ISL_2461641, EPI_ISL_2461642, EPI_ISL_2461643, EPI_ISL_2461644, EPI_ISL_2461645, EPI_ISL_2461646, EPI_ISL_2461647, EPI_ISL_2461648, EPI_ISL_2461650, EPI_ISL_2461651, EPI_ISL_2461652, EPI_ISL_2461653, EPI_ISL_2461654, EPI_ISL_2461655, EPI_ISL_2461656, EPI_ISL_2461657, EPI_ISL_2461658, EPI_ISL_2461659, EPI_ISL_2461660,
EPI_ISL_2461661, EPI_ISL_2461662, EPI_ISL_2461663, EPI_ISL_2461664, EPI_ISL_2461665, EPI_ISL_2461666, EPI_ISL_2461667, EPI_ISL_2461668, EPI_ISL_2461669, EPI_ISL_2461670, EPI_ISL_2461671, EPI_ISL_2461672, EPI_ISL_2461673, EPI_ISL_2461674, EPI_ISL_2461675, EPI_ISL_2461676, EPI_ISL_2461677, EPI_ISL_2461678, EPI_ISL_2461679, EPI_ISL_2461680, EPI_ISL_2461681, EPI_ISL_2461682,
EPI_ISL_2461683, EPI_ISL_2461684, EPI_ISL_2461685, EPI_ISL_2461686, EPI_ISL_2461687, EPI_ISL_2461688, EPI_ISL_2461689, EPI_ISL_2461690, EPI_ISL_2461691, EPI_ISL_2461692, EPI_ISL_2461693, EPI_ISL_2461694, EPI_ISL_2461695, EPI_ISL_2461696, EPI_ISL_2461697, EPI_ISL_2461698, EPI_ISL_2461699, EPI_ISL_2461700, EPI_ISL_2461701, EPI_ISL_2461702, EPI_ISL_2461703, EPI_ISL_2461704,
EPI_ISL_2461705, EPI_ISL_2461706, EPI_ISL_2461707, EPI_ISL_2461708, EPI_ISL_2461709, EPI_ISL_2461710, EPI_ISL_2461711, EPI_ISL_2461712, EPI_ISL_2461713, EPI_ISL_2461714, EPI_ISL_2461715, EPI_ISL_2461716, EPI_ISL_2461717, EPI_ISL_2461718, EPI_ISL_2461719, EPI_ISL_2461720, EPI_ISL_2461721, EPI_ISL_2461722, EPI_ISL_2461723, EPI_ISL_2461729, EPI_ISL_2461730,
EPI_ISL_2461731, EPI_ISL_2461732, EPI_ISL_2461733, EPI_ISL_2461734, EPI_ISL_2461735, EPI_ISL_2461736, EPI_ISL_2461737, EPI_ISL_2461738, EPI_ISL_2461739, EPI_ISL_2461740, EPI_ISL_2461741, EPI_ISL_2461742, EPI_ISL_2461743, EPI_ISL_2461744, EPI_ISL_2461745, EPI_ISL_2461746, EPI_ISL_2461747, EPI_ISL_2461748, EPI_ISL_2461749, EPI_ISL_2461750, EPI_ISL_2461751, EPI_ISL_2461752,
EPI_ISL_2461753, EPI_ISL_2461754, EPI_ISL_2461755, EPI_ISL_2461756, EPI_ISL_2461757, EPI_ISL_2461758, EPI_ISL_2461759, EPI_ISL_2461760, EPI_ISL_2461761, EPI_ISL_2461763, EPI_ISL_2461764, EPI_ISL_2461765, EPI_ISL_2461766, EPI_ISL_2461767, EPI_ISL_2461768, EPI_ISL_2461769, EPI_ISL_2461770, EPI_ISL_2461771, EPI_ISL_2461772, EPI_ISL_2461773, EPI_ISL_2461774, EPI_ISL_2461775,
EPI_ISL_2461778, EPI_ISL_2461779, EPI_ISL_2461780, EPI_ISL_2461781, EPI_ISL_2461782, EPI_ISL_2461783, EPI_ISL_2461784, EPI_ISL_2461785, EPI_ISL_2461786, EPI_ISL_2461787, EPI_ISL_2461788, EPI_ISL_2461789, EPI_ISL_2461791, EPI_ISL_2461792, EPI_ISL_2461793, EPI_ISL_2461794, EPI_ISL_2461795, EPI_ISL_2461796, EPI_ISL_2461797, EPI_ISL_2461798, EPI_ISL_2461799, EPI_ISL_2461800,
EPI_ISL_2461801, EPI_ISL_2461802, EPI_ISL_2461803, EPI_ISL_2461804, EPI_ISL_2461805, EPI_ISL_2461806, EPI_ISL_2461807, EPI_ISL_2461808, EPI_ISL_2461809, EPI_ISL_2461810, EPI_ISL_2461811, EPI_ISL_2461812, EPI_ISL_2461813, EPI_ISL_2461814, EPI_ISL_2461815, EPI_ISL_2461816, EPI_ISL_2461817, EPI_ISL_2461818, EPI_ISL_2461819, EPI_ISL_2461820, EPI_ISL_2461821, EPI_ISL_2461822,
EPI_ISL_2461823, EPI_ISL_2461824, EPI_ISL_2461825, EPI_ISL_2461826, EPI_ISL_2461827, EPI_ISL_2461828, EPI_ISL_2461829, EPI_ISL_2461830, EPI_ISL_2461831, EPI_ISL_2461832, EPI_ISL_2461833, EPI_ISL_2461834, EPI_ISL_2461835, EPI_ISL_2461836, EPI_ISL_2461837, EPI_ISL_2461838, EPI_ISL_2461839, EPI_ISL_2461840, EPI_ISL_2461841, EPI_ISL_2461842, EPI_ISL_2461843, EPI_ISL_2461844,
EPI_ISL_2461845, EPI_ISL_2461846, EPI_ISL_2461847, EPI_ISL_2461848, EPI_ISL_2461849, EPI_ISL_2461850, EPI_ISL_2461851, EPI_ISL_2461852, EPI_ISL_2461853, EPI_ISL_2461854, EPI_ISL_2461855, EPI_ISL_2461856, EPI_ISL_2461857, EPI_ISL_2461858, EPI_ISL_2461859, EPI_ISL_2461860, EPI_ISL_2461861, EPI_ISL_2461862, EPI_ISL_2461863, EPI_ISL_2461864, EPI_ISL_2461865, EPI_ISL_2461866,
EPI_ISL_2461867, EPI_ISL_2461868, EPI_ISL_2461869, EPI_ISL_2461870, EPI_ISL_2461871, EPI_ISL_2461872, EPI_ISL_2461873, EPI_ISL_2461874, EPI_ISL_2461875, EPI_ISL_2461876, EPI_ISL_2461877, EPI_ISL_2461878, EPI_ISL_2461879, EPI_ISL_2461880, EPI_ISL_2461881, EPI_ISL_2461882, EPI_ISL_2461883, EPI_ISL_2461884, EPI_ISL_2461885, EPI_ISL_2461886, EPI_ISL_2461887, EPI_ISL_2461888,
EPI_ISL_2461889, EPI_ISL_2461890, EPI_ISL_2461891, EPI_ISL_2461892, EPI_ISL_2461893, EPI_ISL_2461894, EPI_ISL_2461895, EPI_ISL_2461896, EPI_ISL_2461897, EPI_ISL_2461898, EPI_ISL_2461899, EPI_ISL_2461900, EPI_ISL_2461901, EPI_ISL_2461902, EPI_ISL_2461903, EPI_ISL_2461904, EPI_ISL_2461905, EPI_ISL_2461906, EPI_ISL_2461907, EPI_ISL_2461908, EPI_ISL_2461909, EPI_ISL_2461910,
EPI_ISL_2461911, EPI_ISL_2461912, EPI_ISL_2461913, EPI_ISL_2461914, EPI_ISL_2461915, EPI_ISL_2461916, EPI_ISL_2461917, EPI_ISL_2461918, EPI_ISL_2461919, EPI_ISL_2461920, EPI_ISL_2461921, EPI_ISL_2461922, EPI_ISL_2461923, EPI_ISL_2461924, EPI_ISL_2461925, EPI_ISL_2461926, EPI_ISL_2461927, EPI_ISL_2461928, EPI_ISL_2461929, EPI_ISL_2461930, EPI_ISL_2461931, EPI_ISL_2461932,
EPI_ISL_2461933, EPI_ISL_2461934, EPI_ISL_2461935, EPI_ISL_2461936, EPI_ISL_2461938, EPI_ISL_2461939, EPI_ISL_2461940, EPI_ISL_2461941, EPI_ISL_2461942, EPI_ISL_2461943, EPI_ISL_2461944, EPI_ISL_2461945, EPI_ISL_2461946, EPI_ISL_2461947, EPI_ISL_2461948, EPI_ISL_2461949, EPI_ISL_2461950, EPI_ISL_2461951, EPI_ISL_2461952, EPI_ISL_2461953, EPI_ISL_2461954, EPI_ISL_2461955,
EPI_ISL_2461956, EPI_ISL_2461957, EPI_ISL_2461958, EPI_ISL_2461959, EPI_ISL_2461960, EPI_ISL_2461961, EPI_ISL_2461962, EPI_ISL_2461963, EPI_ISL_2461964, EPI_ISL_2461965, EPI_ISL_2461966, EPI_ISL_2461967, EPI_ISL_2461968, EPI_ISL_2461969, EPI_ISL_2461970, EPI_ISL_2461971, EPI_ISL_2461972, EPI_ISL_2461973, EPI_ISL_2461974, EPI_ISL_2461975, EPI_ISL_2461976, EPI_ISL_2461977,
EPI_ISL_2461978, EPI_ISL_2461979, EPI_ISL_2461980, EPI_ISL_2461981, EPI_ISL_2461982, EPI_ISL_2461983, EPI_ISL_2461984, EPI_ISL_2461985, EPI_ISL_2461986, EPI_ISL_2461987, EPI_ISL_2461988, EPI_ISL_2461989, EPI_ISL_2461990, EPI_ISL_2461991, EPI_ISL_2461992, EPI_ISL_2461993, EPI_ISL_2461994, EPI_ISL_2462002, EPI_ISL_2462003, EPI_ISL_2462004, EPI_ISL_2462005, EPI_ISL_2462006,
EPI_ISL_2462007, EPI_ISL_2462008, EPI_ISL_2462009, EPI_ISL_2462010, EPI_ISL_2462011, EPI_ISL_2462012, EPI_ISL_2462013, EPI_ISL_2462014, EPI_ISL_2462015, EPI_ISL_2462016, EPI_ISL_2462017, EPI_ISL_2462018, EPI_ISL_2462019, EPI_ISL_2462020, EPI_ISL_2462021, EPI_ISL_2462022, EPI_ISL_2462023, EPI_ISL_2462024, EPI_ISL_2462025, EPI_ISL_2462026, EPI_ISL_2462027, EPI_ISL_2462028,
EPI_ISL_2462029, EPI_ISL_2462030, EPI_ISL_2462031, EPI_ISL_2462032, EPI_ISL_2462033, EPI_ISL_2462034, EPI_ISL_2462035, EPI_ISL_2462036, EPI_ISL_2462037, EPI_ISL_2462038, EPI_ISL_2462039, EPI_ISL_2462040, EPI_ISL_2462041, EPI_ISL_2462042, EPI_ISL_2462043, EPI_ISL_2502027, EPI_ISL_2502028, EPI_ISL_2502029, EPI_ISL_2502030, EPI_ISL_2502031, EPI_ISL_2502032, EPI_ISL_2502033,
EPI_ISL_2502034, EPI_ISL_2502035, EPI_ISL_2502036, EPI_ISL_2502037, EPI_ISL_2502038, EPI_ISL_2502039, EPI_ISL_2502040, EPI_ISL_2502041, EPI_ISL_2502042, EPI_ISL_2502043, EPI_ISL_2502044, EPI_ISL_2502045, EPI_ISL_2502046, EPI_ISL_2502047, EPI_ISL_2502048, EPI_ISL_2502049, EPI_ISL_2502050, EPI_ISL_2502051, EPI_ISL_2502052, EPI_ISL_2502053, EPI_ISL_2502054, EPI_ISL_2502055,
EPI_ISL_2502056, EPI_ISL_2502057, EPI_ISL_2502058, EPI_ISL_2502059, EPI_ISL_2502061, EPI_ISL_2502062, EPI_ISL_2502063, EPI_ISL_2502064, EPI_ISL_2502065, EPI_ISL_2502066, EPI_ISL_2502067, EPI_ISL_2502068, EPI_ISL_2502069, EPI_ISL_2502070, EPI_ISL_2502071, EPI_ISL_2502072, EPI_ISL_2502073, EPI_ISL_2502074, EPI_ISL_2502075, EPI_ISL_2502076, EPI_ISL_2502077, EPI_ISL_2502078,

EPI_ISL_2502079, EPI_ISL_2502080, EPI_ISL_2502081, EPI_ISL_2502082, EPI_ISL_2502083, EPI_ISL_2502084, EPI_ISL_2502085, EPI_ISL_2502086, EPI_ISL_2502087, EPI_ISL_2502088, EPI_ISL_2502089, EPI_ISL_2502090, EPI_ISL_2502091, EPI_ISL_2502092, EPI_ISL_2502093, EPI_ISL_2502094, EPI_ISL_2502095, EPI_ISL_2502096, EPI_ISL_2502097, EPI_ISL_2502099, EPI_ISL_2502100, EPI_ISL_2502101,
EPI_ISL_2502102, EPI_ISL_2502103, EPI_ISL_2502104, EPI_ISL_2502105, EPI_ISL_2502107, EPI_ISL_2502108, EPI_ISL_2502109, EPI_ISL_2502110, EPI_ISL_2502111, EPI_ISL_2502112, EPI_ISL_2502113, EPI_ISL_2502114, EPI_ISL_2502115, EPI_ISL_2502116, EPI_ISL_2502117, EPI_ISL_2502118, EPI_ISL_2502119, EPI_ISL_2502120, EPI_ISL_2502121, EPI_ISL_2502122, EPI_ISL_2502123, EPI_ISL_2502124,
EPI_ISL_2502125, EPI_ISL_2502126, EPI_ISL_2502127, EPI_ISL_2502128, EPI_ISL_2502129, EPI_ISL_2502130, EPI_ISL_2502131, EPI_ISL_2502132, EPI_ISL_2502134, EPI_ISL_2502135, EPI_ISL_2502136, EPI_ISL_2502137, EPI_ISL_2502138, EPI_ISL_2502139, EPI_ISL_2502140, EPI_ISL_2502141, EPI_ISL_2502142, EPI_ISL_2502143, EPI_ISL_2502144, EPI_ISL_2502145, EPI_ISL_2502146, EPI_ISL_2502147,
EPI_ISL_2502148, EPI_ISL_2502149, EPI_ISL_2502150, EPI_ISL_2502152, EPI_ISL_2502153, EPI_ISL_2502154, EPI_ISL_2502155, EPI_ISL_2502156, EPI_ISL_2502157, EPI_ISL_2502158, EPI_ISL_2502159, EPI_ISL_2502160, EPI_ISL_2502161, EPI_ISL_2502162, EPI_ISL_2502163, EPI_ISL_2502164, EPI_ISL_2502165, EPI_ISL_2502166, EPI_ISL_2502167, EPI_ISL_2502168, EPI_ISL_2502169, EPI_ISL_2502170,
EPI_ISL_2502171, EPI_ISL_2502173, EPI_ISL_2502174, EPI_ISL_2502175, EPI_ISL_2502176, EPI_ISL_2502177, EPI_ISL_2502179, EPI_ISL_2502180, EPI_ISL_2502181, EPI_ISL_2502182, EPI_ISL_2502183, EPI_ISL_2502184, EPI_ISL_2502186, EPI_ISL_2502187, EPI_ISL_2502188, EPI_ISL_2502189, EPI_ISL_2502190, EPI_ISL_2502191, EPI_ISL_2502192, EPI_ISL_2502193, EPI_ISL_2502194, EPI_ISL_2502195,
EPI_ISL_2502197, EPI_ISL_2502198, EPI_ISL_2502199, EPI_ISL_2502200, EPI_ISL_2502201, EPI_ISL_2502202, EPI_ISL_2502203, EPI_ISL_2502204, EPI_ISL_2502205, EPI_ISL_2502206, EPI_ISL_2502207, EPI_ISL_2502208, EPI_ISL_2502209, EPI_ISL_2502210, EPI_ISL_2502211, EPI_ISL_2502212, EPI_ISL_2502213, EPI_ISL_2502214, EPI_ISL_2502215, EPI_ISL_2502216, EPI_ISL_2502217, EPI_ISL_2502218,
EPI_ISL_2502219, EPI_ISL_2502220, EPI_ISL_2502221, EPI_ISL_2502222, EPI_ISL_2502223, EPI_ISL_2502224, EPI_ISL_2502225, EPI_ISL_2502226, EPI_ISL_2502227, EPI_ISL_2502228, EPI_ISL_2502229, EPI_ISL_2502230, EPI_ISL_2502231, EPI_ISL_2502232, EPI_ISL_2502233, EPI_ISL_2502234, EPI_ISL_2502235, EPI_ISL_2502236, EPI_ISL_2502237, EPI_ISL_2502238, EPI_ISL_2502239, EPI_ISL_2502240,
EPI_ISL_2502241, EPI_ISL_2502243, EPI_ISL_2502244, EPI_ISL_2502245, EPI_ISL_2502246, EPI_ISL_2502248, EPI_ISL_2502249, EPI_ISL_2502250, EPI_ISL_2502251, EPI_ISL_2502252, EPI_ISL_2502253, EPI_ISL_2502254, EPI_ISL_2502255, EPI_ISL_2502256, EPI_ISL_2502257, EPI_ISL_2502258, EPI_ISL_2502259, EPI_ISL_2502260, EPI_ISL_2502261, EPI_ISL_2502262, EPI_ISL_2502263, EPI_ISL_2502264,
EPI_ISL_2502265, EPI_ISL_2502266, EPI_ISL_2502267, EPI_ISL_2502268, EPI_ISL_2502269, EPI_ISL_2502270, EPI_ISL_2502271, EPI_ISL_2502272, EPI_ISL_2502273, EPI_ISL_2502274, EPI_ISL_2502275, EPI_ISL_2502276, EPI_ISL_2502277, EPI_ISL_2502278, EPI_ISL_2502279, EPI_ISL_2502280, EPI_ISL_2502281, EPI_ISL_2502282, EPI_ISL_2502283, EPI_ISL_2502284, EPI_ISL_2502285, EPI_ISL_2502286,
EPI_ISL_2502287, EPI_ISL_2502288, EPI_ISL_2502289, EPI_ISL_2502290, EPI_ISL_2502291, EPI_ISL_2502292, EPI_ISL_2502293, EPI_ISL_2502294, EPI_ISL_2502295, EPI_ISL_2502296, EPI_ISL_2502297, EPI_ISL_2502298, EPI_ISL_2502299, EPI_ISL_2502300, EPI_ISL_2502301, EPI_ISL_2502302, EPI_ISL_2502303, EPI_ISL_2502304, EPI_ISL_2502305, EPI_ISL_2502306, EPI_ISL_2502307, EPI_ISL_2504190,
EPI_ISL_2504191, EPI_ISL_2504192, EPI_ISL_2504193, EPI_ISL_2504194, EPI_ISL_2504195, EPI_ISL_2504196, EPI_ISL_2504197, EPI_ISL_2504198, EPI_ISL_2504199, EPI_ISL_2504200, EPI_ISL_2504201, EPI_ISL_2504202, EPI_ISL_2504203, EPI_ISL_2504204, EPI_ISL_2504205, EPI_ISL_2504206, EPI_ISL_2504207, EPI_ISL_2504208, EPI_ISL_2504209, EPI_ISL_2504210, EPI_ISL_2504211, EPI_ISL_2504212,
EPI_ISL_2504213, EPI_ISL_2504214, EPI_ISL_2504215, EPI_ISL_2504216, EPI_ISL_2504217, EPI_ISL_2504218, EPI_ISL_2504219, EPI_ISL_2504220, EPI_ISL_2504221, EPI_ISL_2504222, EPI_ISL_2504223, EPI_ISL_2504224, EPI_ISL_2504225, EPI_ISL_2504227, EPI_ISL_2504228, EPI_ISL_2504229, EPI_ISL_2504230, EPI_ISL_2504238, EPI_ISL_2504239, EPI_ISL_2504240, EPI_ISL_2504241, EPI_ISL_2504242,
EPI_ISL_2504243, EPI_ISL_2504244, EPI_ISL_2504246, EPI_ISL_2504247, EPI_ISL_2504248, EPI_ISL_2504249, EPI_ISL_2504250, EPI_ISL_2504251, EPI_ISL_2504252, EPI_ISL_2504253, EPI_ISL_2504254, EPI_ISL_2504255, EPI_ISL_2504256, EPI_ISL_2504257, EPI_ISL_2504258, EPI_ISL_2504259, EPI_ISL_2504260, EPI_ISL_2504261, EPI_ISL_2504262, EPI_ISL_2504263, EPI_ISL_2504264, EPI_ISL_2504265,
EPI_ISL_2504266, EPI_ISL_2504267, EPI_ISL_2504268, EPI_ISL_2504269, EPI_ISL_2504270, EPI_ISL_2504271, EPI_ISL_2504272, EPI_ISL_2504273, EPI_ISL_2504274, EPI_ISL_2504275, EPI_ISL_2504276, EPI_ISL_2504277, EPI_ISL_2504278, EPI_ISL_2504279, EPI_ISL_2504280, EPI_ISL_2504281, EPI_ISL_2504282, EPI_ISL_2504283, EPI_ISL_2504284, EPI_ISL_2504285, EPI_ISL_2504286, EPI_ISL_2504287,
EPI_ISL_2504288, EPI_ISL_2504289, EPI_ISL_2504290, EPI_ISL_2504291, EPI_ISL_2504292, EPI_ISL_2504293, EPI_ISL_2504294, EPI_ISL_2504295, EPI_ISL_2504296, EPI_ISL_2504297, EPI_ISL_2504298, EPI_ISL_2504299, EPI_ISL_2504300, EPI_ISL_2504301, EPI_ISL_2504302, EPI_ISL_2504303, EPI_ISL_2504304, EPI_ISL_2504305, EPI_ISL_2504306, EPI_ISL_2504307, EPI_ISL_2504308, EPI_ISL_2504309,
EPI_ISL_2504310, EPI_ISL_2504311, EPI_ISL_2504312, EPI_ISL_2504313, EPI_ISL_2504314, EPI_ISL_2504315, EPI_ISL_2504316, EPI_ISL_2504317, EPI_ISL_2504318, EPI_ISL_2504319, EPI_ISL_2504320, EPI_ISL_2504321, EPI_ISL_2504322, EPI_ISL_2504323, EPI_ISL_2504340, EPI_ISL_2504341, EPI_ISL_2504342, EPI_ISL_2504343, EPI_ISL_2504344, EPI_ISL_2504345, EPI_ISL_2504347, EPI_ISL_2504348,
EPI_ISL_2504349, EPI_ISL_2504350, EPI_ISL_2504351, EPI_ISL_2504352, EPI_ISL_2504353, EPI_ISL_2504354, EPI_ISL_2504355, EPI_ISL_2504356, EPI_ISL_2504357, EPI_ISL_2504358, EPI_ISL_2504359, EPI_ISL_2504360, EPI_ISL_2504361, EPI_ISL_2504362, EPI_ISL_2504363, EPI_ISL_2504364, EPI_ISL_2504365, EPI_ISL_2504366, EPI_ISL_2504367, EPI_ISL_2504368, EPI_ISL_2504369, EPI_ISL_2504370,
EPI_ISL_2504371, EPI_ISL_2504372, EPI_ISL_2504373, EPI_ISL_2504374, EPI_ISL_2504375, EPI_ISL_2504376, EPI_ISL_2504377, EPI_ISL_2504378, EPI_ISL_2504379, EPI_ISL_2504380, EPI_ISL_2504381, EPI_ISL_2504382, EPI_ISL_2504383, EPI_ISL_2504384, EPI_ISL_2504385, EPI_ISL_2504386, EPI_ISL_2504387, EPI_ISL_2504388, EPI_ISL_2504389, EPI_ISL_2504390, EPI_ISL_2504391, EPI_ISL_2504392,
EPI_ISL_2504393, EPI_ISL_2504394, EPI_ISL_2504395, EPI_ISL_2504396, EPI_ISL_2504397, EPI_ISL_2504398, EPI_ISL_2504399, EPI_ISL_2504400, EPI_ISL_2504401, EPI_ISL_2504402, EPI_ISL_2504403, EPI_ISL_2504404, EPI_ISL_2504405, EPI_ISL_2504406, EPI_ISL_2504407, EPI_ISL_2504408, EPI_ISL_2504409, EPI_ISL_2504410, EPI_ISL_2504411, EPI_ISL_2504412, EPI_ISL_2504413, EPI_ISL_2504414,
EPI_ISL_2504415, EPI_ISL_2504416, EPI_ISL_2504417, EPI_ISL_2504418, EPI_ISL_2504419, EPI_ISL_2504420, EPI_ISL_2504421, EPI_ISL_2504422, EPI_ISL_2504423, EPI_ISL_2504424, EPI_ISL_2504425, EPI_ISL_2504426, EPI_ISL_2504427, EPI_ISL_2504428, EPI_ISL_2504429, EPI_ISL_2504430, EPI_ISL_2504431, EPI_ISL_2504432, EPI_ISL_2504433, EPI_ISL_2504434, EPI_ISL_2504435, EPI_ISL_2504436,
EPI_ISL_2504437, EPI_ISL_2504438, EPI_ISL_2504439, EPI_ISL_2504440, EPI_ISL_2504441, EPI_ISL_2504442, EPI_ISL_2504443, EPI_ISL_2504444, EPI_ISL_2504445, EPI_ISL_2504446, EPI_ISL_2504447, EPI_ISL_2504448, EPI_ISL_2504449, EPI_ISL_2504450, EPI_ISL_2504451, EPI_ISL_2504452, EPI_ISL_2504453, EPI_ISL_2504454, EPI_ISL_2504455, EPI_ISL_2504456, EPI_ISL_2504457, EPI_ISL_2504458,
EPI_ISL_2504459, EPI_ISL_2504460, EPI_ISL_2504461, EPI_ISL_2504462, EPI_ISL_2504463, EPI_ISL_2504464, EPI_ISL_2504465, EPI_ISL_2504466, EPI_ISL_2504467, EPI_ISL_2504468, EPI_ISL_2504469, EPI_ISL_2504470, EPI_ISL_2504471, EPI_ISL_2504472, EPI_ISL_2504473, EPI_ISL_2504474, EPI_ISL_2504475, EPI_ISL_2504476, EPI_ISL_2504477, EPI_ISL_2504478, EPI_ISL_2504479, EPI_ISL_2504480,
EPI_ISL_2504481, EPI_ISL_2504482, EPI_ISL_2504483, EPI_ISL_2504484, EPI_ISL_2504485, EPI_ISL_2504486, EPI_ISL_2504487, EPI_ISL_2504488, EPI_ISL_2504489, EPI_ISL_2504490, EPI_ISL_2504491, EPI_ISL_2504492, EPI_ISL_2504493, EPI_ISL_2504494, EPI_ISL_2504495, EPI_ISL_2504496, EPI_ISL_2504497, EPI_ISL_2504498, EPI_ISL_2504499, EPI_ISL_2504500, EPI_ISL_2504501, EPI_ISL_2504502,
EPI_ISL_2504503, EPI_ISL_2504504, EPI_ISL_2504505, EPI_ISL_2504506, EPI_ISL_2504507, EPI_ISL_2504508, EPI_ISL_2504509, EPI_ISL_2504510, EPI_ISL_2504511, EPI_ISL_2504512, EPI_ISL_2504513, EPI_ISL_2504514, EPI_ISL_2504515, EPI_ISL_2504516, EPI_ISL_2504517, EPI_ISL_2504518, EPI_ISL_2504519, EPI_ISL_2504520, EPI_ISL_2504521, EPI_ISL_2504522, EPI_ISL_2504523, EPI_ISL_2504524,
EPI_ISL_2504525, EPI_ISL_2504526, EPI_ISL_2504527, EPI_ISL_2504528, EPI_ISL_2504616, EPI_ISL_2504617, EPI_ISL_2504618, EPI_ISL_2504619, EPI_ISL_2504620, EPI_ISL_2504621, EPI_ISL_2504622, EPI_ISL_2504623, EPI_ISL_2504624, EPI_ISL_2504625, EPI_ISL_2504626, EPI_ISL_2504627, EPI_ISL_2504628, EPI_ISL_2504629, EPI_ISL_2504630, EPI_ISL_2504631, EPI_ISL_2504632, EPI_ISL_2504633,
EPI_ISL_2504634, EPI_ISL_2504635, EPI_ISL_2504636, EPI_ISL_2504637, EPI_ISL_2504638, EPI_ISL_2504639, EPI_ISL_2504640, EPI_ISL_2504641, EPI_ISL_2504642, EPI_ISL_2504643, EPI_ISL_2504644, EPI_ISL_2504645, EPI_ISL_2504646, EPI_ISL_2504647, EPI_ISL_2504648, EPI_ISL_2504649, EPI_ISL_2504650, EPI_ISL_2504651, EPI_ISL_2504652, EPI_ISL_2504653, EPI_ISL_2504654, EPI_ISL_2504655,
EPI_ISL_2504656, EPI_ISL_2504657, EPI_ISL_2504658, EPI_ISL_2504659, EPI_ISL_2504660, EPI_ISL_2504661, EPI_ISL_2504662, EPI_ISL_2504663, EPI_ISL_2504664, EPI_ISL_2504665, EPI_ISL_2504666, EPI_ISL_2504667, EPI_ISL_2504668, EPI_ISL_2504669, EPI_ISL_2504670, EPI_ISL_2504671, EPI_ISL_2504672, EPI_ISL_2504673, EPI_ISL_2504674, EPI_ISL_2504675, EPI_ISL_2504676, EPI_ISL_2504677,
EPI_ISL_2504678, EPI_ISL_2504679, EPI_ISL_2504680, EPI_ISL_2504681, EPI_ISL_2504682, EPI_ISL_2504683, EPI_ISL_2504684, EPI_ISL_2504685, EPI_ISL_2504686, EPI_ISL_2504687, EPI_ISL_2504688, EPI_ISL_2504689, EPI_ISL_2504690, EPI_ISL_2504691, EPI_ISL_2504692, EPI_ISL_2504693, EPI_ISL_2504694, EPI_ISL_2504695, EPI_ISL_2504696, EPI_ISL_2504697, EPI_ISL_2504698, EPI_ISL_2504699,
EPI_ISL_2504700, EPI_ISL_2504701, EPI_ISL_2504702, EPI_ISL_2504703, EPI_ISL_2504704, EPI_ISL_2504705, EPI_ISL_2504706, EPI_ISL_2504707, EPI_ISL_2504708, EPI_ISL_2504709, EPI_ISL_2504710, EPI_ISL_2504711, EPI_ISL_2504712, EPI_ISL_2504713, EPI_ISL_2504714, EPI_ISL_2504715, EPI_ISL_2504716, EPI_ISL_2504717, EPI_ISL_2504718, EPI_ISL_2504719, EPI_ISL_2504720, EPI_ISL_2504721,
EPI_ISL_2504722, EPI_ISL_2504723, EPI_ISL_2504724, EPI_ISL_2504725, EPI_ISL_2504726, EPI_ISL_2504727, EPI_ISL_2504728, EPI_ISL_2504729, EPI_ISL_2504730, EPI_ISL_2504731, EPI_ISL_2504732, EPI_ISL_2504733, EPI_ISL_2504734, EPI_ISL_2504735, EPI_ISL_2504736, EPI_ISL_2504737, EPI_ISL_2504738, EPI_ISL_2504739, EPI_ISL_2504740, EPI_ISL_2504741, EPI_ISL_2504742, EPI_ISL_2504743,
EPI_ISL_2504744, EPI_ISL_2504745, EPI_ISL_2504746, EPI_ISL_2504747, EPI_ISL_2504748, EPI_ISL_2504749, EPI_ISL_2504750, EPI_ISL_2504751, EPI_ISL_2504752, EPI_ISL_2504753, EPI_ISL_2504754, EPI_ISL_2504755, EPI_ISL_2504756, EPI_ISL_2504757, EPI_ISL_2504758, EPI_ISL_2504759, EPI_ISL_2504760, EPI_ISL_2504761, EPI_ISL_2504762, EPI_ISL_2504763, EPI_ISL_2504764, EPI_ISL_2504765,
EPI_ISL_2504766, EPI_ISL_2504767, EPI_ISL_2504768, EPI_ISL_2504769, EPI_ISL_2504770, EPI_ISL_2504771, EPI_ISL_2504772, EPI_ISL_2504773, EPI_ISL_2504774, EPI_ISL_2504775, EPI_ISL_2504776, EPI_ISL_2504777, EPI_ISL_2504778, EPI_ISL_2504779, EPI_ISL_2504780, EPI_ISL_2504781, EPI_ISL_2504782, EPI_ISL_2504783, EPI_ISL_2504784, EPI_ISL_2504785, EPI_ISL_2504786, EPI_ISL_2504787,
EPI_ISL_2504788, EPI_ISL_2504789, EPI_ISL_2504790, EPI_ISL_2504791, EPI_ISL_2504792, EPI_ISL_2504793, EPI_ISL_2504794, EPI_ISL_2504795, EPI_ISL_2504796, EPI_ISL_2504797, EPI_ISL_2504798, EPI_ISL_2504799, EPI_ISL_2504800, EPI_ISL_2504801, EPI_ISL_2504802, EPI_ISL_2504803, EPI_ISL_2504804, EPI_ISL_2504805, EPI_ISL_2504806, EPI_ISL_2504807, EPI_ISL_2504808, EPI_ISL_2504809,
EPI_ISL_2504810, EPI_ISL_2504811, EPI_ISL_2504812, EPI_ISL_2504813, EPI_ISL_2504814, EPI_ISL_2504815, EPI_ISL_2504816, EPI_ISL_2504817, EPI_ISL_2504818, EPI_ISL_2504819, EPI_ISL_2504820, EPI_ISL_2504821, EPI_ISL_2504822, EPI_ISL_2504823, EPI_ISL_2504824, EPI_ISL_2504825, EPI_ISL_2504826, EPI_ISL_2504827, EPI_ISL_2504828, EPI_ISL_2504829, EPI_ISL_2504830, EPI_ISL_2504831,
EPI_ISL_2504832, EPI_ISL_2504863, EPI_ISL_2504864, EPI_ISL_2504865, EPI_ISL_2504866, EPI_ISL_2504867, EPI_ISL_2504868, EPI_ISL_2504869, EPI_ISL_2504870, EPI_ISL_2504871, EPI_ISL_2504872, EPI_ISL_2504873, EPI_ISL_2504874, EPI_ISL_2504875, EPI_ISL_2504876, EPI_ISL_2504877, EPI_ISL_2504878, EPI_ISL_2504879, EPI_ISL_2504880, EPI_ISL_2504881, EPI_ISL_2504882, EPI_ISL_2504883,
EPI_ISL_2504884, EPI_ISL_2504885, EPI_ISL_2504886, EPI_ISL_2504887, EPI_ISL_2504888, EPI_ISL_2504889, EPI_ISL_2504890, EPI_ISL_2504891, EPI_ISL_2504892, EPI_ISL_2504893, EPI_ISL_2504894, EPI_ISL_2504895, EPI_ISL_2504896, EPI_ISL_2504897, EPI_ISL_2504898, EPI_ISL_2504899, EPI_ISL_2504900, EPI_ISL_2504901, EPI_ISL_2504902, EPI_ISL_2504903, EPI_ISL_2504904, EPI_ISL_2504905,
EPI_ISL_2504906, EPI_ISL_2504907, EPI_ISL_2504908, EPI_ISL_2504909, EPI_ISL_2504910, EPI_ISL_2504911, EPI_ISL_2504912, EPI_ISL_2504913, EPI_ISL_2504914, EPI_ISL_2504915, EPI_ISL_2504916, EPI_ISL_2504917, EPI_ISL_2504918, EPI_ISL_2504919, EPI_ISL_2504920, EPI_ISL_2504921, EPI_ISL_2504922, EPI_ISL_2504923, EPI_ISL_2504924, EPI_ISL_2504925, EPI_ISL_2504926, EPI_ISL_2504927,
EPI_ISL_2504928, EPI_ISL_2504929, EPI_ISL_2504930, EPI_ISL_2504931, EPI_ISL_2504932, EPI_ISL_2504933, EPI_ISL_2504934, EPI_ISL_2504935, EPI_ISL_2504936, EPI_ISL_2504937, EPI_ISL_2504938, EPI_ISL_2504939, EPI_ISL_2504940, EPI_ISL_2504941, EPI_ISL_2504942, EPI_ISL_2504943, EPI_ISL_2504944, EPI_ISL_2504945, EPI_ISL_2504946, EPI_ISL_2504947, EPI_ISL_2504948, EPI_ISL_2504949,
EPI_ISL_2504950, EPI_ISL_2504951, EPI_ISL_2504952, EPI_ISL_2504953, EPI_ISL_2504954, EPI_ISL_2504955, EPI_ISL_2504956, EPI_ISL_2504957, EPI_ISL_2504958, EPI_ISL_2504959, EPI_ISL_2504960, EPI_ISL_2504961, EPI_ISL_2504962, EPI_ISL_2504963, EPI_ISL_2504964, EPI_ISL_2504965, EPI_ISL_2504966, EPI_ISL_2504967, EPI_ISL_2504968, EPI_ISL_2504969, EPI_ISL_2504970, EPI_ISL_2504971,
EPI_ISL_2504972, EPI_ISL_2504973, EPI_ISL_2504974, EPI_ISL_2504975, EPI_ISL_2504976, EPI_ISL_2504977, EPI_ISL_2504978, EPI_ISL_2504979, EPI_ISL_2504980, EPI_ISL_2504981, EPI_ISL_2504982, EPI_ISL_2504983, EPI_ISL_2504984, EPI_ISL_2504985, EPI_ISL_2504986, EPI_ISL_2504987, EPI_ISL_2504988, EPI_ISL_2504989, EPI_ISL_2504990, EPI_ISL_2504991, EPI_ISL_2504992, EPI_ISL_2504993,
EPI_ISL_2504994, EPI_ISL_2504995, EPI_ISL_2504996, EPI_ISL_2504997, EPI_ISL_2504998, EPI_ISL_2504999, EPI_ISL_2505000, EPI_ISL_2505001, EPI_ISL_2505002, EPI_ISL_2505003, EPI_ISL_2505004, EPI_ISL_2505005, EPI_ISL_2505006, EPI_ISL_2505007, EPI_ISL_2505008, EPI_ISL_2505009, EPI_ISL_2505010, EPI_ISL_2505011, EPI_ISL_2505012, EPI_ISL_2505013, EPI_ISL_2505014, EPI_ISL_2505015,
EPI_ISL_2505016, EPI_ISL_2505017, EPI_ISL_2505018, EPI_ISL_2505019, EPI_ISL_2505020, EPI_ISL_2505021, EPI_ISL_2505022, EPI_ISL_2505023, EPI_ISL_2505024, EPI_ISL_2505025, EPI_ISL_2505026, EPI_ISL_2505027, EPI_ISL_2505028, EPI_ISL_2505029, EPI_ISL_2505030, EPI_ISL_2505031, EPI_ISL_2505032, EPI_ISL_2505033, EPI_ISL_2505034, EPI_ISL_2505035, EPI_ISL_2505036, EPI_ISL_2505037,
EPI_ISL_2505038, EPI_ISL_2505039, EPI_ISL_2505040, EPI_ISL_2505041, EPI_ISL_2505042, EPI_ISL_2505043, EPI_ISL_2505044, EPI_ISL_2505045, EPI_ISL_2505046, EPI_ISL_2505047, EPI_ISL_2505048, EPI_ISL_2505049, EPI_ISL_2505050, EPI_ISL_2505051, EPI_ISL_2505052, EPI_ISL_2505053, EPI_ISL_2505054, EPI_ISL_2505055, EPI_ISL_2505056, EPI_ISL_2505057, EPI_ISL_2505058, EPI_ISL_2505059,
EPI_ISL_2505060, EPI_ISL_2505061, EPI_ISL_2505062, EPI_ISL_2505063, EPI_ISL_2505064, EPI_ISL_2505065, EPI_ISL_2505066, EPI_ISL_2505067, EPI_ISL_2505068, EPI_ISL_2505069, EPI_ISL_2505070, EPI_ISL_2505071, EPI_ISL_2505072, EPI_ISL_2505073, EPI_ISL_2505074, EPI_ISL_2505075, EPI_ISL_2505076, EPI_ISL_2505077, EPI_ISL_2505078, EPI_ISL_2505079, EPI_ISL_2505080, EPI_ISL_2505081,
EPI_ISL_2505082, EPI_ISL_2505083, EPI_ISL_2505084, EPI_ISL_2505085, EPI_ISL_2505086, EPI_ISL_2505087, EPI_ISL_2505088, EPI_ISL_2505089, EPI_ISL_2505090, EPI_ISL_2505091, EPI_ISL_2505092, EPI_ISL_2505093, EPI_ISL_2505094, EPI_ISL_2505095, EPI_ISL_2505096, EPI_ISL_2505097, EPI_ISL_2505098, EPI_ISL_2505099, EPI_ISL_2505100, EPI_ISL_2505101, EPI_ISL_2505102, EPI_ISL_2505103,
EPI_ISL_2505104, EPI_ISL_2505105, EPI_ISL_2505106, EPI_ISL_2505107, EPI_ISL_2505108, EPI_ISL_2505109, EPI_ISL_2505110, EPI_ISL_2505111, EPI_ISL_2505112, EPI_ISL_2505113, EPI_ISL_2505114, EPI_ISL_2505115, EPI_ISL_2505116, EPI_ISL_2505117, EPI_ISL_2505118, EPI_ISL_2505119, EPI_ISL_2505120, EPI_ISL_2505121, EPI_ISL_2505122, EPI_ISL_2505123, EPI_ISL_2505124, EPI_ISL_2505125,
EPI_ISL_2505126, EPI_ISL_2505127, EPI_ISL_2505128, EPI_ISL_2505129, EPI_ISL_2505130, EPI_ISL_2505131, EPI_ISL_2505132, EPI_ISL_2505133, EPI_ISL_2505134, EPI_ISL_2505135, EPI_ISL_2505136, EPI_ISL_2505137, EPI_ISL_2505138, EPI_ISL_2505139, EPI_ISL_2505140, EPI_ISL_2505141, EPI_ISL_2505142, EPI_ISL_2505143, EPI_ISL_2505144, EPI_ISL_2505145, EPI_ISL_2505146, EPI_ISL_2505147,
EPI_ISL_2505148, EPI_ISL_2505149, EPI_ISL_2505150, EPI_ISL_2505151, EPI_ISL_2505152, EPI_ISL_2505153, EPI_ISL_2505154, EPI_ISL_2505155, EPI_ISL_2505156, EPI_ISL_2505157, EPI_ISL_2505158, EPI_ISL_2505159, EPI_ISL_2505160, EPI_ISL_2505161, EPI_ISL_2505162, EPI_ISL_2505163, EPI_ISL_2555544, EPI_ISL_2555545, EPI_ISL_2555546, EPI_ISL_2555547, EPI_ISL_2555548, EPI_ISL_2555549,
EPI_ISL_2555550, EPI_ISL_2555551, EPI_ISL_2555552, EPI_ISL_2555553, EPI_ISL_2555554, EPI_ISL_2555555, EPI_ISL_2555556, EPI_ISL_2555557, EPI_ISL_2555558, EPI_ISL_2555559, EPI_ISL_2555560, EPI_ISL_2555561, EPI_ISL_2555562, EPI_ISL_2555563, EPI_ISL_2555564, EPI_ISL_2555565, EPI_ISL_2555566, EPI_ISL_2555567, EPI_ISL_2555568, EPI_ISL_2555569, EPI_ISL_2555570, EPI_ISL_2555571,
EPI_ISL_2555572, EPI_ISL_2555573, EPI_ISL_2555574, EPI_ISL_2555575, EPI_ISL_2555576, EPI_ISL_2555577, EPI_ISL_2555578, EPI_ISL_2555579, EPI_ISL_2555580, EPI_ISL_2555581, EPI_ISL_2555582, EPI_ISL_2555583, EPI_ISL_2555584, EPI_ISL_2555585, EPI_ISL_2555586, EPI_ISL_2555587, EPI_ISL_2555588, EPI_ISL_2555589, EPI_ISL_2555590, EPI_ISL_2555591, EPI_ISL_2555592, EPI_ISL_2555593,
EPI_ISL_2555594, EPI_ISL_2555595, EPI_ISL_2555596, EPI_ISL_2555597, EPI_ISL_2555598, EPI_ISL_2555599, EPI_ISL_2555600, EPI_ISL_2555601, EPI_ISL_2555602, EPI_ISL_2555603, EPI_ISL_2555604, EPI_ISL_2555605, EPI_ISL_2555606, EPI_ISL_2555607, EPI_ISL_2555608, EPI_ISL_2555609, EPI_ISL_2555610, EPI_ISL_2555611, EPI_ISL_2555612, EPI_ISL_2555613, EPI_ISL_2555614, EPI_ISL_2555615,
EPI_ISL_2555616, EPI_ISL_2555617, EPI_ISL_2555618, EPI_ISL_2555619, EPI_ISL_2555620, EPI_ISL_2555621, EPI_ISL_2555622, EPI_ISL_2555623, EPI_ISL_2555624, EPI_ISL_2555625, EPI_ISL_2555626, EPI_ISL_2555627, EPI_ISL_2555628, EPI_ISL_2555629, EPI_ISL_2555630, EPI_ISL_2555631, EPI_ISL_2555632, EPI_ISL_2555633, EPI_ISL_2555634, EPI_ISL_2555635, EPI_ISL_2555636, EPI_ISL_2555637,
EPI_ISL_2555638, EPI_ISL_2555639, EPI_ISL_2555640, EPI_ISL_2555641, EPI_ISL_2555642, EPI_ISL_2555643, EPI_ISL_2555644, EPI_ISL_2555645, EPI_ISL_2555646, EPI_ISL_2555647, EPI_ISL_2555648, EPI_ISL_2555649, EPI_ISL_2555650, EPI_ISL_2555651, EPI_ISL_2555652, EPI_ISL_2555653, EPI_ISL_2555654, EPI_ISL_2555655, EPI_ISL_2555656, EPI_ISL_2555657, EPI_ISL_2555658, EPI_ISL_2555659,
EPI_ISL_2555660, EPI_ISL_2555733, EPI_ISL_2555734, EPI_ISL_2555735, EPI_ISL_2555736, EPI_ISL_2555737, EPI_ISL_2555738, EPI_ISL_2555739, EPI_ISL_2555740, EPI_ISL_2555741, EPI_ISL_2555742, EPI_ISL_2555743, EPI_ISL_2555744, EPI_ISL_2555745, EPI_ISL_2555746, EPI_ISL_2555747, EPI_ISL_2555748, EPI_ISL_2555749, EPI_ISL_2555750, EPI_ISL_2555751, EPI_ISL_2555752, EPI_ISL_2555753,
EPI_ISL_2555754, EPI_ISL_2555755, EPI_ISL_2555756, EPI_ISL_2555757, EPI_ISL_2555758, EPI_ISL_2555759, EPI_ISL_2555760, EPI_ISL_2555761, EPI_ISL_2555762, EPI_ISL_2555763, EPI_ISL_2555764, EPI_ISL_2555765, EPI_ISL_2555766, EPI_ISL_2555767, EPI_ISL_2555768, EPI_ISL_2555769, EPI_ISL_2555770, EPI_ISL_2555771, EPI_ISL_2555772, EPI_ISL_2555773, EPI_ISL_2555774, EPI_ISL_2555775,
EPI_ISL_2555776, EPI_ISL_2555777, EPI_ISL_2555778, EPI_ISL_2555779, EPI_ISL_2555780, EPI_ISL_2555781, EPI_ISL_2555782, EPI_ISL_2555783, EPI_ISL_2555784, EPI_ISL_2555785, EPI_ISL_2555786, EPI_ISL_2555787, EPI_ISL_2555788, EPI_ISL_2555789, EPI_ISL_2555790, EPI_ISL_2555791, EPI_ISL_2555792, EPI_ISL_2555793, EPI_ISL_2555794, EPI_ISL_2555795, EPI_ISL_2555796, EPI_ISL_2555797,
EPI_ISL_2555798, EPI_ISL_2555799, EPI_ISL_2555800, EPI_ISL_2555801, EPI_ISL_2555802, EPI_ISL_2555803, EPI_ISL_2555804, EPI_ISL_2555805, EPI_ISL_2555806, EPI_ISL_2555807, EPI_ISL_2555808, EPI_ISL_2555809, EPI_ISL_2555810, EPI_ISL_2555811, EPI_ISL_2555812, EPI_ISL_2555813, EPI_ISL_2555814, EPI_ISL_2555815, EPI_ISL_2555816, EPI_ISL_2555817, EPI_ISL_2555818, EPI_ISL_2555819,
EPI_ISL_2555820, EPI_ISL_2555821, EPI_ISL_2555822, EPI_ISL_2555823, EPI_ISL_2555824, EPI_ISL_2555825, EPI_ISL_2555826, EPI_ISL_2555827, EPI_ISL_2555828, EPI_ISL_2555829, EPI_ISL_2555830, EPI_ISL_2555831, EPI_ISL_2555832, EPI_ISL_2555833, EPI_ISL_2555834, EPI_ISL_2555835, EPI_ISL_2555836, EPI_ISL_2555837, EPI_ISL_2555838, EPI_ISL_2555839, EPI_ISL_2555840, EPI_ISL_2555841,
EPI_ISL_2555842, EPI_ISL_2555843, EPI_ISL_2555844, EPI_ISL_2555845, EPI_ISL_2555846, EPI_ISL_2555847, EPI_ISL_2555848, EPI_ISL_2555849, EPI_ISL_2555850, EPI_ISL_2555851, EPI_ISL_2555852, EPI_ISL_2555853, EPI_ISL_2555854, EPI_ISL_2555855, EPI_ISL_2555856, EPI_ISL_2555857, EPI_ISL_2555858, EPI_ISL_2555859, EPI_ISL_2555860, EPI_ISL_2555861, EPI_ISL_2555862, EPI_ISL_2555863,
EPI_ISL_2555864, EPI_ISL_2555865, EPI_ISL_2555866, EPI_ISL_2555867, EPI_ISL_2555868, EPI_ISL_2555869, EPI_ISL_2555870, EPI_ISL_2555871, EPI_ISL_2555872, EPI_ISL_2555873, EPI_ISL_2555874, EPI_ISL_2555875, EPI_ISL_2555876, EPI_ISL_2555877, EPI_ISL_2555878, EPI_ISL_2555879, EPI_ISL_2555880, EPI_ISL_2555881, EPI_ISL_2555882, EPI_ISL_2555883, EPI_ISL_2555884, EPI_ISL_2555885,
EPI_ISL_2555886, EPI_ISL_2555887, EPI_ISL_2555888, EPI_ISL_2555889, EPI_ISL_2555890, EPI_ISL_2555891, EPI_ISL_2555892, EPI_ISL_2555893, EPI_ISL_2555894, EPI_ISL_2555895, EPI_ISL_2555896, EPI_ISL_2555897, EPI_ISL_2555898, EPI_ISL_2555899, EPI_ISL_2555900, EPI_ISL_2555901, EPI_ISL_2555902, EPI_ISL_2555903, EPI_ISL_2555904, EPI_ISL_2555905, EPI_ISL_2555906, EPI_ISL_2555907,
EPI_ISL_2555908, EPI_ISL_2555909, EPI_ISL_2555910, EPI_ISL_2555911, EPI_ISL_2555912, EPI_ISL_2555913, EPI_ISL_2555914, EPI_ISL_2555915, EPI_ISL_2555916, EPI_ISL_2555917, EPI_ISL_2555918, EPI_ISL_2555919, EPI_ISL_2555920, EPI_ISL_2555921, EPI_ISL_2555922, EPI_ISL_2555923, EPI_ISL_2555924, EPI_ISL_2555925, EPI_ISL_2555926, EPI_ISL_2555927, EPI_ISL_2555928, EPI_ISL_2555929,
EPI_ISL_2555930, EPI_ISL_2555931, EPI_ISL_2555932, EPI_ISL_2555933, EPI_ISL_2555934, EPI_ISL_2555935, EPI_ISL_2555936, EPI_ISL_2555937, EPI_ISL_2555938, EPI_ISL_2555939, EPI_ISL_2555940, EPI_ISL_2555941, EPI_ISL_2555942, EPI_ISL_2555943, EPI_ISL_2555944, EPI_ISL_2555945, EPI_ISL_2555946, EPI_ISL_2555947, EPI_ISL_2555948, EPI_ISL_2555949, EPI_ISL_2555950, EPI_ISL_2555951,
EPI_ISL_2555952, EPI_ISL_2555953, EPI_ISL_2555954, EPI_ISL_2555955, EPI_ISL_2555956, EPI_ISL_2555957, EPI_ISL_2555958, EPI_ISL_2555959, EPI_ISL_2555960, EPI_ISL_2555961, EPI_ISL_2555962, EPI_ISL_2555963, EPI_ISL_2555964, EPI_ISL_2555965, EPI_ISL_2555966, EPI_ISL_2555967, EPI_ISL_2555968, EPI_ISL_2555971, EPI_ISL_2555972, EPI_ISL_2555973, EPI_ISL_2555974, EPI_ISL_2555975,
EPI_ISL_2555976, EPI_ISL_2555977, EPI_ISL_2555978, EPI_ISL_2555979, EPI_ISL_2555980, EPI_ISL_2555981, EPI_ISL_2555982, EPI_ISL_2555983, EPI_ISL_2555984, EPI_ISL_2555985, EPI_ISL_2555986, EPI_ISL_2555987, EPI_ISL_2555988, EPI_ISL_2555989, EPI_ISL_2555990, EPI_ISL_2555991, EPI_ISL_2555992, EPI_ISL_2555993, EPI_ISL_2555994, EPI_ISL_2555995, EPI_ISL_2555996, EPI_ISL_2555997,
EPI_ISL_2555998, EPI_ISL_2555999, EPI_ISL_2556000, EPI_ISL_2556001, EPI_ISL_2556002, EPI_ISL_2556003, EPI_ISL_2556004, EPI_ISL_2556005, EPI_ISL_2556006, EPI_ISL_2556007, EPI_ISL_2556008, EPI_ISL_2556009, EPI_ISL_2556010, EPI_ISL_2556011, EPI_ISL_2556012, EPI_ISL_2556013, EPI_ISL_2556014, EPI_ISL_2556015, EPI_ISL_2556016, EPI_ISL_2556017, EPI_ISL_2556018, EPI_ISL_2556019,
EPI_ISL_2556020, EPI_ISL_2556021, EPI_ISL_2556022, EPI_ISL_2556023, EPI_ISL_2556024, EPI_ISL_2556025, EPI_ISL_2556026, EPI_ISL_2556027, EPI_ISL_2556028, EPI_ISL_2556029, EPI_ISL_2556030, EPI_ISL_2556031, EPI_ISL_2556032, EPI_ISL_2556033, EPI_ISL_2556034, EPI_ISL_2556035, EPI_ISL_2556036, EPI_ISL_2556037, EPI_ISL_2556038, EPI_ISL_2556039, EPI_ISL_2556040, EPI_ISL_2556041,
EPI_ISL_2556042, EPI_ISL_2556043, EPI_ISL_2556044, EPI_ISL_2556045, EPI_ISL_2556046, EPI_ISL_2556047, EPI_ISL_2556048, EPI_ISL_2556049, EPI_ISL_2556050, EPI_ISL_2556051, EPI_ISL_2556052, EPI_ISL_2556053, EPI_ISL_2556054, EPI_ISL_2556055, EPI_ISL_2556056, EPI_ISL_2556057, EPI_ISL_2556058, EPI_ISL_2556059, EPI_ISL_2556060, EPI_ISL_2556061, EPI_ISL_2556062, EPI_ISL_2556063,
EPI_ISL_2556064, EPI_ISL_2556065, EPI_ISL_2556066, EPI_ISL_2556067, EPI_ISL_2556068, EPI_ISL_2556069, EPI_ISL_2556070, EPI_ISL_2556071, EPI_ISL_2556072, EPI_ISL_2556073, EPI_ISL_2556074, EPI_ISL_2556075, EPI_ISL_2556076, EPI_ISL_2556077, EPI_ISL_2556078, EPI_ISL_2556079, EPI_ISL_2556080, EPI_ISL_2556081, EPI_ISL_2556082, EPI_ISL_2556083, EPI_ISL_2556084, EPI_ISL_2556085,
EPI_ISL_2556086, EPI_ISL_2556087, EPI_ISL_2556088, EPI_ISL_2556089, EPI_ISL_2556090, EPI_ISL_2556091, EPI_ISL_2556092, EPI_ISL_2556093, EPI_ISL_2556094, EPI_ISL_2556095, EPI_ISL_2556096, EPI_ISL_2556097, EPI_ISL_2556098, EPI_ISL_2556099, EPI_ISL_2556100, EPI_ISL_2556101, EPI_ISL_2556102, EPI_ISL_2556103, EPI_ISL_2556104, EPI_ISL_2556105, EPI_ISL_2556106, EPI_ISL_2556107,
EPI_ISL_2556108, EPI_ISL_2556109, EPI_ISL_2556110, EPI_ISL_2556111, EPI_ISL_2556112, EPI_ISL_2556121, EPI_ISL_2556122, EPI_ISL_2556123, EPI_ISL_2556124, EPI_ISL_2556125, EPI_ISL_2556126, EPI_ISL_2556127, EPI_ISL_2556128, EPI_ISL_2556129, EPI_ISL_2556130, EPI_ISL_2556131, EPI_ISL_2556132, EPI_ISL_2556133, EPI_ISL_2556134, EPI_ISL_2556135, EPI_ISL_2556136, EPI_ISL_2556137,
EPI_ISL_2556138, EPI_ISL_2556139, EPI_ISL_2556140, EPI_ISL_2556141, EPI_ISL_2556142, EPI_ISL_2556143, EPI_ISL_2556144, EPI_ISL_2556145, EPI_ISL_2556146, EPI_ISL_2556147, EPI_ISL_2556148, EPI_ISL_2556149, EPI_ISL_2556150, EPI_ISL_2556151, EPI_ISL_2556152, EPI_ISL_2556153, EPI_ISL_2556154, EPI_ISL_2556155, EPI_ISL_2556156, EPI_ISL_2556157, EPI_ISL_2556158, EPI_ISL_2556159,
EPI_ISL_2556160, EPI_ISL_2556161, EPI_ISL_2556162, EPI_ISL_2556163, EPI_ISL_2556164, EPI_ISL_2556165, EPI_ISL_2556166, EPI_ISL_2556167, EPI_ISL_2556168, EPI_ISL_2556169, EPI_ISL_2556170, EPI_ISL_2556171, EPI_ISL_2556172, EPI_ISL_2556173, EPI_ISL_2556174, EPI_ISL_2556175, EPI_ISL_2556176, EPI_ISL_2556177, EPI_ISL_2556178, EPI_ISL_2556179, EPI_ISL_2556180, EPI_ISL_2556181,
EPI_ISL_2556182, EPI_ISL_2556183, EPI_ISL_2556184, EPI_ISL_2556185, EPI_ISL_2556186, EPI_ISL_2556187, EPI_ISL_2556188, EPI_ISL_2556189, EPI_ISL_2556190, EPI_ISL_2556191, EPI_ISL_2556192, EPI_ISL_2556193, EPI_ISL_2556194, EPI_ISL_2556195, EPI_ISL_2556196, EPI_ISL_2556197, EPI_ISL_2556198, EPI_ISL_2556199, EPI_ISL_2556200, EPI_ISL_2556201, EPI_ISL_2556202, EPI_ISL_2556203,
EPI_ISL_2556204, EPI_ISL_2556205, EPI_ISL_2556206, EPI_ISL_2556207, EPI_ISL_2556208, EPI_ISL_2556209, EPI_ISL_2556210, EPI_ISL_2556211, EPI_ISL_2556212, EPI_ISL_2556213, EPI_ISL_2556214, EPI_ISL_2556215, EPI_ISL_2556216, EPI_ISL_2556217, EPI_ISL_2556218, EPI_ISL_2556219, EPI_ISL_2556220, EPI_ISL_2556221, EPI_ISL_2556222, EPI_ISL_2556223, EPI_ISL_2556224, EPI_ISL_2556225,
EPI_ISL_2556226, EPI_ISL_2556227, EPI_ISL_2556228, EPI_ISL_2556229, EPI_ISL_2556230, EPI_ISL_2556231, EPI_ISL_2556232, EPI_ISL_2556233, EPI_ISL_2556234, EPI_ISL_2556235, EPI_ISL_2556236, EPI_ISL_2556237, EPI_ISL_2556238, EPI_ISL_2556239, EPI_ISL_2556240, EPI_ISL_2556241, EPI_ISL_2556242, EPI_ISL_2556243, EPI_ISL_2556244, EPI_ISL_2556245, EPI_ISL_2556246, EPI_ISL_2556247,
EPI_ISL_2556248, EPI_ISL_2556249, EPI_ISL_2556250, EPI_ISL_2556251, EPI_ISL_2556252, EPI_ISL_2556253, EPI_ISL_2556254, EPI_ISL_2556255, EPI_ISL_2556256, EPI_ISL_2556257, EPI_ISL_2556258, EPI_ISL_2556259, EPI_ISL_2556260, EPI_ISL_2556261, EPI_ISL_2556262, EPI_ISL_2556263, EPI_ISL_2556264, EPI_ISL_2556265, EPI_ISL_2556266, EPI_ISL_2556267, EPI_ISL_2556268, EPI_ISL_2556269,
EPI_ISL_2556270, EPI_ISL_2556271, EPI_ISL_2556272, EPI_ISL_2556273, EPI_ISL_2556274, EPI_ISL_2556275, EPI_ISL_2556276, EPI_ISL_2556277, EPI_ISL_2556278, EPI_ISL_2556279, EPI_ISL_2556280, EPI_ISL_2556281, EPI_ISL_2556282, EPI_ISL_2556283, EPI_ISL_2556284, EPI_ISL_2556285, EPI_ISL_2556286, EPI_ISL_2556287, EPI_ISL_2556288, EPI_ISL_2556289, EPI_ISL_2556290, EPI_ISL_2556291,
EPI_ISL_2556292, EPI_ISL_2556293, EPI_ISL_2556294, EPI_ISL_2556295, EPI_ISL_2556296, EPI_ISL_2556297, EPI_ISL_2556298, EPI_ISL_2556299, EPI_ISL_2556300, EPI_ISL_2556301, EPI_ISL_2556302, EPI_ISL_2556303, EPI_ISL_2556304, EPI_ISL_2556305, EPI_ISL_2556306, EPI_ISL_2556307, EPI_ISL_2556308, EPI_ISL_2556309, EPI_ISL_2556310, EPI_ISL_2556311, EPI_ISL_2556312, EPI_ISL_2556313,
EPI_ISL_2556314, EPI_ISL_2556315, EPI_ISL_2556316, EPI_ISL_2556317, EPI_ISL_2556318, EPI_ISL_2556319, EPI_ISL_2556320, EPI_ISL_2556321, EPI_ISL_2556322, EPI_ISL_2556323, EPI_ISL_2556324, EPI_ISL_2556325, EPI_ISL_2556326, EPI_ISL_2556327, EPI_ISL_2556328, EPI_ISL_2556329, EPI_ISL_2556330, EPI_ISL_2556331, EPI_ISL_2556332, EPI_ISL_2556333, EPI_ISL_2556334, EPI_ISL_2556335,
EPI_ISL_2556336, EPI_ISL_2556337, EPI_ISL_2556338, EPI_ISL_2556339, EPI_ISL_2556340, EPI_ISL_2556341, EPI_ISL_2556342, EPI_ISL_2556343, EPI_ISL_2556344, EPI_ISL_2556345, EPI_ISL_2556346, EPI_ISL_2556347, EPI_ISL_2556348, EPI_ISL_2556349, EPI_ISL_2556350, EPI_ISL_2556351, EPI_ISL_2556352, EPI_ISL_2556353, EPI_ISL_2556354, EPI_ISL_2556355, EPI_ISL_2556356, EPI_ISL_2556357,
EPI_ISL_2556358, EPI_ISL_2556359, EPI_ISL_2556360, EPI_ISL_2556361, EPI_ISL_2556362, EPI_ISL_2556363, EPI_ISL_2556364, EPI_ISL_2556365, EPI_ISL_2556366, EPI_ISL_2556367, EPI_ISL_2556368, EPI_ISL_2556369, EPI_ISL_2556370, EPI_ISL_2556371, EPI_ISL_2556372, EPI_ISL_2556373, EPI_ISL_2556374, EPI_ISL_2556375, EPI_ISL_2556376, EPI_ISL_2556377, EPI_ISL_2556378, EPI_ISL_2556379,
EPI_ISL_2556380, EPI_ISL_2556381, EPI_ISL_2556382, EPI_ISL_2556383, EPI_ISL_2556384, EPI_ISL_2556385, EPI_ISL_2556386, EPI_ISL_2556387, EPI_ISL_2556388, EPI_ISL_2556389, EPI_ISL_2556390, EPI_ISL_2556391, EPI_ISL_2556392, EPI_ISL_2556393, EPI_ISL_2556394, EPI_ISL_2556395, EPI_ISL_2556396, EPI_ISL_2556397, EPI_ISL_2556398, EPI_ISL_2556399, EPI_ISL_2556400, EPI_ISL_2556401,
EPI_ISL_2556402, EPI_ISL_2556403, EPI_ISL_2556404, EPI_ISL_2556405, EPI_ISL_2556406, EPI_ISL_2556407, EPI_ISL_2556408, EPI_ISL_2556418, EPI_ISL_2556419, EPI_ISL_2556420, EPI_ISL_2556421, EPI_ISL_2556422, EPI_ISL_2556423, EPI_ISL_2556424, EPI_ISL_2556425, EPI_ISL_2556426, EPI_ISL_2556427, EPI_ISL_2556428, EPI_ISL_2556429, EPI_ISL_2556430, EPI_ISL_2556431, EPI_ISL_2556432,
EPI_ISL_2556433, EPI_ISL_2556434, EPI_ISL_2556435, EPI_ISL_2556436, EPI_ISL_2556437, EPI_ISL_2556438, EPI_ISL_2556439, EPI_ISL_2556440, EPI_ISL_2556441, EPI_ISL_2556442, EPI_ISL_2556443, EPI_ISL_2556444, EPI_ISL_2556445, EPI_ISL_2556446, EPI_ISL_2556447, EPI_ISL_2556448, EPI_ISL_2556449, EPI_ISL_2556450, EPI_ISL_2556451, EPI_ISL_2556452, EPI_ISL_2556453, EPI_ISL_2556454,
EPI_ISL_2556455, EPI_ISL_2556456, EPI_ISL_2556457, EPI_ISL_2556458, EPI_ISL_2556459, EPI_ISL_2556460, EPI_ISL_2556461, EPI_ISL_2556462, EPI_ISL_2556463, EPI_ISL_2556464, EPI_ISL_2556465, EPI_ISL_2556466, EPI_ISL_2556467, EPI_ISL_2556468, EPI_ISL_2556469, EPI_ISL_2556470, EPI_ISL_2556471, EPI_ISL_2556472, EPI_ISL_2556473, EPI_ISL_2556474, EPI_ISL_2556475, EPI_ISL_2556476,
EPI_ISL_2556477, EPI_ISL_2556478, EPI_ISL_2556479, EPI_ISL_2556480, EPI_ISL_2556481, EPI_ISL_2556482, EPI_ISL_2556483, EPI_ISL_2556484, EPI_ISL_2556485, EPI_ISL_2556486, EPI_ISL_2556487, EPI_ISL_2556488, EPI_ISL_2556489, EPI_ISL_2556490, EPI_ISL_2556491, EPI_ISL_2556492, EPI_ISL_2556493, EPI_ISL_2556494, EPI_ISL_2556495, EPI_ISL_2556496, EPI_ISL_2556497, EPI_ISL_2556498,
EPI_ISL_2556499, EPI_ISL_2556500, EPI_ISL_2556501, EPI_ISL_2556502, EPI_ISL_2556503, EPI_ISL_2556504, EPI_ISL_2556505, EPI_ISL_2556506, EPI_ISL_2556507, EPI_ISL_2556508, EPI_ISL_2556509, EPI_ISL_2556510, EPI_ISL_2556511, EPI_ISL_2556512, EPI_ISL_2556513, EPI_ISL_2556514, EPI_ISL_2556515, EPI_ISL_2556516, EPI_ISL_2556517, EPI_ISL_2556518, EPI_ISL_2556519, EPI_ISL_2556520,
EPI_ISL_2556521, EPI_ISL_2556522, EPI_ISL_2556523, EPI_ISL_2556524, EPI_ISL_2556525, EPI_ISL_2556526, EPI_ISL_2556527, EPI_ISL_2556528, EPI_ISL_2556529, EPI_ISL_2556530, EPI_ISL_2556531, EPI_ISL_2556532, EPI_ISL_2556533, EPI_ISL_2556534, EPI_ISL_2556535, EPI_ISL_2556536, EPI_ISL_2556537, EPI_ISL_2556538, EPI_ISL_2556539, EPI_ISL_2556540, EPI_ISL_2556541, EPI_ISL_2556542,
EPI_ISL_2556543, EPI_ISL_2556544, EPI_ISL_2556545, EPI_ISL_2556546, EPI_ISL_2556547, EPI_ISL_2556548, EPI_ISL_2556549, EPI_ISL_2556551, EPI_ISL_2556552, EPI_ISL_2556553, EPI_ISL_2556554, EPI_ISL_2556556, EPI_ISL_2556557, EPI_ISL_2556558, EPI_ISL_2556559, EPI_ISL_2556560, EPI_ISL_2556561, EPI_ISL_2556562, EPI_ISL_2556563, EPI_ISL_2556564, EPI_ISL_2556565, EPI_ISL_2556566,
EPI_ISL_2556567, EPI_ISL_2556568, EPI_ISL_2556569, EPI_ISL_2556570, EPI_ISL_2556571, EPI_ISL_2556572, EPI_ISL_2556573, EPI_ISL_2556574, EPI_ISL_2556575, EPI_ISL_2556576, EPI_ISL_2556577, EPI_ISL_2556578, EPI_ISL_2556579, EPI_ISL_2556580, EPI_ISL_2556581, EPI_ISL_2556582, EPI_ISL_2556583, EPI_ISL_2556584, EPI_ISL_2556585, EPI_ISL_2556586, EPI_ISL_2556587, EPI_ISL_2556588,
EPI_ISL_2556589, EPI_ISL_2556590, EPI_ISL_2556591, EPI_ISL_2556592, EPI_ISL_2556593, EPI_ISL_2556594, EPI_ISL_2556595, EPI_ISL_2556596, EPI_ISL_2556597, EPI_ISL_2556598, EPI_ISL_2556599, EPI_ISL_2556600, EPI_ISL_2556601, EPI_ISL_2556602, EPI_ISL_2556603, EPI_ISL_2556604, EPI_ISL_2556605, EPI_ISL_2556606, EPI_ISL_2556607, EPI_ISL_2556608, EPI_ISL_2556609, EPI_ISL_2556610,
EPI_ISL_2556611, EPI_ISL_2556612, EPI_ISL_2556613, EPI_ISL_2556614, EPI_ISL_2556615, EPI_ISL_2556616, EPI_ISL_2556617, EPI_ISL_2556618, EPI_ISL_2556619, EPI_ISL_2556620, EPI_ISL_2556621, EPI_ISL_2556622, EPI_ISL_2556623, EPI_ISL_2556624, EPI_ISL_2556625, EPI_ISL_2556626, EPI_ISL_2556627, EPI_ISL_2556628, EPI_ISL_2556629, EPI_ISL_2556630, EPI_ISL_2556631, EPI_ISL_2556632,
EPI_ISL_2556633, EPI_ISL_2556634, EPI_ISL_2556635, EPI_ISL_2556636, EPI_ISL_2556637, EPI_ISL_2556638, EPI_ISL_2556639, EPI_ISL_2556640, EPI_ISL_2556641, EPI_ISL_2556642, EPI_ISL_2556643, EPI_ISL_2556644, EPI_ISL_2556645, EPI_ISL_2556646, EPI_ISL_2556647, EPI_ISL_2556648, EPI_ISL_2556649, EPI_ISL_2556650, EPI_ISL_2556651, EPI_ISL_2556652, EPI_ISL_2556653, EPI_ISL_2556654,
EPI_ISL_2556655, EPI_ISL_2556656, EPI_ISL_2556657, EPI_ISL_2556658, EPI_ISL_2556659, EPI_ISL_2556660, EPI_ISL_2556661, EPI_ISL_2556662, EPI_ISL_2556663, EPI_ISL_2556664, EPI_ISL_2556665, EPI_ISL_2556666, EPI_ISL_2556667, EPI_ISL_2556668, EPI_ISL_2556710, EPI_ISL_2556711, EPI_ISL_2556712, EPI_ISL_2556713, EPI_ISL_2556714, EPI_ISL_2556715, EPI_ISL_2556716, EPI_ISL_2556717,
EPI_ISL_2556718, EPI_ISL_2556730, EPI_ISL_2556731, EPI_ISL_2556732, EPI_ISL_2556733, EPI_ISL_2556734, EPI_ISL_2556735, EPI_ISL_2556736, EPI_ISL_2556737, EPI_ISL_2556738, EPI_ISL_2556739, EPI_ISL_2556740, EPI_ISL_2556741, EPI_ISL_2556742, EPI_ISL_2556743, EPI_ISL_2556744, EPI_ISL_2556745, EPI_ISL_2556746, EPI_ISL_2556747, EPI_ISL_2556748, EPI_ISL_2556749, EPI_ISL_2556750,
EPI_ISL_2556751, EPI_ISL_2556752, EPI_ISL_2556753, EPI_ISL_2556754, EPI_ISL_2556755, EPI_ISL_2556756, EPI_ISL_2556757, EPI_ISL_2556758, EPI_ISL_2556759, EPI_ISL_2556760, EPI_ISL_2556761, EPI_ISL_2556762, EPI_ISL_2556763, EPI_ISL_2556764, EPI_ISL_2556766, EPI_ISL_2556767, EPI_ISL_2556768, EPI_ISL_2556769, EPI_ISL_2556770, EPI_ISL_2556771, EPI_ISL_2556772, EPI_ISL_2556833,
EPI_ISL_2556834, EPI_ISL_2556835, EPI_ISL_2556836, EPI_ISL_2556837, EPI_ISL_2556838, EPI_ISL_2556839, EPI_ISL_2556840, EPI_ISL_2556841, EPI_ISL_2556842, EPI_ISL_2556843, EPI_ISL_2556844, EPI_ISL_2556845, EPI_ISL_2556846, EPI_ISL_2556847, EPI_ISL_2556848, EPI_ISL_2556849, EPI_ISL_2556850, EPI_ISL_2556851, EPI_ISL_2556852, EPI_ISL_2556853, EPI_ISL_2556854, EPI_ISL_2556855,
EPI_ISL_2556856, EPI_ISL_2556857, EPI_ISL_2556858, EPI_ISL_2556859, EPI_ISL_2556860, EPI_ISL_2556861, EPI_ISL_2556862, EPI_ISL_2556863, EPI_ISL_2556864, EPI_ISL_2556865, EPI_ISL_2556866, EPI_ISL_2556867, EPI_ISL_2556868, EPI_ISL_2556869, EPI_ISL_2556870, EPI_ISL_2556871, EPI_ISL_2556872, EPI_ISL_2556873, EPI_ISL_2556874, EPI_ISL_2556875, EPI_ISL_2556876, EPI_ISL_2556877,
EPI_ISL_2556878, EPI_ISL_2556879, EPI_ISL_2556880, EPI_ISL_2556881, EPI_ISL_2556882, EPI_ISL_2556883, EPI_ISL_2556884, EPI_ISL_2556885, EPI_ISL_2556886, EPI_ISL_2556887, EPI_ISL_2556888, EPI_ISL_2556889, EPI_ISL_2556890, EPI_ISL_2556891, EPI_ISL_2556892, EPI_ISL_2556893, EPI_ISL_2556894, EPI_ISL_2556895, EPI_ISL_2556896, EPI_ISL_2556897, EPI_ISL_2556898, EPI_ISL_2556899,
EPI_ISL_2556900, EPI_ISL_2556901, EPI_ISL_2556902, EPI_ISL_2556903, EPI_ISL_2556904, EPI_ISL_2556905, EPI_ISL_2556906, EPI_ISL_2556907, EPI_ISL_2556908, EPI_ISL_2556909, EPI_ISL_2556910, EPI_ISL_2556911, EPI_ISL_2556912, EPI_ISL_2556913, EPI_ISL_2556914, EPI_ISL_2556915, EPI_ISL_2556916, EPI_ISL_2556917, EPI_ISL_2556918, EPI_ISL_2556919, EPI_ISL_2556920, EPI_ISL_2556921,
EPI_ISL_2556922, EPI_ISL_2556923, EPI_ISL_2556924, EPI_ISL_2556925, EPI_ISL_2556926, EPI_ISL_2556927, EPI_ISL_2556928, EPI_ISL_2556929, EPI_ISL_2556930, EPI_ISL_2556931, EPI_ISL_2556932, EPI_ISL_2556933, EPI_ISL_2556934, EPI_ISL_2556935, EPI_ISL_2556936, EPI_ISL_2556937, EPI_ISL_2556938, EPI_ISL_2556939, EPI_ISL_2556940, EPI_ISL_2556941, EPI_ISL_2556942, EPI_ISL_2556943,
EPI_ISL_2556944, EPI_ISL_2556945, EPI_ISL_2556946, EPI_ISL_2556947, EPI_ISL_2556948, EPI_ISL_2556949, EPI_ISL_2556950, EPI_ISL_2556951, EPI_ISL_2556952, EPI_ISL_2556953, EPI_ISL_2556954, EPI_ISL_2556955, EPI_ISL_2556956, EPI_ISL_2556957, EPI_ISL_2556958, EPI_ISL_2556959, EPI_ISL_2556960, EPI_ISL_2556961, EPI_ISL_2556962, EPI_ISL_2556963, EPI_ISL_2556964, EPI_ISL_2556965,
EPI_ISL_2556966, EPI_ISL_2556967, EPI_ISL_2556968, EPI_ISL_2556969, EPI_ISL_2556970, EPI_ISL_2556971, EPI_ISL_2556972, EPI_ISL_2556973, EPI_ISL_2556974, EPI_ISL_2556975, EPI_ISL_2556976, EPI_ISL_2556977, EPI_ISL_2556978, EPI_ISL_2556979, EPI_ISL_2556980, EPI_ISL_2556981, EPI_ISL_2556982, EPI_ISL_2556983, EPI_ISL_2556984, EPI_ISL_2556985, EPI_ISL_2556986, EPI_ISL_2556987,
EPI_ISL_2556988, EPI_ISL_2556989, EPI_ISL_2556990, EPI_ISL_2556991, EPI_ISL_2556992, EPI_ISL_2556993, EPI_ISL_2556994, EPI_ISL_2556995, EPI_ISL_2556996, EPI_ISL_2556997, EPI_ISL_2556998, EPI_ISL_2556999, EPI_ISL_2557000, EPI_ISL_2557001, EPI_ISL_2557002, EPI_ISL_2557003, EPI_ISL_2557004, EPI_ISL_2557005, EPI_ISL_2557006, EPI_ISL_2557007, EPI_ISL_2557008, EPI_ISL_2557009,
EPI_ISL_2557010, EPI_ISL_2557011, EPI_ISL_2557012, EPI_ISL_2557013, EPI_ISL_2557014, EPI_ISL_2557015, EPI_ISL_2557016, EPI_ISL_2557017, EPI_ISL_2557018, EPI_ISL_2557019, EPI_ISL_2557020, EPI_ISL_2557021, EPI_ISL_2557022, EPI_ISL_2557023, EPI_ISL_2557024, EPI_ISL_2557025, EPI_ISL_2557026, EPI_ISL_2557027, EPI_ISL_2557028, EPI_ISL_2557029, EPI_ISL_2557030, EPI_ISL_2557031,
EPI_ISL_2557032, EPI_ISL_2557033, EPI_ISL_2955675, EPI_ISL_2955676, EPI_ISL_2955677, EPI_ISL_2955678, EPI_ISL_2955679, EPI_ISL_2955680, EPI_ISL_2955681, EPI_ISL_2955682, EPI_ISL_2955683, EPI_ISL_2955684, EPI_ISL_2955685, EPI_ISL_2955686, EPI_ISL_2955687, EPI_ISL_2955688, EPI_ISL_2955689, EPI_ISL_2955690, EPI_ISL_2955691, EPI_ISL_2955692, EPI_ISL_2955693, EPI_ISL_2955694,
EPI_ISL_2955695, EPI_ISL_2955696, EPI_ISL_2955697, EPI_ISL_2955698, EPI_ISL_2955699, EPI_ISL_2955700, EPI_ISL_2955701, EPI_ISL_2955702, EPI_ISL_2955703, EPI_ISL_2955704, EPI_ISL_2955705, EPI_ISL_2955706, EPI_ISL_2955707, EPI_ISL_2955708, EPI_ISL_2955709, EPI_ISL_2955710, EPI_ISL_2955711, EPI_ISL_2955712, EPI_ISL_2955713, EPI_ISL_2955769, EPI_ISL_2955770, EPI_ISL_2955771,
EPI_ISL_2955772, EPI_ISL_2955773, EPI_ISL_2955774, EPI_ISL_2955775, EPI_ISL_2955776, EPI_ISL_2955777, EPI_ISL_2955778, EPI_ISL_2955779, EPI_ISL_2955780, EPI_ISL_2955781, EPI_ISL_2955782, EPI_ISL_2955783, EPI_ISL_2955784, EPI_ISL_2955836, EPI_ISL_2955837, EPI_ISL_2955838, EPI_ISL_2955839, EPI_ISL_2955840, EPI_ISL_2955841, EPI_ISL_2955842, EPI_ISL_2955843, EPI_ISL_2955844,
EPI_ISL_2955845, EPI_ISL_2955846, EPI_ISL_2955847, EPI_ISL_2955848, EPI_ISL_2955849, EPI_ISL_2955850, EPI_ISL_2955851, EPI_ISL_2955852, EPI_ISL_2955853, EPI_ISL_2955854, EPI_ISL_2955855, EPI_ISL_2955856, EPI_ISL_3066838

see above	National Centre for Disease Control (NCDC) Biotechnology Division, Delhi	NCDC Delhi, Biotechnology Division INSACOG	Hema Gogia; Hemlata Lall; Kalaiarasan Ponnusamy; Mahesh S Dhar; Manoj K Singh; Meena Datta; Partha Rakshit; Preeti Madan; Priyanka Singh; Radhakrishnan V. S; Robin Marwal; Sandhya Kabra; Sujeet K Singh; Uma Sharma
EPI_ISL_436423, EPI_ISL_436424, EPI_ISL_436425, EPI_ISL_436426, EPI_ISL_436427, EPI_ISL_436428, EPI_ISL_436429, EPI_ISL_436430, EPI_ISL_436431, EPI_ISL_436432, EPI_ISL_436433, EPI_ISL_436434, EPI_ISL_436435, EPI_ISL_436436, EPI_ISL_436437, EPI_ISL_436445, EPI_ISL_436448, EPI_ISL_436450, EPI_ISL_436451, EPI_ISL_436452, EPI_ISL_436454, EPI_ISL_436455, EPI_ISL_482491, EPI_ISL_482492, EPI_ISL_482493, EPI_ISL_482494, EPI_ISL_482495, EPI_ISL_482496, EPI_ISL_482497, EPI_ISL_482498, EPI_ISL_482499, EPI_ISL_482500, EPI_ISL_482501, EPI_ISL_482502, EPI_ISL_482503, EPI_ISL_482504, EPI_ISL_482505, EPI_ISL_482506, EPI_ISL_482507, EPI_ISL_482508, EPI_ISL_482509, EPI_ISL_482510, EPI_ISL_482511, EPI_ISL_482512, EPI_ISL_482513, EPI_ISL_482514, EPI_ISL_482515, EPI_ISL_482516, EPI_ISL_482517, EPI_ISL_482518, EPI_ISL_482519, EPI_ISL_482520, EPI_ISL_482521, EPI_ISL_482522, EPI_ISL_482523, EPI_ISL_482524, EPI_ISL_482525, EPI_ISL_482526, EPI_ISL_482527, EPI_ISL_482528, EPI_ISL_482529, EPI_ISL_482530, EPI_ISL_482531, EPI_ISL_482532, EPI_ISL_482533, EPI_ISL_482534, EPI_ISL_482535, EPI_ISL_482536, EPI_ISL_482537, EPI_ISL_482538, EPI_ISL_482539, EPI_ISL_482540, EPI_ISL_482541, EPI_ISL_482542, EPI_ISL_482543, EPI_ISL_482544, EPI_ISL_482545, EPI_ISL_482546, EPI_ISL_482547, EPI_ISL_482548, EPI_ISL_482549, EPI_ISL_482550, EPI_ISL_482551, EPI_ISL_482552, EPI_ISL_482553, EPI_ISL_482554, EPI_ISL_482555, EPI_ISL_482556, EPI_ISL_482557, EPI_ISL_482558, EPI_ISL_482559, EPI_ISL_482560, EPI_ISL_482561, EPI_ISL_482562, EPI_ISL_482563, EPI_ISL_482564, EPI_ISL_482565, EPI_ISL_482566, EPI_ISL_482567, EPI_ISL_482568, EPI_ISL_482569, EPI_ISL_482570, EPI_ISL_482571, EPI_ISL_482572, EPI_ISL_482573, EPI_ISL_482574, EPI_ISL_482587, EPI_ISL_482588, EPI_ISL_482589, EPI_ISL_482590, EPI_ISL_482591, EPI_ISL_482592, EPI_ISL_482593, EPI_ISL_482594, EPI_ISL_482595, EPI_ISL_482596, EPI_ISL_482597, EPI_ISL_482598, EPI_ISL_482599, EPI_ISL_482600, EPI_ISL_482601, EPI_ISL_482602, EPI_ISL_482603, EPI_ISL_482604, EPI_ISL_482605, EPI_ISL_482606, EPI_ISL_482607, EPI_ISL_482608, EPI_ISL_482609, EPI_ISL_482610, EPI_ISL_482611, EPI_ISL_482612, EPI_ISL_482613, EPI_ISL_482614, EPI_ISL_482615, EPI_ISL_482616, EPI_ISL_482617, EPI_ISL_482618, EPI_ISL_482619, EPI_ISL_482620, EPI_ISL_482621, EPI_ISL_482622, EPI_ISL_482623, EPI_ISL_482624, EPI_ISL_482625, EPI_ISL_482626, EPI_ISL_482627, EPI_ISL_482628, EPI_ISL_482629, EPI_ISL_482630, EPI_ISL_482631, EPI_ISL_482632, EPI_ISL_482633, EPI_ISL_482634, EPI_ISL_482635, EPI_ISL_482636, EPI_ISL_482637, EPI_ISL_482638, EPI_ISL_482639, EPI_ISL_482640, EPI_ISL_482641, EPI_ISL_482642, EPI_ISL_482643, EPI_ISL_482644, EPI_ISL_482645, EPI_ISL_482646, EPI_ISL_482647, EPI_ISL_482648, EPI_ISL_482649, EPI_ISL_482650, EPI_ISL_482651, EPI_ISL_482652, EPI_ISL_482653, EPI_ISL_482654, EPI_ISL_482655, EPI_ISL_482656, EPI_ISL_482657, EPI_ISL_482658, EPI_ISL_482659, EPI_ISL_482660, EPI_ISL_482661, EPI_ISL_482662, EPI_ISL_482663, EPI_ISL_482664, EPI_ISL_482665, EPI_ISL_482666, EPI_ISL_482667, EPI_ISL_482668, EPI_ISL_482669, EPI_ISL_482670, EPI_ISL_482671, EPI_ISL_636738, EPI_ISL_636740, EPI_ISL_636741, EPI_ISL_636745, EPI_ISL_636749, EPI_ISL_636751, EPI_ISL_636752, EPI_ISL_636754, EPI_ISL_636755, EPI_ISL_636757, EPI_ISL_636758, EPI_ISL_636760, EPI_ISL_636761, EPI_ISL_636762, EPI_ISL_636763, EPI_ISL_636764, EPI_ISL_636765, EPI_ISL_636766, EPI_ISL_636767, EPI_ISL_636768, EPI_ISL_636769, EPI_ISL_636770, EPI_ISL_636771, EPI_ISL_636774, EPI_ISL_636775, EPI_ISL_636776, EPI_ISL_636777, EPI_ISL_636778, EPI_ISL_636779, EPI_ISL_636780, EPI_ISL_636787, EPI_ISL_636788, EPI_ISL_636789, EPI_ISL_636790, EPI_ISL_636795, EPI_ISL_636802, EPI_ISL_636803, EPI_ISL_636809, EPI_ISL_636814, EPI_ISL_636815, EPI_ISL_636816, EPI_ISL_636817, EPI_ISL_636818, EPI_ISL_636819, EPI_ISL_636820, EPI_ISL_636821, EPI_ISL_636825, EPI_ISL_636832, EPI_ISL_636833			
see above	National Centre for Disease control (NCDC)	NCDC/CSIR-IGIB	Aarti Tewari; Ajit Shewale; Anurag Agrawal#; Anurag Agrawal*; Aparna Swaminathan; Asangla Kamai; Bharathram Uppili; Bharathram Uppili2*; Bibhash Nandi; Debasis Dash; Dhirendra Kumar; Hema Gogia; Hemlata Lall; Himanshu Vashisht; Ishtaq Ahmed; Mahesh S Dhar; Mahesh S. Dhar1*; Manish Kumar; Manju Bala; Meena Datta; Mitali Mukerji; Mohammed Faruq; Mohammed Faruq#; Nidhi Saini; Nishu Tyagi; Partha Rakshit#; Partha Rakshit*; Pooja Sharma; Pooja Sharma2*; Poonam Gupta; Pramod Kumar#; Prateek Singh; Preeti Madan; Priyanka Singh; RadhaKrishnan VS; Radhakrishanan VS; Rajesh Pandey#; Robin Marwal; Robin Marwal1*; Sandhya Kabra; Saruchi Wadhwa; Satyabrata Bag; Shaista Khan; Simmi Tiwari; Simrita Singh; Sujeet Singh; Tushar Nale; Uma Sharma; Varun Jaiswal; Vivekanand A
EPI_ISL_2955785, EPI_ISL_2955786, EPI_ISL_2955787, EPI_ISL_2955788, EPI_ISL_2955789, EPI_ISL_2955790, EPI_ISL_2955791, EPI_ISL_2955792, EPI_ISL_2955793, EPI_ISL_2955794, EPI_ISL_2955795, EPI_ISL_2955796, EPI_ISL_2955797, EPI_ISL_2955798, EPI_ISL_2955799, EPI_ISL_2955800, EPI_ISL_2955801, EPI_ISL_2955802, EPI_ISL_2955803, EPI_ISL_2955804, EPI_ISL_2955805, EPI_ISL_2955806, EPI_ISL_2955807, EPI_ISL_2955808, EPI_ISL_2955809, EPI_ISL_2955810, EPI_ISL_2955811, EPI_ISL_2955812, EPI_ISL_2955813, EPI_ISL_2955814, EPI_ISL_2955815, EPI_ISL_2955816, EPI_ISL_2955817, EPI_ISL_2955818, EPI_ISL_2955819, EPI_ISL_2955820, EPI_ISL_2955821, EPI_ISL_2955822, EPI_ISL_2955823, EPI_ISL_2955824, EPI_ISL_2955825, EPI_ISL_2955826, EPI_ISL_2955827, EPI_ISL_2955828, EPI_ISL_2955829, EPI_ISL_2955830, EPI_ISL_2955831, EPI_ISL_2955832, EPI_ISL_2955833, EPI_ISL_2955834, EPI_ISL_2955835, EPI_ISL_3066839, EPI_ISL_3066840, EPI_ISL_3066841, EPI_ISL_3066842, EPI_ISL_3066843, EPI_ISL_3066844, EPI_ISL_3066845, EPI_ISL_3066846, EPI_ISL_3066847, EPI_ISL_3066848, EPI_ISL_3066849, EPI_ISL_3066850, EPI_ISL_3066851, EPI_ISL_3066852, EPI_ISL_3066853			
see above	Northern Railway Central Hospital, New Delhi, India	NCDC Delhi, Biotechnology Division INSACOG	Hema Gogia; Hemlata Lall; Kalaiarasan Ponnusamy; Mahesh S Dhar; Manoj K Singh; Meena Datta; Partha Rakshit; Preeti Madan; Priyanka Singh; Radhakrishnan V. S; Robin Marwal; Sandhya Kabra; Sujeet K Singh; Uma Sharma
EPI_ISL_2001166, EPI_ISL_2001167, EPI_ISL_2001175, EPI_ISL_2001176	PanGenomics International Pvt. Ltd.	Gujarat Biotechnology Research Centre	Chaitanya Joshi; Dinesh Kumar; Dipali Dhawan; Janvi Raval; Madhvi Joshi; Nitesh Shah; Nitin Savaliya; Ramesh Pandit; Sonal Sharma; Twinkle Soni; Umang Mishra; Zarna Patel; Zuber Saiyed
EPI_ISL_1844689	SAROJ HOSPITAL	Biotechnology Division, NCDC	Hema Gogia; Hemlata Lall; Kalaiarasan Ponnusamy; Mahesh S Dhar; Manoj K Singh; Meena Datta; Partha Rakshit; Preeti Madan; Priyanka Singh; Radhakrishnan V. S; Robin Marwal; Sandhya Kabra; Sujeet K Singh; Uma Sharma
EPI_ISL_2967203, EPI_ISL_2967204, EPI_ISL_2967205, EPI_ISL_2967206, EPI_ISL_2967207, EPI_ISL_2967208, EPI_ISL_2967209, EPI_ISL_2967210, EPI_ISL_2967211, EPI_ISL_2967212, EPI_ISL_2967213, EPI_ISL_2967214, EPI_ISL_2967215, EPI_ISL_2967216, EPI_ISL_2967217			
see above	Sri Ganga Ram Hospital, New Delhi, India	NCDC Delhi, Biotechnology Division	Hema Gogia; Hemlata Lall; Kalaiarasan Ponnusamy; Mahesh S Dhar; Manoj K Singh; Meena Datta; Partha Rakshit; Preeti Madan; Priyanka Singh; Radhakrishnan V. S; Robin Marwal; Sandhya Kabra; Sujeet K Singh; Uma Sharma
EPI_ISL_508495, EPI_ISL_653879	Translational Health Science and Technology Institute	National Institute of Biomedical Genomics	Anbalagan Ananthraj; Arindam Maitra; Gagandeep Kang; Guruprasad Medigeshi; Imran Khan; Madhu Pareek; Saumitra Das; Sharanabasava Patil
EPI_ISL_2033743, EPI_ISL_2033745, EPI_ISL_2033747, EPI_ISL_2350786, EPI_ISL_2350787, EPI_ISL_2350788, EPI_ISL_2350789, EPI_ISL_2350790, EPI_ISL_3102105			
see above	Translational Health Science and Technology Institute -ESIC medical college and hospital, Faridabad	THSTI Bioassay laboratory	Akshay Kanakan; Anil Kumar Pandey; Anil Pandey; Anish Maitra; Guruprasad R Medigeshi; Heena Shaman; Janani Srinivasa Vasudevan; Janmejay Singh; Jigme Wangchuk; Kamal Pargai; Naseem Ahmed Khan; Neha Jha; Partha Chattopadhyay; Priyanka Mehta; Rajesh Pandey; Ranjeet Maurya; Saurabh Kumar
EPI_ISL_1844738, EPI_ISL_1844743, EPI_ISL_1844747, EPI_ISL_1844752, EPI_ISL_1844756, EPI_ISL_1844761	VIRAL RESEARCH & DIAGNOSTIC LAB, GMC	Biotechnology Division, NCDC	Hema Gogia; Hemlata Lall; Kalaiarasan Ponnusamy; Mahesh S Dhar; Manoj K Singh; Meena Datta; Partha Rakshit; Preeti Madan; Priyanka Singh; Radhakrishnan V. S; Robin Marwal; Sandhya Kabra; Sujeet K Singh; Uma Sharma
EPI_ISL_1844715, EPI_ISL_1844720, EPI_ISL_1844724, EPI_ISL_1844729, EPI_ISL_1844733	Viral Testing Laboratory, GADVASU	Biotechnology Division, NCDC	Hema Gogia; Hemlata Lall; Kalaiarasan Ponnusamy; Mahesh S Dhar; Manoj K Singh; Meena Datta; Partha Rakshit; Preeti Madan; Priyanka Singh; Radhakrishnan V. S; Robin Marwal; Sandhya Kabra; Sujeet K Singh; Uma Sharma

We gratefully acknowledge the following Authors from the Originating laboratories responsible for obtaining the specimens, as well as the Submitting laboratories where the genome data were generated and shared via GISAID, on which this research is based.

All Submitters of data may be contacted directly via www.gisaid.org

Authors are sorted alphabetically.

Accession ID	Originating Laboratory	Submitting Laboratory	Authors
EPI_ISL_2458629, EPI_ISL_2458636, EPI_ISL_2458668, EPI_ISL_2459670, EPI_ISL_2459748, EPI_ISL_2459785, EPI_ISL_2459881, EPI_ISL_2459882, EPI_ISL_2460663, EPI_ISL_2461529, EPI_ISL_2461623, EPI_ISL_2461724, EPI_ISL_2461725, EPI_ISL_2461726, EPI_ISL_2461727, EPI_ISL_2461762, EPI_ISL_2461790, EPI_ISL_2461937, EPI_ISL_2461995, EPI_ISL_2461996, EPI_ISL_2461997, EPI_ISL_2461998, EPI_ISL_2461999, EPI_ISL_2462000, EPI_ISL_2462001, EPI_ISL_2502060, EPI_ISL_2502098, EPI_ISL_2502106, EPI_ISL_2502133, EPI_ISL_2502151, EPI_ISL_2502172, EPI_ISL_2502178, EPI_ISL_2502185, EPI_ISL_2502196, EPI_ISL_2502242, EPI_ISL_2502247, EPI_ISL_2504346, EPI_ISL_2555969, EPI_ISL_2555970, EPI_ISL_2556409, EPI_ISL_2556410, EPI_ISL_2556411, EPI_ISL_2556412, EPI_ISL_2556413, EPI_ISL_2556414, EPI_ISL_2556415, EPI_ISL_2556416, EPI_ISL_2556417, EPI_ISL_2556719, EPI_ISL_2556720, EPI_ISL_2556721, EPI_ISL_2556722, EPI_ISL_2556723, EPI_ISL_2556724, EPI_ISL_2556725, EPI_ISL_2556726, EPI_ISL_2556727, EPI_ISL_2556728, EPI_ISL_2556729, EPI_ISL_2955672, EPI_ISL_2955673, EPI_ISL_2955674			
see above	National Centre for Disease Control (NCDC) Biotechnology Division, Delhi	NCDC Delhi, Biotechnology Division INSACOG	Hema Gogia; Hemlata Lall; Kalaiarasan Ponnusamy; Mahesh S Dhar; Manoj K Singh; Meena Datta; Partha Rakshit; Preeti Madan; Priyanka Singh; Radhakrishnan V. S; Robin Marwal; Sandhya Kabra; Sujeet K Singh; Uma Sharma
