# Supplementary material for: Genomic characterization and epidemiology of an emerging SARS-CoV-2 variant in Delhi, India
Source: Science. 2021 Oct 14;374(6570):995–9. doi: 10.1126/science.abj9932 (PMC7612010; doi:10.1126/science.abj9932)
Supplement: Supplementary file 4 — Data S1 to S9 [file science.abj9932_data_s1_to_s9.zip › science.abj9932_data_s1_to_s9_captions.pdf]

**Data S1 (separate file)**

GISAID accession IDs, date of collection and lineages of the samples used to calculate lineage proportions in different states from Datasets A and B.

**Data S2 (separate file)**

Cases, Tests, Hospitalizations, ICU Admissions, Deaths data for Delhi from the state level database maintained by NCDC.

**Data S3 (separate file)**

Acknowledgement table for genomes accessed from GISAID.

**Data S4 (separate file)**

Details of 24 post-vaccination samples

**Data S5 (separate file)**

Raw Ct Values for July 2020-June 2021 for Delhi

**Data S6 (separate file)**

Serosurvey data for the three phases conducted in Delhi (Fig. 2A)

**Data S7 (separate file)**

sVNT assay data Phase I vs Phase II serosurvey (Fig. 2D)

**Data S8 (separate file)**

Data for Subjects with Reinfection (Fig. 2C)

**Data S9 (separate file)**

Data for Subjects without Reinfection (Fig. 2D)
