# Supplementary material for: Genomic characterization and Epidemiology of an emerging SARS-CoV-2 variant in Delhi, India
Source: Science. Author manuscript; Available in PMC 2021 Nov 19. (PMC7612010; doi:10.1126/science.abj9932)
Supplement: Supplementary file 1 [file EMS137316-supplement-Supplementary_Materials.pdf]

**Supplementary Materials**

Materials and Methods

Supplementary Text

Figs. S1 to S5

Tables S1 to S3

List of INSACOG Consortium Members

Data S1 to S9

References (21-44)

Science
AAAS

# Supplementary Materials for

## Genomic characterization and Epidemiology of an emerging SARS-CoV-2 variant in Delhi, India

Mahesh S Dhar#, Robin Marwal#, Radhakrishnan VS#, Kalaiarasan Ponnusamy#, Bani Jolly#, Rahul C. Bhoyar#, Viren Sardana, Salwa Naushin, Mercy Rophina, Thomas A Mellan, Swapnil Mishra, Charlie Whittaker, Saman Fatihi, Meena Datta, Priyanka Singh, Uma Sharma, Rajat Ujjainiya, Nitin Bhatheja, Mohit Kumar Divakar, Manoj K Singh, Mohamed Imran, Vigneshwar Senthivel, Ranjeet Maurya, Neha Jha, Priyanka Mehta, Vivekanand A, Pooja Sharma, Arvinden VR, Urmila Chaudhary, Namita Soni, Lipi Thukral, Seth Flaxman, Samir Bhatt, Rajesh Pandey, Debasis Dash, Mohammed Faruq, Hemlata Lall, Hema Gogia, Preeti Madan, Sanket Kulkarni, Himanshu Chauhan, Shantanu Sengupta, Sandhya Kabra, The Indian SARS-CoV-2 Genomics Consortium (INSACOG),
Ravindra K. Gupta, Sujeet K Singh, Anurag Agrawal*, Partha Rakshit*

#Equal contribution

*Correspondence to: partho_rakshit@yahoo.com (Partha Rakshit), a.agrawal@igib.in (Anurag Agrawal)

**This PDF file includes:**

Materials and Methods
Supplementary Text
Figs. S1 to S5
Tables S1 to S3
List of INSACOG Consortium Members
Data S1 to S9

**Materials and Methods**

Ethics

All work conducted under this study was approved by Institutional Ethics Committees at NCDC and CSIR-IGIB. Testing and sequencing of positive samples for genomic surveillance is exempted from individual informed consent, being a mandated public health service of NCDC and CSIR-IGIB for public health purposes. The use of deidentified data generated through clinical and public health services for research was reviewed and approved under certificates CSIR-IGIB/IHEC/2020-21/01 Dt. 28.03.2020, and *NCDC Ethical review committee* No: 2020/NERC/14. The serosurvey was approved under certificate CSIR-IGIB/IHEC/2020-21/01 Dt. 28.03.2020 and CSIR-IGIB/IHEC/2020-21/02 Dt. 23.02.2021, for project entitled "Phenome India - A long-term longitudinal observational cohort study of health outcomes" with individual informed consent from each participant (4).

Sampling and Metadata Collection

Nasopharyngeal and throat swab samples from COVID-19 confirmed cases with Ct value < 25 were collected and transported to Biotechnology Division, National Centre for Disease Control (NCDC), New Delhi, from the various testing sites across different states in India as per the sampling strategy of Central Surveillance Unit (CSU) of Integrated Disease Surveillance Programme (IDSP), NCDC. 24 post-vaccination RT-PCR confirmed SARS-CoV-2 positive samples were also included in this study (Data S4). All patient details and metadata were filled on the patient identification form and were accompanied with the samples. A total of 11,335 samples were received for whole genome sequencing at NCDC between November 2020 to May 2021 which were processed for viral genome sequencing.

PCR Amplification, Viral Genome Sequencing and Assembly

Viral RNA was isolated from the patient samples using MagNA Pure RNA extraction system (Roche) following the manufacturer's instructions. Whole-genome sequencing of the viral isolates was done as per the COVIDSeq protocol by Illumina using the NextSeq 550 platform. A total of 376 samples per lot were processed in batches of 94 with indexes A-D by loading 1.4 pmol of the library on the 75 cycle High Output Kit flow cell. Approximately 20GB of data was generated by genome sequencing which was processed using the Illumina DRAGEN COVID Pipeline and DRAGEN COVID Lineage Tools (v3.5.1). The raw data sequencing data generated in binary base call format (BCL) from the NextSeq 550 instrument was demultiplexed to FASTQ files using bcl2fastq (Illumina, v2.20). The raw reads were aligned against the SARS-CoV-2 reference genome (NC_045512.2) following the pipeline (21). The minimum accepted alignment score was set to 12 and alignment results with scores <12 were discarded. The coverage threshold and virus detection threshold were set to 20 and 5 respectively. The variant calling target coverage which specifies the maximum number of reads with a start position overlapping any given position was set at 50. The consensus sequences generated for the samples at the end of the DRAGEN COVID Pipeline were used for downstream analysis including lineage assignment and phylogenetic analysis. Out of the 9,557 genome sequences generated, 7,858 sequences with complete metadata were used for further analysis.

Genome Datasets and Lineage Analysis

Two datasets were compiled to estimate the lineage frequency of SARS-CoV-2 for the state of Delhi and other surrounding states and union territories (UTs) in North India: Punjab, Haryana, Uttar

Pradesh, Chandigarh, Himachal Pradesh, Uttarakhand, Jammu and Kashmir, and Ladakh (Data S1). Dataset A comprises 7,858 genome sequencing data generated in this study. Dataset B comprises SARS-CoV-2 genome sequences from these states publicly available in GISAID (with collection dates up to 30th June 2021). Only those sequences from GISAID having complete date of collection (YYYY-MM-DD) were included in Dataset B and appended to Dataset A. For Dataset A, lineages were assigned to the genome sequences using the Pangolin tool (15,16) (version 3.1.8, pangoLEARN version 2021-07-28) to match the current version of lineage assignments on GISAID. The lineage data was segregated according to states and date of collection. State wise frequency of variant of concern B.1.617.2 (Delta) was plotted along with frequencies of B.1.617.1 (Kappa), B.1.1.7 (Alpha), B.1, B.1.36 and 'other variants'. For Delhi, proportions of the different lineages were calculated as weekly aggregates and for the period April 2020 to June 2021 and plotted in context with the weekly aggregates of the number of new cases of COVID-19 reported from the state and test positivity rate. The dataset of number of tests, confirmed cases and positivity rate for the state of Delhi was taken from the state level database maintained at NCDC (Data S2). For states other than Delhi, sequences with dates of collection between 1 November 2020 and 30 June 2021 were used to analyze lineage frequencies of the virus aggregated monthly. The dataset of the number of tests and confirmed cases for other states was accessed from https://covidtoday.github.io/backend/. A table describing the GISAID accession IDs, date of collection and lineages of the samples used to calculate lineage proportions in different states from Datasets A and B is given as Data S1. The acknowledgement table for genome sequences downloaded from GISAID is given as Data S3. All FASTA sequences included as Dataset A are available at https://github.com/banijolly/ncov-Delhi-Epidemiology (DOI:10.5281/zenodo.5521073). Details for the 24 post-vaccination samples and the respective genome sequence IDs are given in Data S4. Raw Ct values for genes ORF1a (Target 1) and E (Target 2) analysed for the time period between July 2020 – June 2021 is available as Data S5.

Phylogenetic Analysis

1787 genome sequences from the state of Delhi were used for phylogenetic analysis along with an additional 152 B.1.617.2 (Delta) and 271 B.1.1.7 (Alpha) genomes from the state of Maharashtra and Punjab respectively. The phylogenetic tree was constructed following the Nextstrain protocol for genetic epidemiology of SARS-CoV-2 (https://nextstrain.github.io/ncov/) (22,23). Briefly, the genome sequences were aligned against the SARS-CoV-2 reference genome MN908947 (21) using NextAlign (22) using the default parameters of the Nextstrain protocol. The first 100 and last 50 bases were masked in the resulting alignment file before further processing. A fast maximum likelihood phylogenetic tree was constructed using IQTREE2 (24) using a general time reversible model (GTR) by specifying a log-likelihood epsilon value of 0.05 for final model parameter estimation. The tree was further processed to resolve polytomies and rerooted to have sample hCoV-19/Wuhan/WH01/2019 (EPI_ISL_406798) as the root. The pipeline further processes the tree using TreeTime (23) to estimate a skyline coalescent using a fixed clock rate of $8x10^{-4}$. The resulting phylogenetic tree was visualized and annotated using the R package ggtree (25). The phylogenetic network of SARS-CoV-2 isolates from Punjab collected during February-March 2021 was visualized in Auspice (fig. S3). The tree files generated by the analysis are available at https://github.com/banijolly/ncov-Delhi-Epidemiology (DOI:10.5281/zenodo.5521073).

Serosurvey

The serosurvey was conducted through a voluntary participation wherein personnel working at CSIR labs/centers and their family members gave their blood samples in July-Sep 2020 (Phase 1) and January-February 2021 (Phase 2) and May-July 2021 (Phase 3) (details are given in table S1

and Data S6). The study was approved by the Institutional Human Ethics Committee of CSIR-IGIB vide approval CSIR-IGIB/IHEC/2019–20 & CSIR-IGIB/IHEC/2020-21/02 and carried out in over 40 CSIR laboratories and centers spread across the country. Blood samples (6 ml) were collected in EDTA vials from each participant and analyzed on site or transported to CSIR-IGIB, New Delhi for Analysis. Elecsys Anti-SARS-CoV-2 kit from Roche Diagnostics was used to detect antibodies to SARS-CoV-2 NucleoCapsid antigen. It is a qualitative kit which was used for screening and a Cut-off index COI >1 was considered seropositive. Positive samples were further tested for quantitative antibody titers using the same manufacturer's kit directed against the spike protein (S-antigen). An antibody levels >0.8 U/ml was considered sero-positive as per manufacturer's protocol. The detection range of this kit is from 0.4 U/ml to 250 U/ml. For samples, where values of >250 U/ml were obtained; appropriate dilutions were made. Neutralizing antibody (NAb) response directed against the spike protein (RBD site) was assessed using GENScript cPass kit which is a surrogatevirus neutralization test (sVNT). A value of 30% or above was considered to have neutralizing ability. sVNT neutralization assay data for Phase 1 and Phase 2 is available as Data S7 and data for subjects with and without reinfection is available as Data S8 and Data S9 respectively.

**Supplementary Text**

Epidemiological Model

The model described here builds on a previously published model of SARS-CoV-2 transmission introduced in Flaxman et al, 2020 (26), subsequently extended into a two-category framework in Faria et al, 2020 (27). Replication code is available at https://github.com/ImperialCollegeLondon/Delta_Variant_Delhi (DOI:10.5281/zenodo.5524442).

**Model Specification** The model describes two categories, denoted $s \in \{1,2\}$. The population-unadjusted reproduction number for the first category is defined as

$$R_{s=1,t} = \mu_0 \, 2\, \sigma(X_t)\,, \qquad 1$$

where $\mu_0$ is a scale parameter (3.3), $\sigma$ is a logistic function, and $X_t$ is an second-order autoregressive process with weekly time innovations, as specified in earlier work (28). The population-unadjusted reproduction number of the second category is modelled as

$$R_{s=2,t} = \rho \mathbf{1}_{[t_2,\infty)} R_{1,t}\,, \qquad 2$$

with

$$\rho \sim \text{Gamma}(5,5) \in [0,\infty)\,, \qquad 3$$

where $\rho$ is a parameter defining the relative transmissibility of category 2 compared to category 1 and $\mathbf{1}_{[t_2,\infty)}$ is an indicator function taking the value of 0 prior to $t_2$, and 1 thereafter, highlighting that category 2 does not contribute to the observed epidemic evolution before its emergence. The prior for $\rho$ is chosen because it weakly informative, setting 90% of prior mass a between × 0.4 and × 1.8 increase in transmissibility, while maintaining a neutral to conservative default in the context of increased transmissibility since it has a mean of × 1 and median of × 0.9.

Infections arise for each category according to a discrete renewal process (29,30)

$$i_{s,t} = \left(1 - \frac{n_{s,t}}{N}\right) R_{s,t} \sum_{\tau<t} i_{s,\tau}\, g_{t-\tau}\,, \qquad 4$$

where $N$ is the total population size, $n_{s,t}$ is the total extent of population immunity to category $s$ present at time $t$, and $g$ is the generation interval distribution.

The susceptible depletion term for category $s$ is modelled as

$$n_{s,t} = \sum_{\tau<t} i_{s,\tau}\, W_{t-\tau} + \beta_s\left(1 - \alpha_{s,t}\right) \sum_{\tau<t} i_{\backslash s,\tau}\, W_{t-\tau}\,. \qquad 5$$

where $\backslash s$ denotes not-$s$, under assumptions of symmetric cross-immunity with prior

$$\beta \sim \text{Beta}(2,1)\,. \qquad 6$$

Immune escape or the evasion of cross-immunity of Delta, as reported in the analysis, is defined as the complement of the cross-immunity parameter, that is $(1-\beta)$. The prior for $\beta$ has been chosen to reflect our default assumption that the mostly likely scenario is no evasion of cross-immunity. $W_{t-\tau}$ is the time-dependent waning of immunity elicited by previous infection, which is modelled as a Rayleigh survival-type function with Rayleigh parameter of sigma = 310, which produces 50% of individuals still immune after 1 year. The cross-immunity susceptible term $\alpha_{s,t}$ is modelled as

$$\alpha_{s,t} = \frac{(1-\beta_s)\sum_{\tau<t} i_{s,\tau} W_{t-\tau}}{N-\beta_s \sum_{\tau<t} i_{s,\tau} W_{t-\tau}}\,. \qquad 7$$

Infections in Delhi are seeded for six days at the start of the epidemic from $t_1$ as

$$i_{1,t_1} \sim \text{Exponential}(1/\tau)\,, \qquad 8$$

with

$$\tau \sim \text{Exponential}(0.03)\,, \qquad 9$$

and the second category for six days from $t_2$, which is 14-02-2021 in the central scenario, as

$$i_{2,t_2} \sim \text{Normal}(1, 20^2) \in [1, \infty)\,. \qquad 10$$

Non-unit seeding of the B.1.617.2 variant and the diffuse prior represent our uncertainty in the precise date and magnitude of B.1.617.2's introduction/importation into Delhi.

The model generates deaths via the following mechanistic relationship:

$$d_t = \sum_s \text{ifr}_s \sum_{\tau<t} i_{s,\tau}\, \pi_{t-\tau}\,. \qquad 11$$

The infection fatality ratios of each of the categories ($\text{ifr}_s$) are given moderately informative priors:

$$\text{ifr}_s \sim \text{Normal}(0.25, 0.02^2) \in [0,100] \qquad 12$$

with our central estimate based on the results of Brazeau et al (31) and adjusted for the demography of the city. We allow for variation around the estimate of 0.25 however, with the prior providing some support for IFRs in the range 0.15% - 0.35%. This range is similar to estimates by Banaji for Mumbai, a city with comparable demographics, for which an IFR is reported with 95% confidence intervals of (0.15%, 0.33%) (32). A limitation of the model is the assumption of homogeneous exposure across subsets of the population.

**Likelihood component 1.** The observation model uses three types of data from four sources. In the first, the likelihood for the expected deaths $D_t$, is modelled as negative-binomially distributed,

$$D_t \sim \text{NegativeBinomial}\left(d_t(1-\omega), d_t + \frac{d_t^2}{\phi}\right), \qquad 13$$

with mortality data $d_t$ and diffuse dispersion prior

$$\phi \sim \text{Normal}(0, 5^2) \in [0, \infty) \,. \quad 14$$

and underreporting factor $\omega$, which describes the degree of death underascertainment, e.g. a value of 0.25 means 25% of COVID-19 deaths are not reported (due, e.g. to limited testing). The central scenario chosen for death underreporting is 50%, due to the wide range values reported in available literature for Delhi and India (32–37). To mitigate the effect of the uncertainty in death underreporting, sensitivity tests are carried out a range of values, $\omega$ in {10%, 33%, 50%, 66%}, to ensure inferences are robust.

**Likelihood component 2.** The second likelihood is based on genomic data from individuals where infections were sequenced and where the sequence was uploaded to GISAID. Specifically, the proportion of sequenced genomes identified as B.1.617.2 at time $t$ are modelled with a binomial likelihood

$$G_t^+ \sim \text{Binomial}(G_t^+ + G_t^-, \theta_t) \,, \quad 15$$

with positive counts for B.1.617.2 denoted $G_t^+$ and counts for lineages not belonging to B.1.617.2 recorded as $G_t^-$. The success probability for B.1.617.2 positivity is modelled as the infection ratio

$$\theta_t = \frac{\tilde{\imath}_{2,t}}{\tilde{\imath}_{1,t} + \tilde{\imath}_{2,t}} \,, \quad 16$$

where $\tilde{\imath}_{s,t}$ is given by

$$\tilde{\imath}_{s,t} = \sum_{\tau \le t} i_{s,\tau} \, \kappa_{t-\tau} \,, \quad 17$$

to account for the time varying PCR positivity displayed over the natural course of a COVID-19 infection. The distribution $\kappa$ describes the probability of being PCR positive over time following infection, and is based on (38).

**Likelihood component 3.** Serological data are incorporated in our modelling framework, using results from the survey presented in this work, and from Velumani et al. (39). The observed seropositivity $(S_t)$ on a given day, $t$, is modelled as follows

$$S_t^+ \sim \text{Binomial}\left( S_t^+ + S_t^-, \nu_t \sum_{\tau \le t} i_{s,\tau} \, C_{t-\tau} \right) , \quad 18$$

where $C_{t-\tau}$ is the cumulative probability of an individual infected on day $\tau$ having seroconverted less seroreverted by time $t$. This distribution is empirical and based on (40). The term $\nu_t$ is a multiplicative random effect specified to mitigate likely biases in the serological data

$$\nu_t = 2\sigma(Y_t) \,, \qquad Y_t \sim \mu + \eta_t \,, \qquad \mu \sim N(0,1) \,, \qquad \eta_t \sim N(0, \delta) \,, \qquad \delta \sim N^+(0,1) \,, \quad 19$$

where $\sigma$ is the logistic function.

Eq *4* can be modified to account for population effects (decreasing susceptible population over time) such that no over-shooting happens due to discretization as follows (41,42):

$$i_{s,t} = \left(N - n_{s,t}\right)\left(1 - \exp\left(-\frac{i_{s,t}}{N}\right)\right), \quad 20$$

The formula for $i_{s,t}$ is derived from a continuous time model on $[t-1, t]$. This is to avoid discrete time effects such as infections going above the total population $N$. Specifically, we assume that the infections $i(\Delta t)$ in $[t-1, t-1+\Delta t]$ are given by the differential equation $\partial i(\Delta t)/\partial\Delta t = i_t\left(1 - \left(n_{s,t} + i(\Delta t)\right)/N\right)$, which has the solution $i(1) = i_t$ as above.

**Modelling limitations** Inferences from the model are subject to multiple limitations, arising from biases in the data, and in our choices of priors. In particular, the level of underreporting in Delhi is poorly characterised. This uncertainty is mitigated by sensitivity testing, as described in the Supplementary Information. Similarly, uncertainty in the temporal waning of immunity, the date of when Delta was first introduced to Delhi, and the IFRs of SARS-CoV-2 variants in Delhi, all provide sources of bias that we currently only mitigate through sensitivity testing. Furthermore, we note serological data is likely to be systematically biased. This is partially addressed through the inclusion of an additional term in the serology likelihood that provides a multiplicative random effect.

**Computational notes** The analysis uses R version 3.6.3. Inferences are based on 2000 iterations of Hamiltonian Monte Carlo using 2 chains, with rhat statistics confirmed to be less than 1.02. The inference is performed using rstan version 2.21.2. Replication code and data are available at https://github.com/ImperialCollegeLondon/Delta_Variant_Delhi.

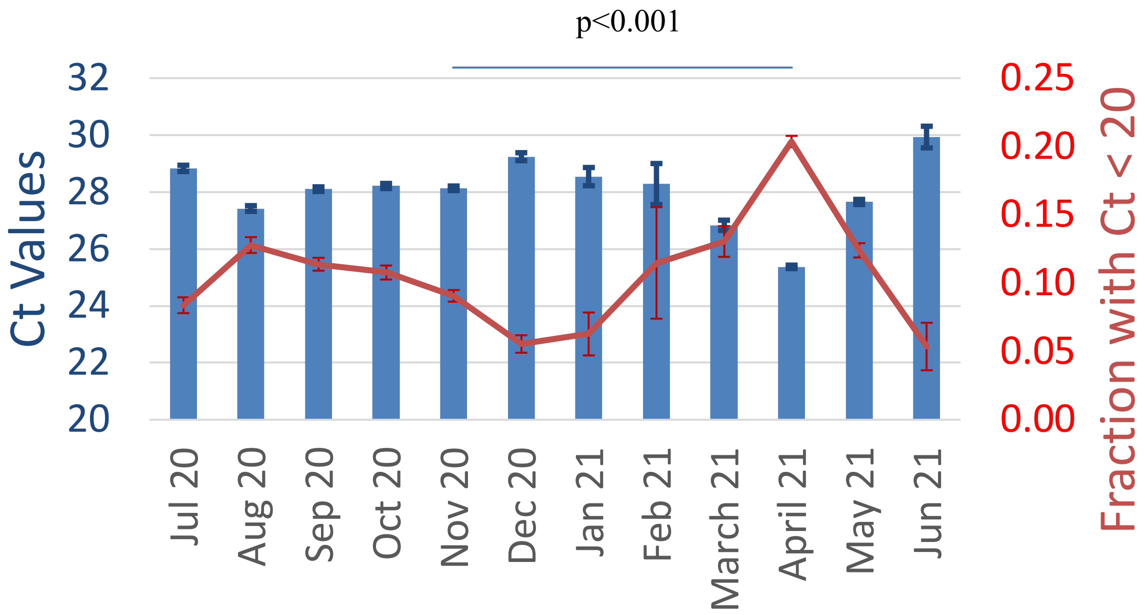


| Month / year | Positive samples (Ct <=35) |
|---|---|
| Jul 20 | 2303 |
| Aug 20 | 3332 |
| Sep 20 | 4552 |
| Oct 20 | 3666 |
| Nov 20 | 4555 |
| Dec 20 | 1281 |
| Jan 21 | 239 |
| Feb 21 | 61 |
| March 21 | 897 |
| April 21 | 9431 |
| May 21 | 3968 |
| Jun 21 | 168 |

**fig. S1. E gene Ct Values (blue) and Fraction of samples with Ct<20 (red) from July 2020 to June 2021.** Mean +/- SE (blue bar) is shown for Ct Values for monthly clinical samples testing positive on a single COBAS 6800 automated testing system at NCDC, Delhi. All Ct values less or equal to 35 were used for the analysis. Number of positive samples meeting the criteria is shown in the table. Proportion +/- SE is shown for fraction of high viral load samples (red line). Statistical significance of difference is calculated from two sample Z-tests for means and proportions between Nov 2020 and April 2021, as corresponding transmission surge time-periods. $p<0.001$

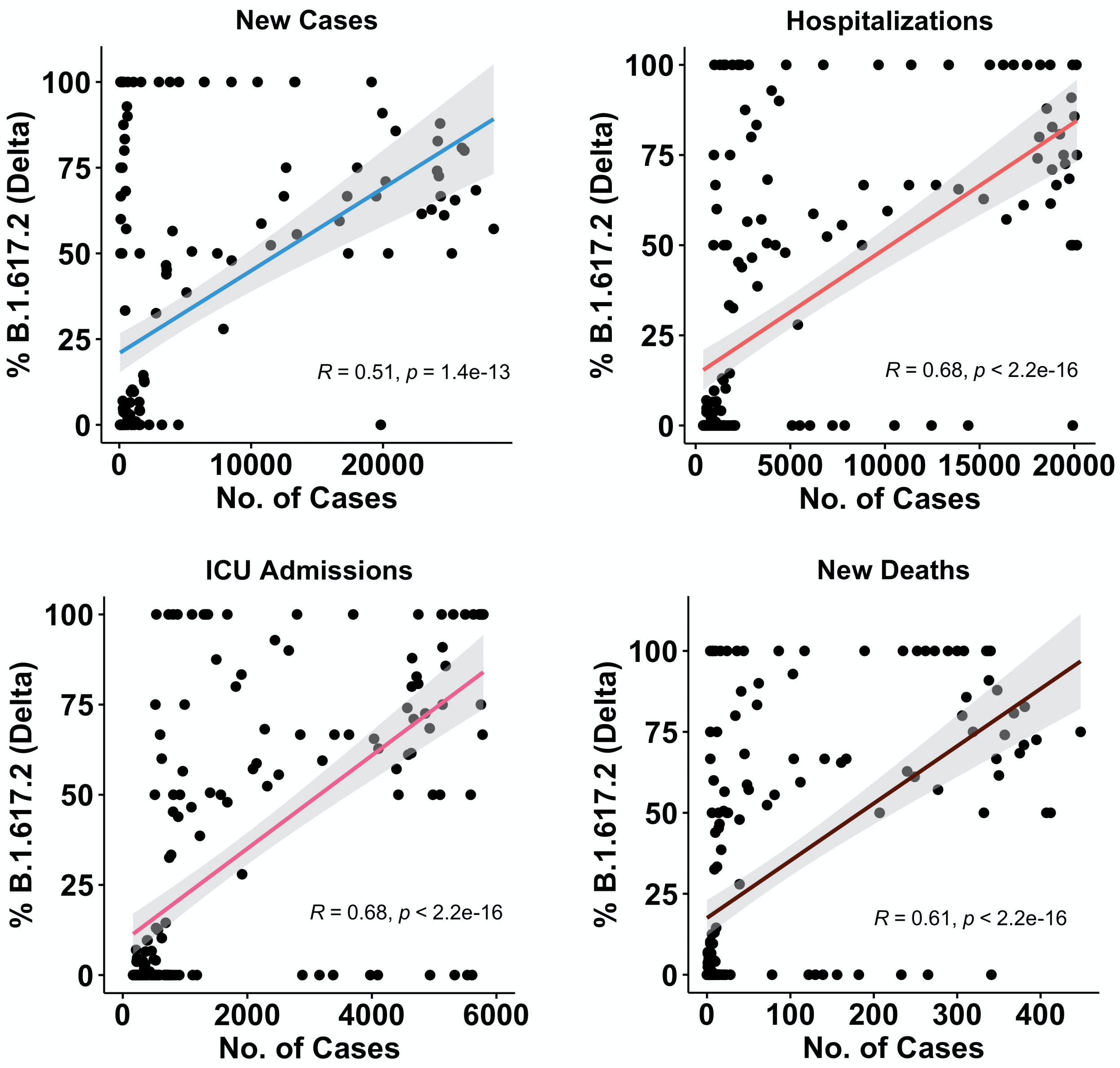


**fig. S2. Correlation plot between %Delta lineage (X-axis) and epidemiological variables for the April surge (Cases, Hospitalizations, ICU, Deaths).** Spearman coefficients were calculated for all epidemiological variables with % Delta.

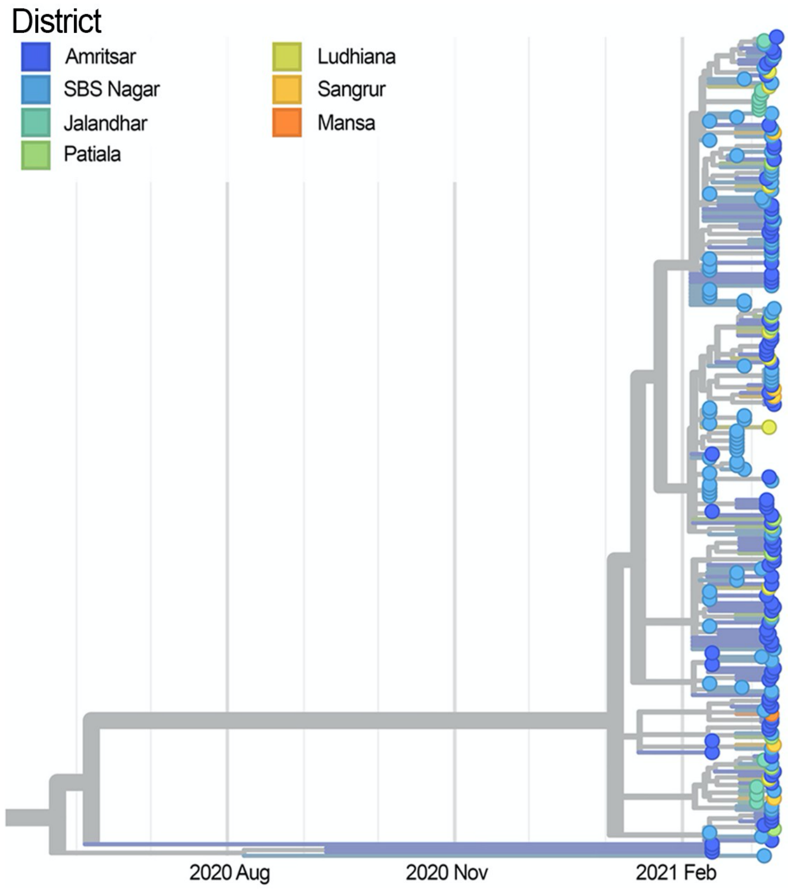


**fig. S3. Molecular signature of super-spreader event in Punjab.** Time-resolved phylogenetic tree for genome sequences from Punjab using samples collected during February-March 2021. Strong identity can be seen between sequences from various districts, corresponding to known social events during this period

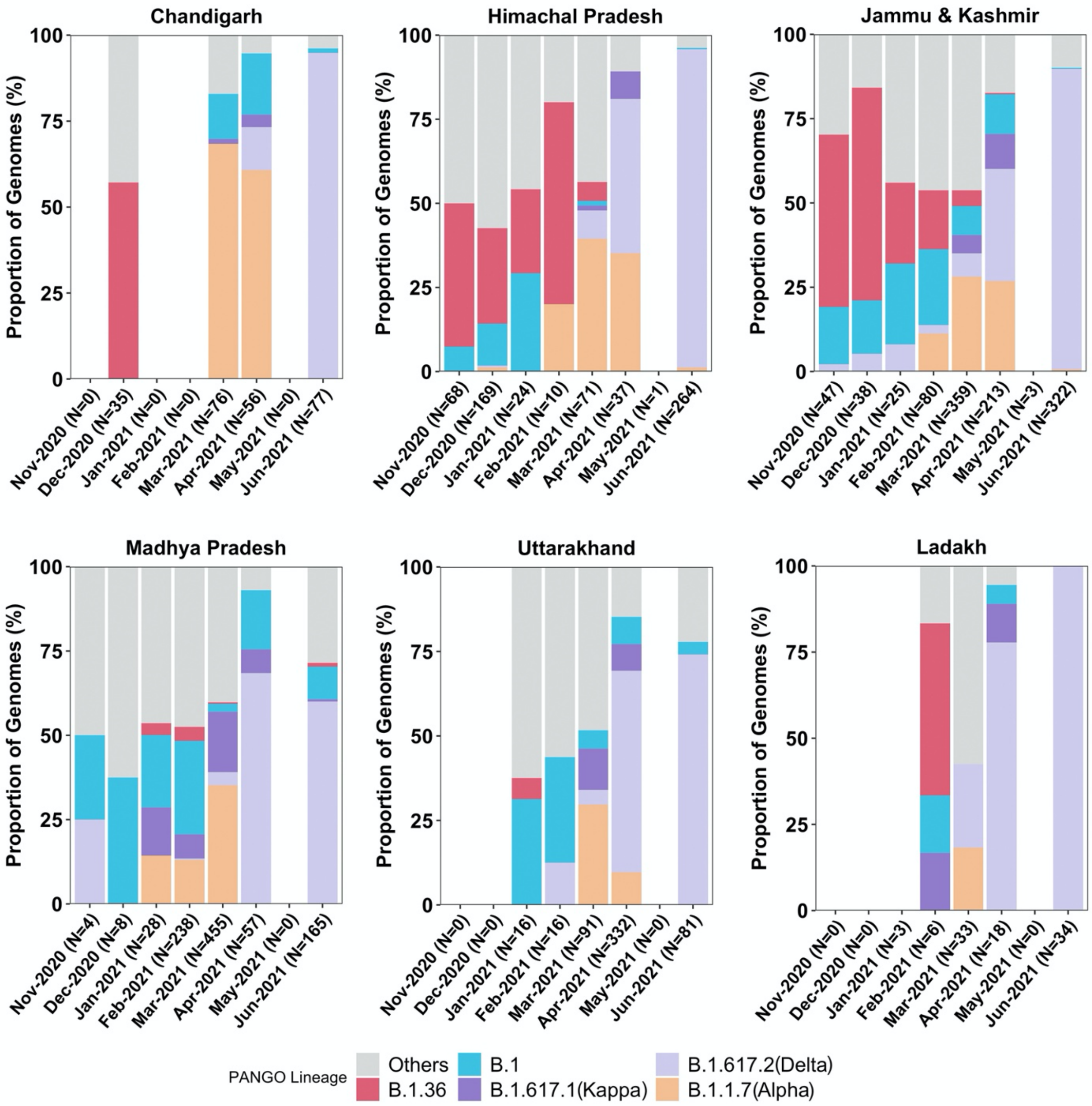


**fig. S4. Displacement of Alpha by Delta strain all over North India.** Normalized stacked bar graphs of main lineages for the states of Chandigarh, Himachal Pradesh, Madhya Pradesh, Uttarakhand, Jammu and Kashmir, and Ladakh. Outbreaks were seen in these states during April and May 2021, coincident with the rise of Delta (fig S5).

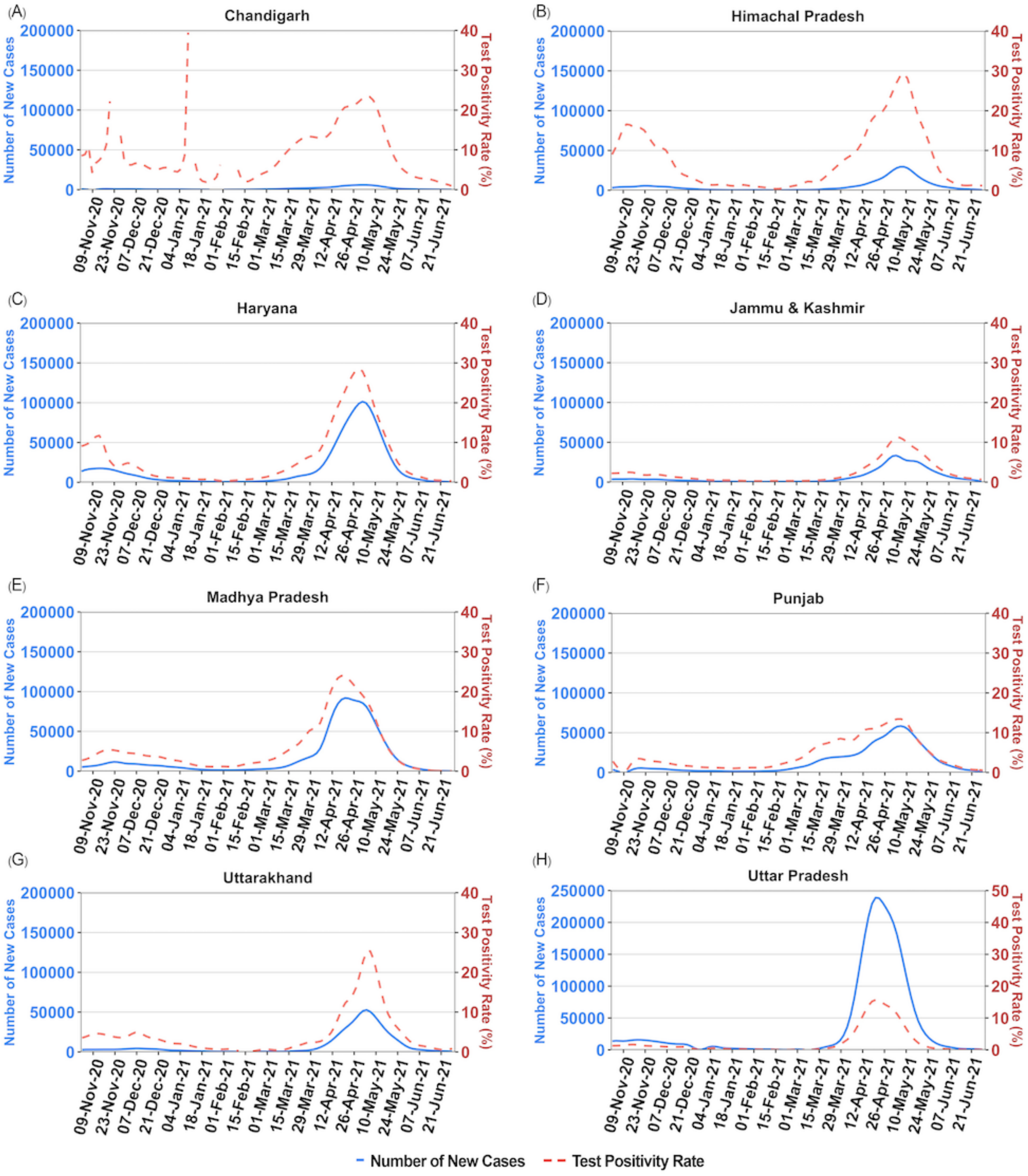


**fig. S5. Outbreaks by Delta strain all over North India.** Biweekly new cases and test positivity rates are shown for states around Delhi from November 2020 to June 2021 , coincident with the rise of Delta. Tests are a mixture of RT-PCR and antigen tests and separate positivity rates are not available. Peaks are coincident with Delta dominance on genome sequencing (Fig 3D-F and fig S4)

**Table S1. Seropositivity in the Delhi CSIR cohort.** Seropositivity is shown for Phase I to III, along with 95% CI. In the beginning of the pandemic, with strong lockdowns, higher infection rates were seen in outsourced staff providing frontline and essential services, while laboratory employees and students were relatively protected. By mid-pandemic, with lockdowns lifted by July 2020, use of private vs public transport also determined risk. By the end of the Delta wave in Delhi, all subgroups reached similar seropositivity, suggesting universally high exposures. For comparisons between serially obtained values, or between sub-groups, statistical significance of difference of proportions was calculated via the standard 2x2 Chi-statistic; $p<0.01$ was considered significant.

| | Subjects | Seropositive (anti-NC antibodies) | Seropositive proportion overall (%) (95%CI) | Seropositive proportion (%)<br>Outsourced Staff vs Staff | Seropositive proportion (%)<br>Public vs Private transport users | Date of Collection | Age Range (Median) | M:F Ratio(%) |
|---|---|---|---|---|---|---|---|---|
| Phase I | 1012 | 149 | 14.72<br>(12.6-17.06) | 24.32 vs 13.09<br>P<0.01 | 17.52 vs 13.9<br>p=NS | 22 July 2020 to 16 Sep 2020 | 18-81<br>(39) | 74.75: 25.25 |
| Phase II | 987 | 415 | 42.05<br>(38.94-45.20)<br>p<0.001 vs Phase I | 57.49 vs 35.61<br>p<0.01 | 48.29 vs 37.67<br>P<0.01 | 2 Jan 2021 to 18 Feb 2021 | 18-84<br>(35) | 68.3:31.7 |
| Phase III | 832 | 728 | 87.5<br>(85.06-89.67)<br>P<0.001 vs Phase II and Phase I | 89.33 vs 86.89<br>p=NS | 89.84 vs 86.52<br>p=NS | 22 May 2021 to 9 July 21 | 18-82<br>(33) | 67.3:32.7 |

**Table S2.**

Inferred changes in epidemiological characteristics of B.1.617.2, depending on the timing of introduction assumed in the model, and level of under-ascertainment present in Delhi mortality data. Results presented are the median, with the 50% Bayesian Credible Interval, bCI, in brackets. Note that "Immune escape" refers specifically to the escape of immunity conferred by prior infection with other variants, rather than escape from immunity acquired through vaccination. It is further important to note that immune escape and transmissibility increase inferred for B.1.617.2 are values given with reference to the composition of earlier and co-circulating variants in Delhi, from the start of the epidemic to 25 May 2021.

| | | Inferred epidemiological characteristic | |
|---|---|---|---|
| **Timing of B.1.617.2 introduction** | **Mortality under-ascertainment** | **Immune escape** | **Transmissibility increase** |
| **15 Jan 2021** | 10% | 0.34 (0.16-0.60) | 1.48 (1.35-1.57) |
| **31 Jan 2021** | 10% | 0.39 (0.18-0.59) | 1.45 (1.36-1.55) |
| **14 Feb 2021** | 10% | 0.42 (0.21-0.64) | 1.47 (1.37-1.58) |
| **28 Feb 2021** | 10% | 0.45 (0.23-0.65) | 1.55 (1.45-1.65) |
| **15 Jan 2021** | 33% | 0.29 (0.11-0.55) | 1.48 (1.35-1.60) |
| **31 Jan 2021** | 33% | 0.29 (0.10-0.55) | 1.49 (1.35-1.59) |
| **14 Feb 2021** | 33% | 0.38 (0.16-0.64) | 1.47 (1.34-1.59) |
| **28 Feb 2021** | 33% | 0.43 (0.20-0.67) | 1.54 (1.42-1.67) |
| **15 Jan 2021** | 50% | 0.15 (0.07-0.35) | 1.54 (1.40-1.62) |
| **31 Jan 2021** | 50% | 0.15 (0.07-0.33) | 1.54 (1.40-1.61) |
| **14 Feb 2021** | 50% | 0.22 (0.08-0.48) | 1.53 (1.35-1.50) |
| **28 Feb 2021** | 50% | 0.31 (0.12-0.59) | 1.56 (1.40-1.70) |
| **15 Jan 2021** | 66% | 0.43 (0.35-0.60) | 1.22 (1.11-1.30) |
| **31 Jan 2021** | 66% | 0.42 (0.35-0.55) | 1.23 (1.13-1.31) |
| **14 Feb 2021** | 66% | 0.49 (0.37-0.67) | 1.23 (1.11-1.32) |
| **28 Feb 2021** | 66% | 0.59 (0.42-0.76) | 1.28 (1.17-1.40) |

**Table S3**

Inferred changes in epidemiological characteristics of B.1.617.2, depending on prior assumptions in the model. Results presented are the median, with the 50% Bayesian Credible Interval, bCI, in brackets. Prior sensitivity analyses assume 50% underreporting in deaths.

| **Sensitivity** | **Prior** | **Inferred epidemiological characteristic** | |
|---|---|---|---|
| | | **Immune escape** | **Transmissibility increase** |
| **Cross-immunity** | Beta(1,1) | 0.43 (0.14-0.79) | 1.38 (1.21-1.58) |
| | Beta(2,1) | 0.22 (0.08-0.48) | 1.53 (1.35-1.50) |
| | Beta(3,1) | 0.12 (0.06-0.26) | 1.60 (1.49-1.67) |
| | Beta(4,1) | 0.10 (0.04-0.18) | 1.62 (1.55-1.68) |
| | Complete escape | 1 | 1.11 (1.08-1.15) |
| | Complete cross-protection | 0 | 1.71 (1.67-1.75) |
| **Timing to 50% waning** | ½ year | 0.57 (0.35-0.76) | 1.51 (1.45-1.58) |
| | 1 year | 0.22 (0.08-0.48) | 1.53 (1.35-1.5) |
| | 2 years | 0.14 (0.08-0.24) | 1.55 (1.45-1.63) |
| **Transmissibility increase** | Gamma(2,2) | 0.13 (0.06-0.29) | 1.60 (1.47-1.67) |
| | Gamma(5,5) | 0.22 (0.08-0.48) | 1.53 (1.35-1.65) |
| | Gamma(10,10) | 0.28 (0.13-0.58) | 1.48 (1.30-1.60) |
| **IFR** | N(0.125,0.02) | 0.73 (0.65-0.81) | 1.11 (1.04-1.17) |
| | N(0.25,0.02) | 0.22 (0.08-0.48) | 1.53 (1.35-1.5) |
| | N(0.5,0.02) | 0.41 (0.20-0.64) | 1.48 (1.39-1.58) |

## List of INSACOG Consortium Members

| NAME | AFFILIATION | EMAIL ID |
|---|---|---|
| Vinay Nandicoori | CSIR Centre for Cellular and Molecular Biology | vinaykn@ccmb.res.in |
| Karthik Bharadwaj Tallapaka | CSIR Centre for Cellular and Molecular Biology | karthikt@ccmb.res.in |
| Divya Tej Sowpati | CSIR Centre for Cellular and Molecular Biology | tej@ccmb.res.in |
| K Thangaraj | Centre for DNA Fingerprinting and Diagnostics | director@cdfd.org.in |
| Murali Dharan Bashyam | Centre for DNA Fingerprinting and Diagnostics | bashyam@cdfd.org.in |
| Ashwin Dalal | Centre for DNA Fingerprinting and Diagnostics | adalal@cdfd.org.in |
| Sridhar Sivasubbu | CSIR Institute of Genomics and Integrative Biology | sridhar@igib.in |
| Vinod Scaria | CSIR Institute of Genomics and Integrative Biology | vinods@igib.in |
| Ajay Parida | Institute of Life Sciences | drajayparida@gmail.com |
| Sunil K Raghav | Institute of Life Sciences | sunilraghav@ils.res.in |
| Punit Prasad | Institute of Life Sciences | punit@ils.res.in |
| Apurva Sarin | Institute For Stem Cell Science and Regenerative Medicine/National Centre for Biological Sciences | sarina@instem.res.in |
| Satyajit Mayor | Institute For Stem Cell Science and Regenerative Medicine/National Centre for Biological Sciences | mayor@ncbs.res.in |
| Uma Ramakrishnan | Institute For Stem Cell Science and Regenerative Medicine/National Centre for Biological Sciences | uramakri@ncbs.res.in |
| Dasaradhi Palakodeti | Institute For Stem Cell Science and Regenerative Medicine/National Centre for Biological Sciences | dasaradhip@instem.res.in |
| Aswin Sai Narain Seshasayee | Institute For Stem Cell Science and Regenerative Medicine/National Centre for Biological Sciences | aswin@ncbs.res.in |

| | | |
|---|---|---|
| Manoj Bhat | National Centre For Cell Science | manojkbhat@nccs.res.in |
| Yogesh Shouche | National Centre For Cell Science | yogesh@nccs.res.in |
| Ajay Pillai | National Centre For Cell Science | ajayp@nccs.res.in |
| Tanzin Dikid | National Centre for Disease Control | tanzindikid@gmail.com |
| Saumitra Das | National Institute of Biomedical Genomics | sdas@nibmg.ac.in |
| Arindam Maitra | National Institute of Biomedical Genomics | am1@nibmg.ac.in |
| Sreedhar Chinnaswamy | National Institute of Biomedical Genomics | sc2@nibmg.ac.in |
| Nidhan Kumar Biswas | National Institute of Biomedical Genomics | nkb1@nibmg.ac.in |
| Anita Sudhir Desai | National Institute of Mental Health and Neuro Sciences | anitasdesai@gmail.com |
| Chitra Pattabiraman | National Institute of Mental Health and Neuro Sciences | pattabiraman.chitra@gmail.com |
| M. V. Manjunatha | National Institute of Mental Health and Neuro Sciences | manjuvswamy@gmail.com |
| Reeta S Mani | National Institute of Mental Health and Neuro Sciences | drreeta@gmail.com |
| Gautam Arunachal Udupi | National Institute of Mental Health and Neuro Sciences | udupi@nimhans.ac.in |
| Priya Abraham | National Institute of Virology | director.niv@icmr.gov.in |
| Potdar Varsha Atul | National Institute of Virology | potdarvarsha9@gmail.com |
| Sarah S Cherian | National Institute of Virology | cheriansarah@yahoo.co.in |

**Data S1 (separate file)**

GISAID accession IDs, date of collection and lineages of the samples used to calculate lineage proportions in different states from Datasets A and B.

**Data S2 (separate file)**

Cases, Tests, Hospitalizations, ICU Admissions, Deaths data for Delhi from the state level database maintained by NCDC.

**Data S3 (separate file)**

Acknowledgement table for genomes accessed from GISAID.

**Data S4 (separate file)**

Details of 24 post-vaccination samples

**Data S5 (separate file)**

Raw Ct Values for July 2020-June 2021 for Delhi

**Data S6 (separate file)**

Serosurvey data for the three phases conducted in Delhi (Fig. 2A)

**Data S7 (separate file)**

sVNT assay data Phase I vs Phase II serosuvey (Fig. 2D)

**Data S8 (separate file)**

Data for Subjects with Reinfection (Fig. 2C)

**Data S9 (separate file)**

Data for Subjects without Reinfection (Fig. 2D)
